# Triterpenoids From *Kadsura coccinea* With Their Anti-inflammatory and Inhibited Proliferation of Rheumatoid Arthritis-Fibroblastoid Synovial Cells Activities

**DOI:** 10.3389/fchem.2021.808870

**Published:** 2021-12-09

**Authors:** Yu-pei Yang, Yu-qing Jian, Yong-bei Liu, Muhammad Ismail, Qing-ling Xie, Huang-he Yu, Bin Wang, Bin Li, Cai-yun Peng, Bin Liu, Rong-yong Man, Wei Wang

**Affiliations:** ^1^ TCM and Ethnomedicine Innovation and Development International Laboratory, Innovative Materia Medica Research Institute, School of Pharmacy, Hunan University of Chinese Medicine, Changsha, China; ^2^ Department of Chemistry, Karakoram International University, Gilgit, Pakistan; ^3^ Hunan Province Key Laboratory of Plant Functional Genomics and Developmental Regulation, College of Biology, Hunan University, Changsha, China; ^4^ Clinic Experimental Research Center, The First People’s Hospital of Huaihua, Huaihua, China

**Keywords:** schisandraceae, *Kadsura coccinea*, heilaohu, triterpenoids, anti-inflammatory, Tujia ethnomedicine

## Abstract

One new 3,4-*seco*-17,13-friedo-lanostane triterpenoid heilaohuacid A (**1**), one new 3,4-*seco*-17,14-friedo-lanostane triterpenoid heilaohuacid B (**2**), five new 3,4*-seco*-lanostane triterpenoids heilaohuacids C-D (**3–4**) and heilaohumethylesters A-C (**7–9**), one new 3,4-*seco*-cycloartane triterpenoid heilaohuacid E (**5**), and one new *intact*-lanostane triterpenoid heilaohuacid F (**6**), together with twenty-two known analogues (**10–31**), were isolated from heilaohu. Their structures were determined using HR-ESI-MS data, 1D and 2D NMR spectra, ^13^C NMR calculations, and electronic circular dichroism (ECD) calculations. Heilaohuacids A and B (**1** and **2**) contain a 3,4-*seco* ring A and unprecedented migration of Me-18 from C-13 to C-17 or C-14 to C-18. This type of lanostane triterpenoid derivatives was rarely reported so far. More importantly, all compounds against inflammatory cytokines IL-6 and TNF-α levels on LPS-induced RAW 264.7 macrophages were evaluated, and compounds **4** and **31** significantly inhibited the release level of IL-6 with IC_50_ values of 8.15 and 9.86 μM, respectively. Meanwhile, compounds 17, 18, and 31 significantly inhibited proliferation of rheumatoid arthritis-fibroblastoid synovial (RA-FLS) cells *in vitro* with IC_50_ values of 7.52, 8.85, and 7.97 μM, respectively.

## Introduction

**FIGURE 1:**
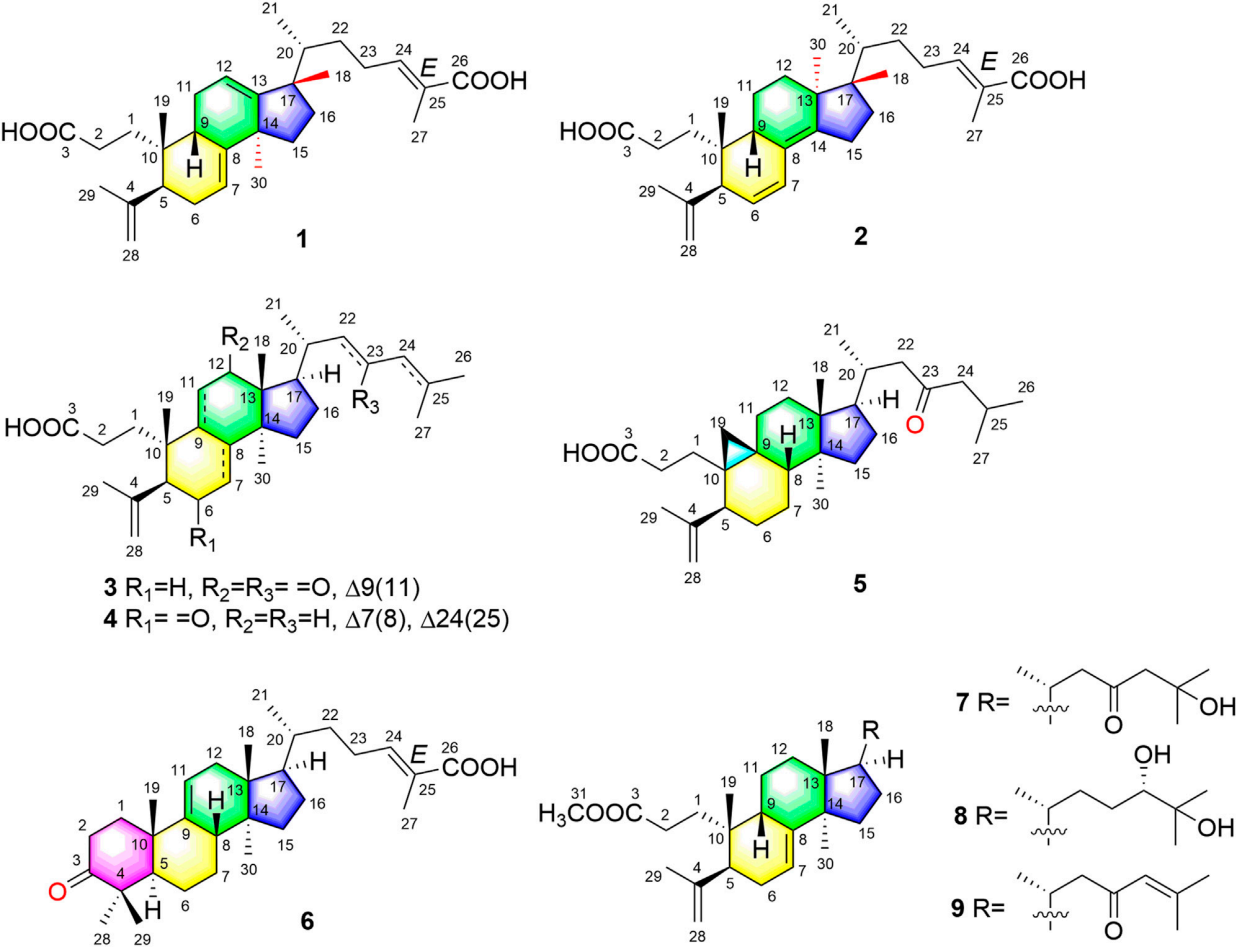
Structures of new triterpenoid compounds **1–9**.

Schisandraceae is a famous medicinal plant family, comprising only two genera *Kadsura* and *Schisandra*. This family of medicinal plants are enriched with lanostane, cycloartane, and schinortriterpenoid (SNT) triterpenoids (Shi et al., 2015), which possesses remarkable anti-inflammation (Yu H.-H. et al., 2019), cytotoxicity (Gao et al., 2008), and anti-HIV activities (Yang et al., 2010). The dried roots of *Kadsura coccinea* called “heilaohu” in Chinese have been used in Tujia ethnomedicine to treat rheumatic arthritis (RA), gastric and duodenal ulcers, etc. (Xu et al., 2019). In the past decade, tremendous development has been made on the chemistry and biological properties of *K. coccinea*, which have yielded a number of dibenzocyclooctadiene lignans (Liu et al., 2014) and lanostane triterpenoids (Hu et al., 2016). In our early research studies, we have reported the isolation and structural elucidation of several new triterpenoids, sesquiterpenoids, and lignans from *K. coccinea* and other species of the same genus (Liu et al., 2018; Cao et al., 2019; Shehla et al., 2020). Furthermore, *Kadsura heteroclita* (Roxb.) Craib. in the same genus *Kadsura* displayed good anti-rheumatoid arthritis, anti-inflammatory, and analgesic effects (Yu H.-H. et al., 2019; Yu H. et al., 2019). Tujia ethnomedicine heilaohu also possess anti-RA agents. Herein, the phytochemistry, anti-RA-FLS cells, and anti-inflammation activity investigations on structurally interesting triterpenoids from the roots of *K. coccinea* were carried out. One new 3,4-*seco*-17,13-friedo-lanostane triterpenoid heilaohuacid A (**1**), one new 3,4-*seco*-17,14-friedo-lanostane triterpenoid heilaohuacid B (**2**), five new 3,4*-seco*-lanostane triterpenoids heilaohuacids C-D (**3–4**) and heilaohumethylesters A-C (**7–9**), one new 3,4-*seco*-cycloartane triterpenoid heilaohuacid E (**5**), and one new *intact*-lanostane triterpenoid heilaohuacid F (**6**) (Figure 1), together with twenty-two known analogues (**10–31**), were isolated from heilaohu (Supplementary Figure S1). Their structures were determined by various chromatographic and spectroscopic techniques. All compounds were evaluated for their anti-inflammatory effects and inhibited proliferation of RA-FLS cell activity. Herein, the isolation, structural elucidation of new compounds **1–9**, along with *in vitro* anti-inflammatory and inhibited proliferation of RA-FLS cell activity screening will be reported.

**GRAPHICAL ABSTRACT:**
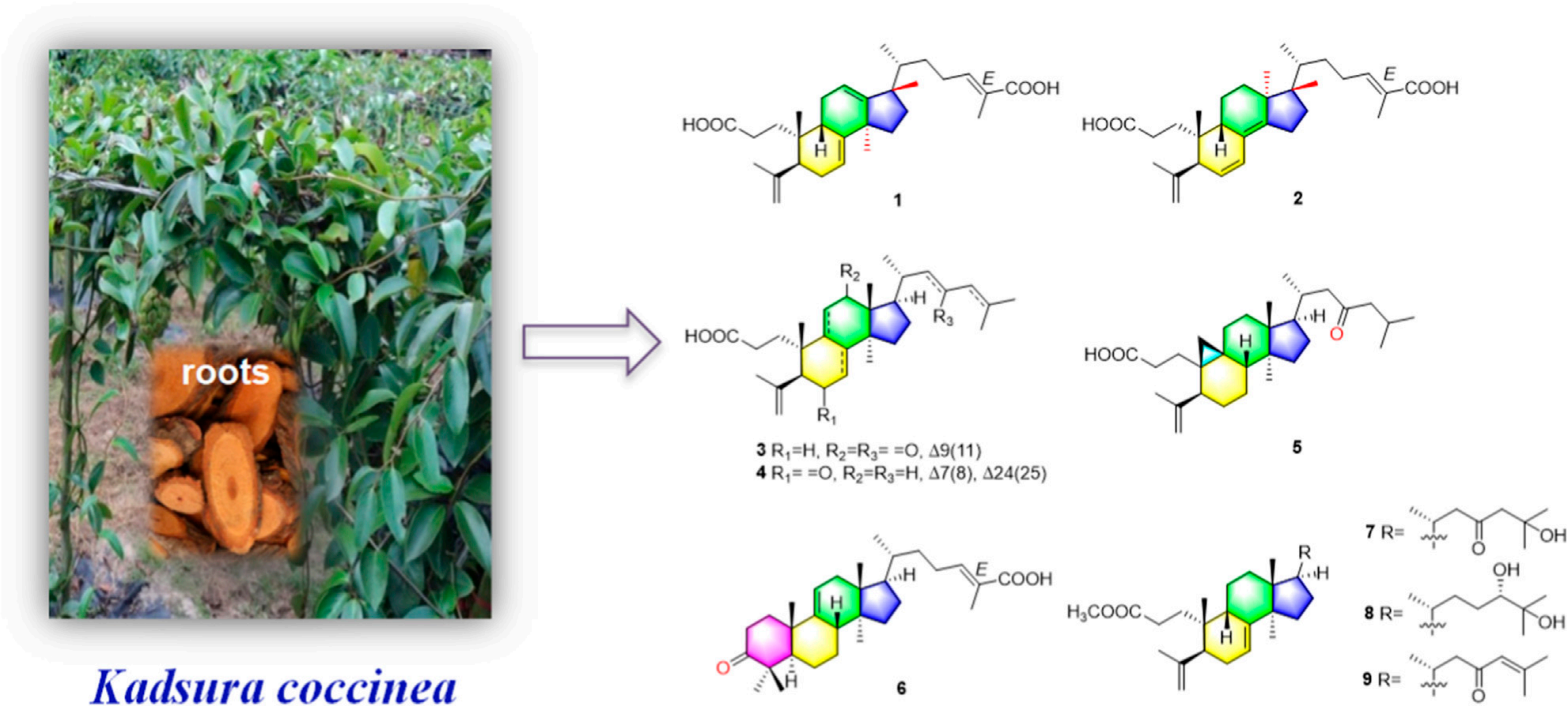


## Results and Discussion

**FIGURE 2:**
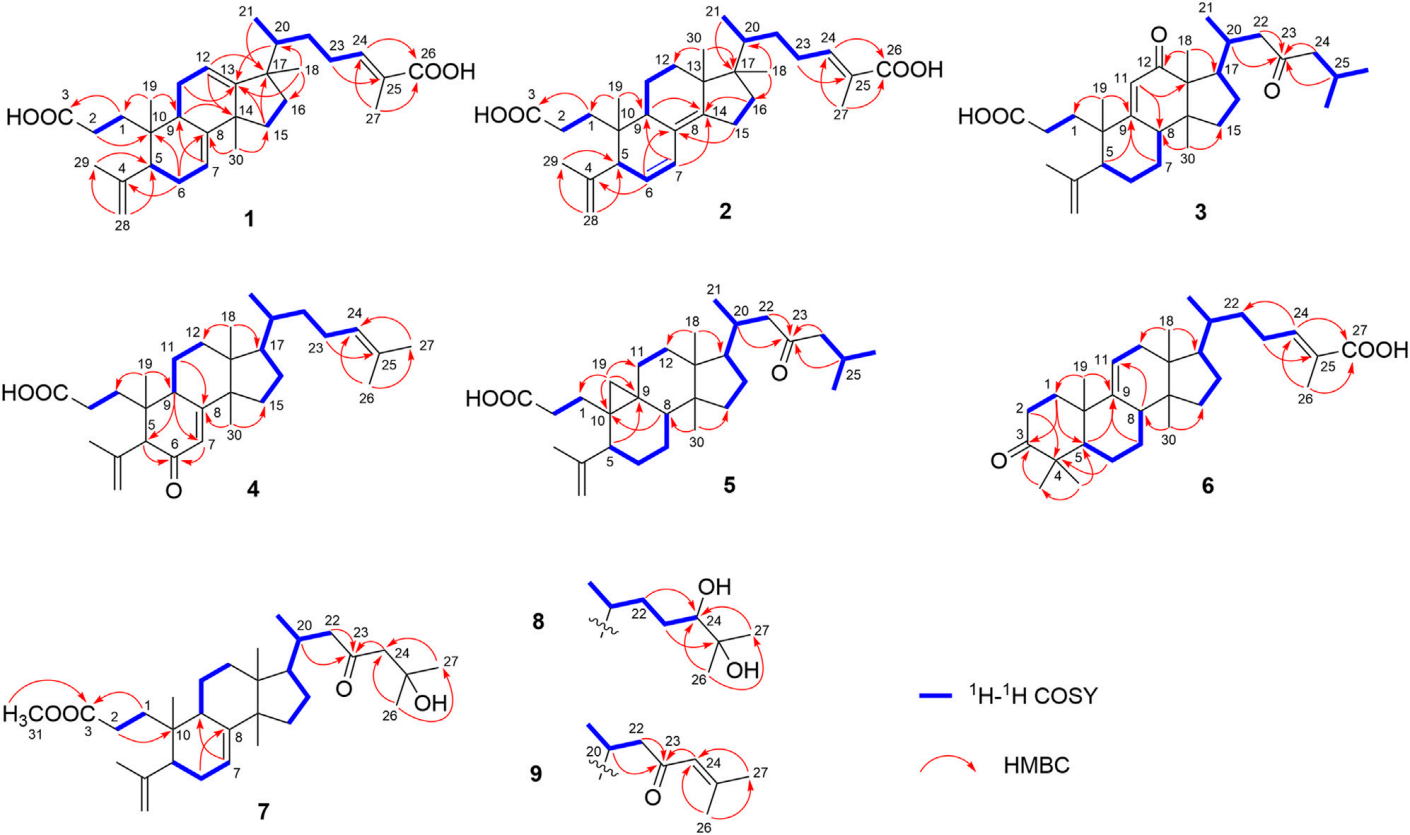
Key ^1^H–^1^H COSY and HMBC correlations of compounds 1–9.

Compound **1** was derived as a white amorphous powder, and the molecular formula C_30_H_44_O_4_, with 9 degrees of unsaturation, was deduced from the HR-ESI-MS at 491.3143 [M + Na]^+^ (calcd. for 491.3137, C_30_H_44_O_4_Na) and its ^13^C NMR data. The ^1^H NMR data showed typical resonances for five tertiary methyls (*δ*_H_ 0.92, 0.92, 1.84, 1.74, and 1.06, each 3H, s), one double methyl (*δ*_H_ 0.88, d, *J* = 6.8 Hz), and five olefinic protons (*δ*_H_ 5.52, 5.58, 6.89, 4.74, and 4.78, each 1H). The ^13^C NMR data with the aid of DEPT and HSQC spectra revealed the presence of six methyls, nine methylenes, six methines (three tri-substituted double bond), and nine quaternary carbons (two carboxyl groups). Detailed analyses of ^1^H NMR, ^13^C NMR, DEPT, and HSQC spectra enabled all proton resonances of **1** to be attributed to their respective carbons (Tables 1, 3). The planar structure of **1** was elucidated by interpretation of HMBC and ^1^H-^1^H COSY spectra. The ^1^H-^1^H COSY spectrum of **1** revealed the presence of five independent spin systems (H_2_-1/H_2_-2, H-5/H-6/H-7, H-9/H_2_-11/H-12, H_2_-15/H_2_-16, and H_3_-21/H-20/H_2_-22/H_2_-23/H-24). The HMBC cross-peaks (Figure 2) of H-1 with C-3, H_2_-28 with C-5 and C-29, H_2_-6 and H_3_-30 with C-8, H_2_-11 with C-13, H-12 with C-13, C-14, and C-17, H_2_-23 and H-24 with C-24, C-25, and C-27, and H_3_-27 with C-24 and C-26 constructed that the ring A was *seco* between C-3 and C-4, as well as the presence of a carboxylic acid at C-26, and three double bone groups at C-7/C-8, C-12/C-13, and C-24/C-25, respectively. Importantly, the HMBC correlations from H_3_-18 to C-17, C-16, and C-20 indicated that an unprecedented migration of Me-18 from C-13 to C-17 occurred. Accordingly, the planar structure of 1 was determined as a novel 3,4-*seco*-17,13-friedo-lanostane triterpenoid derivative with a tricyclic skeleton. To the best of our knowledge, this type of lanostane triterpenoid with A ring 3,4-*seco* along with Me-18 migration from C-13 to C-17 was rarely reported before.

**TABLE 1:** ^1^H NMR data of compounds **1–4** in CDCl_3_ (600 MHz, *δ* in ppm, *J* in Hz).

Positions	1	2	3	4
1	a 1.58 m; b 1.76 m	1.60 m	a 1.92 m; b 2.05 m	1.71 m
2	2.33 m	2.29 m	2.45 m	a 2.23 m
				b 2.27 m
5	2.08 d (6.0)	2.61 d (5.5)	2.21 m	2.82 m
6	a 1.99 m	5.39 dd (9.9, 5.5)	a 1.39 m	—
	b 2.30 m		b 1.70 m	
7	5.52 d (3.0)	6.21 d (9.9)	a 1.37 m	5.92 d (2.5)
			b 1.67 m	
8	—	—	2.62 m	—
9	2.06 m	2.41 m	—	3.17 m
11	a 1.89 m	a 1.56 m	5.76 s	1.73 m
	b 2.02, m	b 1.69 m		
12	5.58 dd (8.0, 2.8)	a 1.63 m	—	a 1.55 m
		b 1.69 m		b 1.64 m
15	a 1.27 m	a 2.27 m	a 1.49 m	a 1.79 m
	b 1.76 m	b 2.37 m	b 1.66 m	b 1.90 m
16	a 1.62 m	a 1.49 m	a 1.35 m	a 1.32 m
	b 1.74 m	b 1.66 m	b 1.96 m	b 2.01 m
17	—	—	2.18 m	1.56 m
18	0.92 s	1.00 s	1.08 s	0.81 s
19	0.92 s	0.86 s	1.20 s	0.97 s
20	1.46 m	1.67 m	1.96 m	1.40 m
21	0.88 d (6.8)	0.84 d (6.7)	0.96 d (6.4)	0.91 d (6.5)
22	a 1.15 m	a 1.24 m	a 2.30 m	a 1.05 m
	b 1.81 m	b 1.75 m	b 2.42 m	b 1.45 m
23	a 2.13 m	2.26 m	—	a 1.88 m
	b 2.30 m			b 2.05 m
24	6.89 m	6.92 m	2.28 m	5.09 t (6.9)
25	—	—	2.15 m	—
26	—	—	0.93 d (6.8)	1.61 s
27	1.84 s	1.85	0.93 d (6.8)	1.69 s
28	4.74 brs 4.78 brs	4.75 brs 4.95 brs	4.77 brs 4.94 brs	4.85 brs 5.03 brs
29	1.74 s	1.78 s	1.80 s	1.87 s
30	1.06 s	0.65 s	0.79 s	1.11 s

**FIGURE 3:**
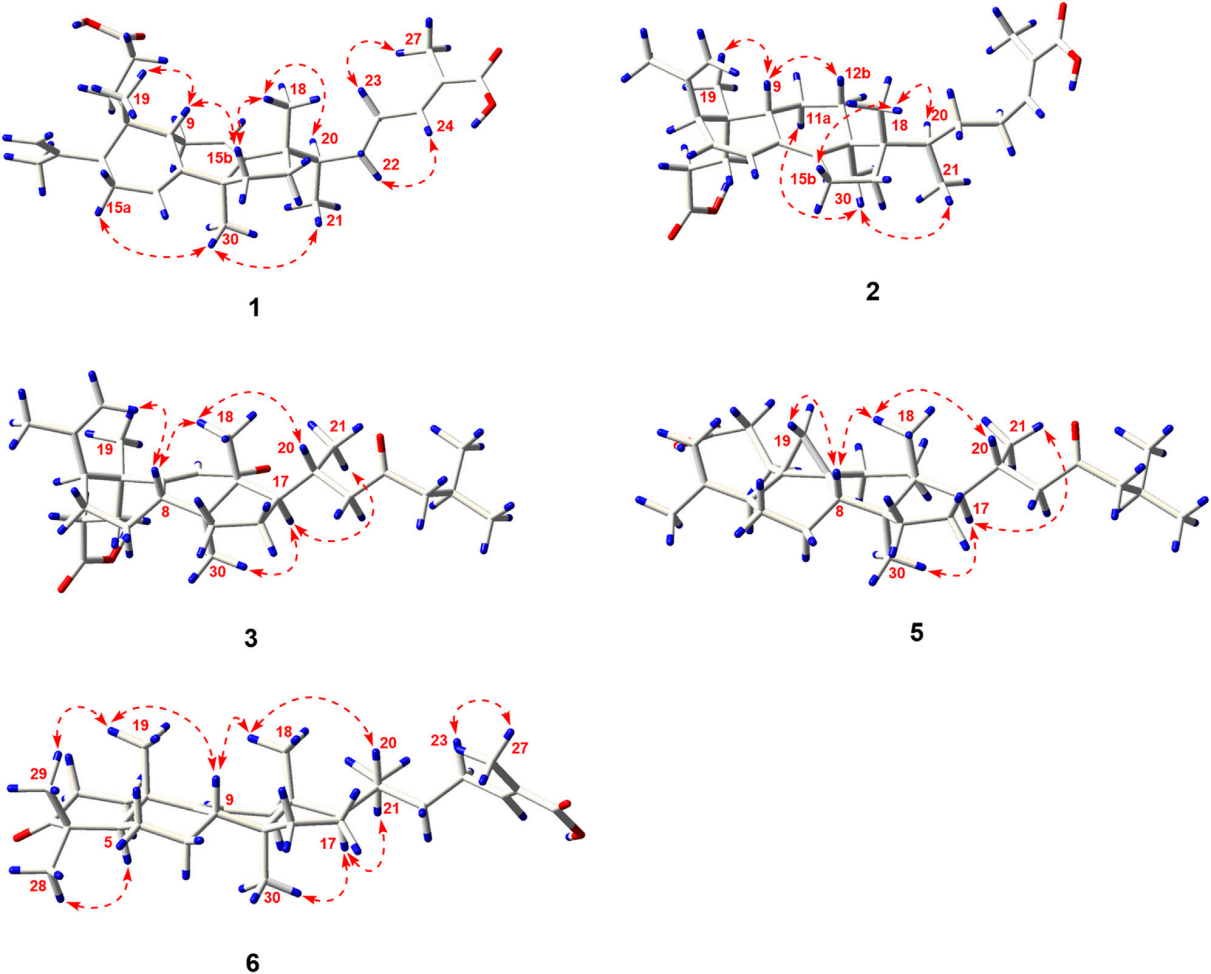
Key ROESY correlations of new compounds **1–3**, **5**, and **6**.

**FIGURE 4:**
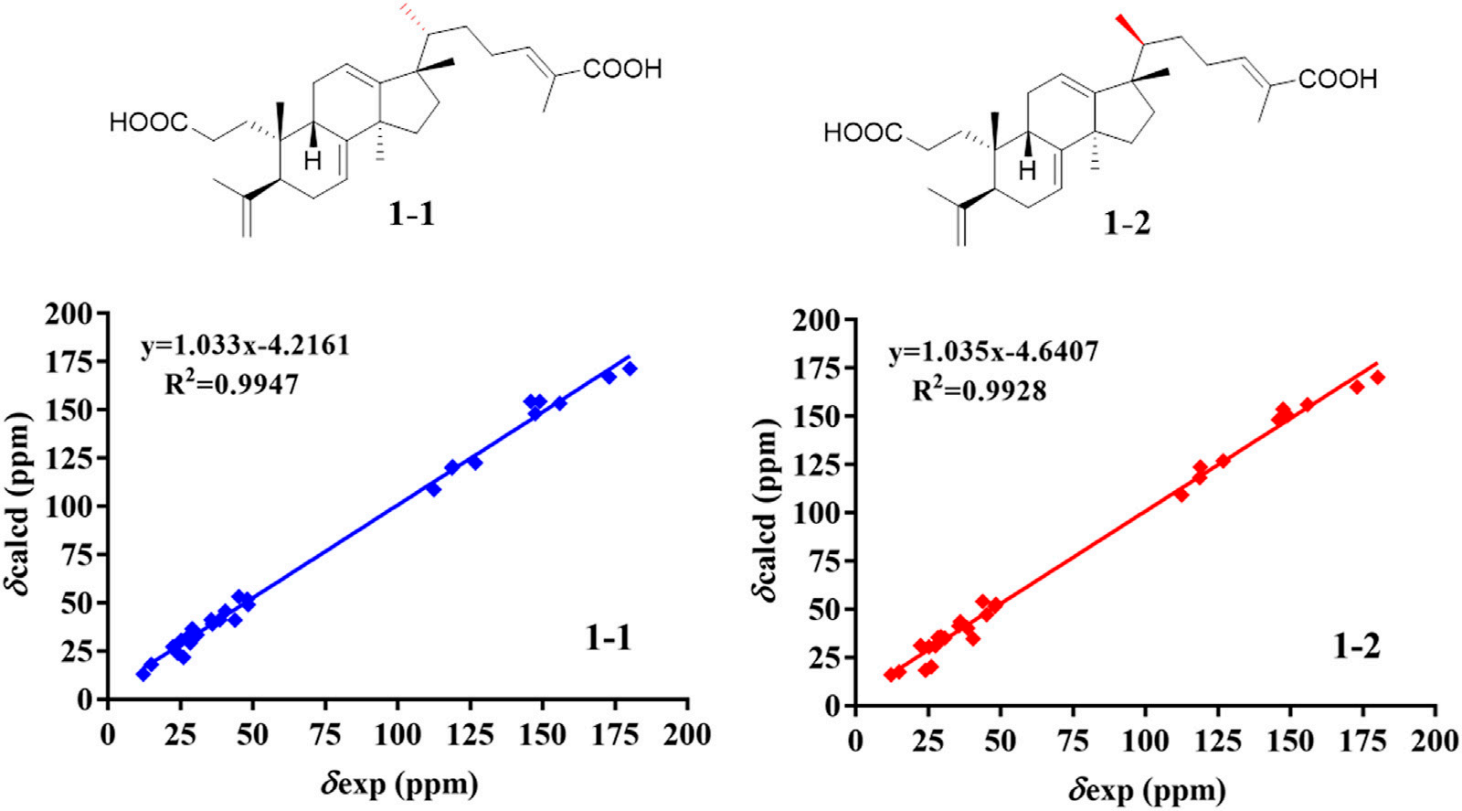
NMR calculations of compound **1**.

**FIGURE 5:**
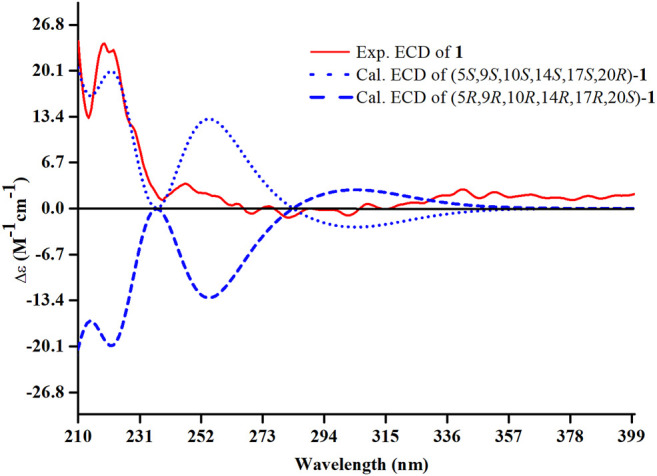
Experimental and calculated ECD spectra of compound **1**.

The relative configuration of **1** was ascertained through interpretation of its ROESY spectrum. H_3_-19 was assigned a *β*-orientation. The NOE correlations (Figure 3) of H_3_-19 with H-9 and H-15b, H_3_-18 with H-15b and H-20, and H-15a with H_3_-30; in addition, there are no NOE correlations between H-9 and H_3_-30, suggesting that H-9 and H_3_-18 were *β*-oriented, and H_3_-30 was *a*-oriented. Lack of NOE correlations of H_3_-27 with H-24 indicated that the double bond between C-24 and C-25 has a *trans*-configuration. Additionally, the relative configuration of C-20 was investigated by the TDDFT to calculate the ^13^C NMR data for **1-1** and **1-2**. As shown in Figure 4, the ^13^C NMR chemical shifts of isomers were calculated at the mPW1PW91/6-31+G** level. The calculation result of **1-1** (*R* ^2^ = 0.9947) matched the experimental data better than **1-2** (*R* ^2^ = 0.9928), which indicated that H_3_-21 has an *a*-orientation. To further elucidate its absolute configuration, the electronic circular dichroism (ECD) spectrum of **1** was recorded in MeOH, and it showed a good agreement with the calculated ECD spectrum of the (5*, 9, 10, 14*, 17*S*, and 20*R*) model (Figure 5), which supported the absolute configuration of **1** should be identical to 5*, 9, 10, 14*, 17*S*, and 20*R*. Thus, compound **1** was elucidated as a novel 3,4-*seco*-17,13-friedo-lanostane triterpenoid and named heilaohuacid A, accordingly.

Heilaohuacid B (**2**) had the same molecular formula of C_30_H_44_O_4_ as compound **1**, based on the HR-ESI-MS at *m/z* 467.3105 [M-H]^−^ (calcd. for 467.3161, C_30_H_43_O_4_). Its ^1^H NMR spectrum displayed signals characteristic of five olefinic protons at *δ*_H_ 6.92 (m, H-24), 6.21 (d, *J* = 9.9 Hz, H-7), 5.39 (dd, *J* = 9.9, 5.5 Hz, H-6), 4.95 (br s, Hb-28), and 4.75 (br s, Ha-28). The ^1^H NMR spectrum also displayed one methyl doublet at *δ*_H_ 0.84 (d, *J* = 6.7 Hz, H_3_-21) and five methyl groups at *δ*_H_ 1.85 (H_3_-27), 1.78 (H_3_-29), 1.00 (H_3_-18), 0.86 (H_3_-19), and 0.65 (H_3_-30). The ^13^C NMR and DEPT spectra data (Table 3) of **2** highlighted the presence of 30 carbon signals, including six methyls, nine methylenes, six methines, and nine quaternary carbons. This confirmed that compound **2** was a lanostane triterpenoid derivative with tricyclic skeleton. The HMBC cross-peaks (Figure 2) of H-1 with C-3, H_2_-28 with C-5 and C-29, H-6 with C-4 and C-8, H-7 with C-9 and C-14, H-9 and H_2_-15 with C-8 and C-14, H_2_-23 and H-24 with C-24, C-25, and C-26, and H_3_-27 with C-24 and C-26 constructed that the ring A was *seco* between C-3 and C-4, as well as the presence of a carboxylic acid at C-26, and three double bone groups at C-6/C-7, C-8/C-14, and C-24/C-25, respectively. Additionally, the HMBC correlations from H_3_-18 to C-17, C-16, and C-20, and from H_3_-30 to C-13, C-12, and C-17 indicated that interesting migrations of Me-18 from C-13 to C-17 and Me-30 from C-14 to C-13 took place. To date, this type of lanostane triterpenoid derivative was rarely reported (Lavoie et al., 2013). Accordingly, the planar structure of **2** was determined as a 3,4-*seco*-17,14-friedo-lanostane triterpenoid.

**FIGURE 6:**
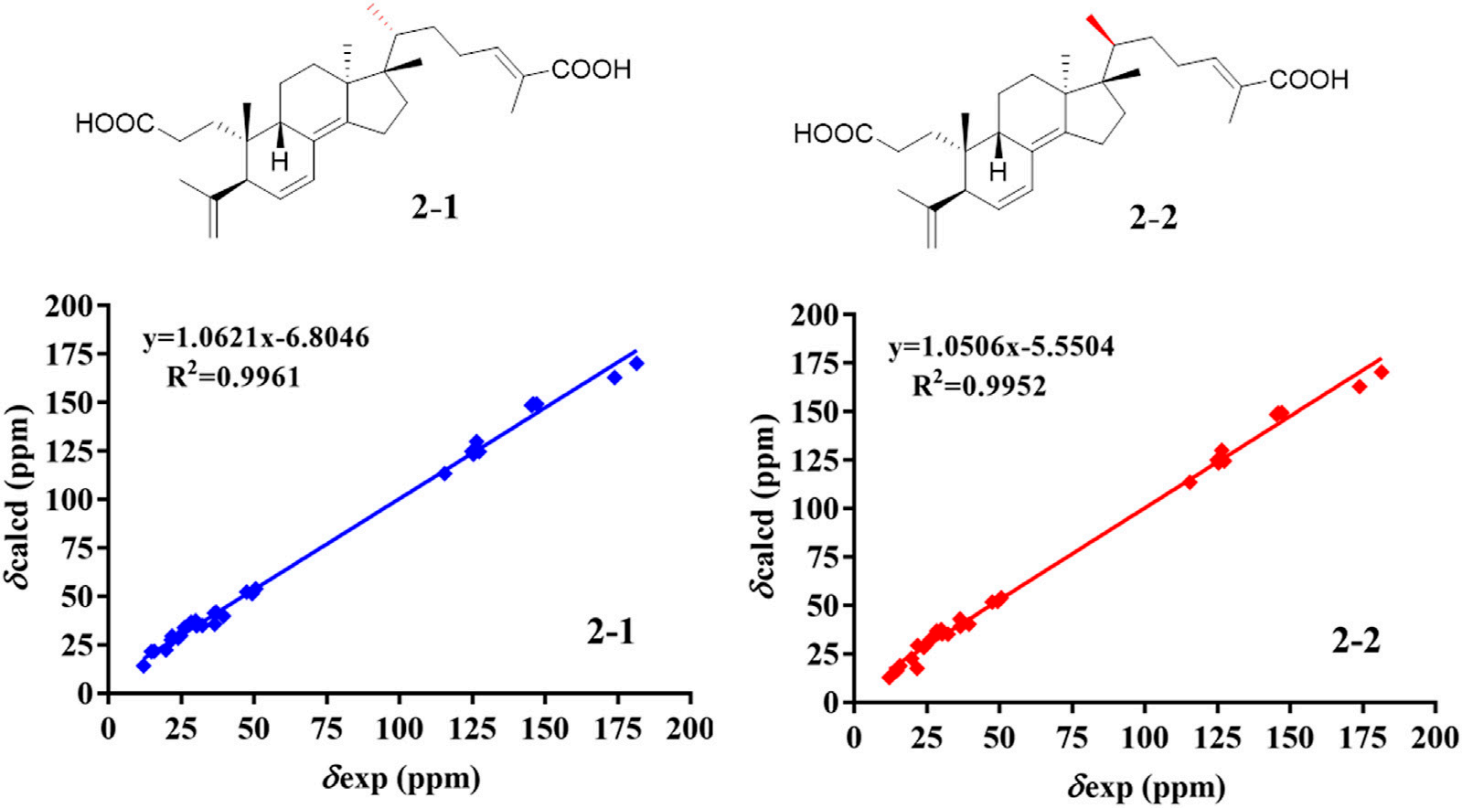
NMR calculations of compound **2**.

**FIGURE 7:**
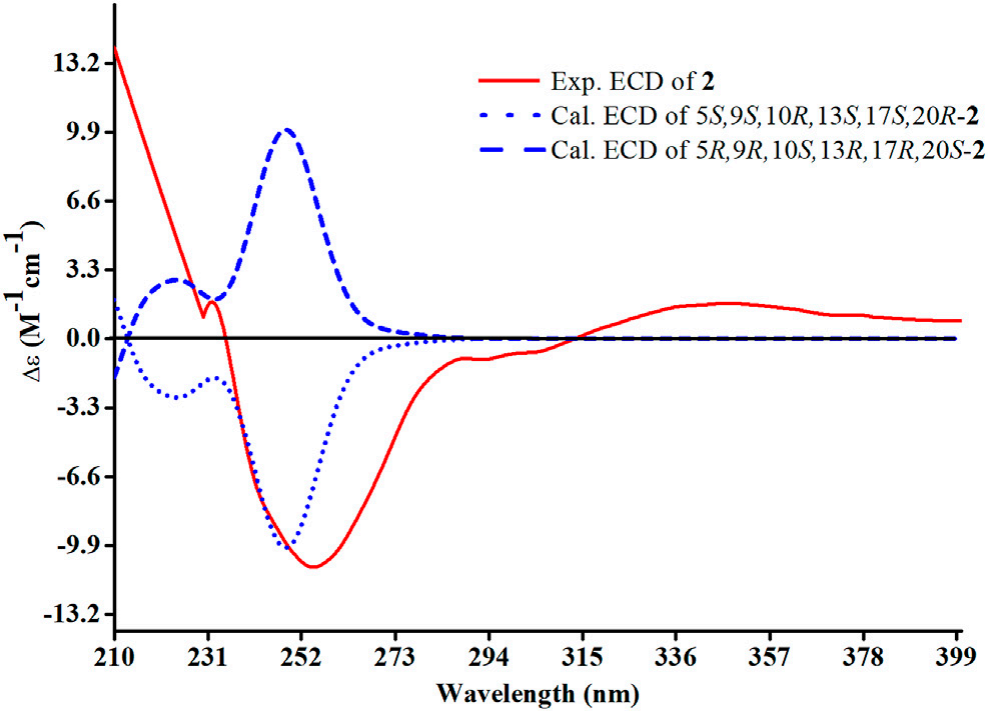
Experimental and calculated ECD spectra of compound **2**.

The relative configuration of **2** was determined following the NOE effects, which showed correlations (Figure 3) of H_3_-19 with H-9 and H-12b, H_3_-18 with H-15b and H-20, and H_3_-30 with H-11a and H_3_-21, suggesting that H-9 and H_3_-18 were *β*-oriented, and H_3_-21 and H_3_-30 were *a*-oriented. Moreover, the no NOE correlations of H_3_-27 with H-24 were found, suggesting that the double bond between C-24 and C-25 has a *trans*-configuration. Additionally, the relative configuration of C-20 was investigated by the TDDFT to calculate the ^13^C NMR data for **2-1** and **2-2**. As shown in Figure 6, the ^13^C NMR chemical shifts of isomers were calculated at the mPW1PW91/6-31+G** level. The calculation result of **2–1** (*R* ^2^ = 0.9961) matched the experimental data better than **2-2** (*R* ^2^ = 0.9952), which indicated that H_3_-21 has an *a*-orientation. The absolute configuration of **2** was determined by ECD data. The experimental ECD spectrum of **2** exhibited a negative cotton effect around 250 nm, which was consistent with the calculated ECD data of the (5*S*,9*S*,10*R*,13*S*,17*S*,20*R*) model (Figure 7). Thus, heilaohuacid B (**2**) was elucidated as a 5*S*, 9*S*, 10*R*, 13*S*, 17*S*, and 20*R* absolute configuration.

**FIGURE 8:**
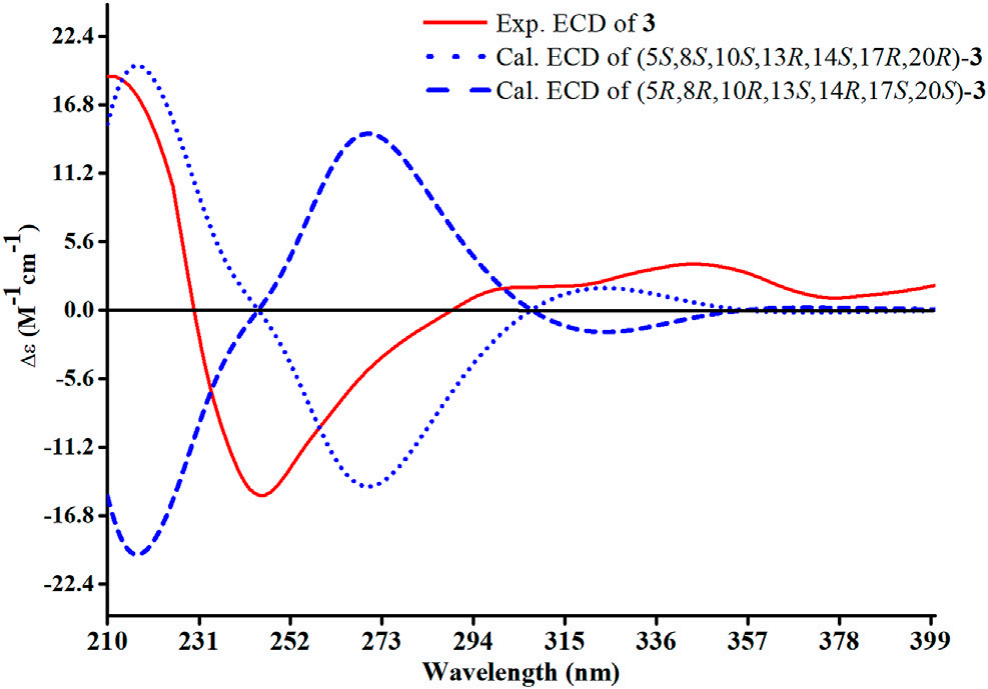
Experimental and calculated ECD spectra of compound **3**.

Compound **3** had a molecular formula of C_30_H_46_O_4_, requiring eight indices of hydrogen deficiency by analyzing the HR-ESI-MS at *m/z* 469.3352 [M-H]^−^ (calcd. for 469.3328, C_30_H_45_O_4_). Comprehensive analysis of the 1D and 2D NMR data revealed it to be the derivative of *seco*-coccinic acid K (Wang et al., 2012). The differences were that a methylene group at C-12 was replaced by a conjugated ketone group (*δ*_C_ 205.3, C-12) and the absence of a methoxy group at C-31 in **3**, which were confirmed by the HMBC correlations of H-11 with C-12, C-9, and C-13, H_3_-18 with C-12, and H_2_-1 and H_2_-2 with C-3. The relative configuration of H-8 was determined to be *β*-oriented, through the NOE correlations of H_3_-18 with H-8. The 5*, 8*, 10*S*, 13*R*, 14*S*, 17*R*, and 20*R* absolute configuration of **3** was determined by comparing the experimental and calculated ECD spectra (Figure 8). Accordingly, the structure of compound **3** was deduced as shown and given the trivial name heilaohuacid C.

Compound **4** was derived as a white amorphous powder with a molecular formula of C_30_H_46_O_3_. The molecular formula of compound **4** was determined by analyzing the HR-ESI-MS at *m/z* 453.3406 [M-H]^−^ (calcd. for 453.3374, C_30_H_45_O_4_). Comprehensive analyses of its NMR data (Tables 1, 3) suggested **4** to be a structural analogue of **3** as both shared the same 3,4-*seco*-lanostane triterpenoid skeleton. However, the obvious differences were that an α,β-conjugated ketone group (*δ*_C_ 200.2, C-6; *δ*_C_ 123.7, C-7; *δ*_C_ 175.3, C-8) shifted from ring C to ring B, a double bond at C-24/C-25 was present, but a ketone group was absent at C-23 in **4**, as supported using the HMBC spectral analyses. The relative configuration was confirmed by ROESY spectral analyses. Based on the NOE correlation of H_3_-19 with H-9, the H-9 was classified as *β*-oriented. Thus, the structure of 4 was assigned as shown in Figure 1, and named heilaohuacid D.

The molecular formula of compound **5** was C_30_H_48_O_3_, as determined by the HR-ESI-MS ion at *m/z* 455.3532 [M-H]^−^ (calcd. for 455.3525, C_30_H_47_O_3_), suggesting 7° of unsaturation. The ^1^H and ^13^C NMR spectra showed typical resonances for 3,4-*seco* ring A *δ*_H_ 4.75, 4.83 (each 1H, br s, H_2_-28), 1.68 (3H, s, H_3_-29) and *δ*_C_ 179.4 (C-3), 149.4 (C-4), 111.6 (C-28), and a pair of methylene doublets at *δ*_H_ 0.42 (*J* = 3.8 Hz) and 0.74 (*J* = 3.8 Hz), characteristics of the C-19 protons and carbon of the cyclopropane ring, suggesting that six was a 3,4-*seco*-cycloartane triterpenoid (Yang et al., 2015). Analysis of the 1D NMR data (Tables 2, 3) revealed that the structure of **5** was very similar to nigranoic acid (**25**) (Sun et al., 1996). The obvious differences were the presence of a ketone group at C-23, and the absence of double bond at C-24/C-25 and carboxyl groups at C-27 in **5**. The HMBC cross-peaks of H_2_-22 (*δ*_H_ 2.15, 2.43) and H_2_-24 (*δ*_H_ 2.26) with C-23 (*δ*_C_ 211.4) supported the ketone group locate at C-23. In a ROESY experiment, H-8 (*δ*_H_ 1.58) showed correlations with H_3_-18 (*δ*_H_ 1.01), which indicated that H-8 was *β*-oriented. Hence, compound **5** was elucidated as shown and named heilaohuacid E.

**TABLE 2:** ^1^H NMR data of compounds **5–9** in CDCl_3_ (600 MHz, *δ* in ppm, *J* in Hz).

Positions	5	6	7	8	9
1	a 1.38 m	a 1.17 m	a 1.58 m	a 1.91 m	a 1.71 m
	b 2.07 m	b 1.56 m	b 1.70 m	b 1.97 m	b 1.29 m
2	a 2.30 m	a 1.80 m	2.27 m	2.27 m	2.27 m
	b 2.53 m	b 2.09 m			
5	2.42 m	1.37 m	2.09 m	2.07 m	2.08 m
6	a 1.28 m	a 2.11 m	a 1.96 m	a 1.96 m	2.27 m
	b 1.53 m	b 2.25 m	b 1.99 m	b 1.99 m	
7	a 1.11 m	1.62 m	5.32 d (3.2)	5.32 d (3.2)	5.31 d (3.0)
	b 1.30 m				
8	1.58 m	2.23 m	—	—	—
9	—	—	2.57 m	2.57 m	2.57 m
11	a 1.27 m	5.31 d (6.0)	a 1.46 m	a 1.54 m	a 1.54 m
	b 2.09 m		b 1.55 m	b 1.62 m	b 1.65 m
12	1.30 m	a 1.93 m	a 1.66 m	a 1.51 m	a 1.83 m
		b 2.09 m	b 1.82 m	b 1.61 m	b 1.67 m
15	1.65 m	1.38 m	a 1.47 m	a 1.50 m	a 1.53 m
			b 1.54 m	b 1.83 m	b 1.47 m
16	a 1.29 m	a 1.34 m	1.90 m	1.92 m	a 1.94 m
	b 1.86 m	b 1.89 m			b 1.26 m
17	1.60 m	1.62 m	1.51 m	1.49 m	1.52 m
18	1.01 s	1.25 s	0.79 s	0.75 s	0.79 s
19	0.42 d (3.8)	0.67 s	0.84 s	0.84 s	0.84 s
	0.74 d (3.8)				
20	2.02 m	1.43 m	2.00 m	1.46 m	2. m
21	0.87 d (6.3)	0.94 d (6.4)	0.88 d (6.4)	0.88 d (6.4)	0.88 d (6.4)
22	a 2.15 m	a 2.04 m	a 2.47 m	1.47 m	2.51 m
	b 2.43 m	b 2.71 m	b 2.49 m		
23	—	a 2.11 m	—	a 1.58 m	—
		b 2.25 m		b 1.68 m	
24	2.26 d (6.9)	6.91 t (7.0)	a 2.56 m	3.34 m	6.05 s
			b 2.60 m		
25	2.14 m	—	—	—	—
26	0.91 d (12.1)	—	1.25 s	1.22 s	1.88 s
27	0.92 d (12.1)	1.84 s	1.25 s	1.17 s	2.14 s
28	4.75 brs 4.83 brs	1.07 s	4.82 brs 4.88 brs	4.82 brs 4.86 brs	4.87 brs 4.82 brs
29	1.68 s	1.08 s	1.80 s	1.79 s	1.79 s
30	0.93 s	0.75 s	1.03 s	1.03 s	1.03 s
31			3.67 s	3.66 s	3.67 s

**TABLE 3:** ^13^C NMR data of compounds **1–9** in CDCl_3_ (150 MHz, *δ* in ppm, *J* in Hz).

Positions	1	2	3	4	5	6	7	8	9
1	29.4 t	28.4 t	28.1 t	28.5 t	28.8 t	34.8 t	29.0 t	29.2 t	29.1 t
2	29.0 t	29.9 t	31.8 t	28.5 t	31.4 t	36.7 t	29.2 t	28.4 t	29.3 t
3	180.2 s	181.5 s	176.3 s	177.9 s	179.4 s	217.3 s	175.1 s	175.1 s	175.2 s
4	149.0 s	145.4 s	146.1 s	141.1 s	149.4 s	47.7 s	149.8 s	149.9 s	149.9 s
5	45.2 d	50.6 d	48.7 d	61.4 d	45.9 d	53.4 d	45.3 d	45.4 d	45.5 d
6	29.5 t	126.5 d	26.7 t	200.2 s	27.7 t	25.9 t	29.7 t	29.7 t	29.6 d
7	118.7 d	125.4 d	27.3 t	123.7 d	25.0 t	22.57 t	118.7 d	117.8 d	118.0 d
8	147.5 s	124.9 s	44.1 d	175.3 s	47.7 d	41.9 d	146.3 s	146.6 s	146.6 s
9	43.8 d	39.5 d	163.5 s	40.3 d	21.3 s	147.1 s	38.7 d	38.8 d	38.9 d
10	36.1 s	37.1 s	43.5 s	42.0 s	27.0 s	39.1 s	36.3 s	36.3 s	36.5 s
11	22.4 t	19.7 t	122.3 d	17.8 t	26.9 t	116.2 d	18.5 t	18.6 t	18.7 t
12	118.9 d	32.3 t	205.3 s	33.1 t	35.5 t	37.2 t	33.8 t	33.6 t	34.0 t
13	155.9 s	47.5 s	57.5 s	43.1 s	45.2 s	44.4 s	43.8 s	43.7 s	43.9 s
14	48.0 s	147.2 s	51.0 s	53.3 s	49.1 s	47.0 s	51.6 s	51.6 s	51.7 s
15	35.6 t	23.9 t	32.6 t	32.8 t	32.9 t	33.9 t	34.0 t	34.0 t	34.2 t
16	38.6 t	36.5 t	28.0 t	27.7 t	28.3 t	28.0 t	28.4 t	28.5 t	28.6 t
17	48.4 s	49.4 s	44.0 d	52.5 d	52.3 d	50.9 d	53.0 d	53.2 d	53.4 d
18	26.0 q	21.6 q	13.1 q	21.7 q	18.1 q	21.8 q	21.7 q	21.7 q	21.9 q
19	24.0 q	21.8 q	26.7 q	22.8 q	29.9 t	14.4 q	24.1 q	24.1 q	24.2 q
20	40.5 d	36.4 d	33.0 d	35.7 d	32.8 d	36.0 d	32.9 d	34.1 d	33.6 d
21	14.9 q	14.7 q	20.6 q	18.3 q	19.4 q	18.2 q	19.4 q	18.3 q	19.6 q
22	30.7 t	30.3 t	50.5 t	35.9 t	50.8 t	34.9 t	51.7 t	36.0 t	51.7 t
23	27.5 t	26.1 t	211.3 s	25.0 t	211.4 s	25.9 t	213.7 s	29.0 t	201.8 s
24	145.9 d	145.9 d	53.0 t	124.9 d	52.6 t	145.7 d	53.6 t	78.8 d	124.5 d
25	126.8 s	127.4 s	24.6 d	131.2 s	24.6 d	126.6 s	69.7 s	73.2 s	154.8 s
26	173.1 s	173.9 s	22.7 q	17.7 q	22.6 q	172.6 s	29.3 q	26.6 q	27.8 q
27	12.2 q	12.1 q	22.6 q	25.7 q	22.7 q	12.0 q	29.4 q	23.3 q	20.8 q
28	112.5 t	115.5 t	114.9 t	114.5 t	111.6 t	22.1 q	111.9 t	111.9 t	112.0 t
29	25.3 q	24.8 q	23.4 q	26.3 q	19.8 q	25.6 q	26.0 q	26.0 q	26.1 q
30	28.4 q	15.7 q	17.9 q	25.1 q	19.3 q	18.4 q	27.5 q	27.5 q	27.6 q
31							51.6 q	51.6 q	51.7 q

Compound **6** was obtained as a white amorphous powder and its molecular formula was deduced to be C_30_H_46_O_3_ based on the HR-ESI-MS showing molecular ion at *m/z* 453.3364 [M-H]^−^ (calcd. for 453.3374, C_30_H_45_O_3_) with 8° of unsaturation. The ^1^H NMR data (Table 2) showed the characteristic signals attributable to one methyl doublet at *δ*_H_ 0.94 (d, *J* = 6.4 Hz, H_3_-21), six methyl singlet protons at *δ*_H_ 1.25 (H_3_-18), 0.67 (H_3_-19), 1.84 (H_3_-27), 1.07 (H_3_-28), 1.08 (H_3_-29), and 0.75 (H_3_-30), and two olefinic protons at *δ*_H_ 5.31 (d, *J* = 6.0 Hz, H-11) and 6.91 (t, *J* = 7.0 Hz, H-24). Analyses of the ^13^C NMR and DEPT data (Table 3) showed that compound **6** contained seven methyls, nine methylenes, six methines (two olefinics), and eight quaternary carbons (two carboxyl group). These evidences indicated that compound **6** was an intact lanostane-type triterpenoid, whose ^1^H and ^13^C NMR spectroscopic data were very similar to those of coccinic acid (Li and Xue, 1986). The only difference was in the geometry of the double bond between C-24 and C-25. Because the carbon chemical shift of C-27 was shifted upfield by 8.0 ppm, compared with coccinic acid, this showed the presence of a double bond between C-24 and C-25 in 6, which was different with that of coccinic acid. Additionally, the configuration of 6 was determined using the ROESY spectrum (Supplementary Figure S42), in which H-27 showed correlation with H-23 but no NOE correlation was observed between H-27 and H-24, demonstrating that the geometry of the double bond must be *E* configuration (Figure 3). Therefore, the structure of **6** was elucidated as shown and assigned name heilaohuacid F.

Heilaohumethylester A (**7**) was assigned a molecular formula of C_31_H_50_O_4_ based on the HR-ESI-MS spectra and NMR data analysis (Tables 2, 3), suggesting that **7** was a methylated analogue of *seco*-coccinic acid C (**16**) (Wang et al., 2008). The presence of a methoxy group (*δ*_C_ 51.6, *δ*_H_ 3.67) was located at C-31, confirmed by the HMBC correlations of H_3_-31 with the carbonyl (*δ*_C_ 175.1) at C-3. The similar chemical shifts, coupling constants, and NOE correlations with **16** determined the relative configurations of **7**. Therefore, the structure of **7** was elucidated as shown.

Compound **8** was deduced to have the molecular formula of C_31_H_52_O_4_ from the molecular ion at *m/z* 511.3734 [M + Na]^+^ (calcd. for C_31_H_52_O_4_Na, 511.3758) in the HR-ESI-MS data. The NMR data of **8** were highly similar to those of **7**; HMBC spectral analysis showed that the obvious differences were absence of a ketone group at C-23 and a hydroxyl group was present at C-24 in **8**. Comparison of the NMR data of **8** with those of a pair of 24-epimers (Hong et al., 2013) with the OH at C-24 possessed different orientations, 24(*S*)-24,25-dihydroxytirucall-7-en-3-one (*δ*_C_ 78.6, *δ*_H_ 3.32) and (24*R*)-24,25-dihydroxytirucall-7-en-3-one (*δ*_C_ 79.5, *δ*_H_ 3.29). This indicated that the NMR data of 8 (*δ*_C_ 78.8, *δ*_H_ 3.34) were almost similar to the corresponding 24(*S*)-24,25-dihydroxytirucall-7-en-3-one, suggesting that the C-24 stereochemistry should be assigned as 24*S* in **8**. According to analysis of the NOE effect, and the ROESY cross-peaks of H_3_-19 with H-9, the H-9 was classified as *β*-oriented. Finally, the structure of **8** was identified, and named heilaohumethylester B accordingly.

Compound **9** was obtained as a white amorphous powder, and its molecular formula was found to be C_31_H_48_O_3_ deduced from HR-ESI-MS, indicating quasi-molecular ion peak at *m/z* 491.3499 [M + Na]^+^ (calcd. for 491.3501, C_31_H_48_O_3_Na). The ^1^H and ^13^C NMR data (Tables 2, 3) resembled that of **7**. However, a proton signal for OH-25 was absent with the presence of a double bond between C-24 and C-25. This was supported by the carbon chemical shifts of C-25 at *δ*_C_ 154.8, and verified by the HMBC correlations. The relative configurations of all the stereo-genic centers were assigned to be the same as **7**. Hence, the structure of **9** was deduced as shown and named heilaohumethylester C.

Twenty-two known analogues were identified as masticadienoic acid (**10**) (Jain et al., 1995), abiesatrine D (**11**) (Yang et al., 2010), 24(*E*)-3,4-*seco*-9*β*H-lanosta-4 (28),7,24-triene-3,26-dioic acid (**12**) (Benosman et al., 1994), (24*Z*)-3,4-*seco-*tirucalla-4(28),7,24-triene-3,26-dioic acid (**13**) (Kim et al., 2004), *seco*-coccinic acids A–C, F, and G (**14-18**) (Wang et al., 2008; Ban et al., 2009; Wang et al., 2012), kadsuracoccinic acids A and C (**19** and **20**) (Li et al., 2008), micranoic acid A (**21**) (Li et al., 2003), schiglausin H (**22**) (Zou et al., 2012), manwuweizic acid (**23**) (Liu et al., 1988), 3-monomethyl ester leucophyllic acid (**24**) (Abdelilah et al., 1994), nigranoic acid (**25**) (Sun et al., 1996), abiesatrine J (**26**) (Yang et al., 2010), changnanic acid (**27**) (Wang et al., 2006), schiglausins T (**28**) (Yu et al., 2016), schisandronic acid (**29**) (Wang et al., 2006), kadsulactone (**30**) (Tan et al., 1991), and schisanlactone B (**31**) (Wang et al., 2006), by comparison of their reported NMR spectroscopic data with those of corresponding published compounds.

The inhibited proliferation activity in RA-FLS cells of the isolated compounds (**1–31**) were evaluated using the MTT method, and methotrexate was used as the positive control (IC_50_ 4.10 µM). The results (Table 4) indicated that compounds **17**, **18**, and **31** exhibited good inhibition activities against RA-FLS cells with IC_50_ values of 7.52, 8.85, and 7.97 µM, respectively. Furthermore, all isolated compounds were evaluated for their inflammatory activity on inflammatory cytokines (IL-6 and TNF-α) released by LPS-induced RAW 264.7 cells. The inflammatory activity of the isolated compounds was determined using ELISA kits, with methotrexate as positive control. The results (Table 4) showed that compounds **4** and **31** suppressed the TNF-*a* expression in cell supernatant with IC_50_ values of 21.41 and 16.00 μM, respectively. Compounds **4**, **29**, and **31** suppressed IL-6 generation with IC_50_ values of 8.15, 17.20, and 9.86 μM, respectively.

**TABLE 4:** Anti-inflammatory and anti-RA-FLS activities data of compounds **1–31**.

Compounds	Anti-inflammatory cytokines^a^ IC_50_ (μΜ)		Anti-RA-FLS activity^b^ IC_50_ (μΜ)
	TNF-α	IL-6	RA-FLS
**4**	21.41	8.15	NA^d^
**17**	NA^b^	NA^b^	7.52
**18**	NA^b^	NA^b^	8.85
**29**	NA^b^	17.20	NA^d^
**31**	16.00	9.86	7.97
Methotrexate ^c^	1.10	4.51	4.10

a Inhibitory effects on LPS-stimulated TNF-α, and IL-6, generations in LPS-induced RAW, 264.7 cells.

b Cytotoxicity against RA-FLS, cells.

c Positive drug.

d No activity.

## Materials and Methods

### General Experimental Procedures

Optical rotations of compounds were determined by a Rudolph Research Analytical Autopol Ⅲ automatic polarimeter. UV analysis of compounds was performed on a Shimadzu 2450 UV-vis spectrometer. An Applied Photophysics Chirascan plus CD spectrometer was used to determine ECD spectrum. An Agilent Technologies Cary 630 FTIR spectrometer was used to determine IR spectra of compounds. ^1^H, ^13^C, ^1^H-^1^H COSY, HSQC, and HMBC spectra of compounds were determined by a Bruker AV-600 spectrometer with a single NMR probe at 600 MHz for ^1^H and 150 MHz for ^13^C in CDCl_3_. HR-ESI-MS experiments were performed using Waters UHPLC-H-CLASS/XEVO G2-XS Q-tof and Agilent 6,530 Accurate-Mass Q-TOF LC/MS. Column chromatographic silica gel was purchased from Qingdao Marine Chemical Inc., P. R. China. Semi-preparative HPLC was performed using an Agilent 1,260 Infinity Ⅱ liquid chromatograph with Agilent C18 (34 mm × 25 cm) column. Extract fractions were analyzed using TLC, and spots were visualized by heating silica gel plates sprayed with 5% H_2_SO_4_ in Vanillin solution. Petroleum ether (PE), hexane, ethyl acetate (EtOAc), ethanol, *n*-butanol (*n*-BuOH), methanol (MeOH), and dichloromethane (CH_2_Cl_2_) were purchased from Shanghai Titan Scientific Co. Ltd. Acetonitrile and methanol (HPLC grade) were purchased from Merck KGaA, 64,271 Darmstadt, Germany.

### Plant Material

The dried roots of *K. coccinea* were collected from Huaihua, Hunan Province, People’s Republic of China, in July 2015. Plant material was identified by one of the co-authors (WW). A voucher specimen (2,015,071,501) was deposited at TCM and Ethnomedicine Innovation and Development International Laboratory, Innovative Materia Medica Research Institute, School of Pharmacy, Hunan University of Chinese Medicine, Changsha, Hunan, People’s Republic of China.

### Extraction and Isolation

The dried roots of *K. coccinea* (100 kg) were extracted twice with 80% ethanol for 2 h each time under reflux and filtered. All extract solvents were evaporated under vacuum to obtain crude EtOH extract (3 kg). Half of the whole ethanol extract (1.5 kg) was suspended in H_2_O and successively partitioned with PE, CH_2_Cl_2_, EtOAc, and *n*-BuOH to give a PE-soluble fraction (182 g), CH_2_Cl_2_-soluble fraction (545 g), EtOAc-soluble fraction (330 g), *n*-BuOH-soluble portion (173 g), and H_2_O layer. The CH_2_Cl_2_-soluble fraction (545 g) was subjected to silica gel column chromatography (CC) eluted with hexane-EtOAc (80:1-0:1) to afford twelve fractions (C1–C12). Fractions C2 (13.1 g), C4 (45.2 g), and C6 (35.6 g) were separated by silica gel CC with cyclohexane-EtOAc (80:1-0:1) to yield **17** (12.0 g) and **13** (600 mg), respectively. Fraction C3 (29.5 g) was chromatographed on a silica gel column eluted with cyclohexane-EtOAc (200:1-0:1) gradients further to give 13 fractions (C3-1–13) and **12** (300 mg). Subfraction C3-5 (6 g) was separated on silica gel CC with hexane-EtOAc (100:1-20:1) to give **18** (2.0 g). Subfraction C3-7 (50 mg) was purified using semipreparative HPLC (99% MeOH in H_2_O) to yield compound **19** (t_R_ 28.3 min, 5 mg). Fraction C5 (49.5 g) was chromatographed on a silica gel column eluted with cyclohexane-CH_2_Cl_2_-EtOAc (80:1:0-200:1-0:5:1) gradients to give 12 further fractions (C5-1∼12). Subfraction C5-8 (5.0 g) was purified by using repeated silica gel CC eluted with hexane-EtOAc (40:1-0:1) and then by semipreparative HPLC with the mobile phase (93% MeOH/H_2_O) to obtain **30** (1 mg, t_R_ 18.3 min) and **8** (5.2 mg, t_R_ 26.8 min). Subfraction C5-9 (5.0 g) was further purified by using silica gel CC with hexane- EtOAc (100:1-0:1) to obtain 14 further fractions (C5-9-1–14), **10** (15 mg) was isolated from subfraction C5-9-12 by using silica gel CC eluted with hexane-CHCl_3_ (20:1-0:1), and subfraction C5-9-11 (128 mg) was purified by using semipreparative HPLC with the mobile phase (72% MeOH/H_2_O) to yield **14** (6 mg, t_R_ 20.2 min) and **29** (15 mg, t_R_ 23.8 min). Subfraction C5-10 (5.3 g) was chromatographed on a silica gel CC and eluted with hexane-EtOAc (40:1-0:1) gradients to give **15** (1.0 g). C5-9-12 (229.2 mg) was purified by semipreparative HPLC with the mobile phase (91% MeOH/H_2_O) obtained **22** (2.0 mg, t_R_ 22.1 min), **11** (4.5 mg, t_R_ 23.1 min), **7** (8.6 mg, t_R_ 24.5 min), and **24** (4.1 mg, t_R_ 31.7 min). Fraction C8 (15.5 g) was chromatographed on a silica gel column while eluted with PE-acetone (40:1-0:1) gradients to obtain 12 fractions (C8-1–12), subfraction C8-8 (15.5 g) was subjected to silica gel CC eluted with PE-acetone (40:1-0:1) to afford twelve subfractions (C8-8-1–12), **20** (3 mg, t_R_ 25.6 min) was isolated from the subfraction C8-8-9 (52.5 mg) by semipreparative HPLC with the mobile phase (93% MeOH/H_2_O), and **28** (3 mg, t_R_ 30.5 min) was isolated from the subfraction C8-8-8 (30.0 mg) by semipreparative HPLC with the mobile phase (90% MeOH/H_2_O). Fraction C9 (53.9 g) was chromatographed on a silica gel column eluted with PE-EtOAc (10:1-0:1) gradients to give 16 fractions (C9-1–16), subfraction C9-8 (5.3 g) was further subjected to silica gel CC with hexane-acetone (40:1-0:1) as mobile phase to obtain 17 further fractions (C9-8-1–17), and **1** (5 mg, t_R_ 64.2 min) and **26** (5 mg, t_R_ 79.1 min) were isolated from the subfraction C9-8-16 (120 mg) by semipreparative HPLC with the mobile phase (73% MeOH/H_2_O, 0–30 min →88% MeOH/H_2_O, 31–81 min →100% MeOH, 82–120 min). Compound **2** (3 mg, t_R_ 24.3 min) was isolated by semipreparative HPLC with the mobile phase (90% MeOH/H_2_O) from subfraction C9-8-15 (50 mg), subfraction C9-8-13 (68.7 mg) was purified by semipreparative HPLC with the mobile phase (70% MeOH/H_2_O) to obtain **4** (5 mg, t_R_ 38.3 min), subfraction C9-8-14 (135 mg) was purified by semipreparative HPLC with the mobile phase (57% MeOH/H_2_O, 0–17 min→85% MeOH/H_2_O, 18–30 min→90% MeOH/H_2_O, 31–60 min) to obtain **25** (12 mg, t_R_ 57.5 min), and **27** (5 mg, t_R_ 55.7 min), subfraction C9-8-15 (181.7 mg) was separated on C18 column eluted with 30%–100% MeOH-H_2_O to yield **21** (4 mg). Subfraction C9-10 (8.0 g) was subjected to silica gel CC eluted with hexane-acetone (40:1-0:1) gradients to **16** (1.0 g). Subfraction C9-12 (3.6 g) was repeatedly purified by silica gel CC with CHCl_3_-EtOAc (40:1-0:1) to obtain 12 further fractions (C9-12-1–12), and subfraction C9-12-2 (89.8 mg) was purified by semipreparative HPLC with the mobile phase (76% MeOH) to yield **31** (8 mg, t_R_ 32.9 min). C9-12–5 (22.9 mg) was purified by semipreparative HPLC with the mobile phase (90% MeOH/H_2_O) to yield **23** (14.1 mg, t_R_ 23.0 min) and **3** (11.6 mg, t_R_ 25.8 min). C9-12–6 (50.0 mg) was purified by semipreparative HPLC with the mobile phase (92% MeOH/H_2_O) to yield **6** (7.6 mg, t_R_ 15.8 min) and **5** (1.3 mg, t_R_ 17.4 min).

The EtOAc-soluble fraction (330 g) was applied to silica gel column chromatography, eluted with PE-EtOAc (100:0-0:100), to give twelve fractions (Fr. E1–E12). Fr. E5 (90 g) was separated by silica gel column chromatography using Hexane-EtOAc (100%-0) to afford eight subfractions (Fr. E5-1–8). Fr. E5-3 was purified by semipreparative HPLC (80% MeOH, 20 min) to obtain compound **9** (18.2 mg).

## Spectroscopic Data

### Heilaohuacid A (1)

White amorphous powder [α]25 D - 11 (*c* 0.01 CHCl_3_); UV (CHCl_3_) λ_max_ (log ε) 219 (4.2) nm, 248 (1.8) nm; IR (KBr) *ν*_max_ 2,926, 2,853, 1,718, 1,696, 1,656, 1,561, 1,457, 1,372, 1,260, 962, and 900 cm^−1^; ^1^H- and ^13^C NMR data, see Tables 1, 3; HR-ESI-MS *m/z* 491.3143 [M + Na]^+^ (calcd. for C_30_H_44_O_4_Na, 491.3137).

### Heilaohuacid B (2)

White amorphous powder [α]25 D - 39 (*c* 0.02 MeOH); UV (MeOH) λ_max_ (log ε) 212 (6.7) nm, 252 (6.52) nm; IR (KBr) *ν*_max_ 3,525, 2,972, 2,866, 1,791, 1,696, 1,652, 1,569, 1,472, 1,394, 1,056, 1,032, and 746 cm^−1^; ^1^H- and ^13^C NMR data, see Tables 1, 3; HR-ESI-MS *m/z* 467.3105 [M-H]^−^ (calcd. for 467.3161, C_30_H_43_O_4_).

### Heilaohuacid C (3)

White amorphous powder [α]25 D - 67 (*c* 0.07 MeOH); UV (MeOH) λ_max_ (log ε) 204 (3.5) nm, 250 (2.8) nm; IR (KBr) *ν*_max_ 3,414, 1,699, 1,652, 1,457, 1,372, 1,260, 962, 900, and 668 cm^−1^; ^1^H- and ^13^C NMR data, see Tables 1, 3; HR-ESI-MS *m/z* 469.3352 [M-H]^−^ (calcd. for C_30_H_45_O_4_, 469.3328).

### Heilaohuacid D (4)

White amorphous powder [α]25 D - 38 (*c* 0.04 MeOH); UV (MeOH) λ_max_ (log ε) 206 (6.4) nm, 245 (6.3) nm; IR (KBr) *ν*_max_ 3,628, 2,950, 1,771, 1,558, 1,436, 1,374, 1,260, 1,032, 667, and 646 cm^−1^; ^1^H- and ^13^C NMR data, see Tables 1, 3; HR-ESI-MS *m/z* 453.3406 [M-H]^−^ (calcd. for 453.3374, C_30_H_45_O_4_).

### Heilaohuacid E (5)

White amorphous powder [α]25 D + 19 (*c* 0.02 MeOH); UV (MeOH) λ_max_ (log ε) 204 (6.3) nm; IR (KBr) *ν*_max_ 3,401, 3,223, 2,972, 2,856, 1,749, 1,699, 1,558, 1,460, 1,246, 1,056, and 643 cm^−1^; ^1^H- and ^13^C NMR data, see Tables 2, 3; HR-ESI-MS *m/z* 455.3532 [M-H]^−^ (calcd. for 455.3525, C_30_H_47_O_3_).

### Heilaohuacid F (6)

White amorphous powder [α]25 D - 83 (*c* 0.06 MeOH); UV (MeOH) λ_max_ (log ε) 204 (3.6) nm, 240 (2.8) nm; IR (KBr) *ν*_max_ 2,944, 2,899, 1,715, 1,652, 1,448, 1,402, 1,372, 1,280, 959, and 900 cm^−1^; ^1^H- and ^13^C NMR data, see Tables 2, 3; HR-ESI-MS *m/z* 453.3364 [M-H]^−^ (calcd. for C_30_H_45_O_3_, 453.3374).

### Heilaohumethylester A (7)

White amorphous powder [α]25 D - 72 (*c* 0.02 MeOH); UV (MeOH) λ_max_ (log ε) 205 (6.6) nm; IR (KBr) *ν*_max_ 3,369, 2,946, 2,833, 1,699, 1,635, 1,506, 1,456, 1,372, 1,035, and 667 cm^−1^; ^1^H- and ^13^C NMR data, see Tables 2, 3; HR-ESI-MS *m/z* 509.3561 [M + Na]^+^ (calcd. for 509.3601, C_31_H_50_O_4_Na).

### Heilaohumethylester B (8)

White amorphous powder [α]25 D - 88 (*c* 0.03 MeOH); UV (MeOH) λ_max_ (log ε) 205 (6.5) nm; IR (KBr) *ν*_max_ 3,518, 3,199, 2,963, 1,718, 1,653, 1,558, 1,260, 1,014, 804, and 650 cm^−1^; ^1^H- and ^13^C NMR data, see Tables 2, 3; HR-ESI-MS *m/z* 511.3734 [M + Na]^+^ (calcd. for C_31_H_52_O_4_Na, 511.3758).

### Heilaohumethylester C (9)

White amorphous powder [α]25 D - 55 (*c* 0.05 CHCl_3_); UV (CHCl_3_) λ_max_ (log ε) 240 (3.4) nm; IR (KBr) *ν*_max_ 2,929, 2,856, 1,757, 1,704, 1,558, 1,460, 1,246, and 1,024 cm^−1^; ^1^H- and ^13^C NMR data, see Tables 2, 3; HR ESI MS *m/z* 491.3499 [M + Na]^+^ (calcd. for C_31_H_48_O_3_Na, 491.3501).

### ECD Calculations

Methods of quantum chemical ECD calculations for compounds **1–3** are described in the Supporting Information (Supplementary Figure S1).

### NMR Calculations

Methods of ^13^C NMR calculations for compounds **1** and **2** are described in the Supporting Information (Supplementary Figure S1).

### Cell Culture

Human RA-FLS cell line was purchased from Fenghui Biological Technology Co., Ltd. (Changsha, China). RAW264.7 cell line was purchased from Fuheng Biological Technology Co., Ltd. (Shanghai, China). Human RA-FLS and RAW264.7 cells were cultured in DMEM/F12 with 10% FBS and DMEM with 10% FBS in 5% CO_2_ at 37°C, respectively.

### Anti-Inflammatory Bioassay

Inhibition effects of all compounds (**1–31**) on release of inflammatory cytokines (IL-6 and TNF-α) in the supernatants on LPS-induced RAW264.7 cells were determined using ELISA kits (BOSTER Biological Technology Co. Ltd., Wuhan, China) following the manufacturer’s instructions. Methotrexate was used as a positive control.

### Inhibited Proliferation Activity Against RA-FLS Bioassay

Inhibited proliferation activity against RA-FLS cells was determined by the standard MTT assay methods as described previously. RA-FLS cells were seeded into 96-well plates and treated with different concentrations of all compounds for 48 h. Ten microliters of MTT (5 mg/ml) was then added to each well and incubated for 4 h. The supernatants were retrieved, and 100 μl of DMSO was added to each well and mixed by shaking for 5 min. Optical density values at 490 nm were measured using a microplate reader.

## Conclusion

The roots of *K. coccinea*, as a Tujia ethnomedicine, have been used to the treat rheumatoid arthritis for a long time in China. The present study has reported that nine new triterpenoids (**1–9**), along with 22 known analogues (**10–31**), were isolated from the roots of *K. coccinea*. Heilaohuacids A and B (**1** and **2**) contain a 3,4-*seco* ring A and unprecedented migration of Me-18 from C-13 to C-17 or C-14 to C-18; their relative and absolute configurations were determined by ^13^C NMR calculations and ECD data analysis. To the best of our knowledge, this type of lanostane triterpenoid derivative was rarely reported so far, which enriched the structural types of lanostane triterpenoids in *K. coccinea*. Additionally, compounds **4**, **17**, **18**, **29**, and **31** showed good anti-RA and/or anti-inflammatory activities. These findings suggest that lanostane triterpenoids from *K. coccinea* might serve as therapeutic agents for RA treatment.

## Data Availability

The original contributions presented in the study are included in the article/Supplementary Material. Further inquiries can be directed to the corresponding authors.
