# Supplementary material for: Triterpenoids From Kadsura coccinea With Their Anti-inflammatory and Inhibited Proliferation of Rheumatoid Arthritis-Fibroblastoid Synovial Cells Activities
Source: Front Chem. 2021 Dec 9;9:808870. doi: 10.3389/fchem.2021.808870 (PMC8695553; doi:10.3389/fchem.2021.808870)
Supplement: Supplementary file 1 [file Table1.DOC]

**Triterpenoids from *Kadsura coccinea* with Their Anti-inflammatory and Inhibited Proliferation of Rheumatoid Arthritis-Fibroblastoid synovial cells Activities**

Yu-pei Yang^a,1^, Yu-qing Jian^a,1^, Yong-bei Liu^a^, Muhammad Ismail^a,d^, Qing-ling Xie^a^, Huang-he Yu^a^, Bin Wang^a^, Bin Li^a^, Cai-yun Peng^a,*^, Bin Liu^b^, Rong-yong Man^c^, Wei Wang^a,*^

^a^*TCM and Ethnomedicine Innovation & Development International Laboratory, Innovative Materia Medica Research Institute, School of Pharmacy, Hunan University of Chinese Medicine, Changsha, Hunan 410208, People’s Republic of China*

^b^*Hunan Province Key Laboratory of Plant Functional Genomics and Developmental Regulation, College of Biology, Hunan University, Changsha, Hunan 410125, People's Republic of China*

^c^*Clinic Experimental Research Center, the first people's hospital of Huaihua, Huaihua, Hunan, 418000, People's Republic of China*

^d^*Department of Chemistry, Karakoram International University, Gilgit-15100, Pakistan*

*[Corresponding](javascript:void(0);) authors. Tel.: +86-136-5743-8606,

E-mail addresses: [wangwei402@hotmail.com](mailto:wangwei402@hotmail.com) (Wei Wang), paudy@126.com (Caiyun Peng).

^1^These authors contributed equally to the work.

Supporting Information

**1.** Experimental Section

**1.1** ECD calculations

**1.2** NMR calculations

**Figure S1.** Structure of triterpenoid compounds **1**-**31**

**Figure S2.** ^1^H NMR (600 MHz, CDCl_3_) spectrum of Heilaohuacid A (**1**)
**Figure S3.** ^13^C NMR (150 MHz, CDCl_3_) spectrum of Heilaohuacid A (**1**)
**Figure S4.** COSY NMR spectrum (CDCl_3_) of Heilaohuacid A (**1**)

**Figure S5.** HSQC NMR spectrum (CDCl_3_) of Heilaohuacid A (**1**)
**Figure S6.** HMBC NMR spectrum (CDCl_3_) of Heilaohuacid A (**1**)
**Figure S7.** ROESY spectrum (CDCl_3_) of Heilaohuacid A (**1**)
**Figure S8.** HRESIMS spectrum of Heilaohuacid A (**1**)

**Figure S9.** ^1^H NMR (600 MHz, CDCl_3_) spectrum of Heilaohuacid B (**2**)
**Figure S10.** ^13^C NMR (150 MHz, CDCl_3_) spectrum of Heilaohuacid B (**2**)
**Figure S11.** COSY NMR spectrum (CDCl_3_) of Heilaohuacid B (**2**)

**Figure S12.** HSQC NMR spectrum (CDCl_3_) of Heilaohuacid B (**2**)
**Figure S12.** HMBC NMR spectrum (CDCl_3_) of Heilaohuacid B (**2**)
**Figure S14.** ROESY spectrum (CDCl_3_) of Heilaohuacid B (**2**)
**Figure S15.** HRESIMS spectrum of Heilaohuacid B (**2**)

**Figure S16.** ^1^H NMR (600 MHz, CDCl_3_) spectrum of Heilaohuacid C (**3**)
**Figure S17.** ^13^C NMR (150 MHz, CDCl_3_) spectrum of Heilaohuacid C (**3**)
**Figure S18.** COSY NMR spectrum (CDCl_3_) of Heilaohuacid C (**3**)

**Figure S19.** HSQC NMR spectrum (CDCl_3_) of Heilaohuacid C (**3**)
**Figure S20.** HMBC NMR spectrum (CDCl_3_) of Heilaohuacid C (**3**)
**Figure S21.** ROESY spectrum (CDCl_3_) of Heilaohuacid C (**3**)
**Figure S22.** HRESIMS spectrum of Heilaohuacid C (**3**)

**Figure S23.** ^1^H NMR (600 MHz, CDCl_3_) spectrum of Heilaohuacid D (**4**)
**Figure S24.** ^13^C NMR (150 MHz, CDCl_3_) spectrum of Heilaohuacid D (**4**)
**Figure S25.** COSY NMR spectrum (CDCl_3_) of Heilaohuacid D (**4**)

**Figure S26.** HSQC NMR spectrum (CDCl_3_) of Heilaohuacid D (**4**)
**Figure S27.** HMBC NMR spectrum (CDCl_3_) of Heilaohuacid D (**4**)
**Figure S28** ROESY spectrum (CDCl_3_) of Heilaohuacid D (**4**)
**Figure S29.** HRESIMS spectrum of Heilaohuacid D (**4**)

**Figure S30.** ^1^H NMR (600 MHz, CDCl_3_) spectrum of Heilaohuacid E (**5**)
**Figure S31.** ^13^C NMR (150 MHz, CDCl_3_) spectrum of Heilaohuacid E (**5**)
**Figure S32.** COSY NMR spectrum (CDCl_3_) of Heilaohuacid E (**5**)

**Figure S33.** HSQC NMR spectrum (CDCl_3_) of Heilaohuacid E (**5**)
**Figure S34.** HMBC NMR spectrum (CDCl_3_) of Heilaohuacid E (**5**)
**Figure S35** ROESY spectrum (CDCl_3_) of Heilaohuacid E (**5**)
**Figure S36.** HRESIMS spectrum of Heilaohuacid E (**5**)

**Figure S37.** ^1^H NMR (600 MHz, CDCl_3_) spectrum of Heilaohuacid F (**6**)
**Figure S38.** ^13^C NMR (150 MHz, CDCl_3_) spectrum of Heilaohuacid F (**6**)
**Figure S39.** COSY NMR spectrum (CDCl_3_) of Heilaohuacid F (**6**)

**Figure S40.** HSQC NMR spectrum (CDCl_3_) of Heilaohuacid F (**6**)
**Figure S41.** HMBC NMR spectrum (CDCl_3_) of Heilaohuacid F (**6**)
**Figure S42** ROESY spectrum (CDCl_3_) of Heilaohuacid F (**6**)
**Figure S43.** HRESIMS spectrum of Heilaohuacid F (**6**)

**Figure S44.** ^1^H NMR (600 MHz, CDCl_3_) spectrum of Heilaohumethylester A (**7**)
**Figure S45.** ^13^C NMR (150 MHz, CDCl_3_) spectrum of Heilaohumethylester A (**7**)
**Figure S46.** COSY NMR spectrum (CDCl_3_) of Heilaohumethylester A (**7**)

**Figure S47.** HSQC NMR spectrum (CDCl_3_) of Heilaohumethylester A (**7**)
**Figure S48.** HMBC NMR spectrum (CDCl_3_) of Heilaohumethylester A (**7**)
**Figure S49** ROESY spectrum (CDCl_3_) of Heilaohumethylester A (**7**)
**Figure S50.** HRESIMS spectrum of Heilaohumethylester A (**7**)

**Figure S51.** ^1^H NMR (600 MHz, CDCl_3_) spectrum of Heilaohumethylester B (**8**)
**Figure S52.** ^13^C NMR (150 MHz, CDCl_3_) spectrum of Heilaohumethylester B (**8**)
**Figure S53.** COSY NMR spectrum (CDCl_3_) of Heilaohumethylester B (**8**)

**Figure S54.** HSQC NMR spectrum (CDCl_3_) of Heilaohumethylester B (**8**)
**Figure S55.** HMBC NMR spectrum (CDCl_3_) of Heilaohumethylester B (**8**)
**Figure S56** ROESY spectrum (CDCl_3_) of Heilaohumethylester B (**8**)
**Figure S57.** HRESIMS spectrum of Heilaohumethylester B (**8**)

**Figure S58.** ^1^H NMR (600 MHz, CDCl_3_) spectrum of Heilaohumethylester C (**9**)
**Figure S59.** ^13^C NMR (150 MHz, CDCl_3_) spectrum of Heilaohumethylester C (**9**)
**Figure S60.** COSY NMR spectrum (CDCl_3_) of Heilaohumethylester C (**9**)

**Figure S61.** HSQC NMR spectrum (CDCl_3_) of Heilaohumethylester C (**9**)
**Figure S62.** HMBC NMR spectrum (CDCl_3_) of Heilaohumethylester C (**9**)
**Figure S63** ROESY spectrum (CDCl_3_) of Heilaohumethylester C (**9**)
**Figure S64.** HRESIMS spectrum of Heilaohumethylester C (**9**)

**TableS1-2** ECD calculations results of compound **1**

**TableS3-4** ECD calculations results of compound **2**

**TableS5-6** ECD calculations results of compound **3**

**TableS7-8** NMR calculations results of compound **1-1**

**TableS9-10** NMR calculations results of compound **1-2**

**TableS11-12** NMR calculations results of compound **2-1**

**TableS13-14** NMR calculations results of compound **2-1**

**1. Experimental Section**

**1.1 ECD calculations of compounds 1-3**

In general, conformational analyses were carried out via random searching in the Sybyl-X 2.0 using the MMFF94S force field with an energy cutoff of 2.5 kcal/mol.[1] The results showed four lowest energy conformer for both compounds. Subsequently, the conformers were re-optimized using DFT at the PBE0-D3(BJ)/def2-SVP level in MeOH using the polarizable conductor calculation model (SMD) by the ORCA4.2.1 program.[2][3] The energies, oscillator strengths, and rotational strengths (velocity) of the first 60 electronic excitations were calculated using the TDDFT methodology at the PBE0/def2-TZVP level in MeOH. The ECD spectra were simulated by the overlapping Gaussian function (half the bandwidth at 1/e peak height, sigma = 0.30 for all)[4]. To get the ﬁnal spectra, the simulated spectra of the conformers were averaged according to the Boltzmann distribution theory and their relative Gibbs free energy (∆G). By comparing the experiment spectra with the calculated model molecules, the absolute configuration of the only chiral center was determined to be.

[1].Sybyl Software, version X 2.0; Tripos Associates Inc.: St. Louis, MO, 2013.

[2]. Neese, F. (2012) The ORCA program system, Wiley Interdiscip. Rev.: Comput. Mol. Sci., 2, 73-78.

[3]. Neese, F. (2017) Software update: the ORCA program system, version 4.0, Wiley Interdiscip. Rev.: Comput. Mol. Sci., 8, e1327.

[4]. Stephens, P. J.; Harada, N. ECD cotton effect approximated by the Gaussian curve and other methods. Chirality2010, 22, 229-233.

**1.2 NMR calculations**

In general, conformational analyses were carried out via random searching in the Sybyl-X 2.0 using the MMFF94S force field with an energy cutoff of 5.0 kcal/mol [1]. The results showed nine lowest energy conformers. Subsequently, the conformers were re-optimized at the B3LYP-D3(BJ)/6-31G** level in PCM chloroform by the GAUSSIAN09 program.^[2]^All conformers used for property calculations in this work were characterized to be stable point on potential energy surface (PES) with no imaginary frequencies. NMR shielding constants were computed using the GIAO method at the mPW1PW91/6-31+G** level in PCM chloroform by the GAUSSIAN09 program [2]. Gibbs free energies for conformers were determined by using thermal correction at B3LYP-D3(BJ)/6-31G** level and electronic energies evaluated at the wB97M-V/def2-TZVP level in PCM chloroform using ORCA [3, 4] Boltzmann weights were computed using relative gibbs free energies. ^[5]^The unscaled chemical shifts (*δ*_u_) were computed using TMS (Tetramethylsilane) as a reference standard according to *δ*_u_ = σ_0_ - σ_x_, where σ_x_ is the Boltzmann averaged shielding tensor (over all significantly populated conformations) and σ_0_ is the shielding tensor of the TMS computed at the same level of theory employed for σ_x_. The scaled chemical shifts (*δ*_s_) were calculated as *δ*_s_ = (*δ*_u_ - b) / m, where m and b are the slope and intercept, respectively, deduced from a linear regression calculation on a plot of *δ*_u_ against *δ*_exp_. The DP4^+^ calculations were run by the Excel spreadsheet available for free at sarotti-nmr.weebly.com or as part of the Supporting Information of the original paper [5]. Finally we identified is the right isomer.

[1].Sybyl Software, version X 2.0; Tripos Associates Inc.: St. Louis, MO, 2013.
[2].Gaussian 09, Revision E.01, M. J. Frisch, G. W. Trucks, H. B. Schlegel, G. E. Scuseria, M. A. Robb, J. R. Cheeseman, G. Scalmani, V. Barone, B. Mennucci, G. A. Petersson, H. Nakatsuji, M. Caricato, X. Li, H. P. Hratchian, A. F. Izmaylov, J. Bloino, G. Zheng, J. L. Sonnenberg, M. Hada, M. Ehara, K. Toyota, R. Fukuda, J. Hasegawa, M. Ishida, T. Nakajima, Y. Honda, O. Kitao, H. Nakai, T. Vreven, J. A. Montgomery, Jr., J. E. Peralta, F. Ogliaro, M. Bearpark, J. J. Heyd, E. Brothers, K. N. Kudin, V. N. Staroverov, R. Kobayashi, J. Normand, K. Raghavachari, A. Rendell, J. C. Burant, S. S. Iyengar, J. Tomasi, M. Cossi, N. Rega, J. M. Millam, M. Klene, J. E. Knox, J. B. Cross, V. Bakken, C. Adamo, J. Jaramillo, R. Gomperts, R. E. Stratmann, O. Yazyev, A. J. Austin, R. Cammi, C. Pomelli, J. W. Ochterski, R. L. Martin, K. Morokuma, V. G. Zakrzewski, G. A. Voth, P. Salvador, J. J. Dannenberg, S. Dapprich, A. D. Daniels, ?. Farkas, J. B. Foresman, J. V. Ortiz, J. Cioslowski, and D. J. Fox, Gaussian, Inc., Wallingford CT, 2009.
[3]. Neese, F. (2012) The ORCA program system, Wiley Interdiscip. Rev.: Comput. Mol. Sci., 2, 73-78
[4]. Neese, F. (2017) Software update: the ORCA program system, version 4.0, Wiley Interdiscip. Rev.: Comput. Mol. Sci., 8, e1327.

**Figure S1.** Structure of triterpenoid compounds **1**-**31**


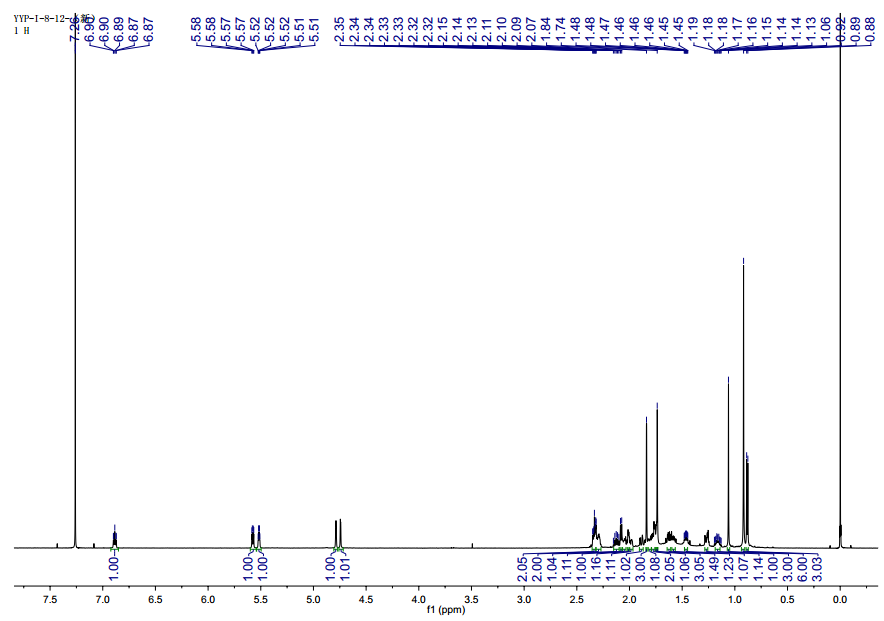
**Figure S2.** ^1^H NMR (600 MHz, CDCl_3_) spectrum of Heilaohuacid A (**1**)

**
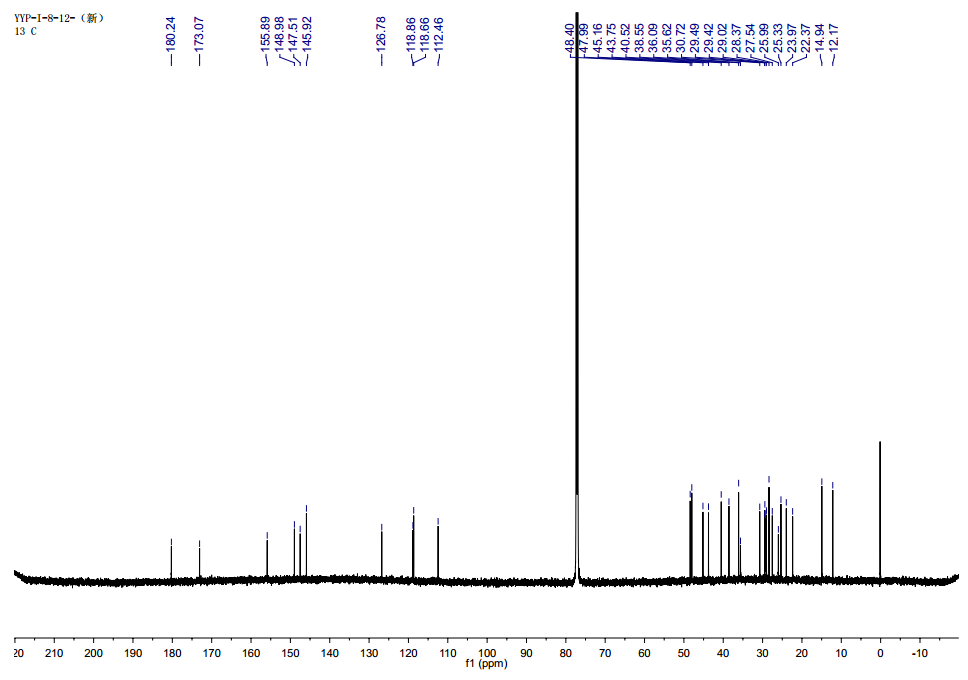
**

**Figure S3.** ^13^C NMR (150 MHz, CDCl_3_) spectrum of Heilaohuacid A (**1**)


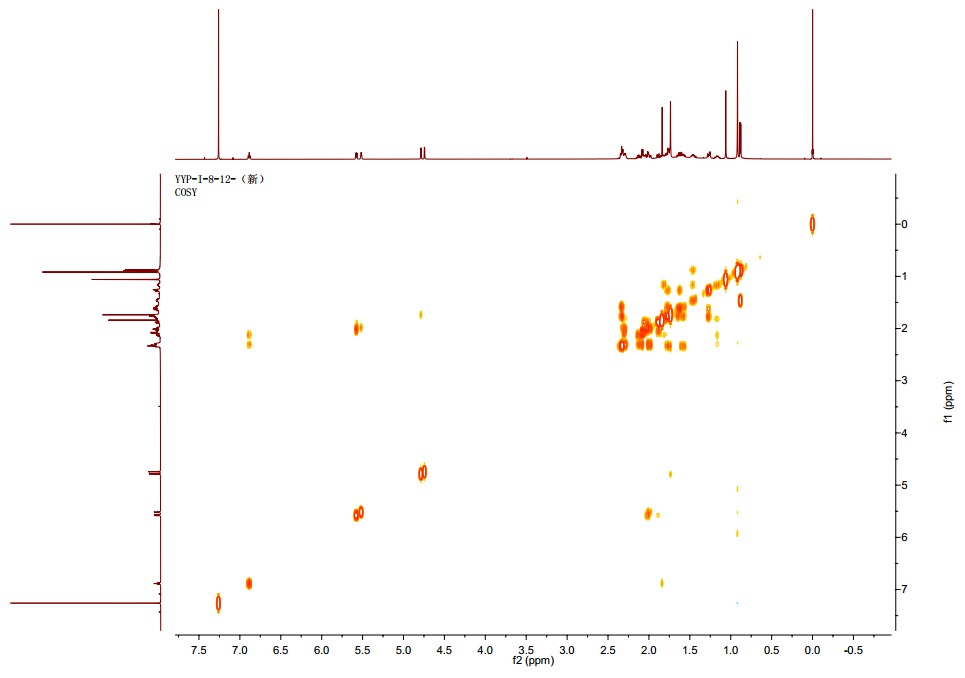


**Figure S4.** COSY NMR spectrum (CDCl_3_) of Heilaohuacid A (**1**)


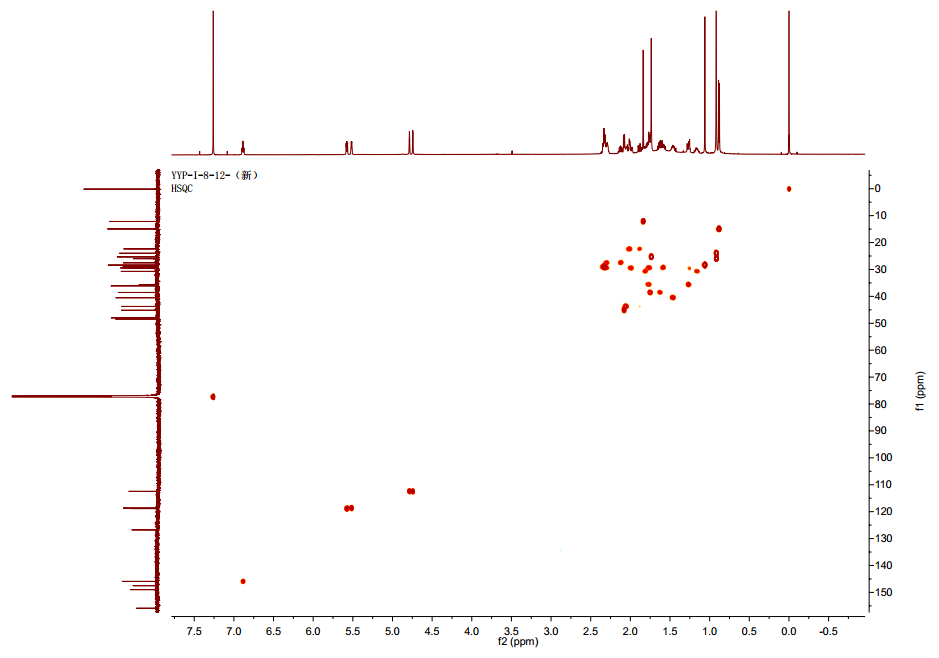
**Figure S5.** HSQC NMR spectrum (CDCl_3_) of Heilaohuacid A (**1**)


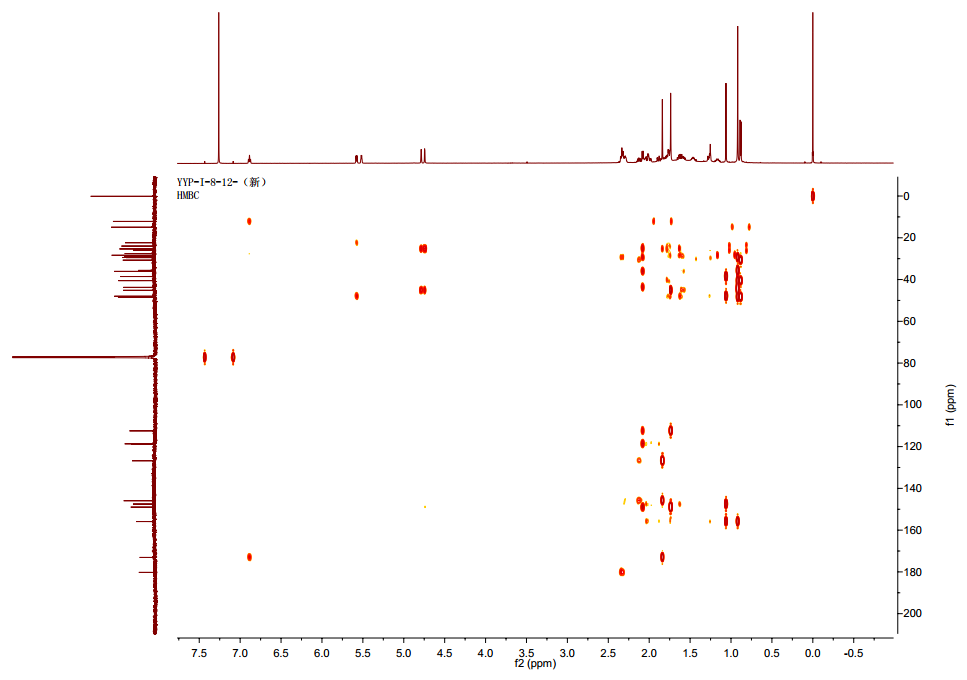


**Figure S6.** HMBC NMR spectrum (CDCl_3_) of Heilaohuacid A (**1**)


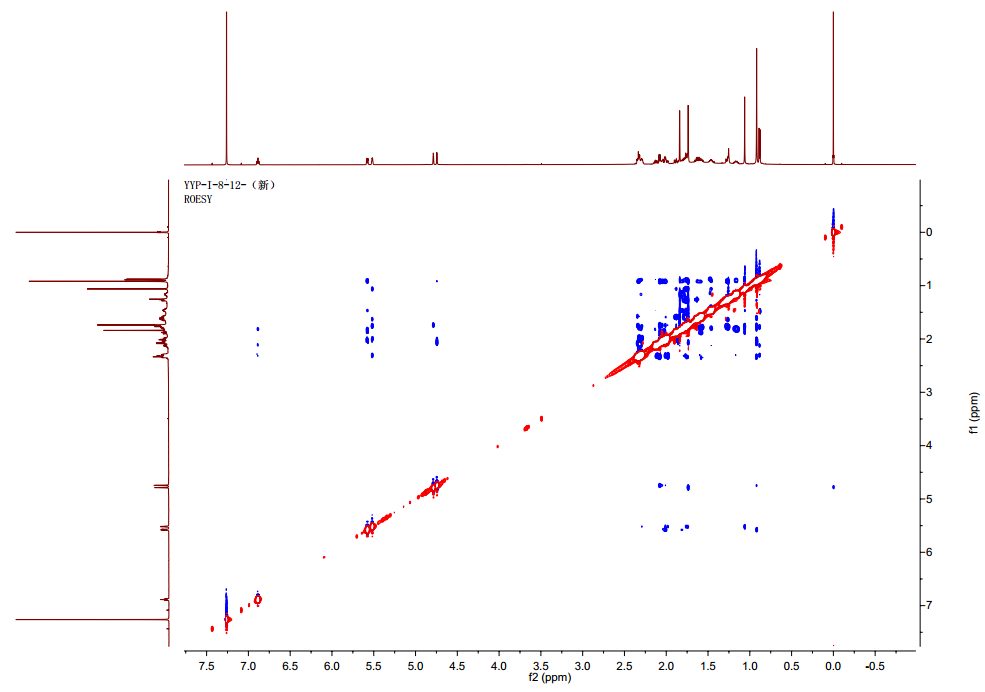


**Figure S7.** ROESY spectrum (CDCl_3_) of Heilaohuacid A (**1**)


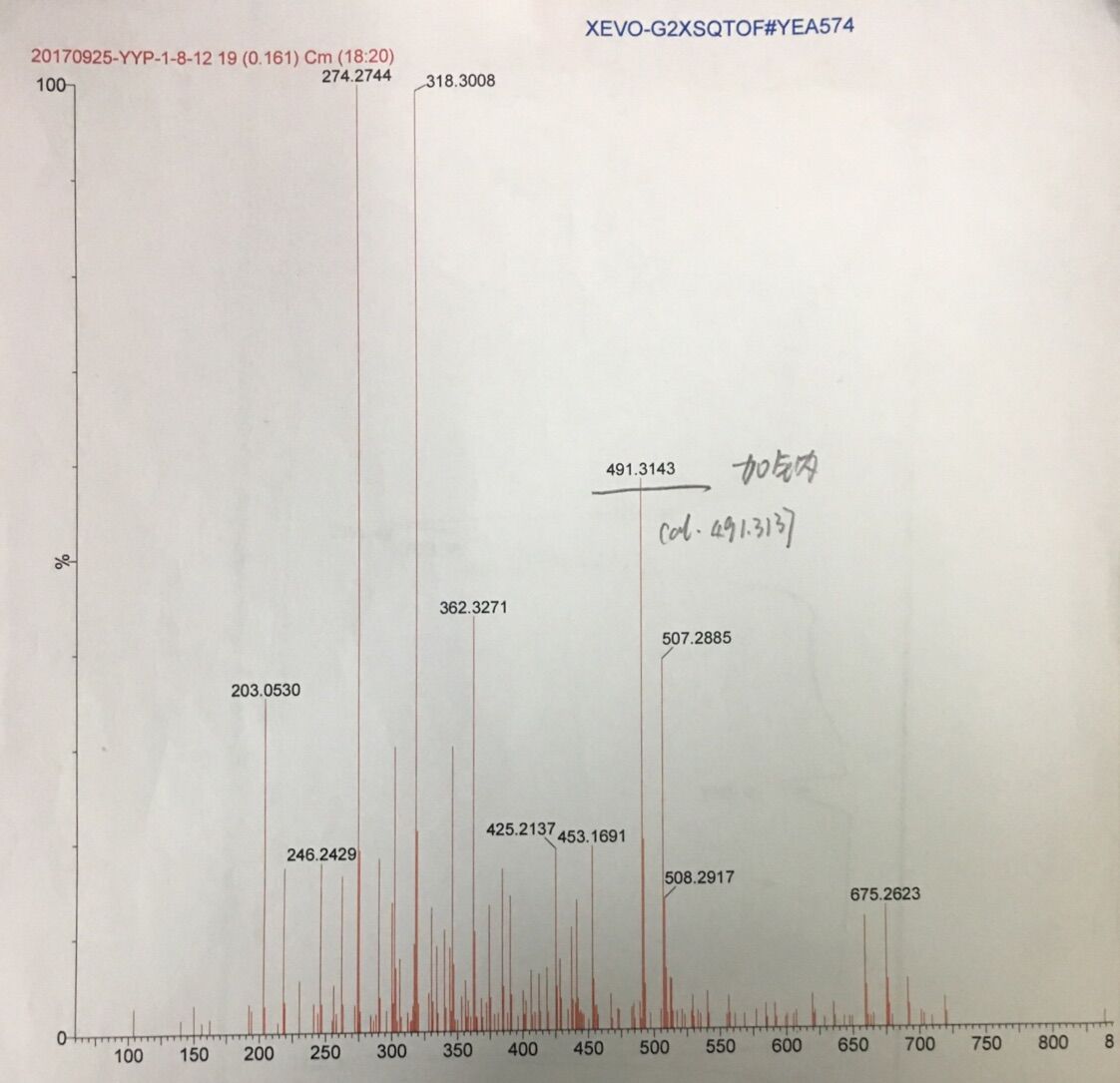
**Figure S8.** HRESIMS spectrum of Heilaohuacid A (**1**)


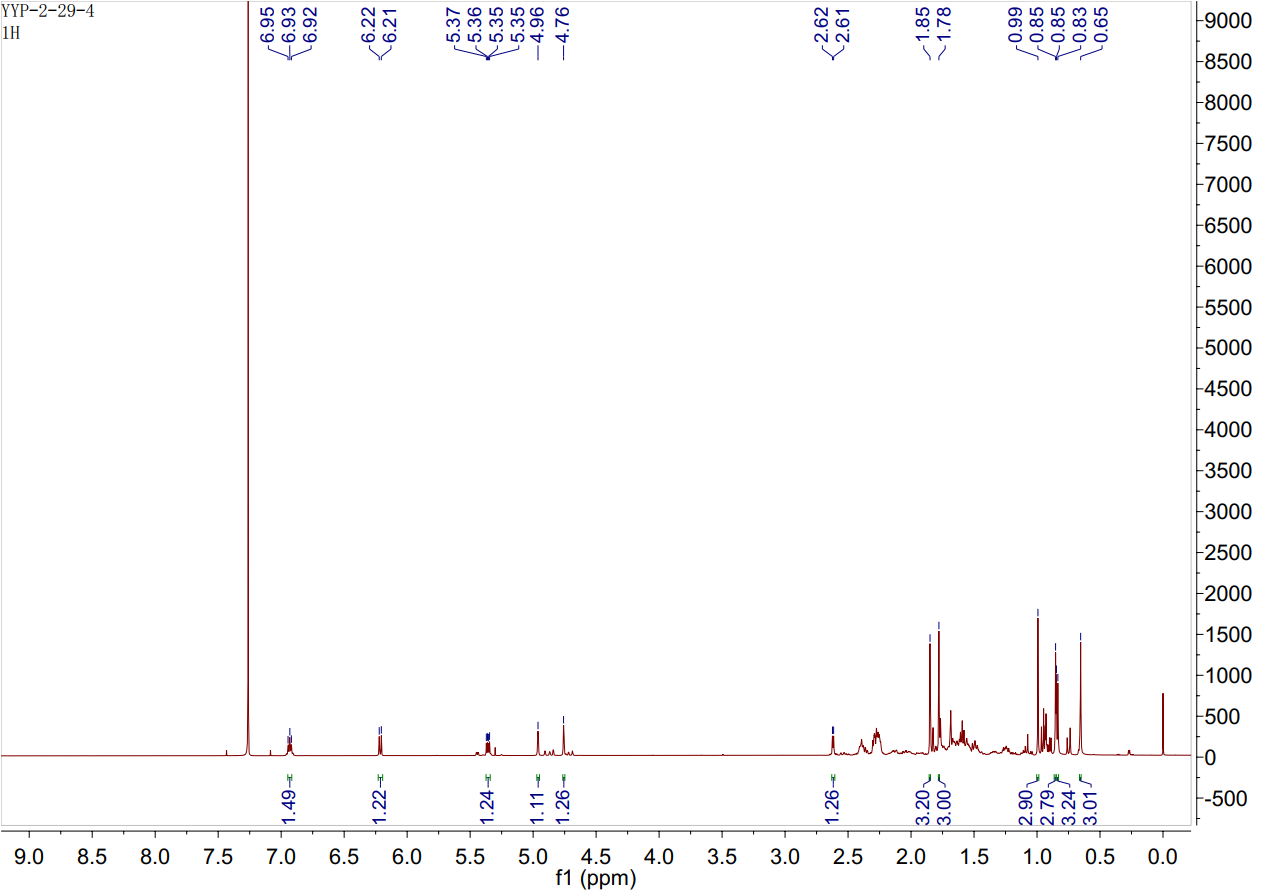
**Figure S9.** ^1^H NMR (600 MHz, CDCl_3_) spectrum of Heilaohuacid B (**2**)


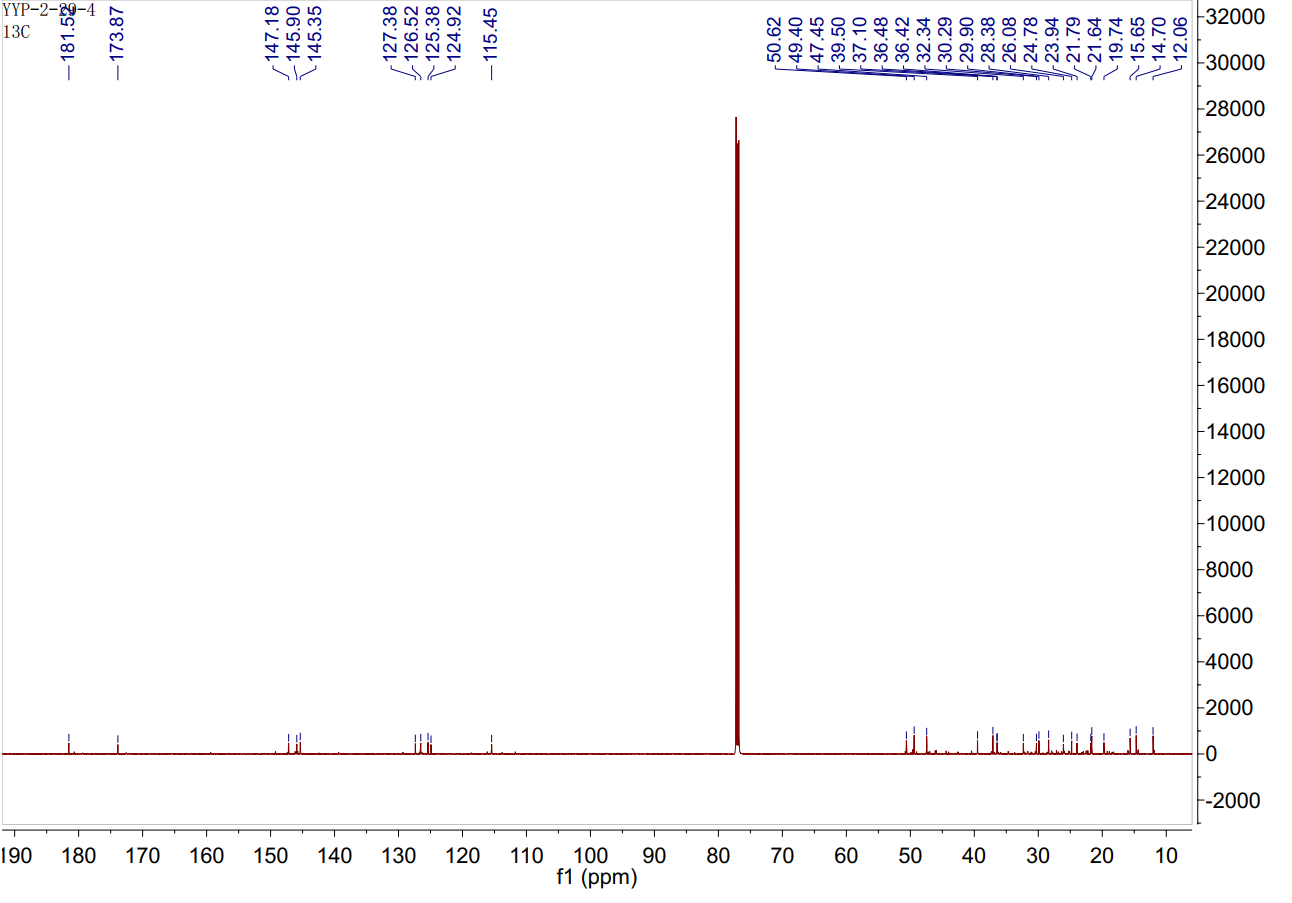


**Figure S10.** ^13^C NMR (150 MHz, CDCl_3_) spectrum of Heilaohuacid B (**2**)


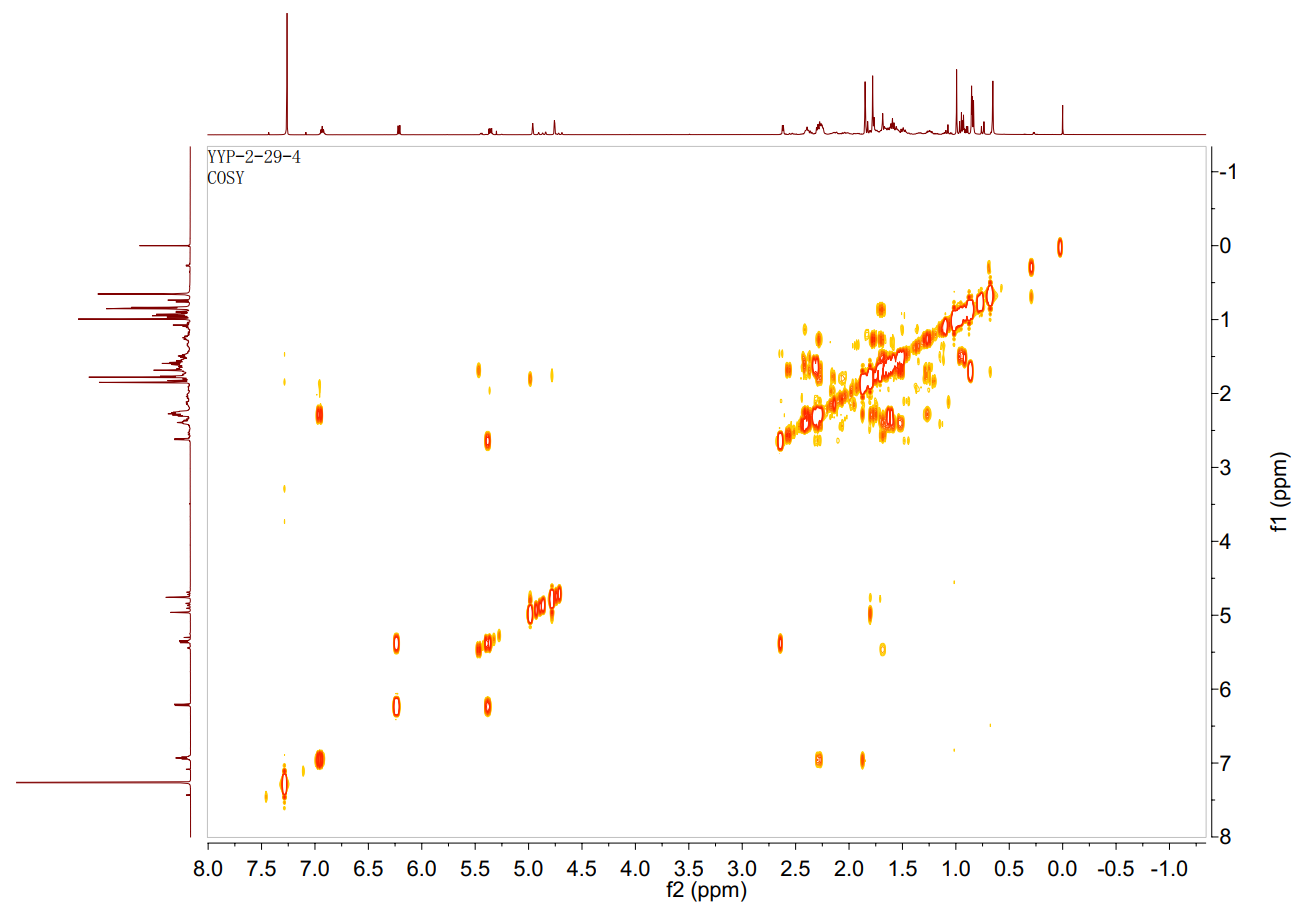


**Figure S11.** COSY NMR spectrum (CDCl_3_) of Heilaohuacid B (**2**)


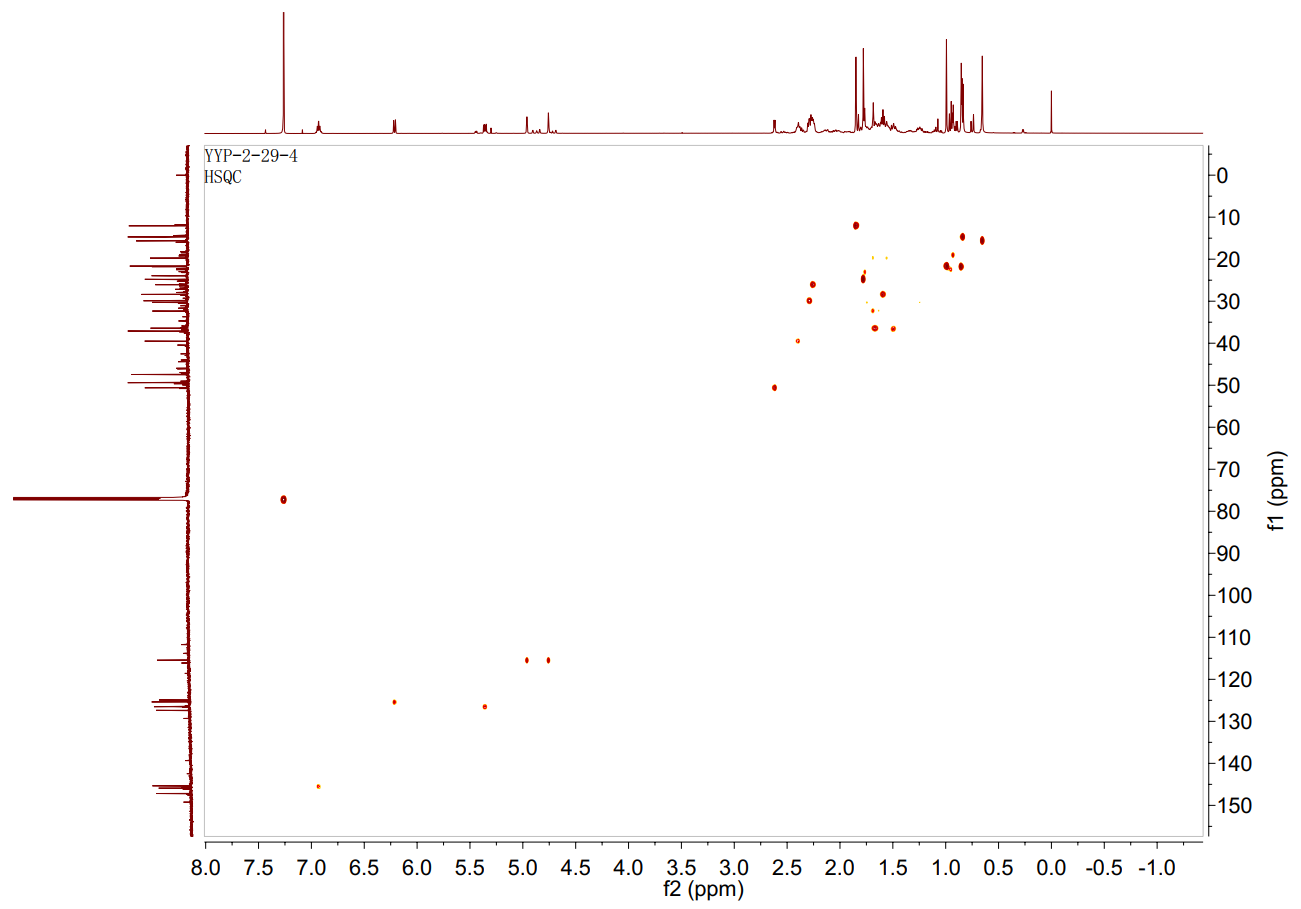


**Figure S12.** HSQC NMR spectrum (CDCl_3_) of Heilaohuacid B (**2**)


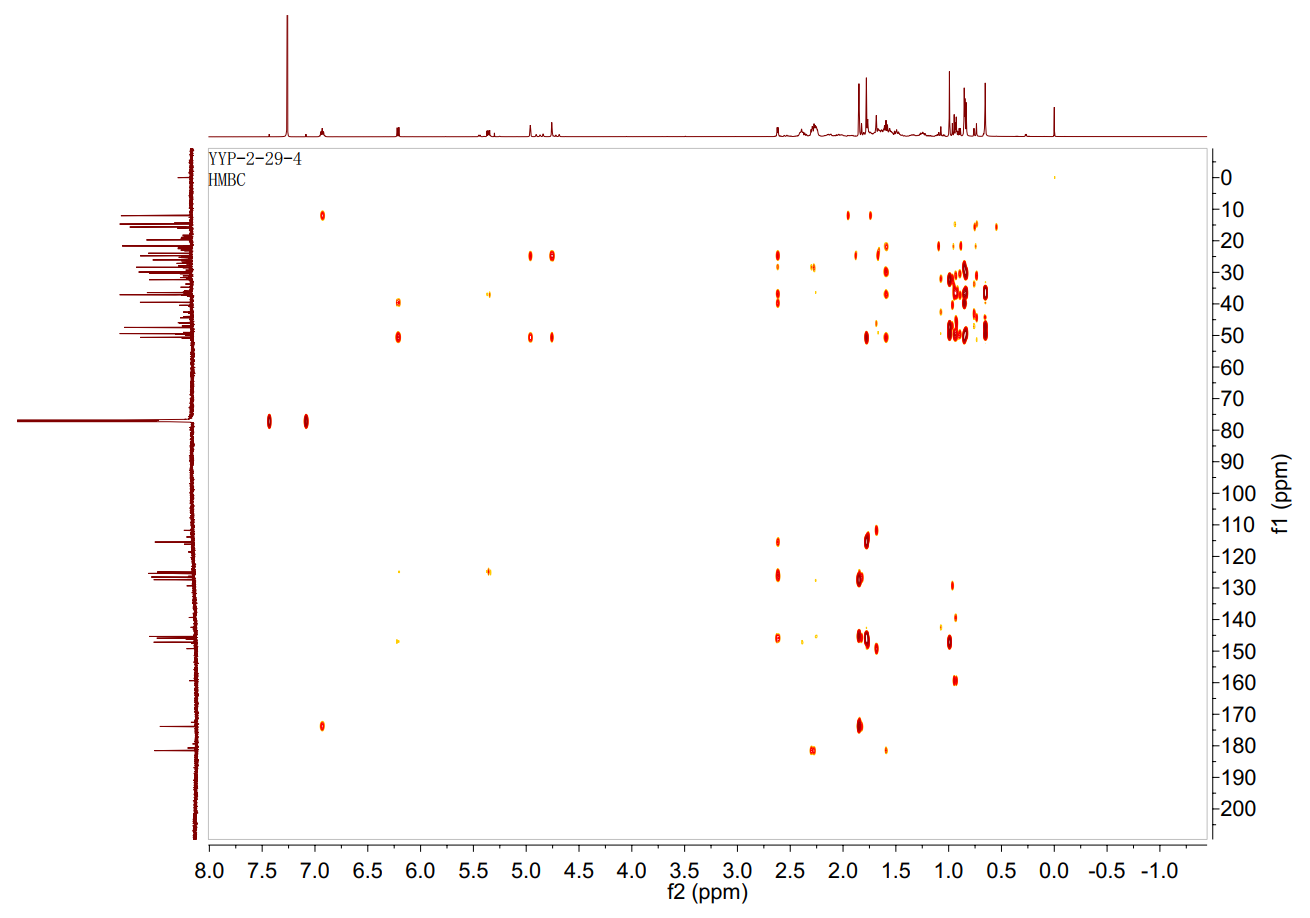


**Figure S13.** HMBC NMR spectrum (CDCl_3_) of Heilaohuacid B (**2**)


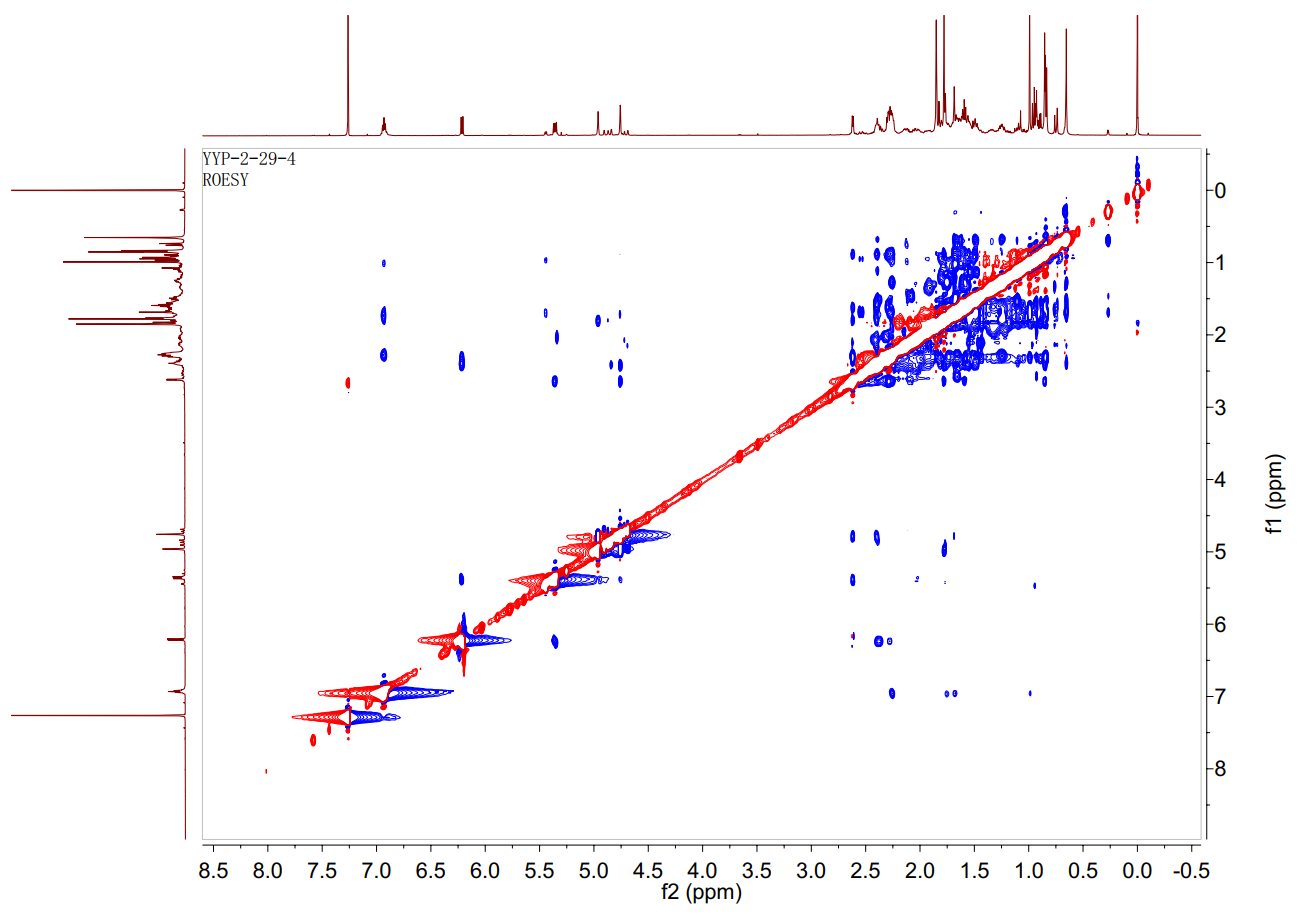


**Figure S14.** ROESY spectrum (CDCl_3_) of Heilaohuacid B (**2**)


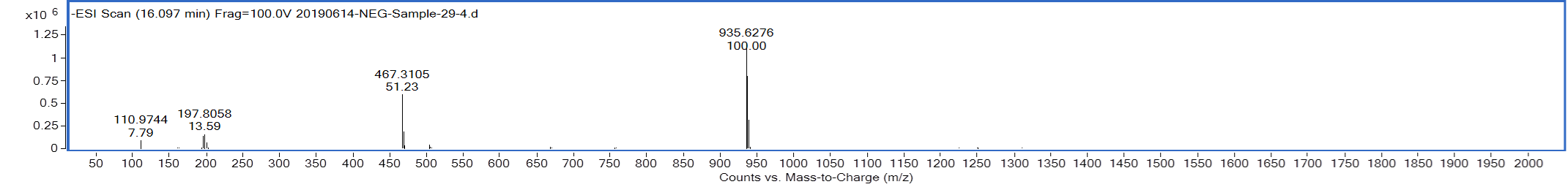


**Figure S15.** HRESIMS spectrum of Heilaohuacid B (**2**)


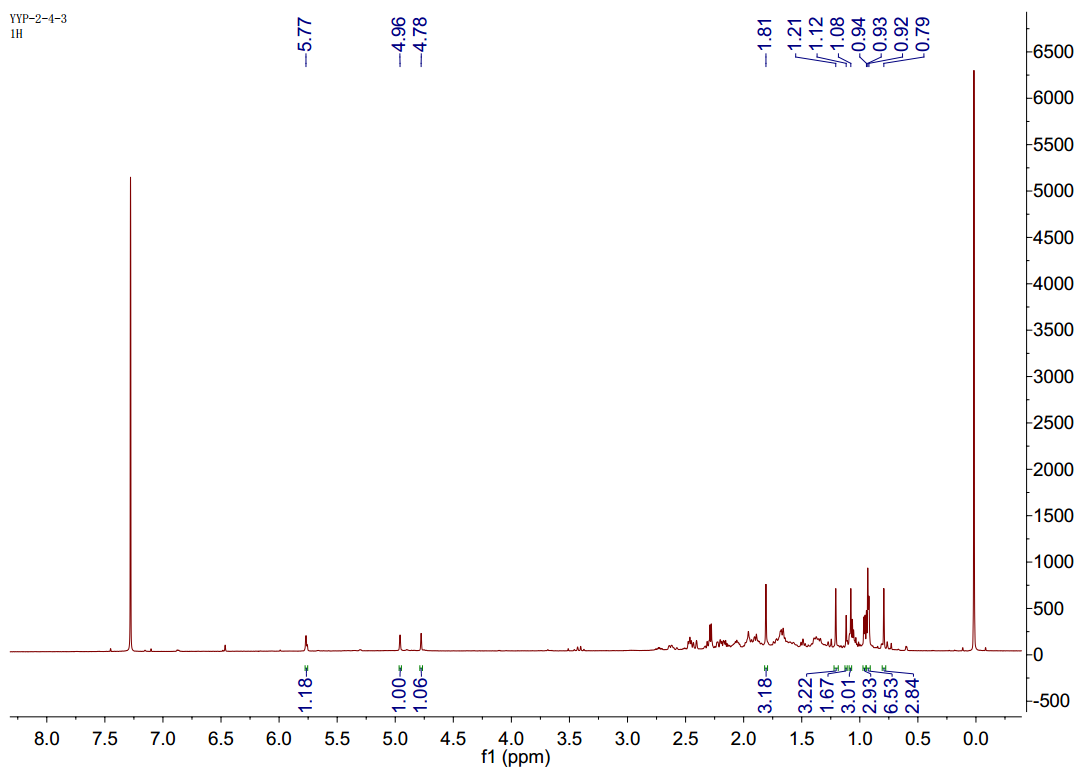
**Figure S16.** ^1^H NMR (600 MHz, CDCl_3_) spectrum of Heilaohuacid C (**3**)


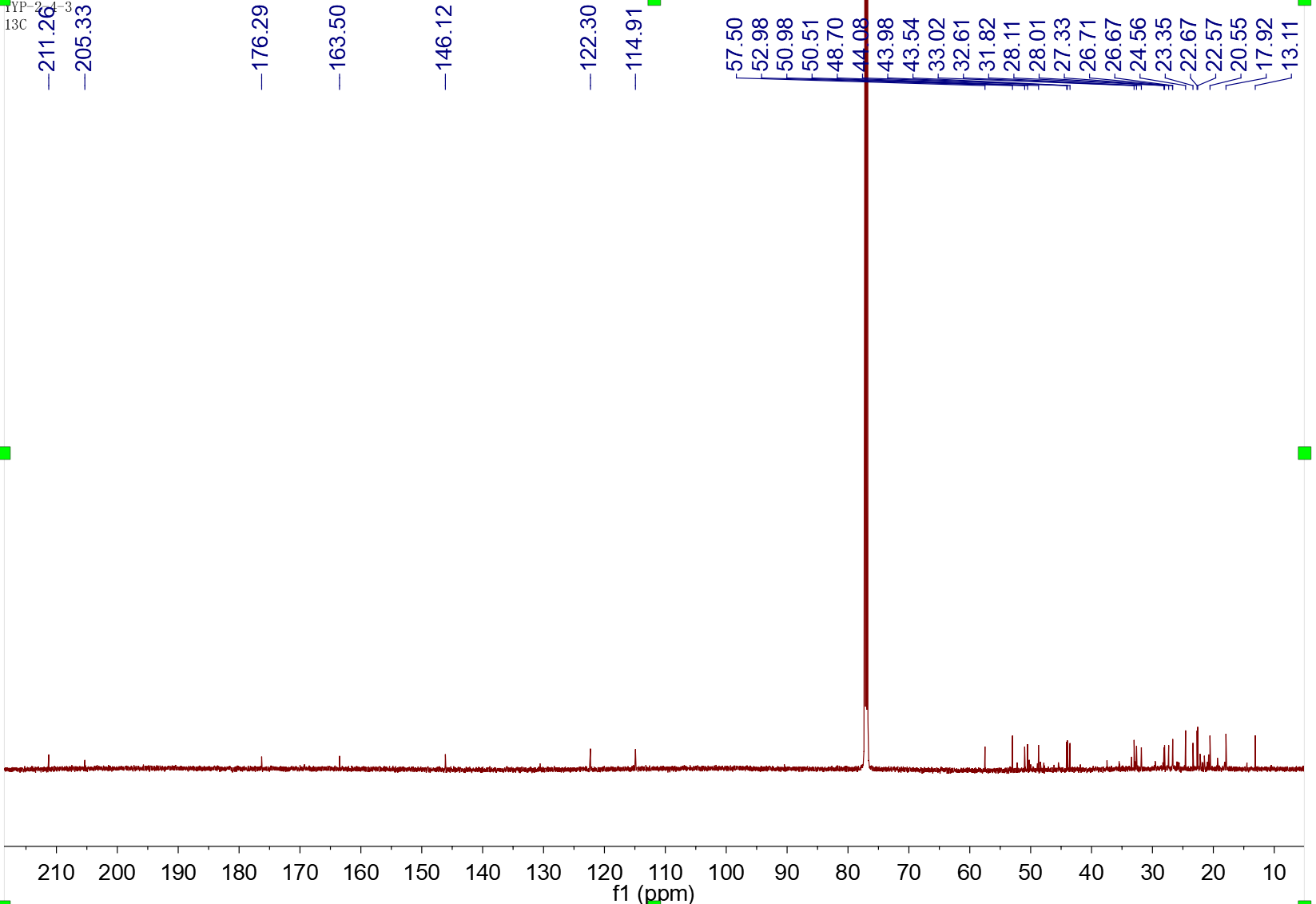


**Figure S17.** ^13^C NMR (150 MHz, CDCl_3_) spectrum of Heilaohuacid C (**3**)


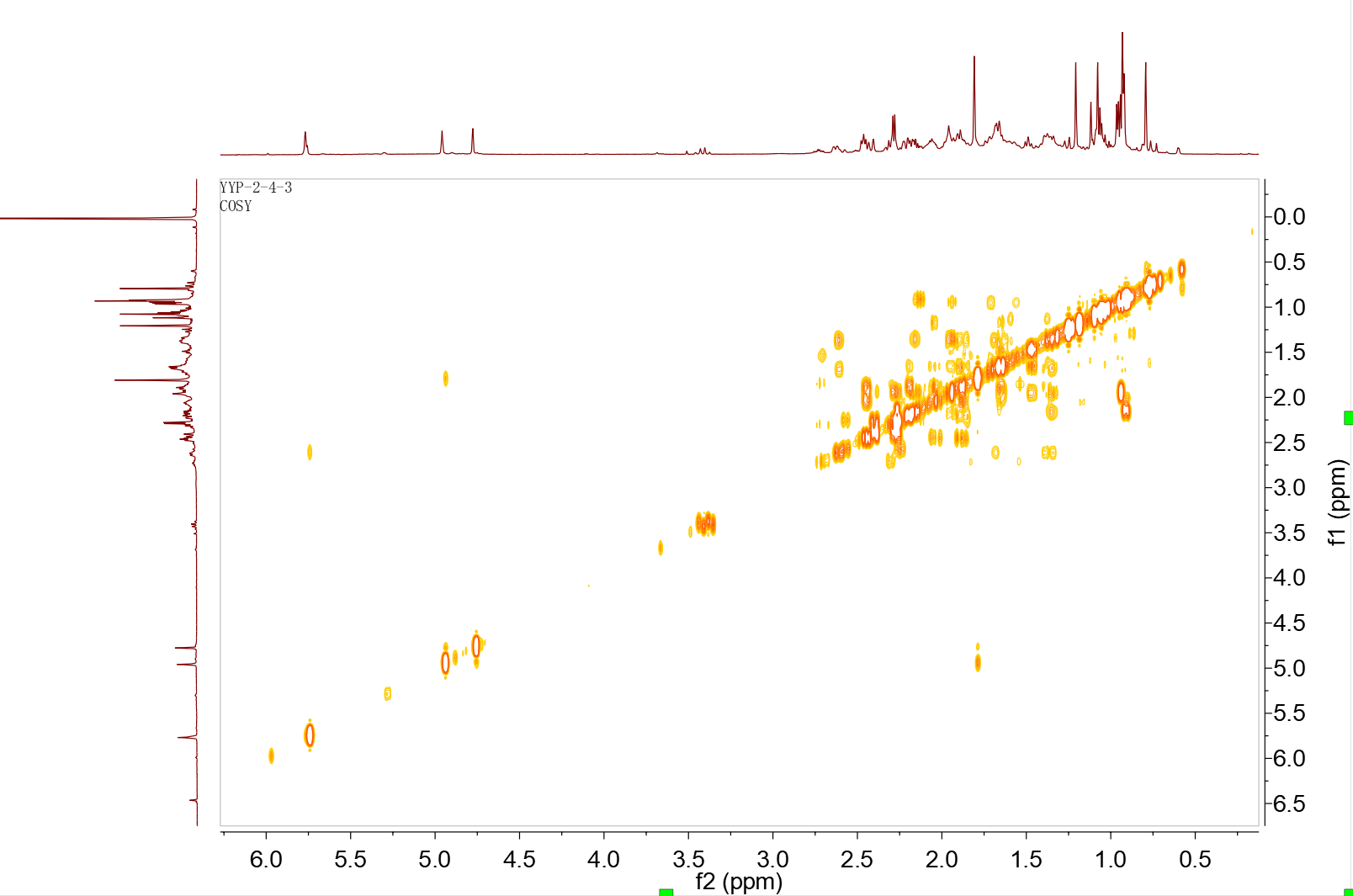


**Figure S18.** COSY NMR spectrum (CDCl_3_) of Heilaohuacid C (**3**)
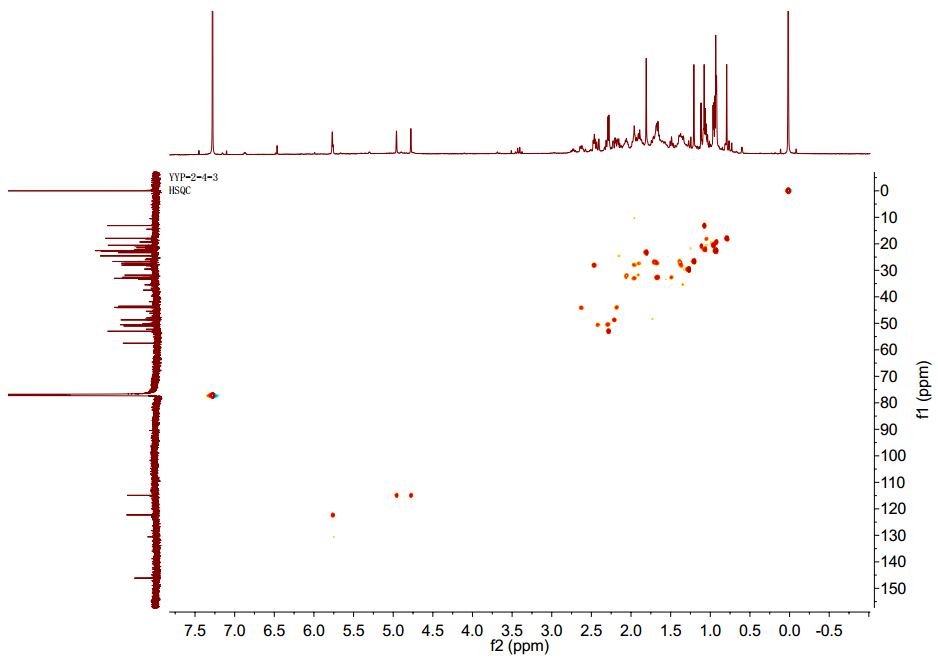


**Figure S19.** HSQC NMR spectrum (CDCl_3_) of Heilaohuacid C (**3**)


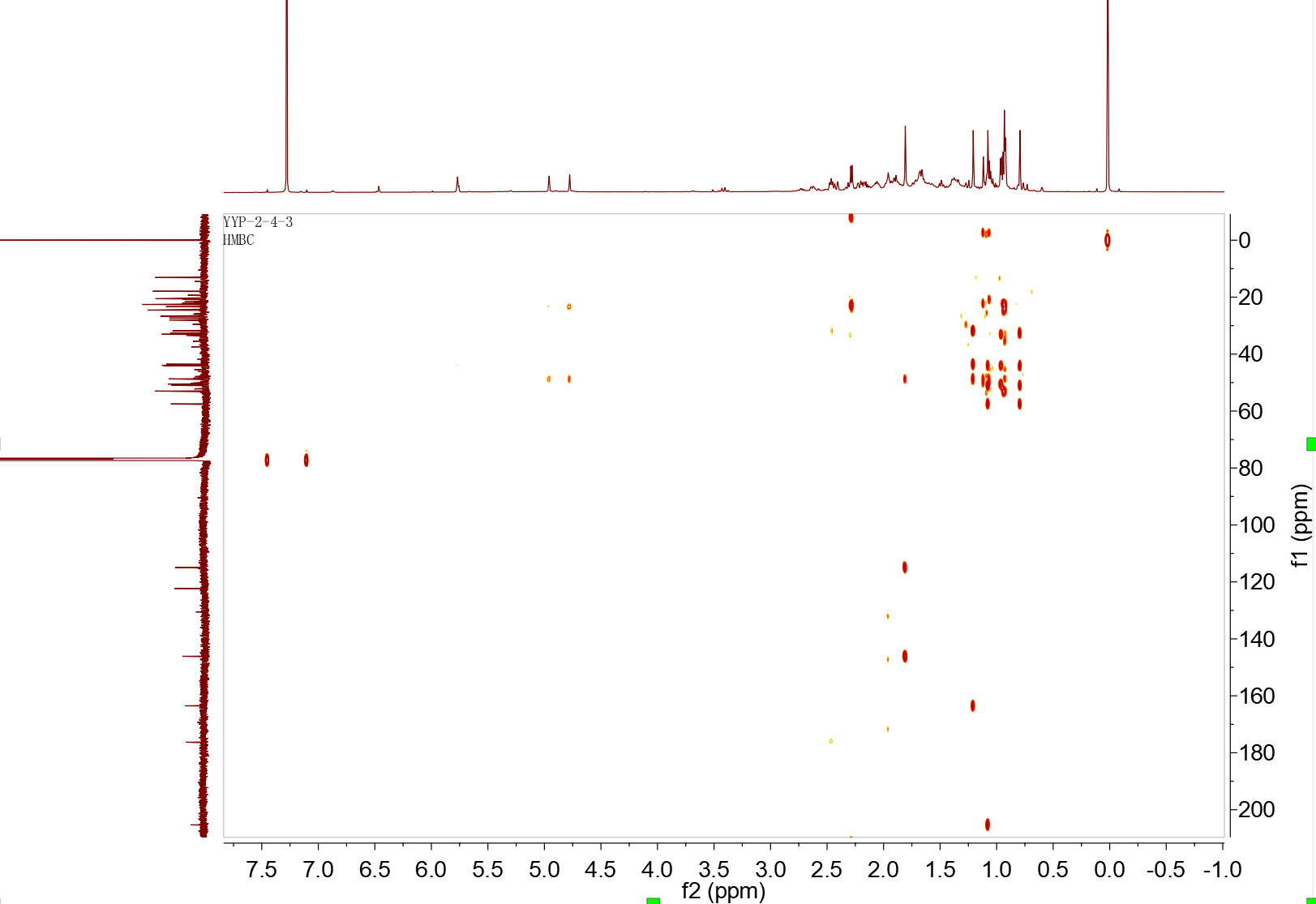


**Figure S20.** HMBC NMR spectrum (CDCl_3_) of Heilaohuacid C (**3**)


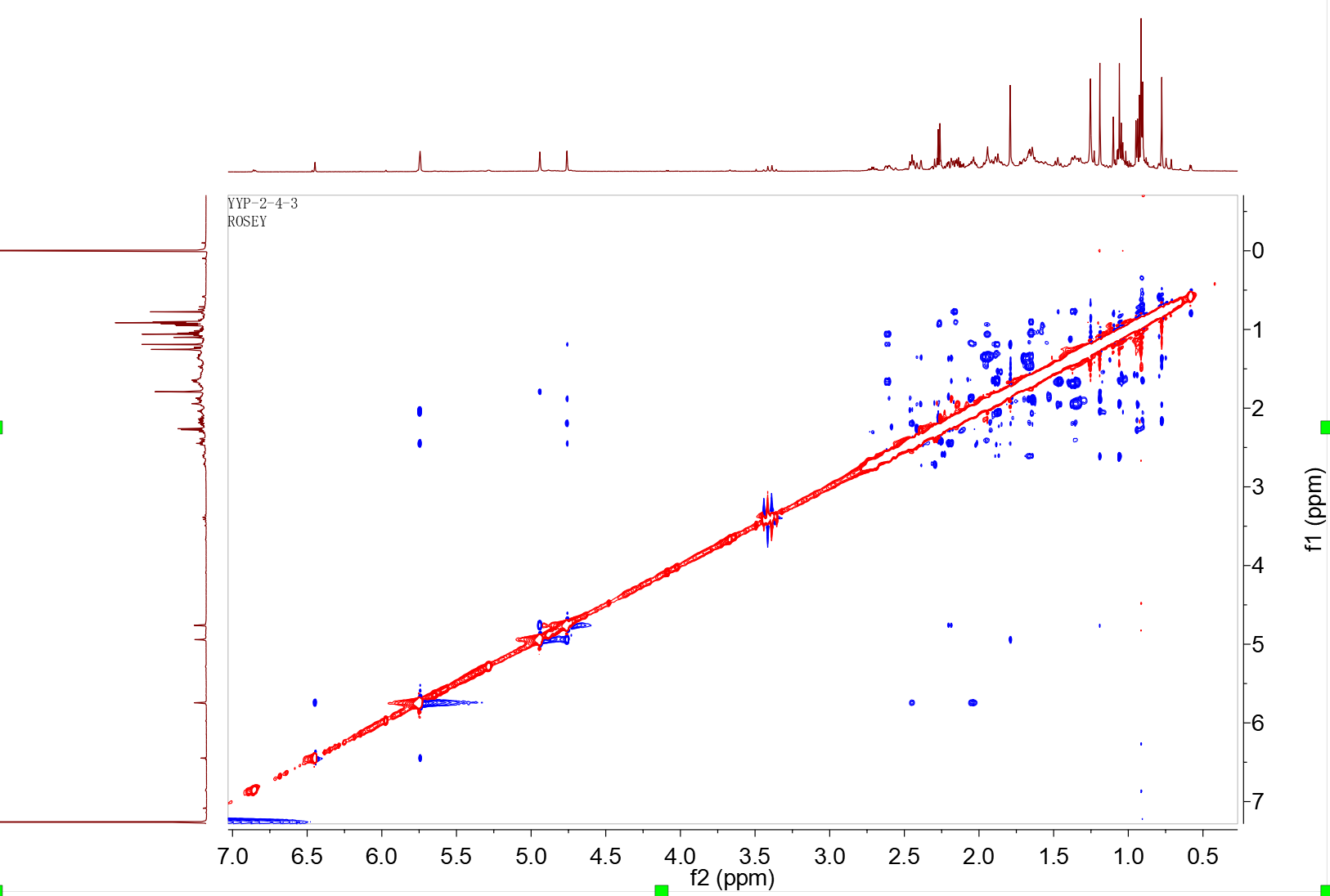


**Figure S21.** ROESY spectrum (CDCl_3_) of Heilaohuacid C (**3**)

**Figure S22.** HRESIMS spectrum of Heilaohuacid C (**3**)


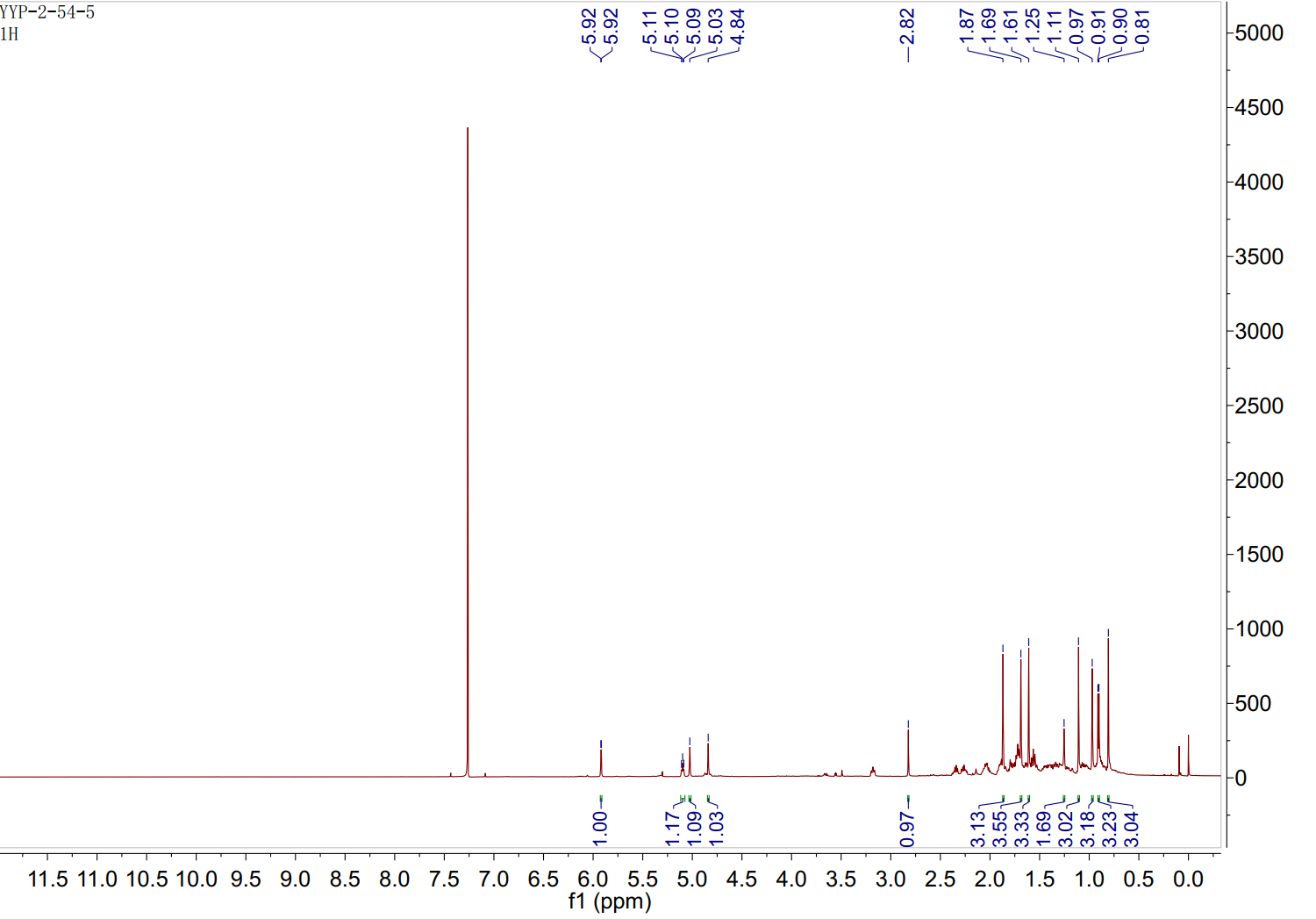


**Figure S23.** ^1^H NMR (600 MHz, CDCl_3_) spectrum of Heilaohuacid D (**4**)


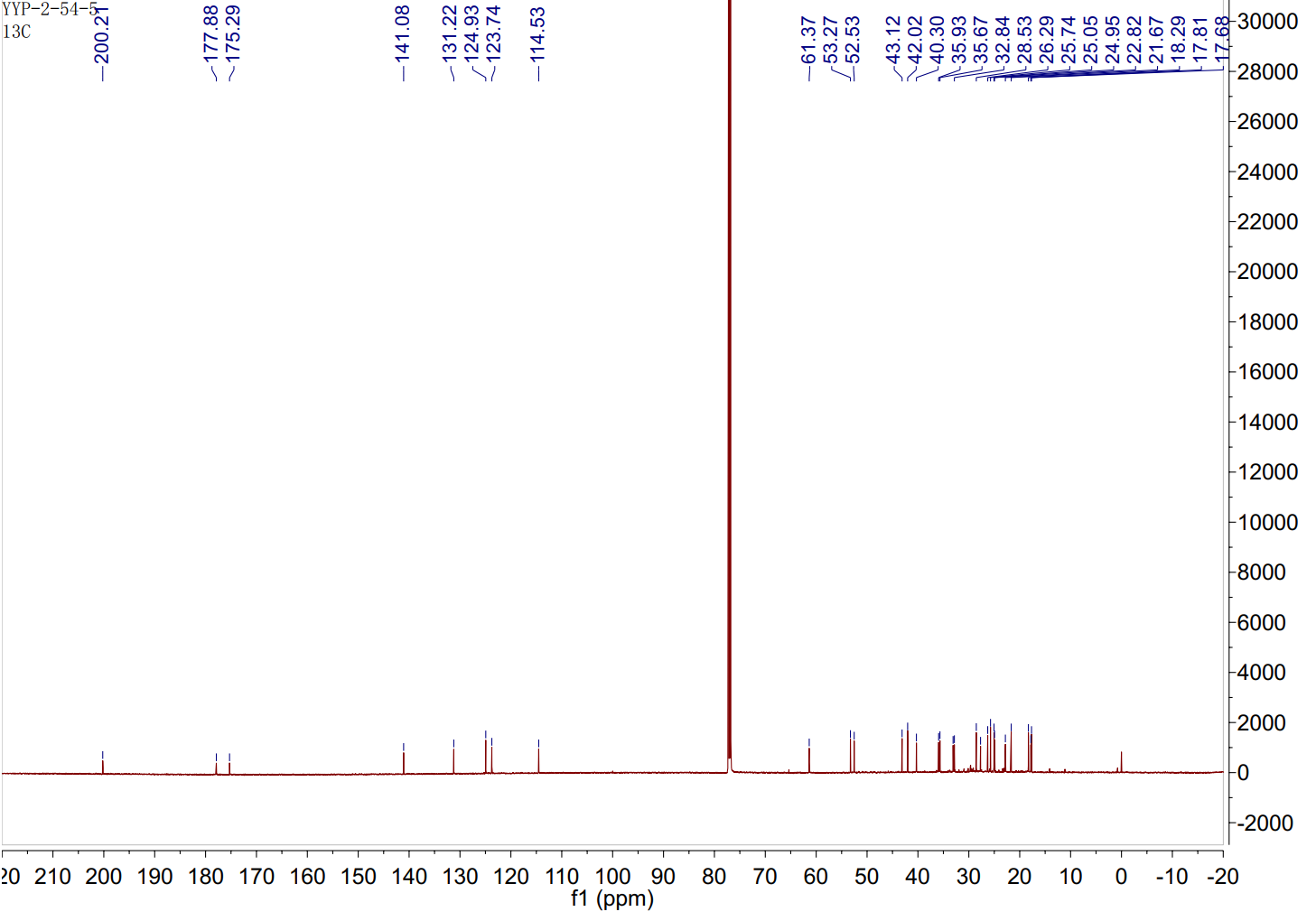


**Figure S24.** ^13^C NMR (150 MHz, CDCl_3_) spectrum of Heilaohuacid D (**4**)


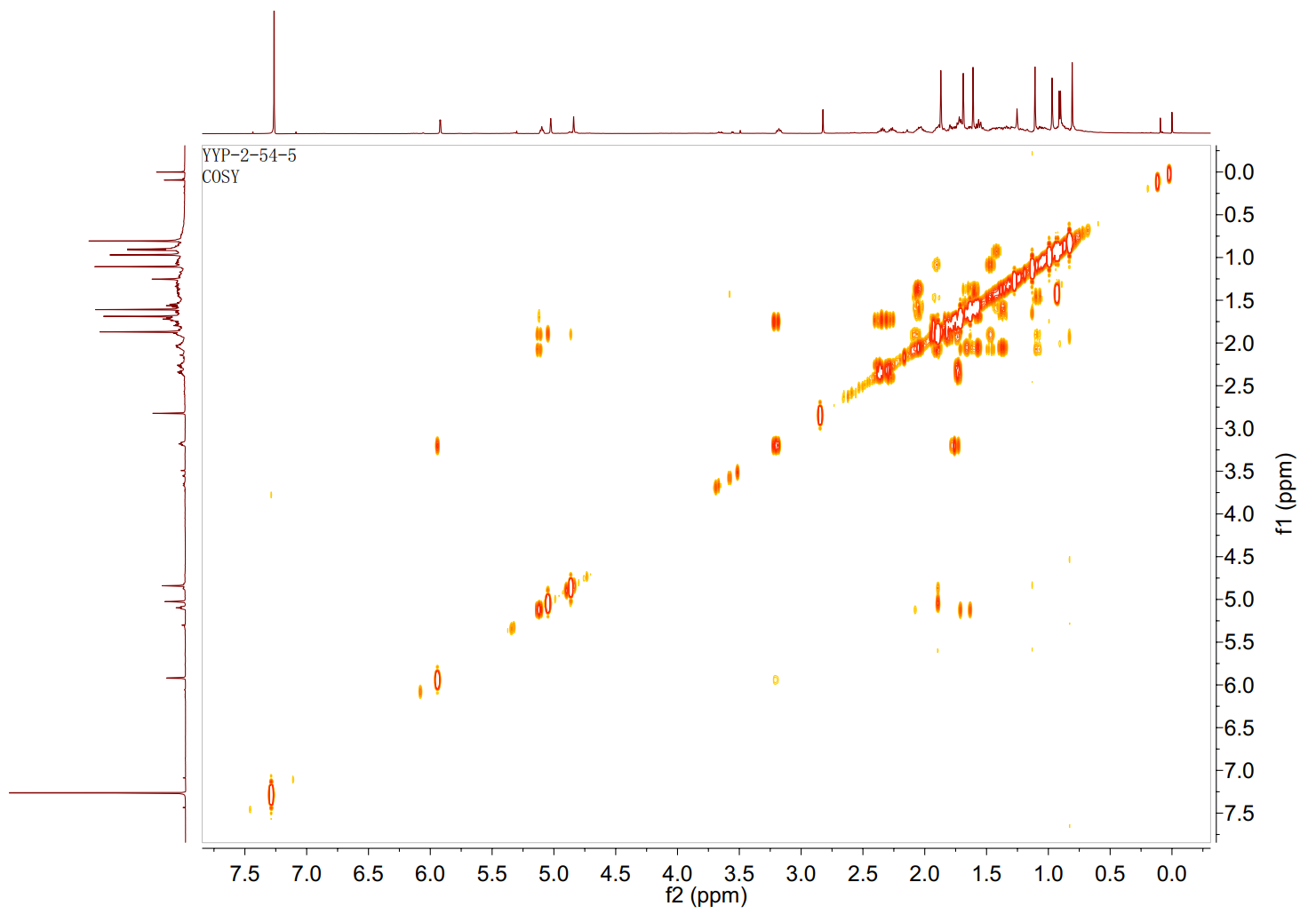


**Figure S25.** COSY NMR spectrum (CDCl_3_) of Heilaohuacid D (**4**)


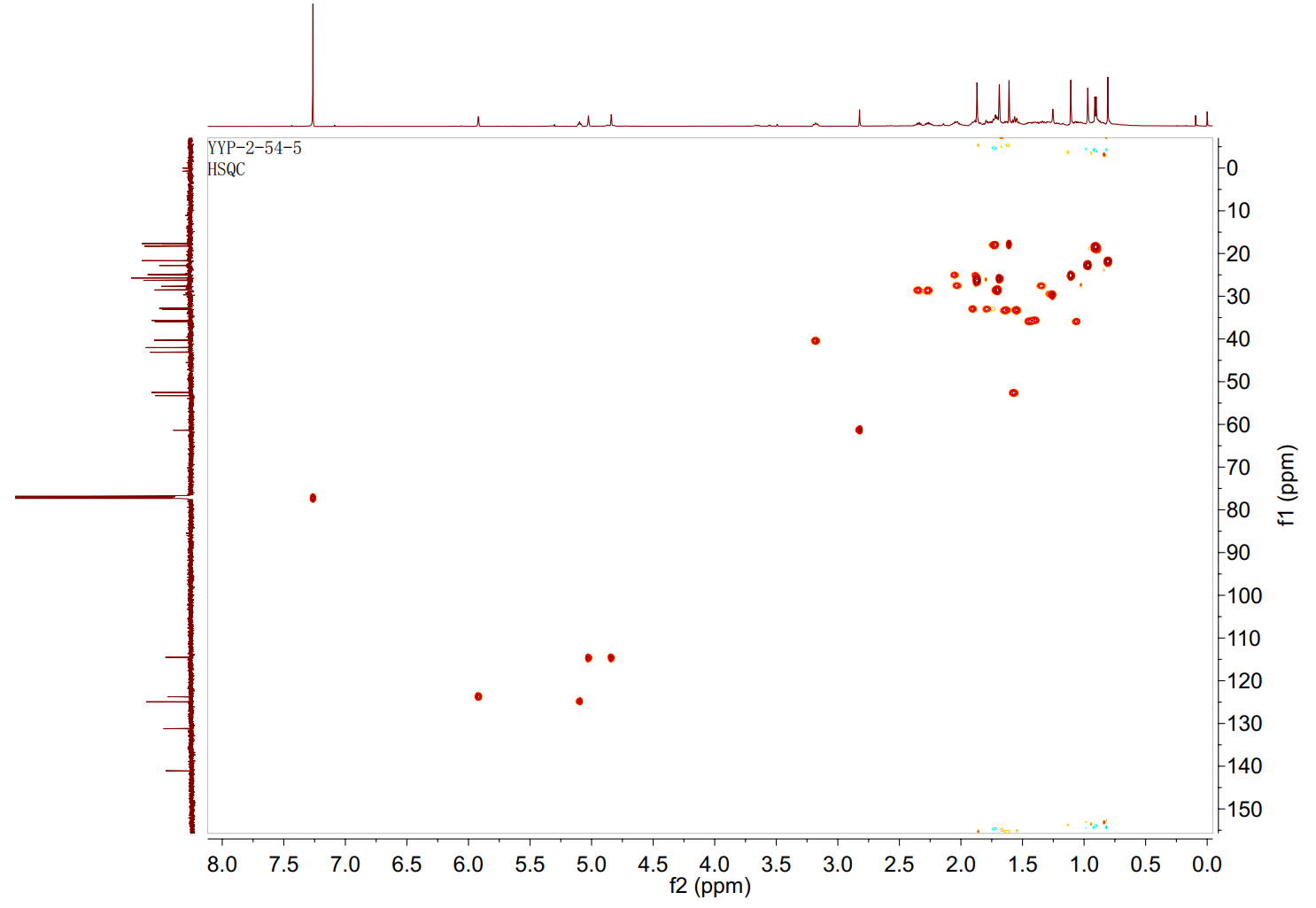


**Figure S26.** HSQC NMR spectrum (CDCl_3_) of Heilaohuacid D (**4**)


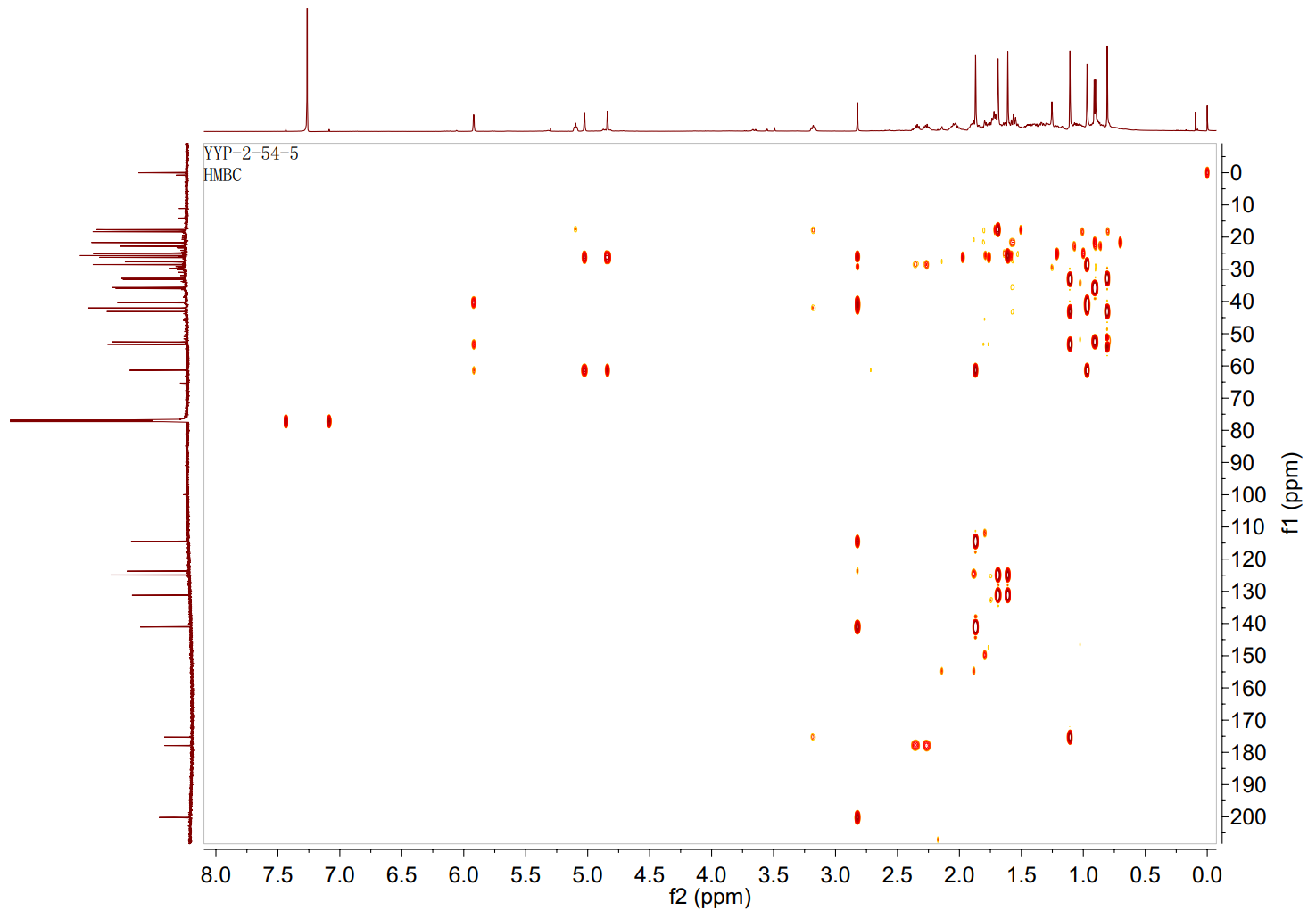


**Figure S27.** HMBC NMR spectrum (CDCl_3_) of Heilaohuacid D (**4**)


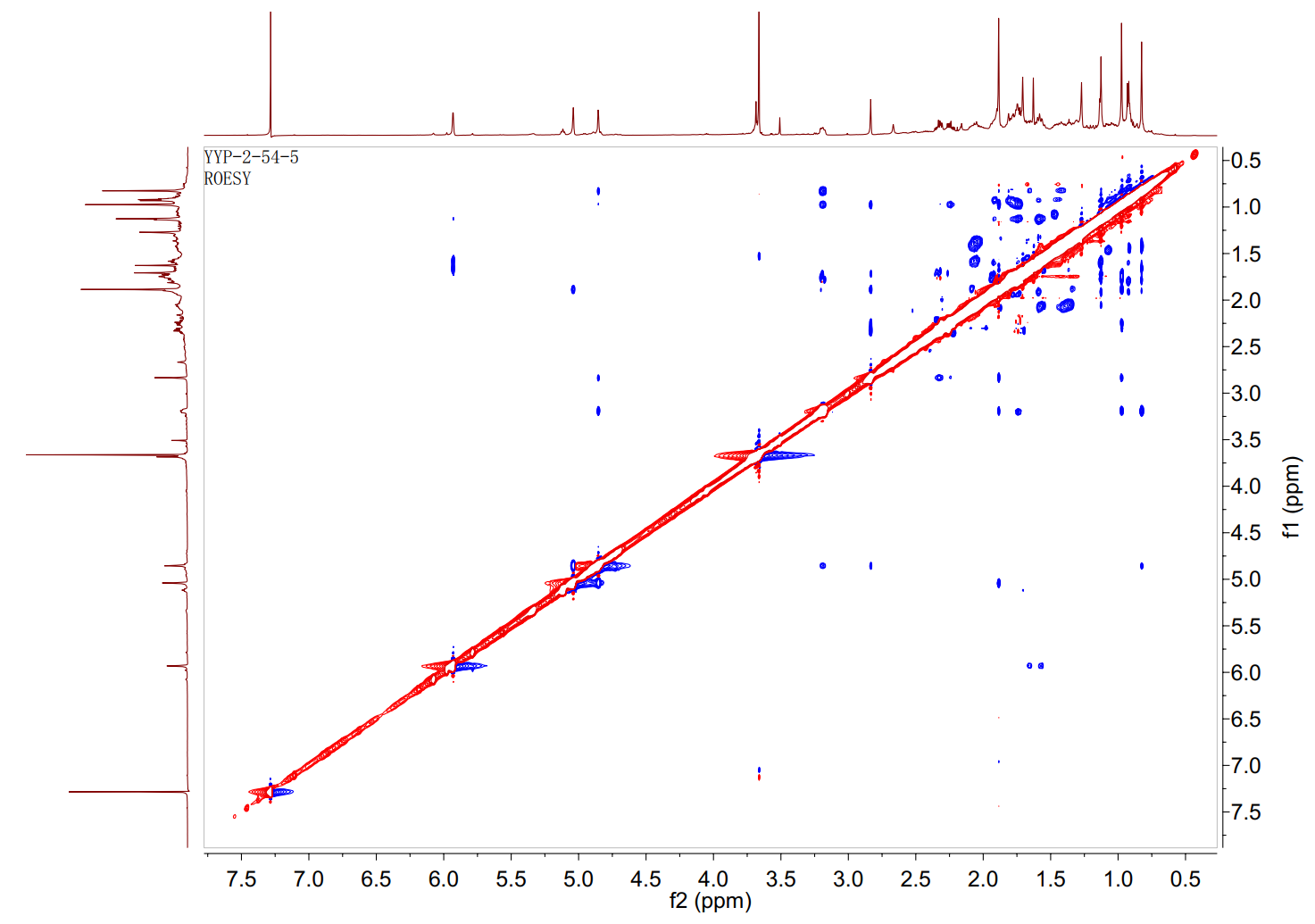


**Figure S28.** ROESY spectrum (CDCl_3_) of Heilaohuacid D (**4**)

**Figure S29.** HRESIMS spectrum of Heilaohuacid D (**4**)


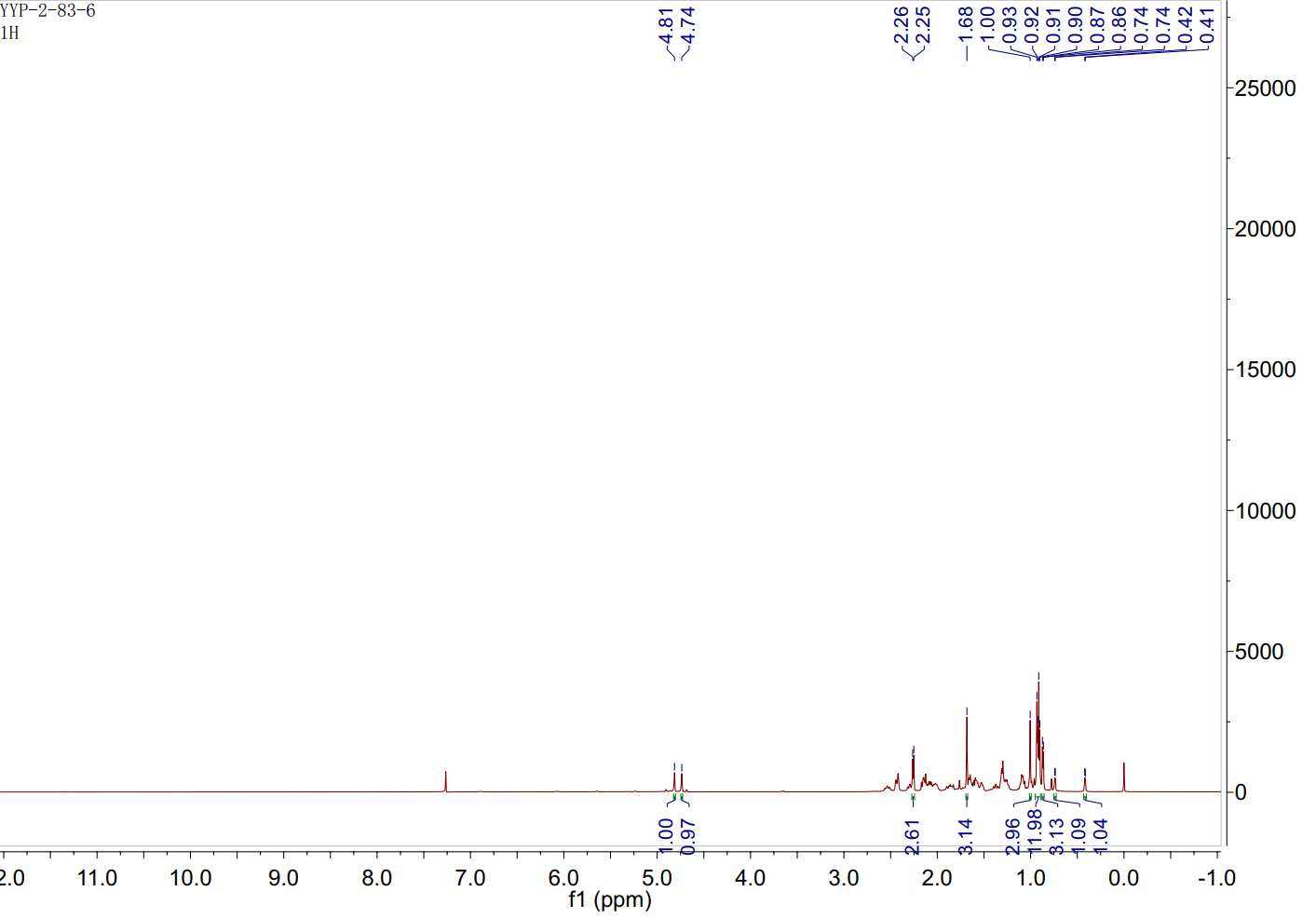


**Figure S30.** ^1^H NMR (600 MHz, CDCl_3_) spectrum of Heilaohuacid E (**5**)


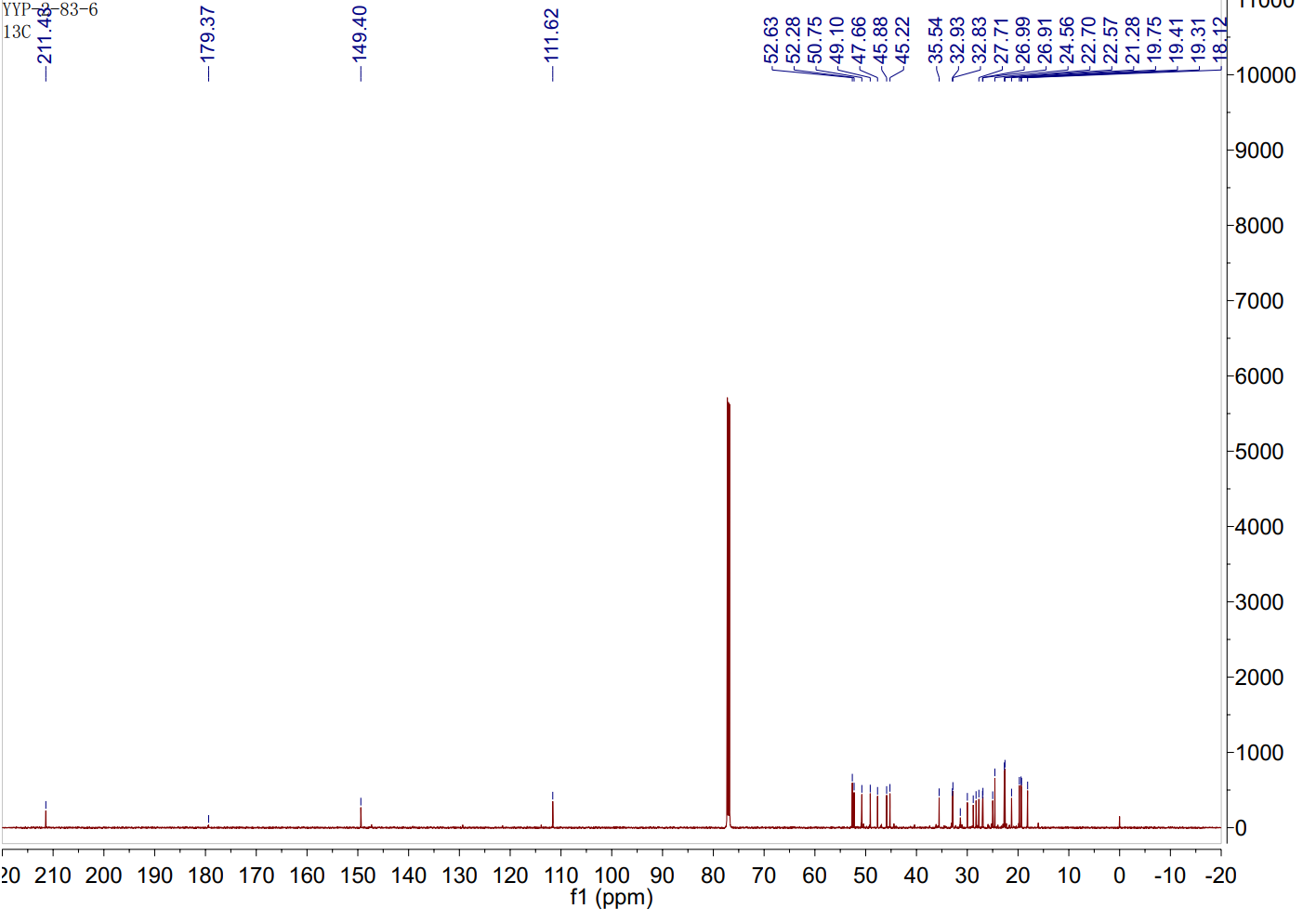


**Figure S31.** ^13^C NMR (150 MHz, CDCl_3_) spectrum of Heilaohuacid E (**5**)


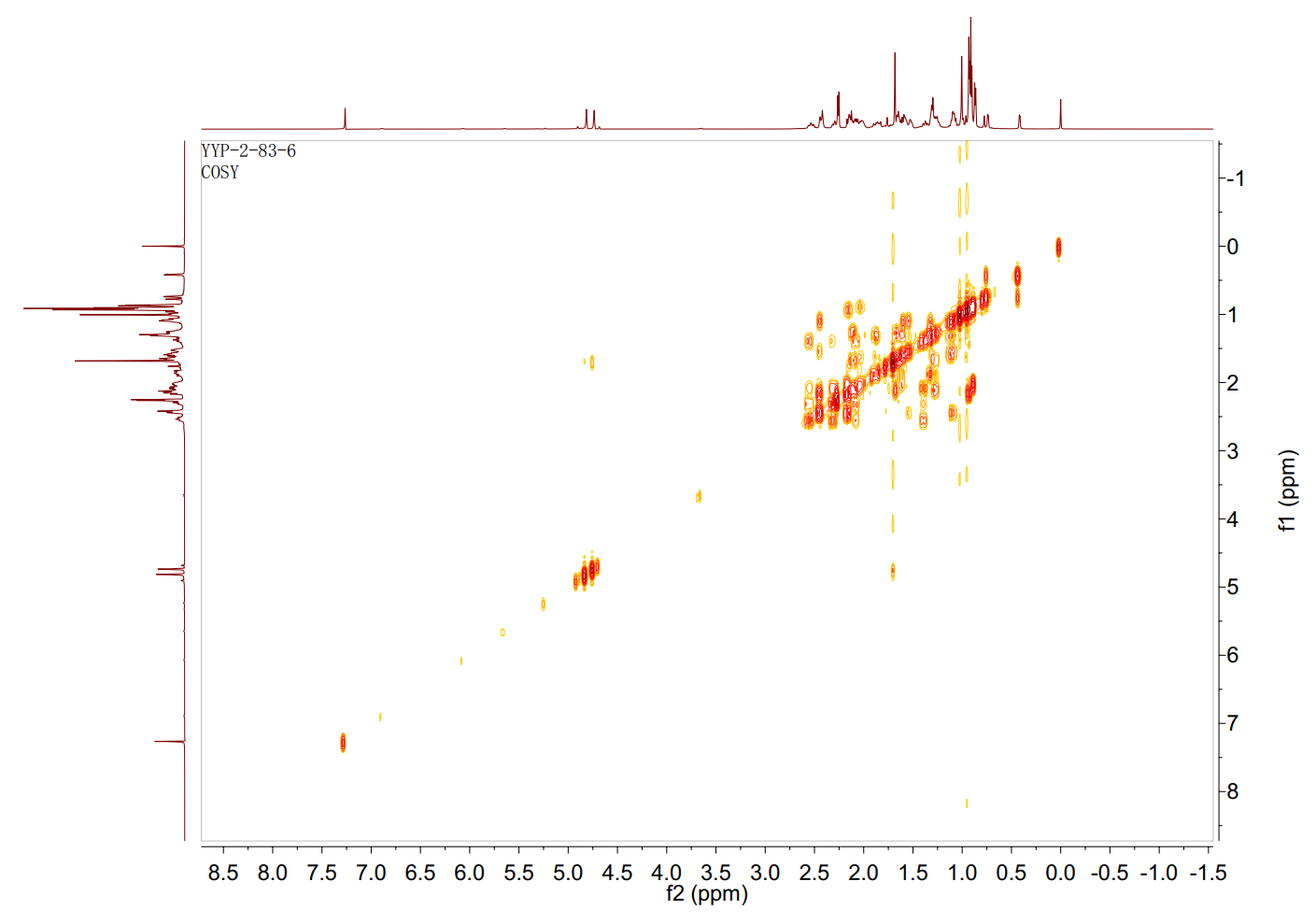


**Figure S32.** COSY NMR spectrum (CDCl_3_) of Heilaohuacid E (**5**)


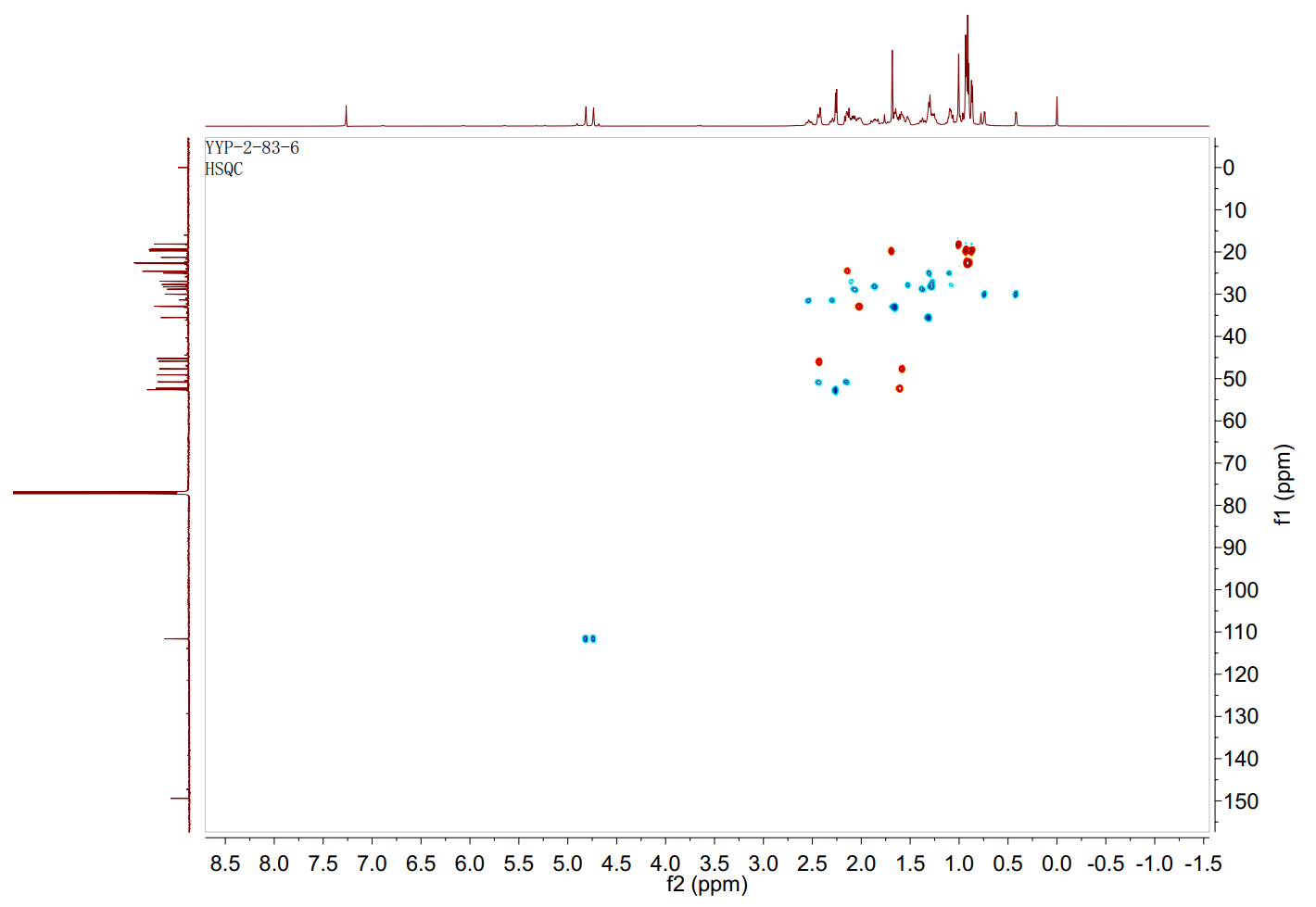


**Figure S33.** HSQC NMR spectrum (CDCl_3_) of Heilaohuacid E (**5**)


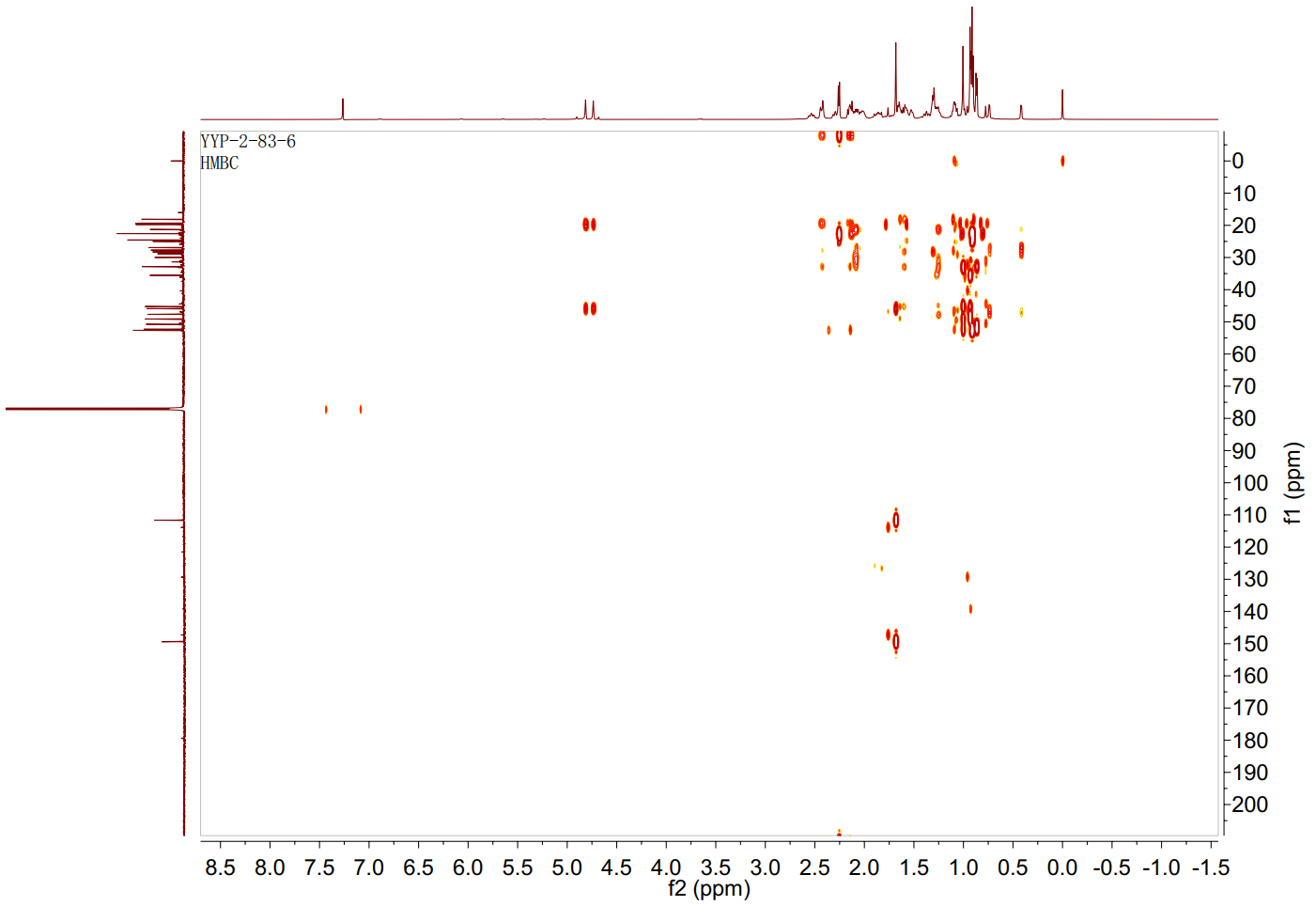


**Figure S34.** HMBC NMR spectrum (CDCl_3_) of Heilaohuacid E (**5**)


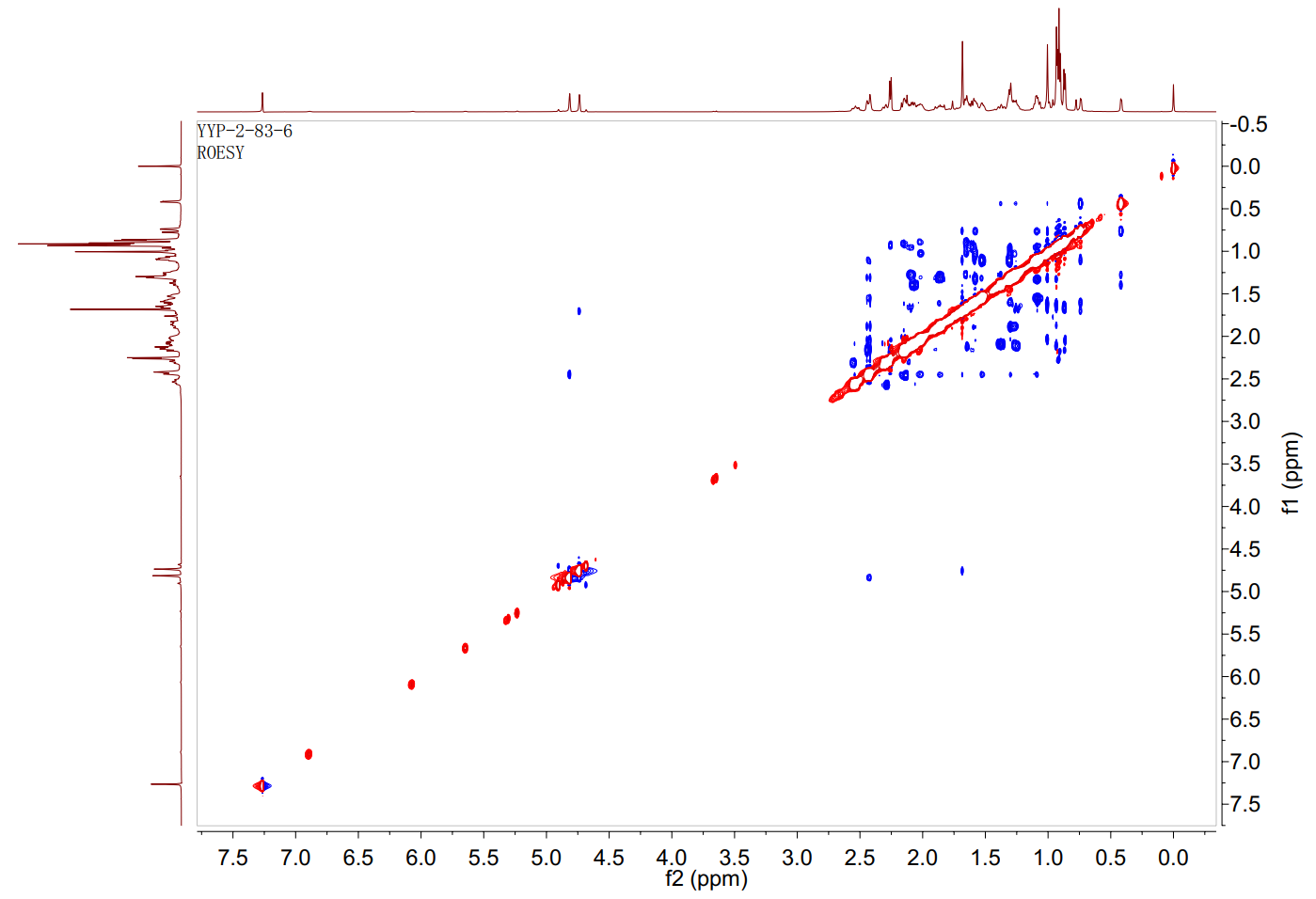


**Figure S35.** ROESY spectrum (CDCl_3_) of Heilaohuacid E (**5**)

**Figure S36.** HRESIMS spectrum of Heilaohuacid E (**5**)


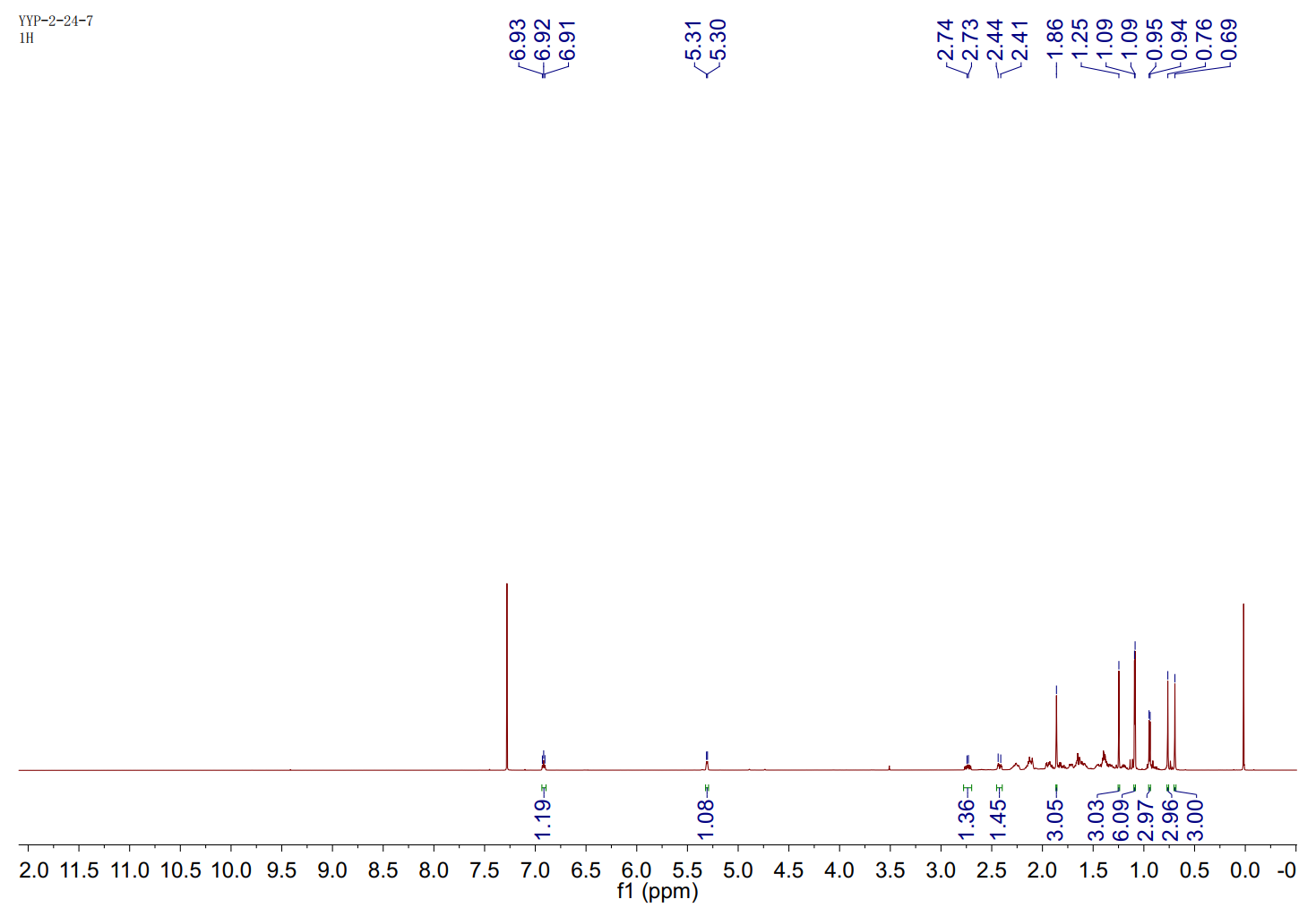


**Figure S37.** ^1^H NMR (600 MHz, CDCl_3_) spectrum of Heilaohuacid F (**6**)


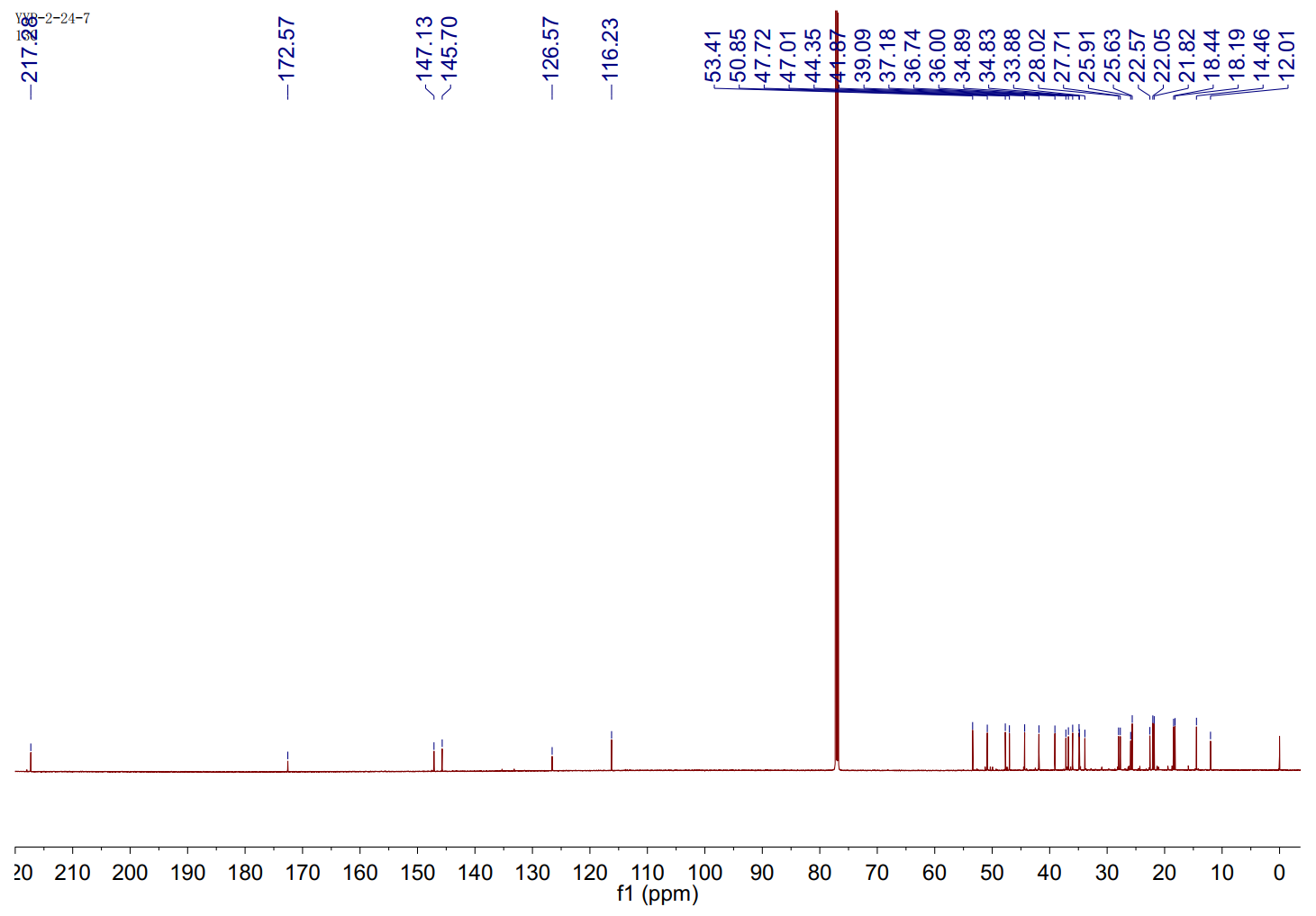


**Figure S38.** ^13^C NMR (150 MHz, CDCl_3_) spectrum of Heilaohuacid F (**6**)


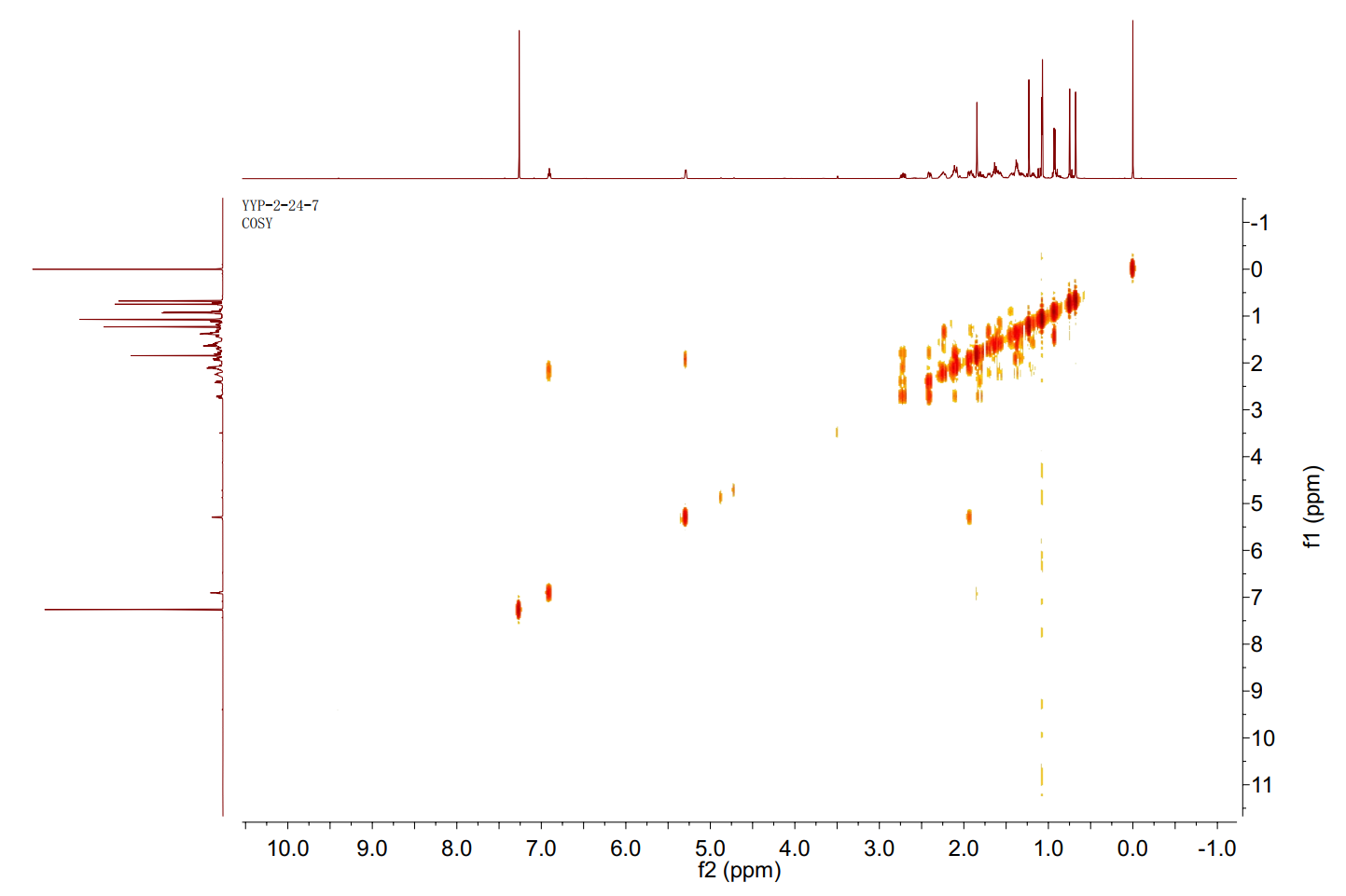


**Figure S39.** COSY NMR spectrum (CDCl_3_) of Heilaohuacid F (**6**)


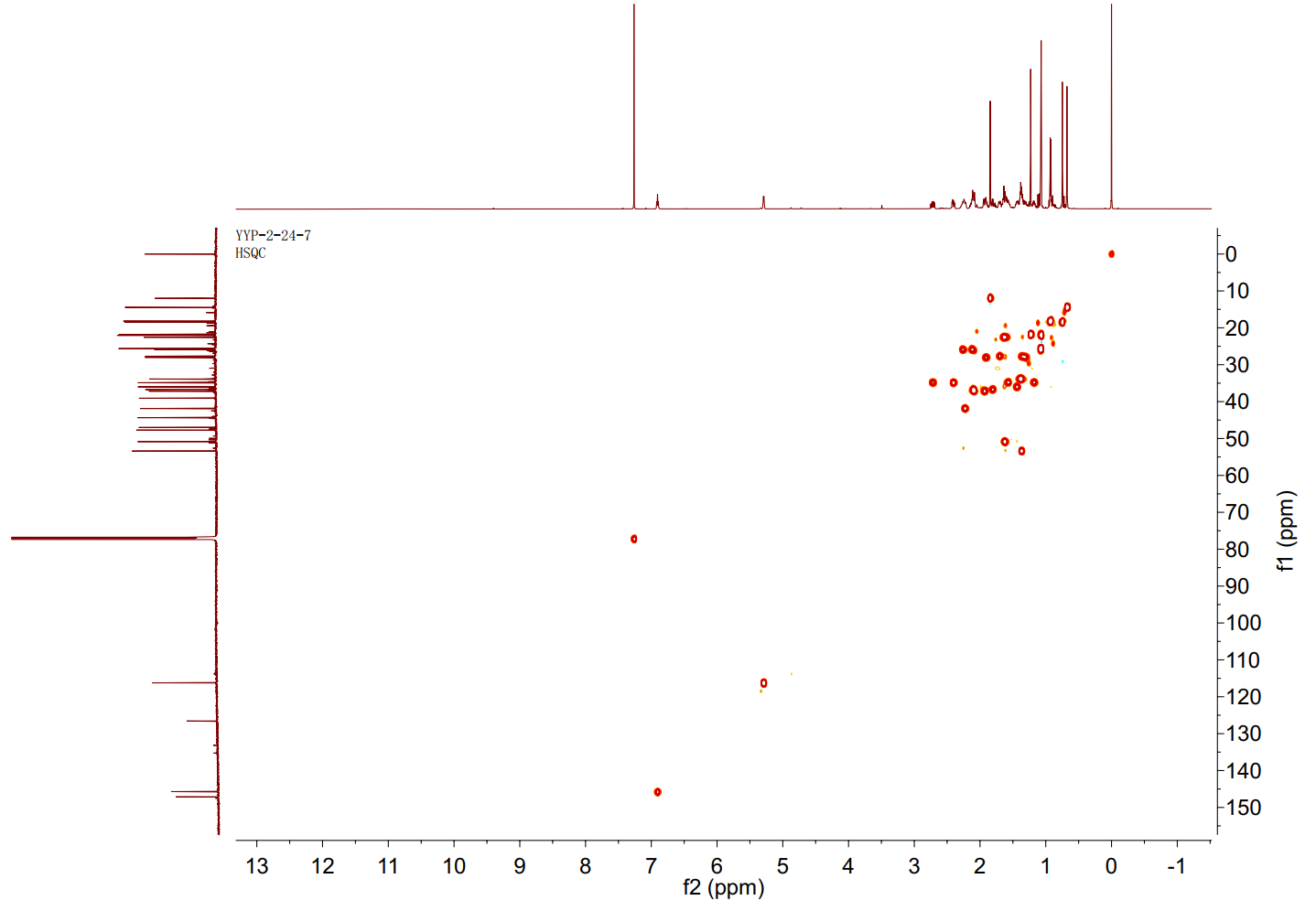


**Figure S40.** HSQC NMR spectrum (CDCl_3_) of Heilaohuacid F (**6**)


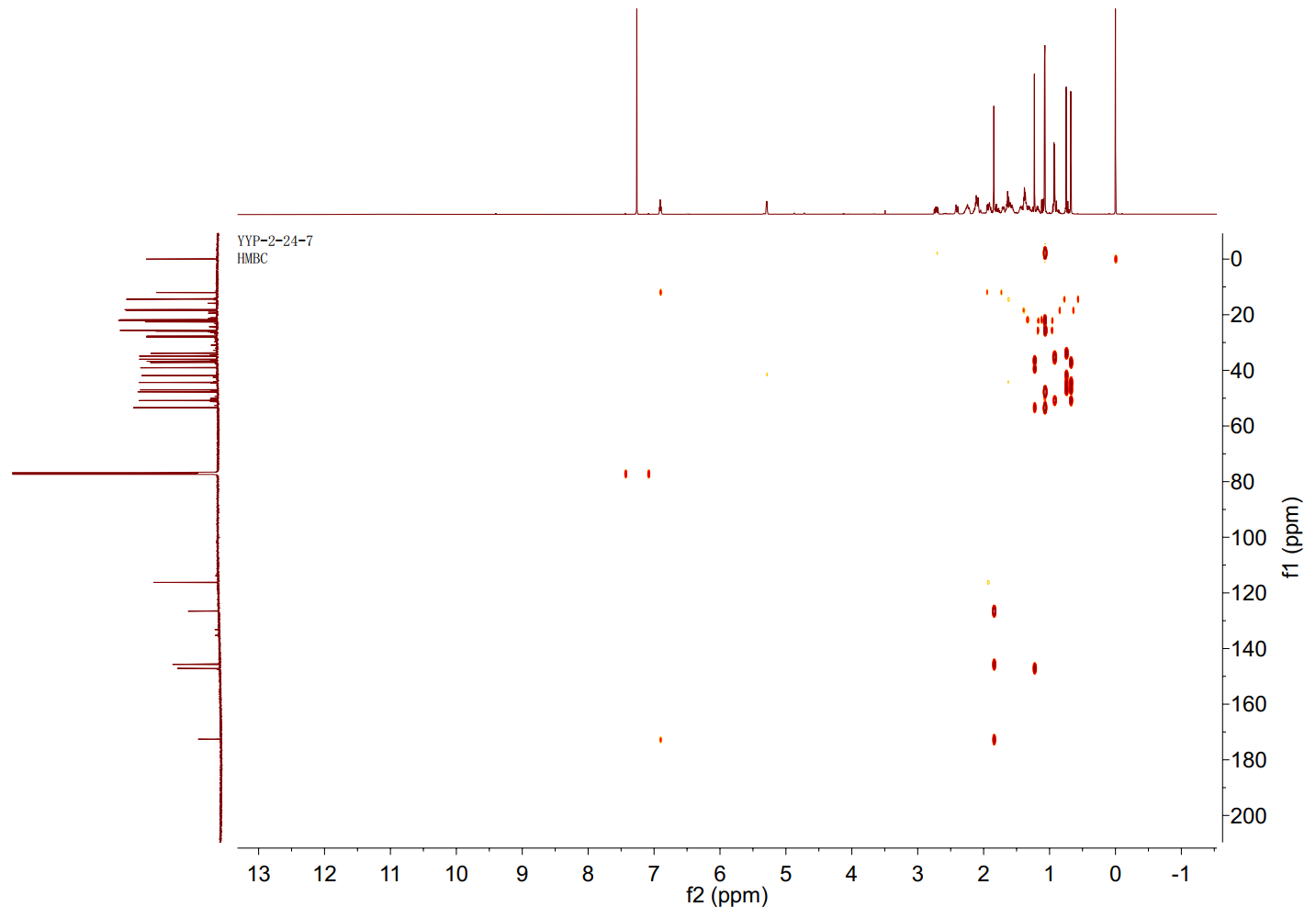


**Figure S41.** HMBC NMR spectrum (CDCl_3_) of Heilaohuacid F (**6**)


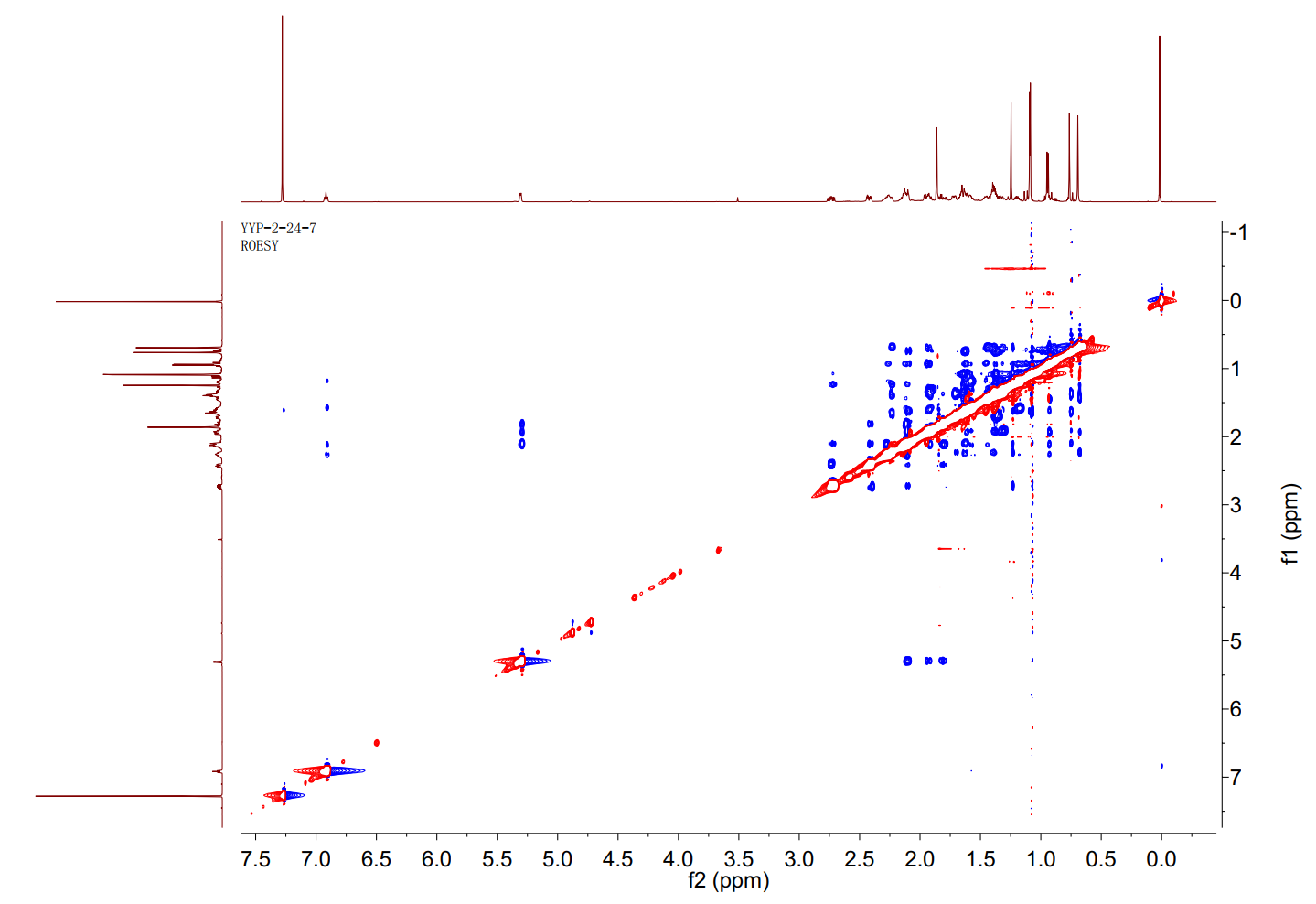


**Figure S42.** ROESY NMR spectrum (CDCl_3_) of Heilaohuacid F (**6**)

**Figure S43.** HRESIMS spectrum of Heilaohuacid F (**6**)


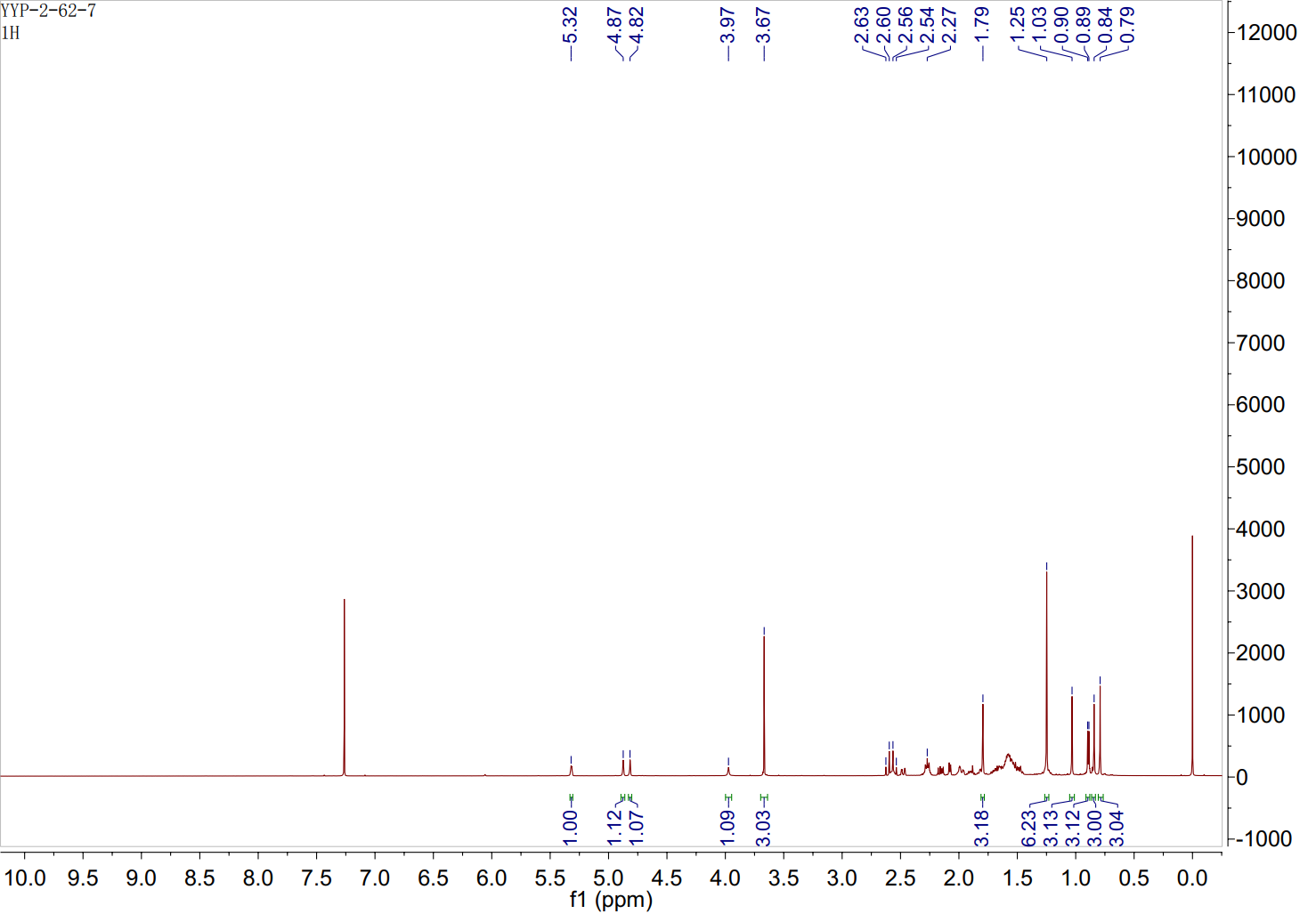


**Figure S44.** ^1^H NMR (600 MHz, CDCl_3_) spectrum of Heilaohumethylester A (**7**)


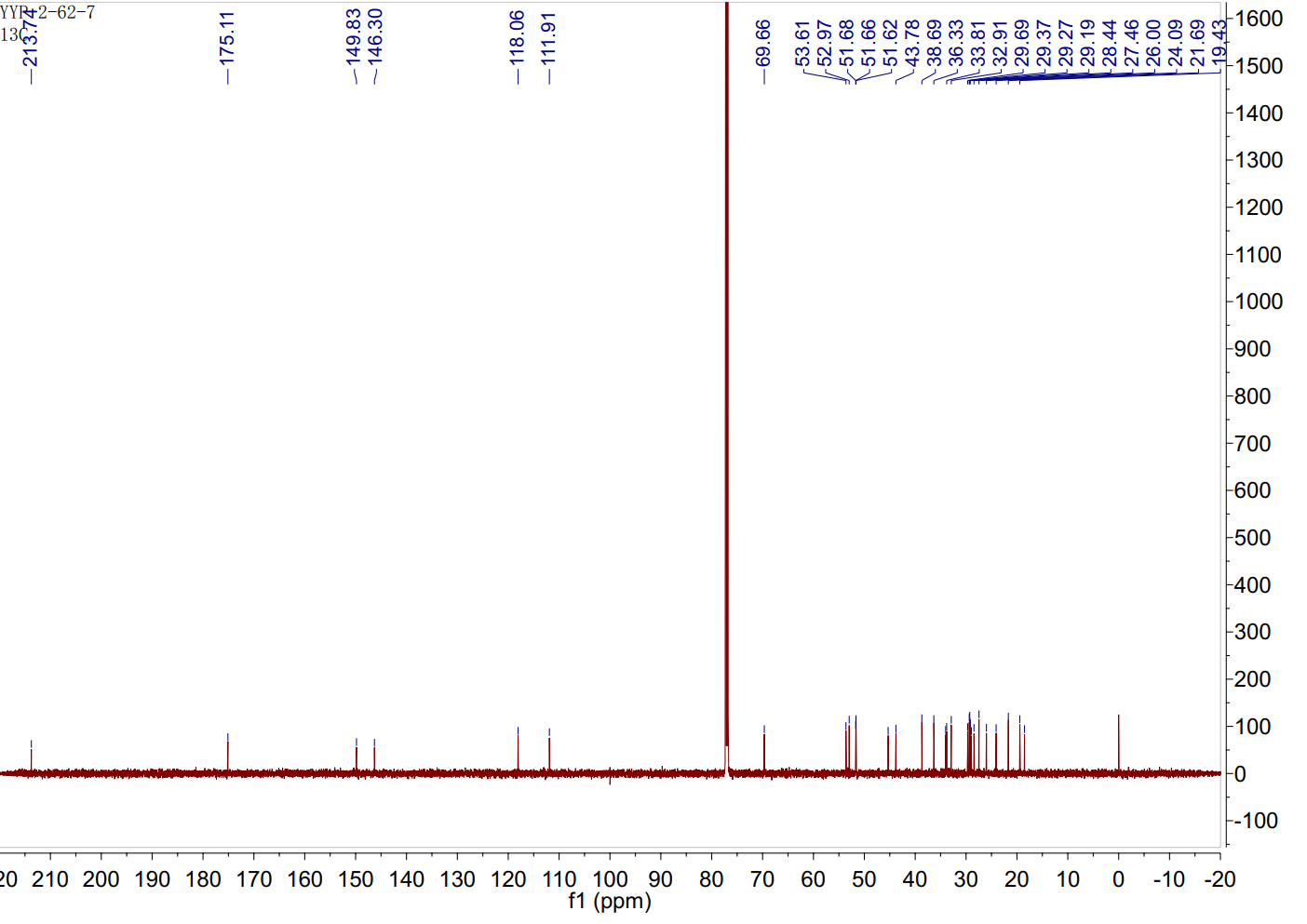


**Figure S45.** ^13^C NMR (150 MHz, CDCl_3_) spectrum of Heilaohumethylester A (**7**)


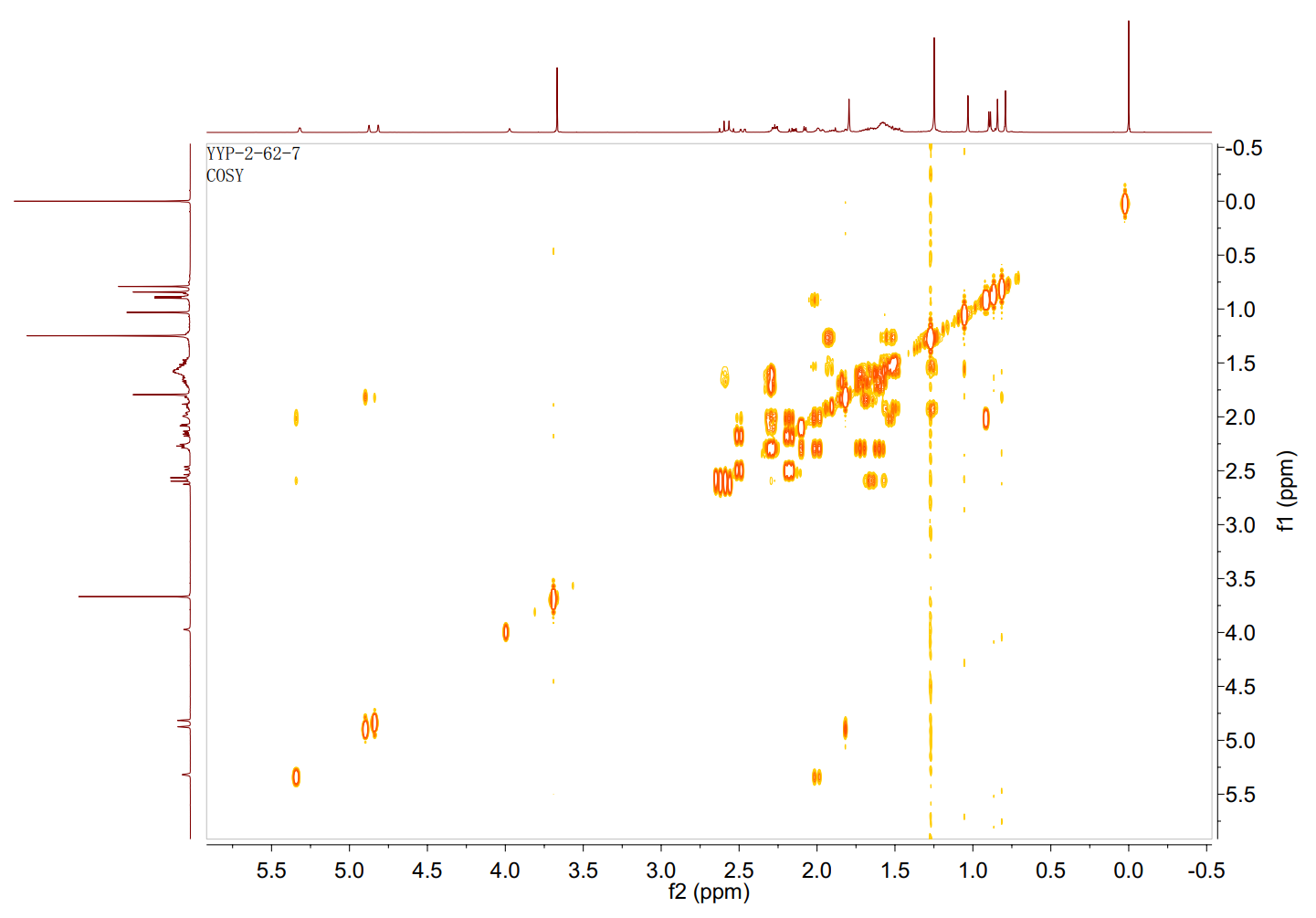


**Figure S46.** COSY NMR spectrum (CDCl_3_) of Heilaohumethylester A (**7**)


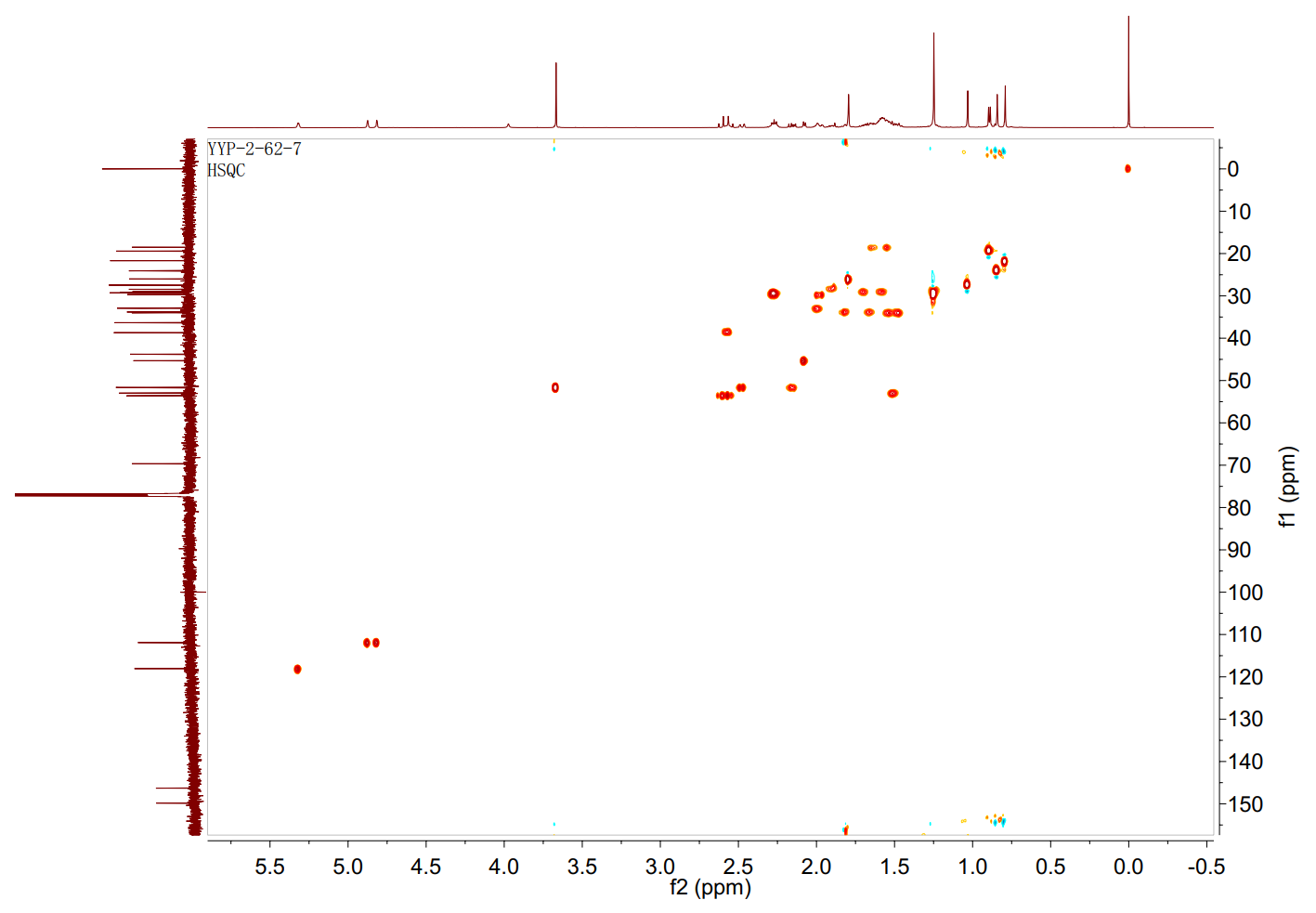


**Figure S47.** HSQC NMR spectrum (CDCl_3_) of Heilaohumethylester A (**7**)


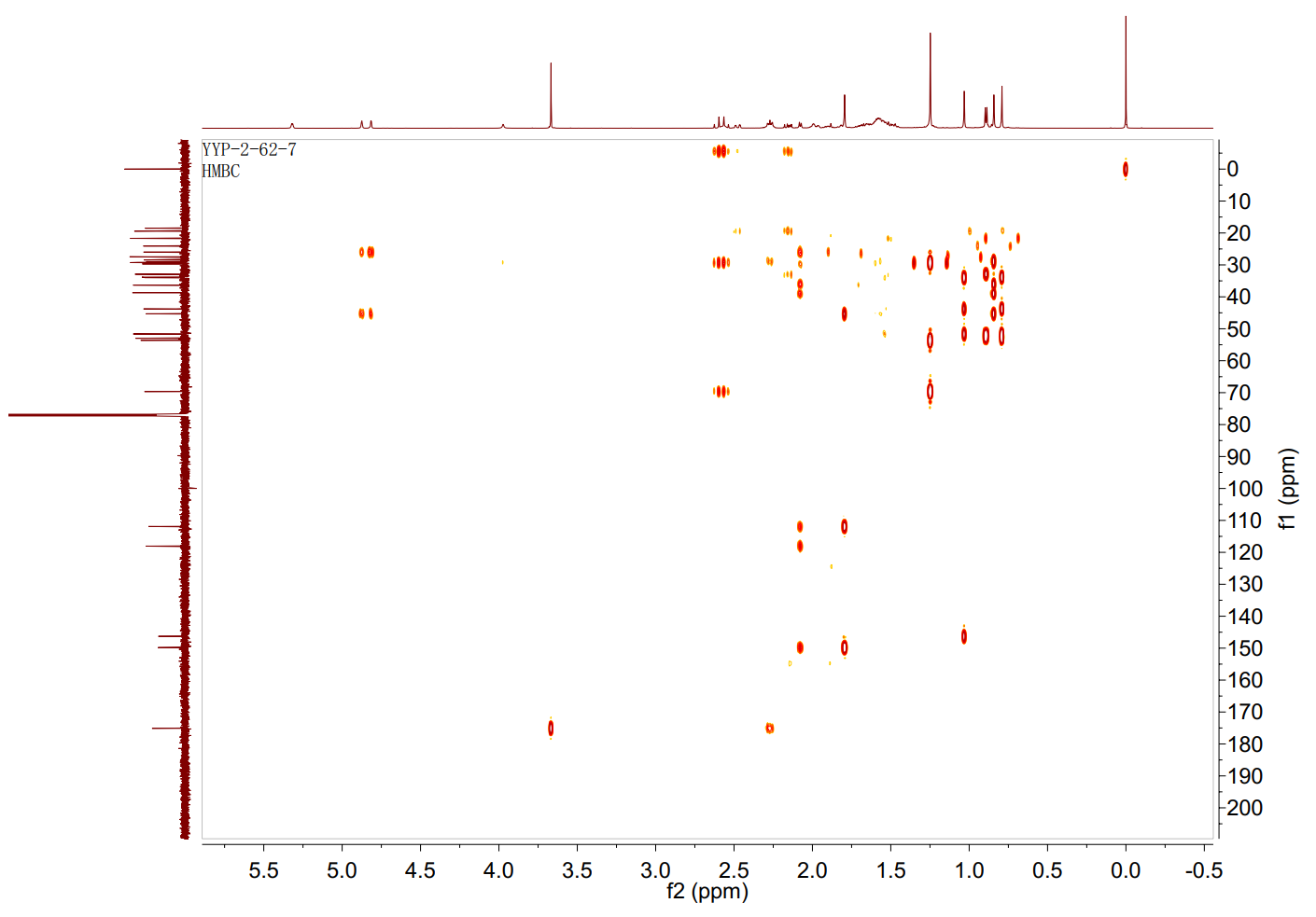


**Figure S48.** HMBC NMR spectrum (CDCl_3_) of Heilaohumethylester A (**7**)


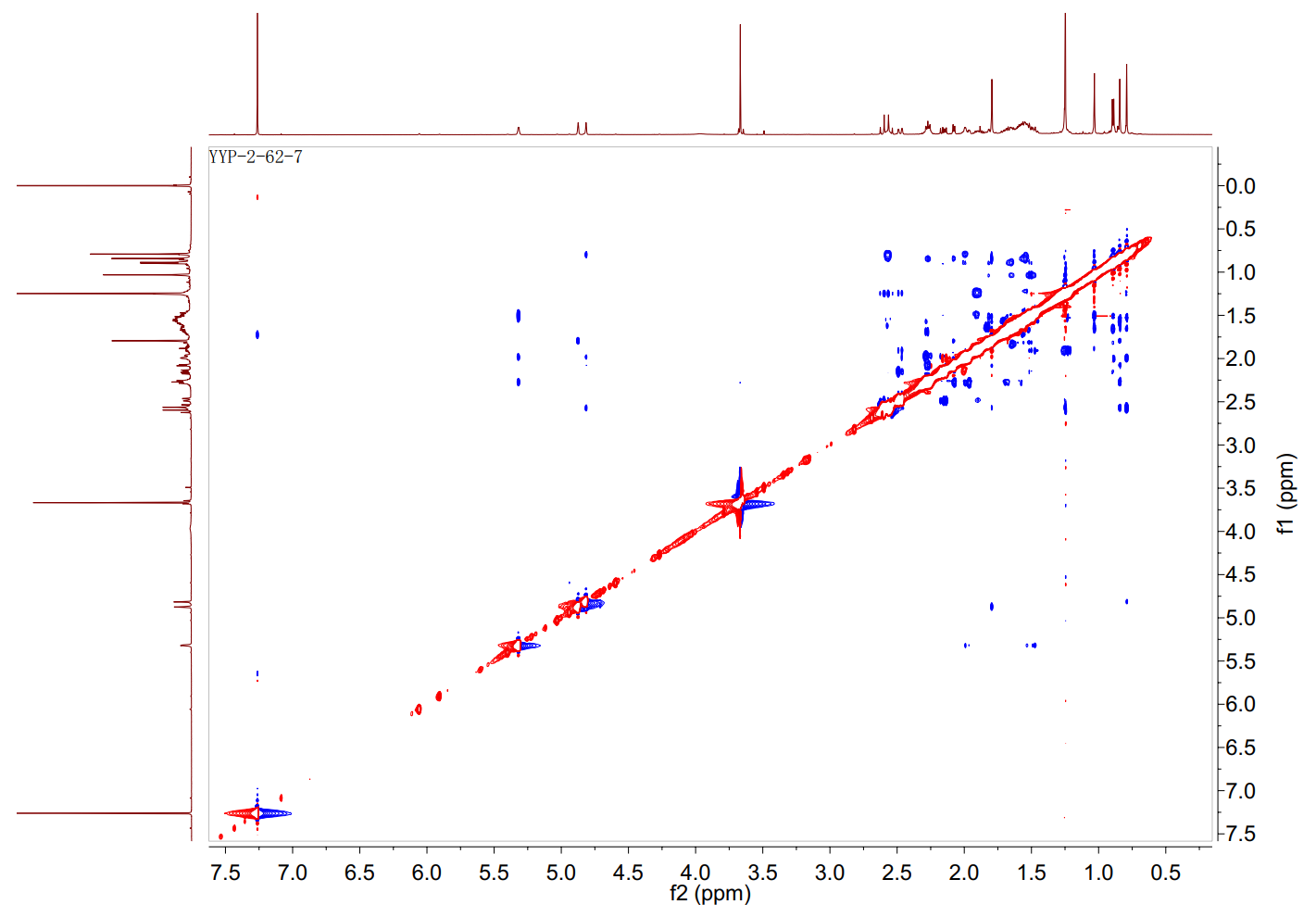


**Figure S49.** ROESY spectrum (CDCl_3_) of Heilaohumethylester A (**7**)

**Figure S50.** HRESIMS spectrum of Heilaohumethylester A (**7**)


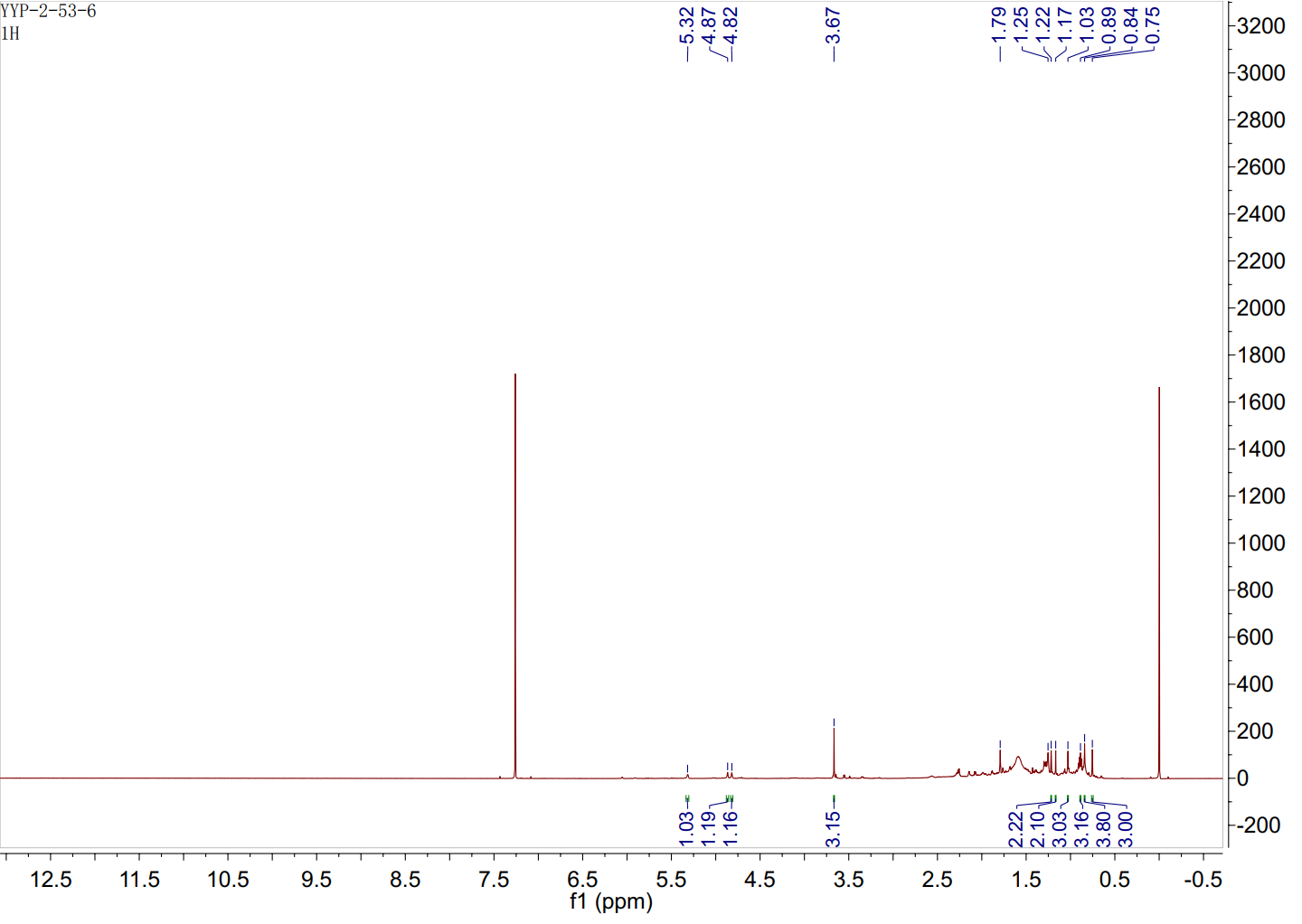


**Figure S51.** ^1^H NMR (600 MHz, CDCl_3_) spectrum of Heilaohumethylester B (**8**)


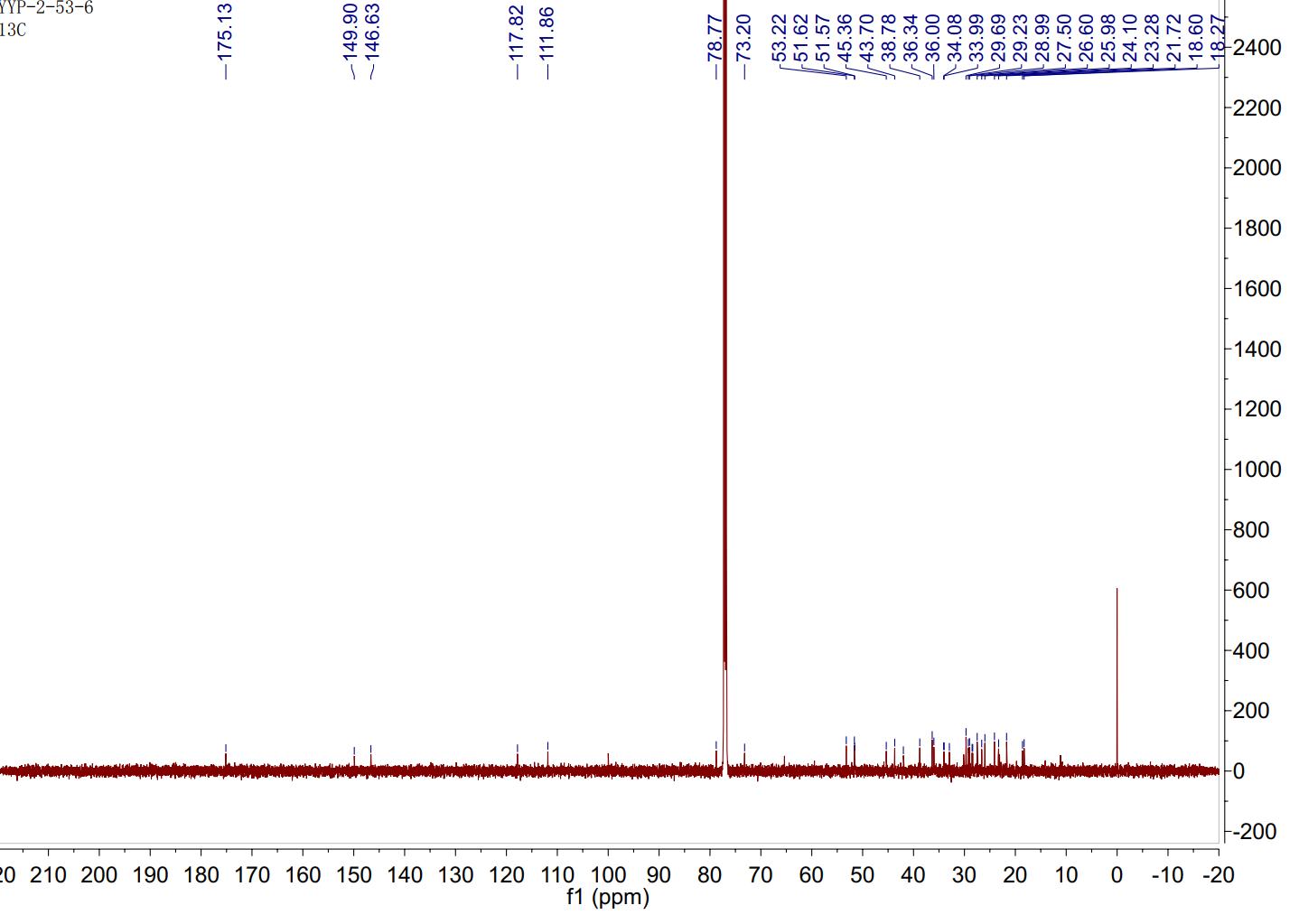


**Figure S52.** ^13^C NMR (150 MHz, CDCl_3_) spectrum of Heilaohumethylester B (**8**)


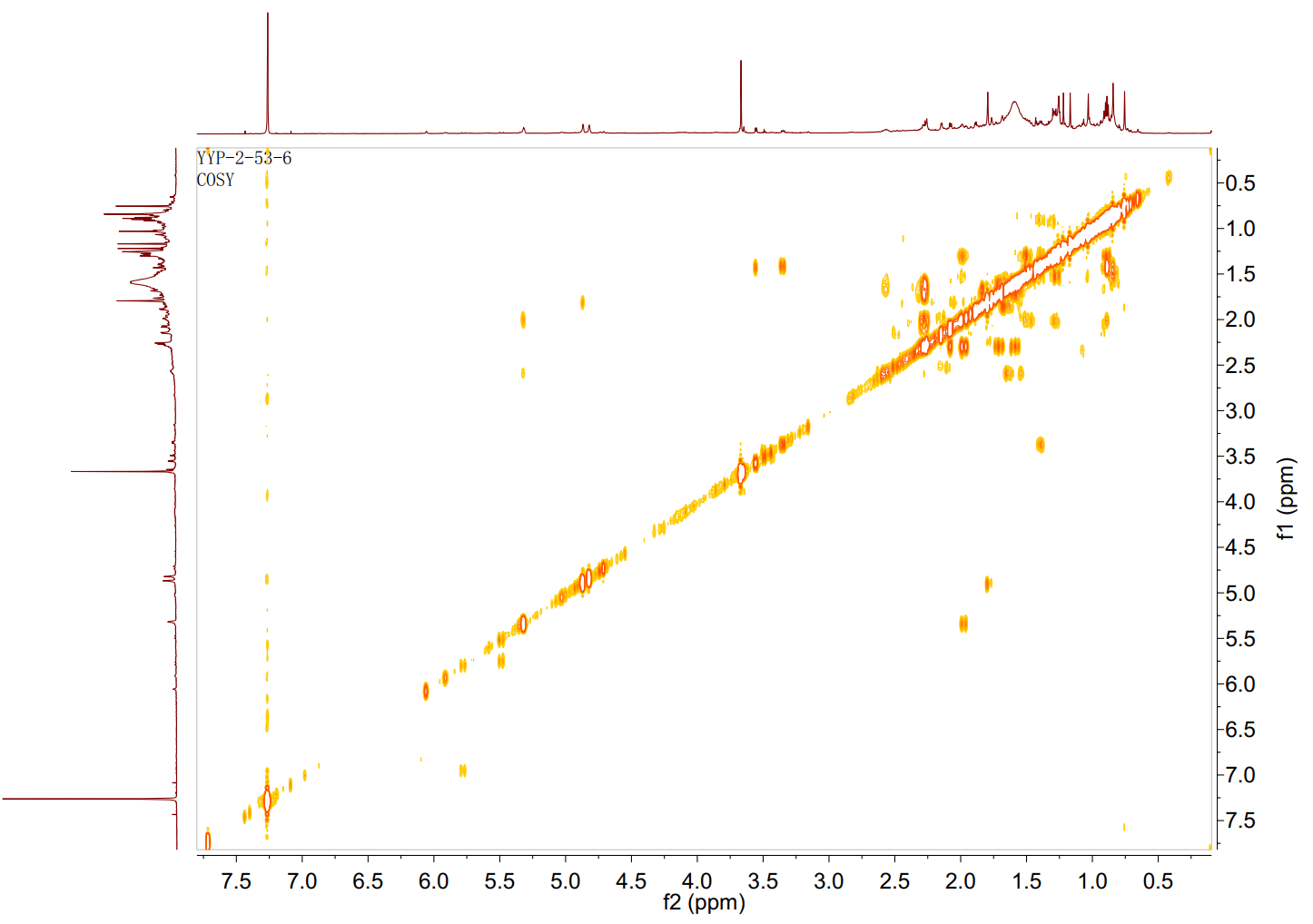


**Figure S53.** COSY NMR spectrum (CDCl_3_) of Heilaohumethylester B (**8**)


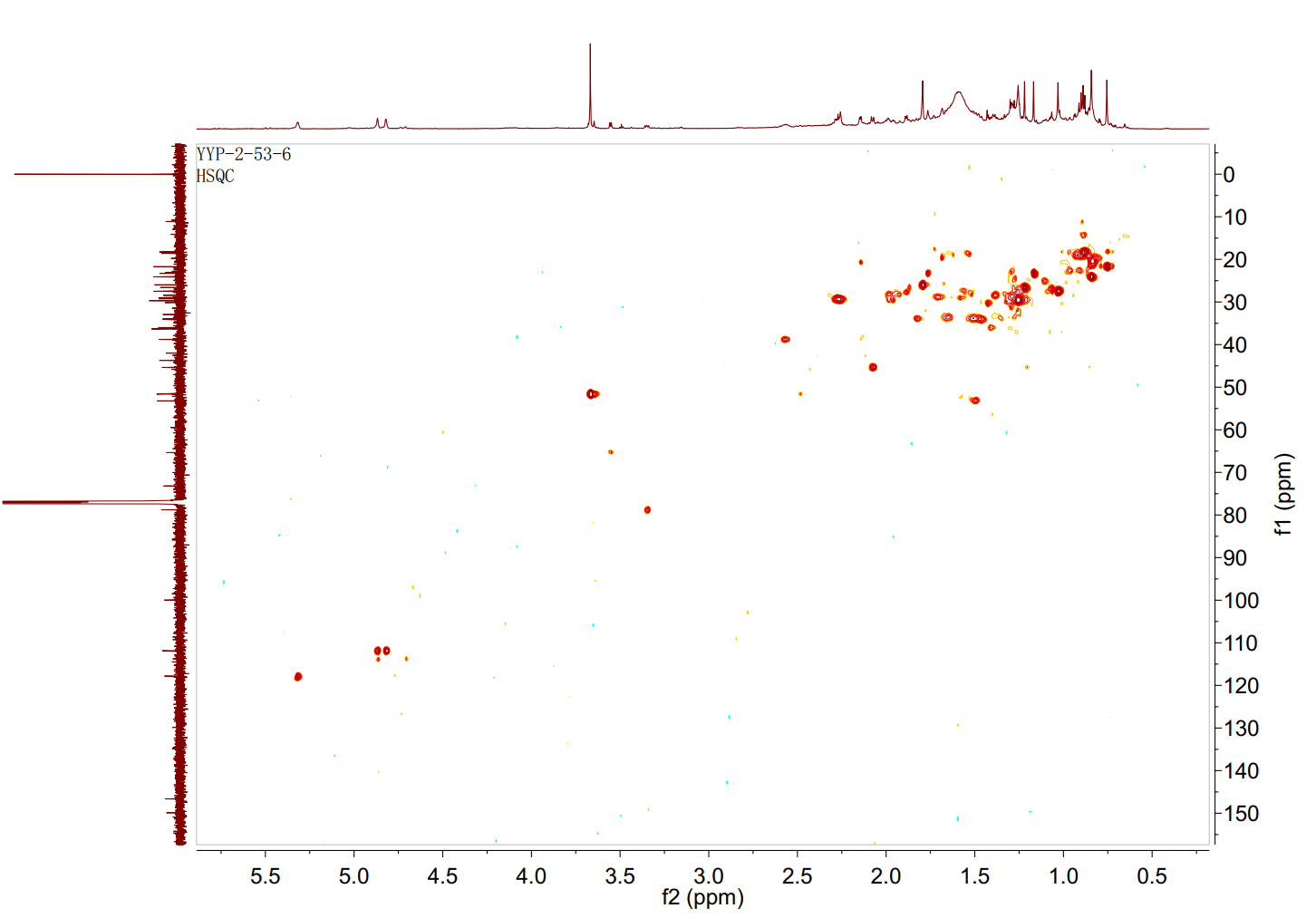


**Figure S54.** HSQC NMR spectrum (CDCl_3_) of Heilaohumethylester B (**8**)


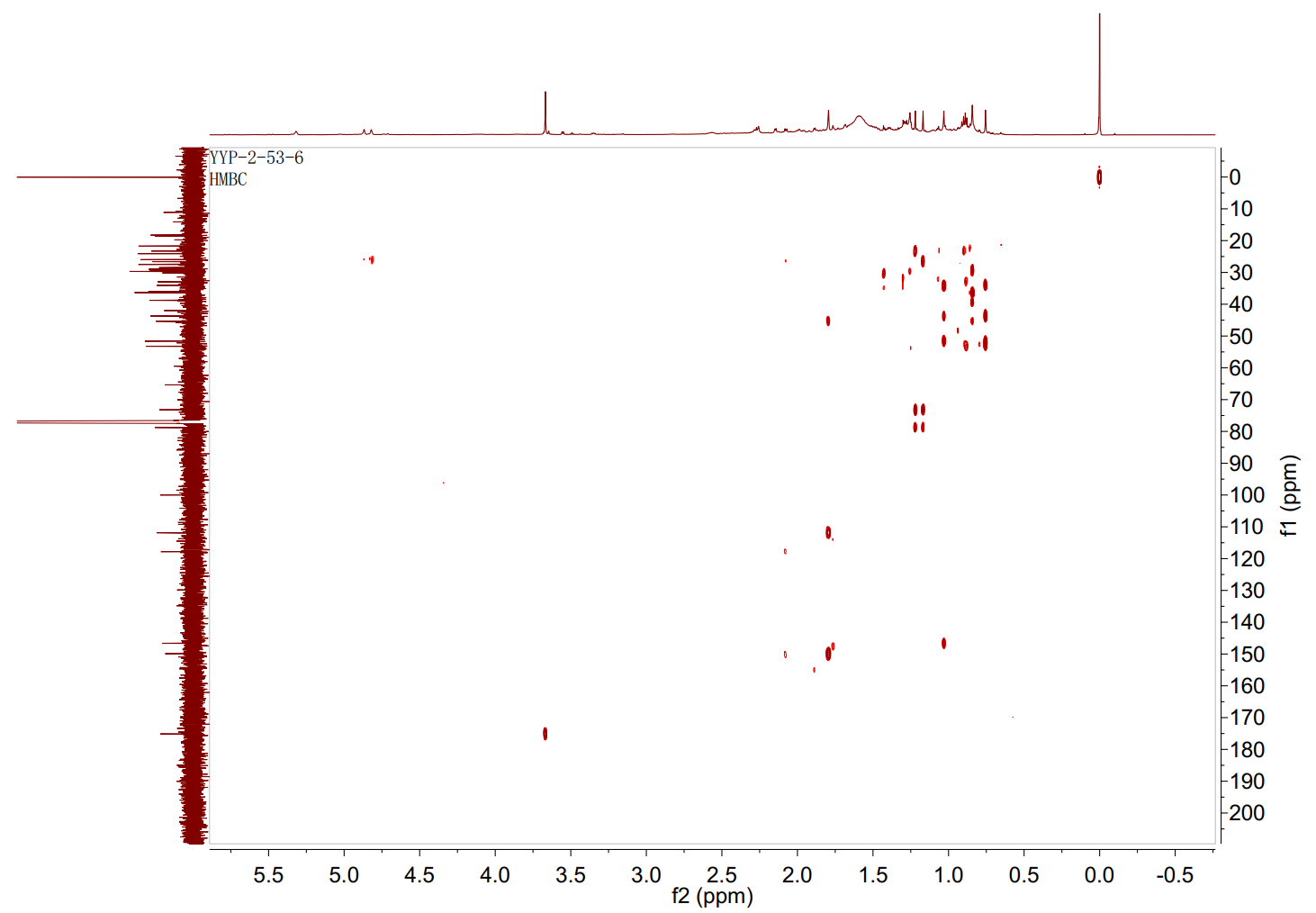


**Figure S55.** HMBC NMR spectrum (CDCl_3_) of Heilaohumethylester B (**8**)


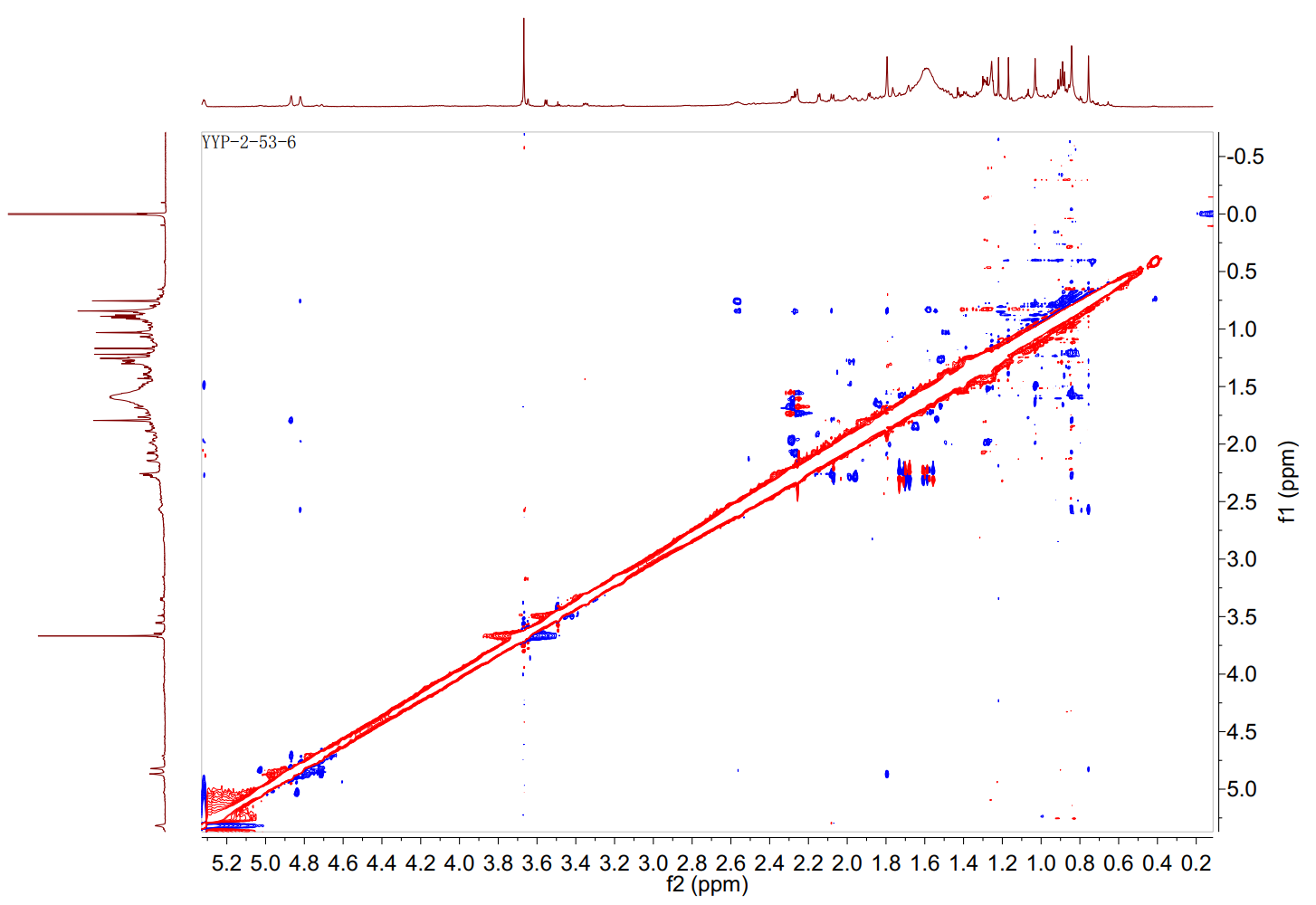


**Figure S56.** ROESY spectrum (CDCl_3_) of Heilaohumethylester B (**8**)

**Figure S57.** HRESIMS spectrum of Heilaohumethylester B (**8**)


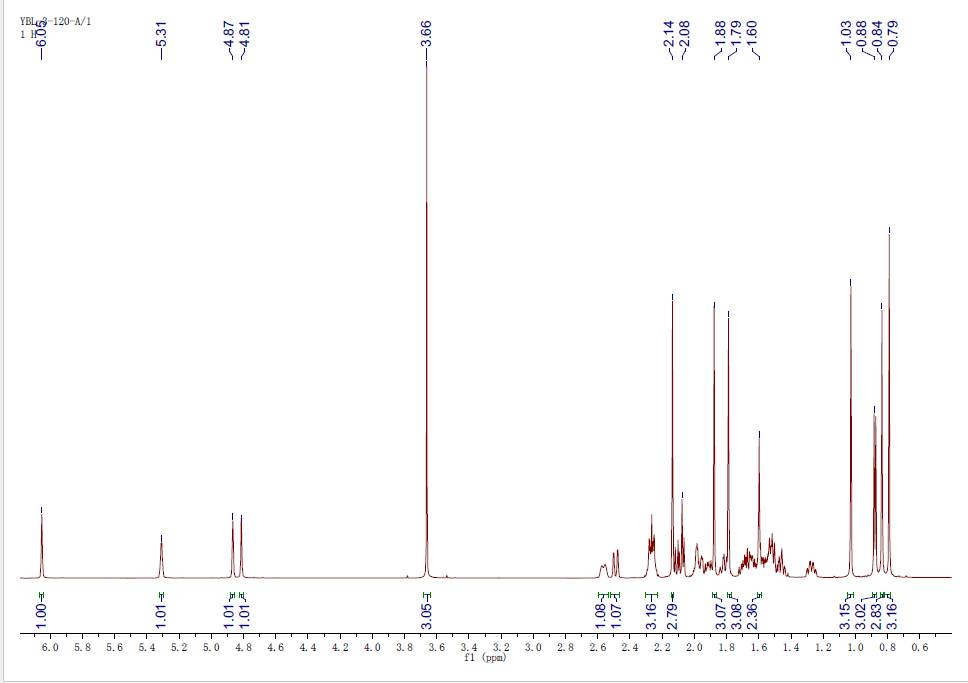
**Figure S58.** ^1^H NMR (600 MHz, CDCl_3_) spectrum of Heilaohumethylester C (**9**)


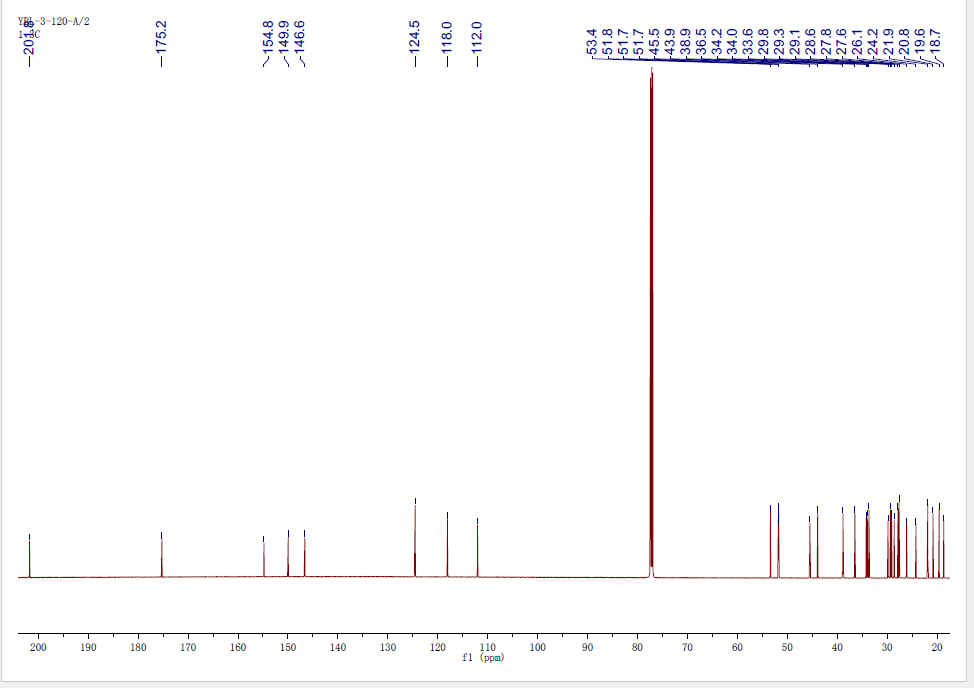


**Figure S59.** ^13^C NMR (150 MHz, CDCl_3_) spectrum of Heilaohumethylester C (**9**)


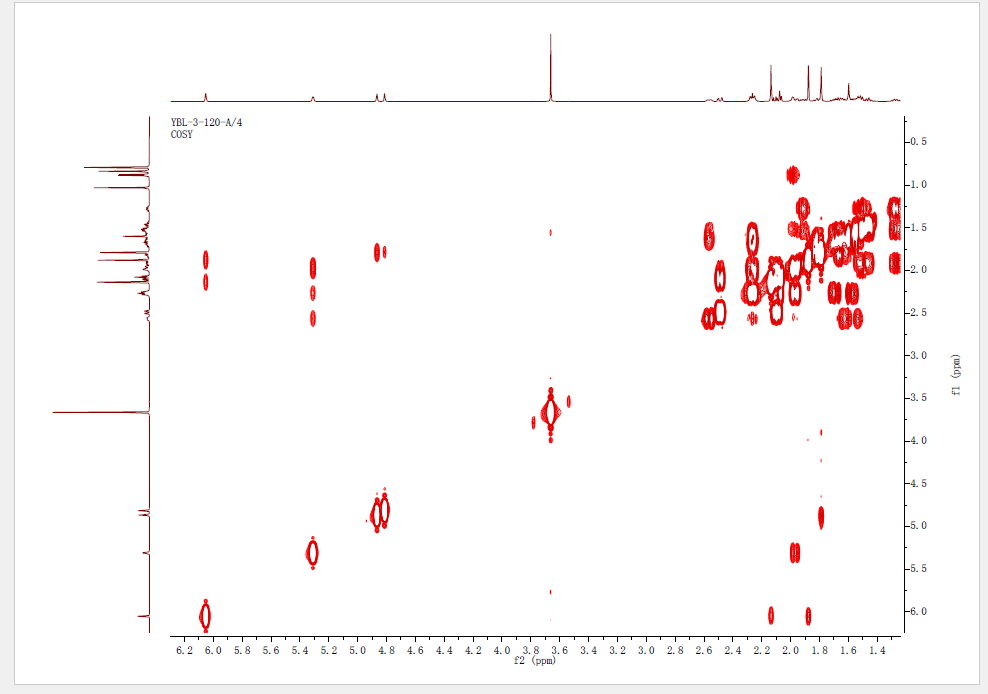


**Figure S60.** COSY NMR spectrum (CDCl_3_) of Heilaohumethylester C (**9**)


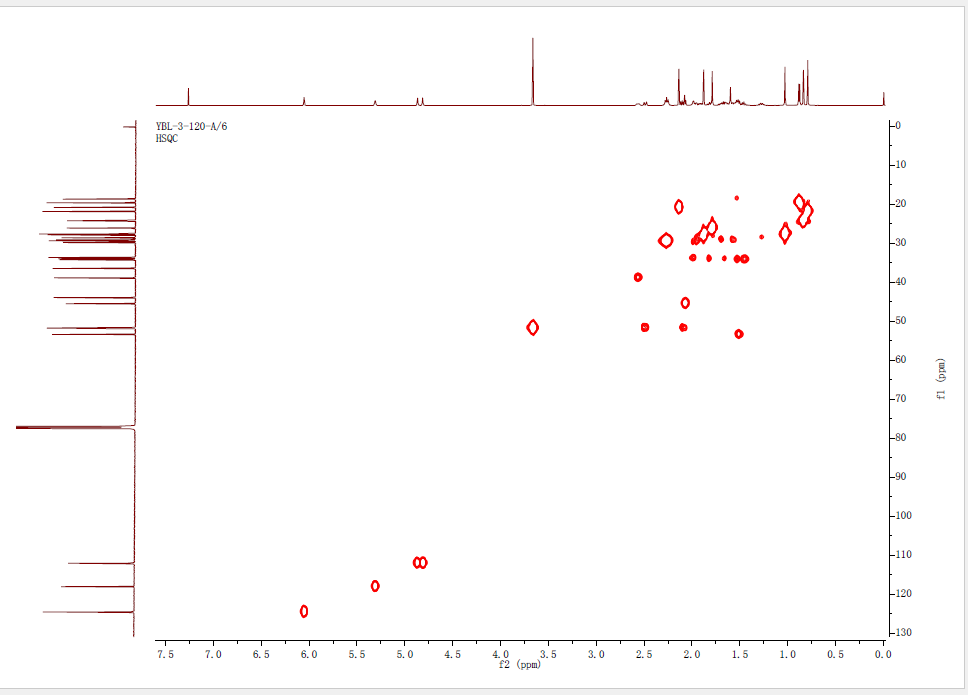
**Figure S61.** HSQC NMR spectrum (CDCl_3_) of Heilaohumethylester C (**9**)


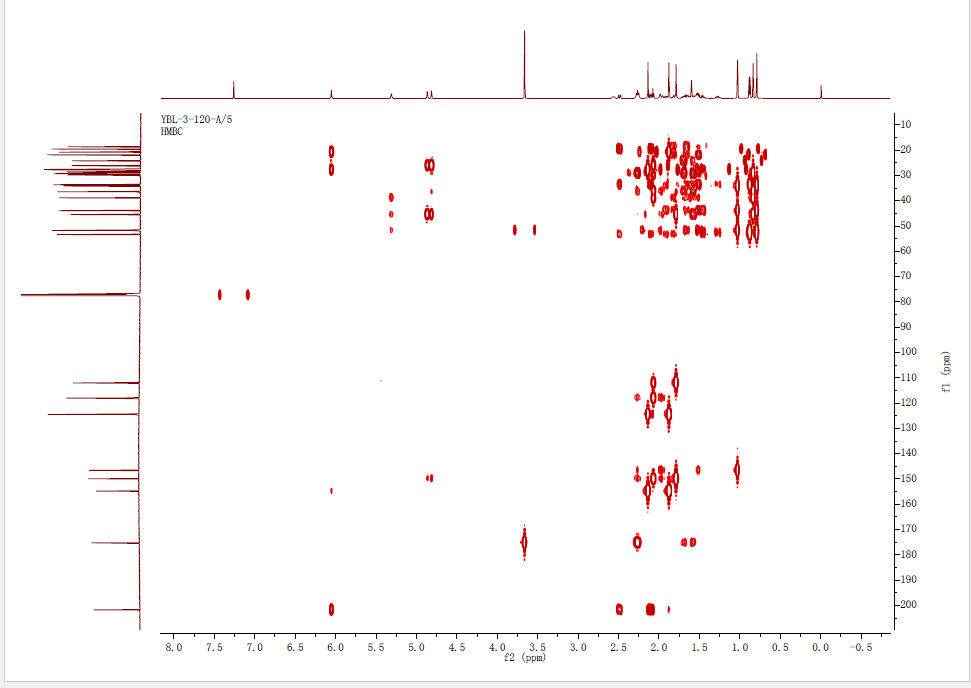


**Figure S62.** HMBC NMR spectrum (CDCl_3_) of Heilaohumethylester C (**9**)


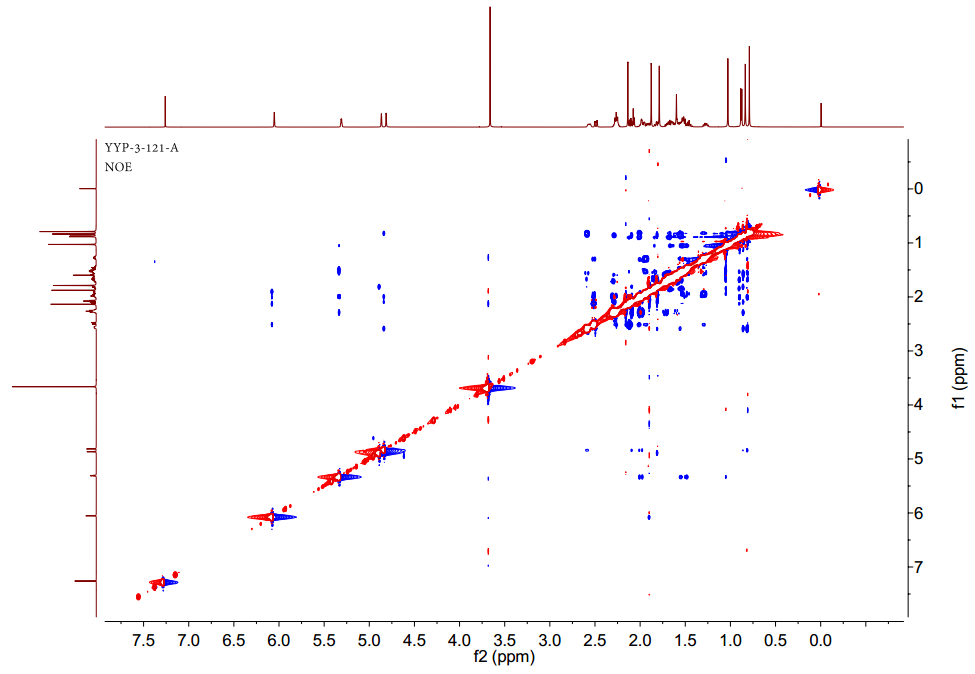


**Figure S63.** ROESY spectrum (CDCl_3_) of Heilaohumethylester C (**9**)


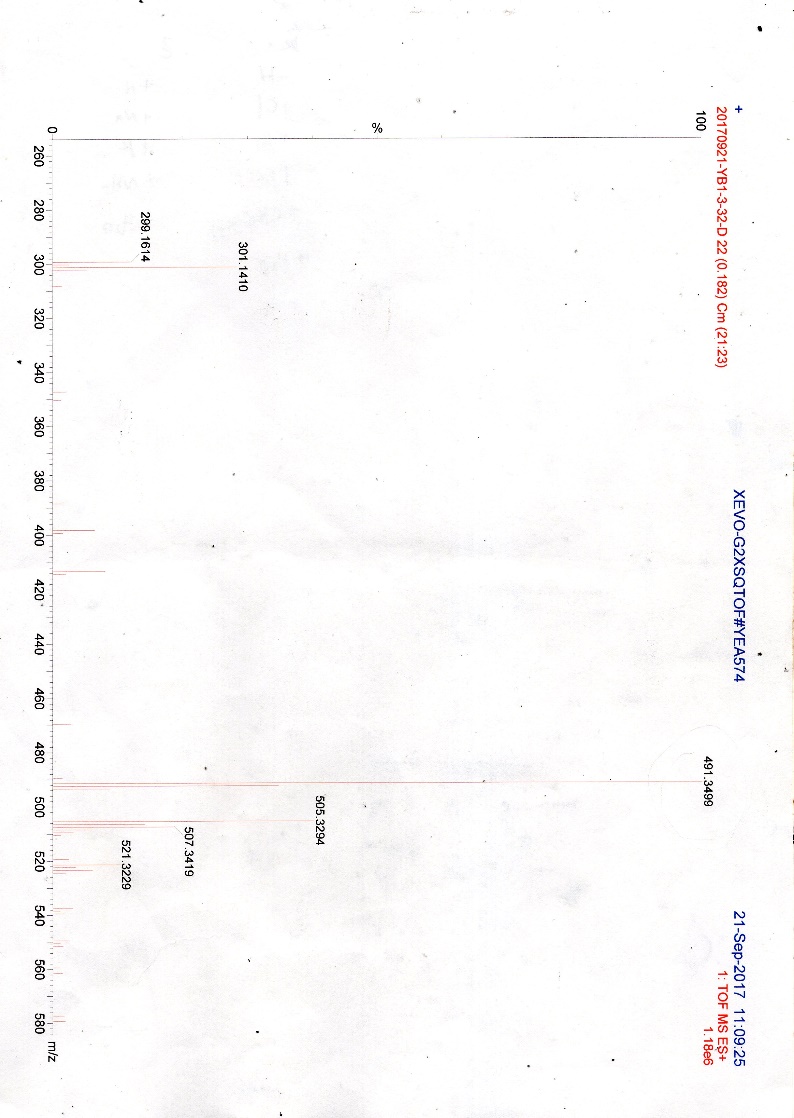


**Figure S64.** HRESIMS spectrum of Heilaohumethylester C (**9**)

2. ECD Results
Table S1.Gibbs free energies^a^ and equilibrium populations^b^ of low-energy conformers of compound **1**

| Conformers | ∆G(a.u.) | P(%)/100 | Single point energy(a.u.) |
| --- | --- | --- | --- |
| C1000001_tddft_ | 0.00000 | 78.3 | -1469.93394 |
| C1000002_tddft_ | 0.01681 | 0.0 | -1469.917126 |
| C1000004_tddft_ | 0.00693 | 0.05 | -1469.927013 |
| C1000005_tddft_ | 0.00895 | 0.01 | -1469.92499 |
| C1000006_tddft_ | 0.01528 | 0.0 | -1469.918664 |
| C1000009_tddft_ | 0.00121 | 21.64 | -1469.932726 |

^a^PBE0/def2-TZVP, in kcal/mol.
^b^From ∆G values at 298.15K.

Table S2.Cartesian coordinates for the low-energy reoptimized random reseach conformers of compound **1** at PBE0-D3(BJ)/def2-SVP level of theory in methanol.

| C1000001_en_ | | Standard Orientation (A.U.) | | | |
| --- | --- | --- | --- | --- | --- |
| Center number | Atomic number | Atomic Type | X | Y | Z |
| 0 | 6 | 0 | -5.439024 | 2.540772 | -5.162925 |
| 1 | 6 | 0 | -7.845054 | -4.389551 | 1.263806 |
| 2 | 6 | 0 | -6.910708 | -3.554296 | -1.325694 |
| 3 | 6 | 0 | -5.617406 | -0.87441 | -1.384865 |
| 4 | 6 | 0 | -5.233582 | -0.233104 | -4.241941 |
| 5 | 6 | 0 | -5.180238 | -5.535054 | -2.573099 |
| 6 | 6 | 0 | -2.442478 | -5.247503 | -1.92582 |
| 7 | 6 | 0 | -1.458047 | -3.243535 | -0.749687 |
| 8 | 6 | 0 | -3.113249 | -1.086207 | 0.129179 |
| 9 | 6 | 0 | 1.382671 | -3.080778 | -0.261759 |
| 10 | 6 | 0 | 2.054546 | -1.18157 | 1.792455 |
| 11 | 6 | 0 | 0.671665 | 0.902542 | 2.065684 |
| 12 | 6 | 0 | -1.551643 | 1.354264 | 0.355281 |
| 13 | 6 | 0 | 2.54708 | -5.538481 | 0.79407 |
| 14 | 6 | 0 | 4.985212 | -4.6467 | 2.06064 |
| 15 | 6 | 0 | 4.32106 | -2.092301 | 3.355734 |
| 16 | 6 | 0 | -7.36345 | 1.04999 | -0.109899 |
| 17 | 6 | 0 | -10.633331 | -4.067008 | 1.7279 |
| 18 | 6 | 0 | -6.364647 | -5.470157 | 2.997593 |
| 19 | 6 | 0 | 6.609671 | -0.23499 | 3.213976 |
| 20 | 6 | 0 | 9.126801 | -1.47348 | 3.951572 |
| 21 | 6 | 0 | 6.310013 | 2.212056 | 4.777575 |
| 22 | 6 | 0 | 7.415389 | 4.550958 | 3.40885 |
| 23 | 6 | 0 | 5.632254 | 5.349705 | 1.380366 |
| 24 | 6 | 0 | 5.828026 | 4.991159 | -1.122504 |
| 25 | 6 | 0 | 8.057704 | 3.884681 | -2.483417 |
| 26 | 1 | 0 | -3.692166 | -1.535692 | 2.081545 |
| 27 | 6 | 0 | 2.712893 | -2.323537 | -2.757083 |
| 28 | 6 | 0 | 3.495037 | -2.583414 | 6.097923 |
| 29 | 6 | 0 | -3.017755 | 4.002456 | -4.923804 |
| 30 | 8 | 0 | -1.130261 | 3.419381 | -6.148766 |
| 31 | 8 | 0 | -3.089509 | 5.910211 | -3.306279 |
| 32 | 6 | 0 | 3.515067 | 5.50336 | -2.624461 |
| 33 | 8 | 0 | 1.659472 | 6.662435 | -1.821118 |
| 34 | 8 | 0 | 3.598946 | 4.477604 | -4.917443 |
| 35 | 1 | 0 | -5.841357 | 2.464864 | -7.193392 |
| 36 | 1 | 0 | -6.984428 | 3.554424 | -4.246997 |
| 37 | 1 | 0 | -8.611154 | -3.373197 | -2.508975 |
| 38 | 1 | 0 | -6.702174 | -1.252042 | -5.290842 |
| 39 | 1 | 0 | -3.424837 | -1.005157 | -4.895307 |
| 40 | 1 | 0 | -5.381269 | -5.438413 | -4.64274 |
| 41 | 1 | 0 | -5.835824 | -7.431352 | -2.042599 |
| 42 | 1 | 0 | -1.20534 | -6.780122 | -2.530129 |
| 43 | 1 | 0 | 1.048524 | 2.285686 | 3.532745 |
| 44 | 1 | 0 | -2.70166 | 2.910109 | 1.073776 |
| 45 | 1 | 0 | -0.870208 | 1.92101 | -1.515061 |
| 46 | 1 | 0 | 2.891164 | -6.965075 | -0.666279 |
| 47 | 1 | 0 | 1.246806 | -6.360663 | 2.183771 |
| 48 | 1 | 0 | 5.751512 | -6.021671 | 3.40512 |
| 49 | 1 | 0 | 6.430438 | -4.352696 | 0.606167 |
| 50 | 1 | 0 | -9.217365 | 1.095016 | -1.036417 |
| 51 | 1 | 0 | -7.647606 | 0.555573 | 1.879132 |
| 52 | 1 | 0 | -6.585773 | 2.962909 | -0.175913 |
| 53 | 1 | 0 | -11.243834 | -2.111317 | 1.430039 |
| 54 | 1 | 0 | -11.713813 | -5.230008 | 0.390718 |
| 55 | 1 | 0 | -11.15613 | -4.634071 | 3.644787 |
| 56 | 1 | 0 | -7.130139 | -6.070503 | 4.806241 |
| 57 | 1 | 0 | -4.371965 | -5.820784 | 2.652948 |
| 58 | 1 | 0 | 6.731733 | 0.316173 | 1.214371 |
| 59 | 1 | 0 | 9.661867 | -3.022646 | 2.695542 |
| 60 | 1 | 0 | 9.057401 | -2.210205 | 5.886302 |
| 61 | 1 | 0 | 10.651432 | -0.072324 | 3.887255 |
| 62 | 1 | 0 | 4.329756 | 2.606138 | 5.207386 |
| 63 | 1 | 0 | 7.258642 | 1.983343 | 6.608358 |
| 64 | 1 | 0 | 7.619689 | 6.096026 | 4.778386 |
| 65 | 1 | 0 | 9.30503 | 4.121235 | 2.682302 |
| 66 | 1 | 0 | 3.852183 | 6.146824 | 2.040197 |
| 67 | 1 | 0 | 9.66299 | 3.618364 | -1.216008 |
| 68 | 1 | 0 | 7.574779 | 2.058597 | -3.329776 |
| 69 | 1 | 0 | 8.650637 | 5.114184 | -4.039066 |
| 70 | 1 | 0 | 4.756524 | -2.126979 | -2.493193 |
| 71 | 1 | 0 | 2.369998 | -3.764447 | -4.204048 |
| 72 | 1 | 0 | 2.000178 | -0.520038 | -3.472866 |
| 73 | 1 | 0 | 1.996702 | -4.009599 | 6.14853 |
| 74 | 1 | 0 | 5.07486 | -3.27725 | 7.245049 |
| 75 | 1 | 0 | 2.741835 | -0.875525 | 6.986167 |
| 76 | 1 | 0 | -1.312548 | 6.479895 | -2.968472 |
| 77 | 1 | 0 | 1.842568 | 4.422707 | -5.609615 |

| C1000002_en_ | | Standard Orientation (A.U.) | | | |
| --- | --- | --- | --- | --- | --- |
| Center number | Atomic number | Atomic Type | X | Y | Z |
| 0 | 6 | 0 | -4.525515 | 2.404522 | -4.403897 |
| 1 | 6 | 0 | -8.761063 | -3.885198 | 1.887256 |
| 2 | 6 | 0 | -7.577737 | -3.299611 | -0.669366 |
| 3 | 6 | 0 | -6.145868 | -0.693034 | -0.784648 |
| 4 | 6 | 0 | -5.484586 | -0.258478 | -3.61375 |
| 5 | 6 | 0 | -5.864427 | -5.465013 | -1.600206 |
| 6 | 6 | 0 | -3.139772 | -5.189233 | -0.893444 |
| 7 | 6 | 0 | -2.126185 | -3.130413 | 0.161682 |
| 8 | 6 | 0 | -3.769941 | -0.913619 | 0.918773 |
| 9 | 6 | 0 | 0.723333 | -2.949932 | 0.545396 |
| 10 | 6 | 0 | 1.435438 | -1.024524 | 2.559404 |
| 11 | 6 | 0 | 0.013579 | 1.010796 | 2.961439 |
| 12 | 6 | 0 | -2.236363 | 1.532326 | 1.313254 |
| 13 | 6 | 0 | 1.99175 | -5.380093 | 1.557518 |
| 14 | 6 | 0 | 4.442623 | -4.429064 | 2.772629 |
| 15 | 6 | 0 | 3.749028 | -1.880402 | 4.059837 |
| 16 | 6 | 0 | -7.909591 | 1.392089 | 0.172975 |
| 17 | 6 | 0 | -11.551891 | -3.388857 | 2.113537 |
| 18 | 6 | 0 | -7.468609 | -4.920437 | 3.790675 |
| 19 | 6 | 0 | 6.086 | -0.058382 | 4.067391 |
| 20 | 6 | 0 | 5.922641 | 2.127612 | 5.961815 |
| 21 | 6 | 0 | 6.90597 | 0.950932 | 1.449014 |
| 22 | 6 | 0 | 5.548835 | 3.362118 | 0.514952 |
| 23 | 6 | 0 | 5.687797 | 3.695043 | -2.283227 |
| 24 | 6 | 0 | 6.758486 | 5.579999 | -3.595767 |
| 25 | 6 | 0 | 8.274029 | 7.714278 | -2.507501 |
| 26 | 1 | 0 | -4.506513 | -1.358562 | 2.817285 |
| 27 | 6 | 0 | 1.895525 | -2.242385 | -2.038482 |
| 28 | 6 | 0 | 2.918171 | -2.44776 | 6.792599 |
| 29 | 6 | 0 | -1.889137 | 2.456065 | -5.509322 |
| 30 | 8 | 0 | -0.22072 | 3.85556 | -4.801652 |
| 31 | 8 | 0 | -1.417731 | 0.840666 | -7.433799 |
| 32 | 6 | 0 | 6.649905 | 5.562042 | -6.427245 |
| 33 | 8 | 0 | 8.323458 | 6.438908 | -7.718193 |
| 34 | 8 | 0 | 4.596411 | 4.516502 | -7.535805 |
| 35 | 1 | 0 | -5.777299 | 3.193196 | -5.861466 |
| 36 | 1 | 0 | -4.539402 | 3.753443 | -2.849964 |
| 37 | 1 | 0 | -9.160718 | -3.128591 | -2.007281 |
| 38 | 1 | 0 | -7.19305 | -0.689724 | -4.707784 |
| 39 | 1 | 0 | -4.073228 | -1.684853 | -4.130516 |
| 40 | 1 | 0 | -5.986803 | -5.614198 | -3.674 |
| 41 | 1 | 0 | -6.611037 | -7.264277 | -0.885664 |
| 42 | 1 | 0 | -1.917018 | -6.768317 | -1.398398 |
| 43 | 1 | 0 | 0.466583 | 2.350094 | 4.453209 |
| 44 | 1 | 0 | -3.437733 | 2.968908 | 2.19141 |
| 45 | 1 | 0 | -1.5785 | 2.329058 | -0.483434 |
| 46 | 1 | 0 | 2.337019 | -6.78287 | 0.074577 |
| 47 | 1 | 0 | 0.756913 | -6.252792 | 2.97478 |
| 48 | 1 | 0 | 5.225899 | -5.771263 | 4.140991 |
| 49 | 1 | 0 | 5.889618 | -4.146924 | 1.318334 |
| 50 | 1 | 0 | -8.377085 | 1.086108 | 2.164841 |
| 51 | 1 | 0 | -7.070686 | 3.272662 | 0.025801 |
| 52 | 1 | 0 | -9.673093 | 1.41241 | -0.915647 |
| 53 | 1 | 0 | -12.037063 | -1.431767 | 1.645069 |
| 54 | 1 | 0 | -12.593203 | -4.58839 | 0.777706 |
| 55 | 1 | 0 | -12.242597 | -3.796657 | 4.01743 |
| 56 | 1 | 0 | -8.385136 | -5.347679 | 5.577608 |
| 57 | 1 | 0 | -5.48285 | -5.412689 | 3.613192 |
| 58 | 1 | 0 | 7.643995 | -1.279859 | 4.716616 |
| 59 | 1 | 0 | 4.333144 | 3.388243 | 5.563058 |
| 60 | 1 | 0 | 7.648703 | 3.26788 | 5.85997 |
| 61 | 1 | 0 | 5.737432 | 1.459657 | 7.906655 |
| 62 | 1 | 0 | 8.938339 | 1.364158 | 1.523611 |
| 63 | 1 | 0 | 6.715844 | -0.535759 | 0.023509 |
| 64 | 1 | 0 | 3.537328 | 3.226253 | 0.992087 |
| 65 | 1 | 0 | 6.283346 | 5.019627 | 1.50711 |
| 66 | 1 | 0 | 4.768322 | 2.1934 | -3.352601 |
| 67 | 1 | 0 | 7.355496 | 9.533174 | -2.883586 |
| 68 | 1 | 0 | 8.546806 | 7.523264 | -0.472615 |
| 69 | 1 | 0 | 10.119569 | 7.785163 | -3.43706 |
| 70 | 1 | 0 | 3.958686 | -2.226887 | -1.958726 |
| 71 | 1 | 0 | 1.316278 | -3.623789 | -3.466207 |
| 72 | 1 | 0 | 1.27597 | -0.371895 | -2.658439 |
| 73 | 1 | 0 | 1.499669 | -3.9543 | 6.803828 |
| 74 | 1 | 0 | 4.531774 | -3.068124 | 7.938177 |
| 75 | 1 | 0 | 2.065273 | -0.794475 | 7.690898 |
| 76 | 1 | 0 | -2.897092 | -0.173247 | -7.789444 |
| 77 | 1 | 0 | 3.232339 | 4.226897 | -6.328766 |

| C1000004_en_ | | Standard Orientation (A.U.) | | | |
| --- | --- | --- | --- | --- | --- |
| Center number | Atomic number | Atomic Type | X | Y | Z |
| 0 | 6 | 0 | -7.181882 | -0.82986 | -6.015576 |
| 1 | 6 | 0 | -7.790983 | -5.25128 | 1.15249 |
| 2 | 6 | 0 | -6.743794 | -4.2628 | -1.340255 |
| 3 | 6 | 0 | -5.955494 | -1.408484 | -1.281467 |
| 4 | 6 | 0 | -5.192197 | -0.509138 | -3.954679 |
| 5 | 6 | 0 | -4.553406 | -5.891431 | -2.347162 |
| 6 | 6 | 0 | -1.99651 | -5.088286 | -1.4381 |
| 7 | 6 | 0 | -1.5287 | -2.932348 | -0.213477 |
| 8 | 6 | 0 | -3.651713 | -1.157029 | 0.512881 |
| 9 | 6 | 0 | 1.167467 | -2.169715 | 0.454505 |
| 10 | 6 | 0 | 1.222779 | -0.293359 | 2.638702 |
| 11 | 6 | 0 | -0.543728 | 1.490352 | 2.820365 |
| 12 | 6 | 0 | -2.688736 | 1.5616 | 0.954116 |
| 13 | 6 | 0 | 2.859484 | -4.349818 | 1.414314 |
| 14 | 6 | 0 | 4.810898 | -3.030463 | 3.096236 |
| 15 | 6 | 0 | 3.323758 | -0.935073 | 4.509006 |
| 16 | 6 | 0 | -8.147154 | 0.214374 | -0.296662 |
| 17 | 6 | 0 | -10.62598 | -5.362874 | 1.356619 |
| 18 | 6 | 0 | -6.329579 | -6.109473 | 3.02143 |
| 19 | 6 | 0 | 5.130995 | 1.271447 | 5.255818 |
| 20 | 6 | 0 | 4.185105 | 2.957238 | 7.413048 |
| 21 | 6 | 0 | 5.866607 | 2.907766 | 2.971991 |
| 22 | 6 | 0 | 8.48479 | 4.154879 | 3.241772 |
| 23 | 6 | 0 | 9.035415 | 5.847443 | 1.048409 |
| 24 | 6 | 0 | 9.991862 | 5.14451 | -1.191365 |
| 25 | 6 | 0 | 10.72283 | 2.473403 | -1.845151 |
| 26 | 1 | 0 | -4.323394 | -1.780532 | 2.385471 |
| 27 | 6 | 0 | 2.378705 | -0.983864 | -1.93487 |
| 28 | 6 | 0 | 2.109281 | -2.113665 | 6.877879 |
| 29 | 6 | 0 | -6.307313 | 0.247938 | -8.527604 |
| 30 | 8 | 0 | -4.288025 | 1.210917 | -8.907557 |
| 31 | 8 | 0 | -8.014046 | 0.055308 | -10.454837 |
| 32 | 6 | 0 | 10.297288 | 7.186485 | -3.130975 |
| 33 | 8 | 0 | 9.822854 | 9.389262 | -2.786261 |
| 34 | 8 | 0 | 11.18341 | 6.448211 | -5.439209 |
| 35 | 1 | 0 | -7.644176 | -2.831011 | -6.330224 |
| 36 | 1 | 0 | -8.964647 | 0.107977 | -5.504247 |
| 37 | 1 | 0 | -8.315611 | -4.410913 | -2.691462 |
| 38 | 1 | 0 | -3.458263 | -1.466177 | -4.550239 |
| 39 | 1 | 0 | -4.718022 | 1.504077 | -3.859814 |
| 40 | 1 | 0 | -4.543395 | -5.842257 | -4.430094 |
| 41 | 1 | 0 | -4.898557 | -7.875276 | -1.845379 |
| 42 | 1 | 0 | -0.432479 | -6.33909 | -1.922245 |
| 43 | 1 | 0 | -0.561906 | 2.832195 | 4.375996 |
| 44 | 1 | 0 | -4.21911 | 2.73289 | 1.701809 |
| 45 | 1 | 0 | -2.093956 | 2.439214 | -0.831012 |
| 46 | 1 | 0 | 3.728259 | -5.420871 | -0.129457 |
| 47 | 1 | 0 | 1.718001 | -5.675387 | 2.52376 |
| 48 | 1 | 0 | 5.737893 | -4.323532 | 4.422556 |
| 49 | 1 | 0 | 6.304478 | -2.188043 | 1.928743 |
| 50 | 1 | 0 | -8.479396 | -0.128541 | 1.717823 |
| 51 | 1 | 0 | -7.753266 | 2.229502 | -0.548484 |
| 52 | 1 | 0 | -9.908394 | -0.199423 | -1.302627 |
| 53 | 1 | 0 | -11.500992 | -3.512868 | 1.045074 |
| 54 | 1 | 0 | -11.394826 | -6.631208 | -0.0953 |
| 55 | 1 | 0 | -11.225197 | -6.063377 | 3.205613 |
| 56 | 1 | 0 | -7.157125 | -6.836771 | 4.754296 |
| 57 | 1 | 0 | -4.282778 | -6.146832 | 2.866214 |
| 58 | 1 | 0 | 6.86098 | 0.327129 | 5.937721 |
| 59 | 1 | 0 | 3.933575 | 1.910625 | 9.175481 |
| 60 | 1 | 0 | 2.3863 | 3.860874 | 6.938828 |
| 61 | 1 | 0 | 5.546745 | 4.468475 | 7.790188 |
| 62 | 1 | 0 | 5.886416 | 1.777566 | 1.239066 |
| 63 | 1 | 0 | 4.423031 | 4.36527 | 2.667063 |
| 64 | 1 | 0 | 8.564446 | 5.266045 | 4.987739 |
| 65 | 1 | 0 | 9.91677 | 2.665146 | 3.421046 |
| 66 | 1 | 0 | 8.538482 | 7.835541 | 1.235175 |
| 67 | 1 | 0 | 10.465646 | 1.20625 | -0.237302 |
| 68 | 1 | 0 | 9.552677 | 1.717034 | -3.385888 |
| 69 | 1 | 0 | 12.714078 | 2.328242 | -2.414372 |
| 70 | 1 | 0 | 1.390387 | 0.736113 | -2.512227 |
| 71 | 1 | 0 | 4.3625 | -0.495132 | -1.607379 |
| 72 | 1 | 0 | 2.286902 | -2.336609 | -3.498337 |
| 73 | 1 | 0 | 1.049166 | -3.818638 | 6.377757 |
| 74 | 1 | 0 | 3.56188 | -2.637866 | 8.262289 |
| 75 | 1 | 0 | 0.78031 | -0.806011 | 7.767633 |
| 76 | 1 | 0 | -9.539376 | -0.752154 | -9.857002 |
| 77 | 1 | 0 | 11.460297 | 4.64508 | -5.4744 |

| C1000005_en_ | | Standard Orientation (A.U.) | | | |
| --- | --- | --- | --- | --- | --- |
| Center number | Atomic number | Atomic Type | X | Y | Z |
| 0 | 6 | 0 | -5.708942 | 0.187461 | -6.977665 |
| 1 | 6 | 0 | -7.429592 | -4.530314 | -0.249671 |
| 2 | 6 | 0 | -6.051811 | -3.340841 | -2.480504 |
| 3 | 6 | 0 | -5.291932 | -0.515919 | -2.045394 |
| 4 | 6 | 0 | -4.213851 | 0.625847 | -4.517924 |
| 5 | 6 | 0 | -3.738193 | -4.894162 | -3.313021 |
| 6 | 6 | 0 | -1.322837 | -4.188499 | -2.021669 |
| 7 | 6 | 0 | -1.017766 | -2.171498 | -0.535832 |
| 8 | 6 | 0 | -3.21766 | -0.428203 | 0.031235 |
| 9 | 6 | 0 | 1.585051 | -1.505138 | 0.554924 |
| 10 | 6 | 0 | 1.320014 | 0.244194 | 2.826743 |
| 11 | 6 | 0 | -0.444955 | 2.035282 | 2.882673 |
| 12 | 6 | 0 | -2.318039 | 2.252897 | 0.760631 |
| 13 | 6 | 0 | 3.086243 | -3.796414 | 1.636262 |
| 14 | 6 | 0 | 3.044967 | -3.454773 | 4.515008 |
| 15 | 6 | 0 | 3.053802 | -0.561765 | 4.967486 |
| 16 | 6 | 0 | -7.599052 | 1.006668 | -1.162605 |
| 17 | 6 | 0 | -10.259511 | -4.674391 | -0.482915 |
| 18 | 6 | 0 | -6.236349 | -5.522346 | 1.740054 |
| 19 | 6 | 0 | 5.791174 | 0.513676 | 4.622502 |
| 20 | 6 | 0 | 7.549973 | -0.211371 | 6.807854 |
| 21 | 6 | 0 | 5.802082 | 3.38432 | 4.205114 |
| 22 | 6 | 0 | 8.43406 | 4.486945 | 3.585095 |
| 23 | 6 | 0 | 9.605428 | 3.176117 | 1.372269 |
| 24 | 6 | 0 | 9.174166 | 3.669854 | -1.076543 |
| 25 | 6 | 0 | 7.473074 | 5.747899 | -2.01286 |
| 26 | 1 | 0 | -4.12562 | -1.162722 | 1.757522 |
| 27 | 6 | 0 | 3.108164 | -0.200536 | -1.575903 |
| 28 | 6 | 0 | 1.984593 | 0.098793 | 7.571329 |
| 29 | 6 | 0 | -8.373037 | 1.233444 | -7.035413 |
| 30 | 8 | 0 | -10.250882 | -0.017346 | -7.318939 |
| 31 | 8 | 0 | -8.576711 | 3.791643 | -6.779069 |
| 32 | 6 | 0 | 10.442557 | 1.961858 | -2.947606 |
| 33 | 8 | 0 | 11.81927 | 0.228138 | -2.402664 |
| 34 | 8 | 0 | 9.951713 | 2.429649 | -5.436754 |
| 35 | 1 | 0 | -4.654462 | 1.070493 | -8.535438 |
| 36 | 1 | 0 | -5.848892 | -1.821559 | -7.422538 |
| 37 | 1 | 0 | -7.420888 | -3.343477 | -4.040501 |
| 38 | 1 | 0 | -2.309863 | -0.13148 | -4.828913 |
| 39 | 1 | 0 | -3.963777 | 2.667848 | -4.220141 |
| 40 | 1 | 0 | -3.454774 | -4.705699 | -5.367563 |
| 41 | 1 | 0 | -4.13422 | -6.908111 | -3.005566 |
| 42 | 1 | 0 | 0.288672 | -5.40681 | -2.42334 |
| 43 | 1 | 0 | -0.665903 | 3.249492 | 4.527815 |
| 44 | 1 | 0 | -3.932784 | 3.38837 | 1.3727 |
| 45 | 1 | 0 | -1.49439 | 3.230649 | -0.876895 |
| 46 | 1 | 0 | 5.030798 | -3.766335 | 0.922516 |
| 47 | 1 | 0 | 2.266883 | -5.606644 | 1.077747 |
| 48 | 1 | 0 | 1.276287 | -4.22305 | 5.282139 |
| 49 | 1 | 0 | 4.602632 | -4.435925 | 5.459755 |
| 50 | 1 | 0 | -7.201348 | 3.036063 | -1.139177 |
| 51 | 1 | 0 | -9.230431 | 0.717804 | -2.397455 |
| 52 | 1 | 0 | -8.151634 | 0.441652 | 0.750878 |
| 53 | 1 | 0 | -11.106852 | -2.81871 | -0.829941 |
| 54 | 1 | 0 | -10.775219 | -5.855178 | -2.109211 |
| 55 | 1 | 0 | -11.120405 | -5.487687 | 1.210004 |
| 56 | 1 | 0 | -7.2926 | -6.392535 | 3.271023 |
| 57 | 1 | 0 | -4.188209 | -5.540021 | 1.881078 |
| 58 | 1 | 0 | 6.553939 | -0.355276 | 2.896931 |
| 59 | 1 | 0 | 7.171947 | 0.937665 | 8.489108 |
| 60 | 1 | 0 | 9.531391 | 0.075802 | 6.288296 |
| 61 | 1 | 0 | 7.350605 | -2.198701 | 7.338128 |
| 62 | 1 | 0 | 4.515988 | 3.840663 | 2.647042 |
| 63 | 1 | 0 | 5.06866 | 4.34783 | 5.891692 |
| 64 | 1 | 0 | 8.227293 | 6.516789 | 3.224852 |
| 65 | 1 | 0 | 9.681582 | 4.305847 | 5.22735 |
| 66 | 1 | 0 | 10.860207 | 1.590751 | 1.756363 |
| 67 | 1 | 0 | 5.839641 | 4.987618 | -3.045904 |
| 68 | 1 | 0 | 6.714377 | 6.860862 | -0.451264 |
| 69 | 1 | 0 | 8.478162 | 7.067355 | -3.262093 |
| 70 | 1 | 0 | 3.427861 | -1.534853 | -3.126334 |
| 71 | 1 | 0 | 2.078453 | 1.427129 | -2.332913 |
| 72 | 1 | 0 | 4.945511 | 0.453733 | -0.882154 |
| 73 | 1 | 0 | 0.02334 | -0.541936 | 7.713368 |
| 74 | 1 | 0 | 3.070803 | -0.822057 | 9.071032 |
| 75 | 1 | 0 | 2.009977 | 2.135695 | 7.937781 |
| 76 | 1 | 0 | -6.919915 | 4.521952 | -6.534227 |
| 77 | 1 | 0 | 8.828123 | 3.856936 | -5.610231 |

| C1000006_en_ | | Standard Orientation (A.U.) | | | |
| --- | --- | --- | --- | --- | --- |
| Center number | Atomic number | Atomic Type | X | Y | Z |
| 0 | 6 | 0 | -8.398674 | 0.302622 | -5.068493 |
| 1 | 6 | 0 | -7.067659 | -6.300064 | 0.036463 |
| 2 | 6 | 0 | -6.591438 | -4.30403 | -1.982519 |
| 3 | 6 | 0 | -6.444654 | -1.537869 | -0.938061 |
| 4 | 6 | 0 | -6.260795 | 0.375781 | -3.136067 |
| 5 | 6 | 0 | -4.24391 | -4.891846 | -3.594049 |
| 6 | 6 | 0 | -1.830677 | -3.80484 | -2.59131 |
| 7 | 6 | 0 | -1.678339 | -2.153277 | -0.689352 |
| 8 | 6 | 0 | -4.035023 | -1.333218 | 0.721973 |
| 9 | 6 | 0 | 0.833874 | -0.888084 | 0.023789 |
| 10 | 6 | 0 | 0.743014 | -0.023834 | 2.772988 |
| 11 | 6 | 0 | -1.343118 | 1.043675 | 3.693493 |
| 12 | 6 | 0 | -3.672424 | 1.228679 | 2.082172 |
| 13 | 6 | 0 | 3.187522 | -2.643149 | -0.073056 |
| 14 | 6 | 0 | 3.836824 | -3.246942 | 2.68597 |
| 15 | 6 | 0 | 3.014313 | -0.936796 | 4.288984 |
| 16 | 6 | 0 | -8.790734 | -0.949554 | 0.661709 |
| 17 | 6 | 0 | -9.759749 | -7.172158 | 0.324926 |
| 18 | 6 | 0 | -5.220016 | -7.344966 | 1.400034 |
| 19 | 6 | 0 | 5.172605 | 1.073404 | 4.663977 |
| 20 | 6 | 0 | 4.126924 | 3.607819 | 5.591131 |
| 21 | 6 | 0 | 6.905354 | 1.475931 | 2.375634 |
| 22 | 6 | 0 | 9.182998 | 3.216511 | 2.914436 |
| 23 | 6 | 0 | 10.944924 | 3.231827 | 0.706741 |
| 24 | 6 | 0 | 10.812784 | 4.7569 | -1.314204 |
| 25 | 6 | 0 | 8.847078 | 6.785262 | -1.656215 |
| 26 | 1 | 0 | -4.299752 | -2.704851 | 2.268657 |
| 27 | 6 | 0 | 1.156857 | 1.351858 | -1.832623 |
| 28 | 6 | 0 | 2.223873 | -1.857814 | 6.929638 |
| 29 | 6 | 0 | -8.149313 | 2.39564 | -7.015721 |
| 30 | 8 | 0 | -6.528146 | 3.9841 | -7.028839 |
| 31 | 8 | 0 | -9.956847 | 2.401143 | -8.85781 |
| 32 | 6 | 0 | 12.755241 | 4.344049 | -3.333933 |
| 33 | 8 | 0 | 14.379186 | 2.745226 | -3.270016 |
| 34 | 8 | 0 | 12.637413 | 5.918558 | -5.374739 |
| 35 | 1 | 0 | -8.449232 | -1.506823 | -6.087603 |
| 36 | 1 | 0 | -10.254807 | 0.511132 | -4.156732 |
| 37 | 1 | 0 | -8.249706 | -4.374238 | -3.23338 |
| 38 | 1 | 0 | -4.462232 | 0.139237 | -4.1295 |
| 39 | 1 | 0 | -6.215267 | 2.286213 | -2.339441 |
| 40 | 1 | 0 | -4.518342 | -4.165806 | -5.527307 |
| 41 | 1 | 0 | -4.064595 | -6.948953 | -3.803911 |
| 42 | 1 | 0 | -0.128295 | -4.349942 | -3.61164 |
| 43 | 1 | 0 | -1.476739 | 1.574982 | 5.67506 |
| 44 | 1 | 0 | -5.313523 | 1.618316 | 3.27649 |
| 45 | 1 | 0 | -3.547167 | 2.790813 | 0.724455 |
| 46 | 1 | 0 | 4.744199 | -1.654578 | -1.009137 |
| 47 | 1 | 0 | 2.840726 | -4.367778 | -1.152885 |
| 48 | 1 | 0 | 2.708415 | -4.875941 | 3.299675 |
| 49 | 1 | 0 | 5.824334 | -3.757329 | 2.972443 |
| 50 | 1 | 0 | -8.76578 | -2.009557 | 2.44 |
| 51 | 1 | 0 | -8.899597 | 1.065905 | 1.115901 |
| 52 | 1 | 0 | -10.532546 | -1.449556 | -0.338609 |
| 53 | 1 | 0 | -11.044931 | -5.605009 | 0.745396 |
| 54 | 1 | 0 | -10.425918 | -8.023399 | -1.446912 |
| 55 | 1 | 0 | -9.941331 | -8.582106 | 1.824166 |
| 56 | 1 | 0 | -5.632854 | -8.793173 | 2.796015 |
| 57 | 1 | 0 | -3.252347 | -6.820147 | 1.139527 |
| 58 | 1 | 0 | 6.375483 | 0.282851 | 6.170264 |
| 59 | 1 | 0 | 2.765443 | 3.375175 | 7.126944 |
| 60 | 1 | 0 | 3.158489 | 4.601625 | 4.053375 |
| 61 | 1 | 0 | 5.637895 | 4.824212 | 6.306024 |
| 62 | 1 | 0 | 7.646092 | -0.339981 | 1.715163 |
| 63 | 1 | 0 | 5.839482 | 2.290615 | 0.798269 |
| 64 | 1 | 0 | 8.533637 | 5.130905 | 3.35199 |
| 65 | 1 | 0 | 10.184612 | 2.519518 | 4.591877 |
| 66 | 1 | 0 | 12.433266 | 1.81106 | 0.673668 |
| 67 | 1 | 0 | 7.544432 | 6.849384 | -0.058238 |
| 68 | 1 | 0 | 9.695314 | 8.673738 | -1.818504 |
| 69 | 1 | 0 | 7.690789 | 6.448562 | -3.347594 |
| 70 | 1 | 0 | -0.536331 | 2.531181 | -1.930506 |
| 71 | 1 | 0 | 2.741814 | 2.563528 | -1.287346 |
| 72 | 1 | 0 | 1.511148 | 0.617876 | -3.736073 |
| 73 | 1 | 0 | 0.620229 | -3.155221 | 6.78537 |
| 74 | 1 | 0 | 3.798968 | -2.859261 | 7.829464 |
| 75 | 1 | 0 | 1.669712 | -0.310826 | 8.184107 |
| 76 | 1 | 0 | -11.119158 | 1.017062 | -8.593794 |
| 77 | 1 | 0 | 11.261702 | 7.104026 | -5.1953 |

| C1000009_en_ | | Standard Orientation (A.U.) | | | |
| --- | --- | --- | --- | --- | --- |
| Center number | Atomic number | Atomic Type | X | Y | Z |
| 0 | 6 | 0 | -6.736613 | 0.485722 | -6.299054 |
| 1 | 6 | 0 | -6.757016 | -5.795095 | -0.55217 |
| 2 | 6 | 0 | -5.822316 | -4.067799 | -2.65742 |
| 3 | 6 | 0 | -5.459708 | -1.244523 | -1.837428 |
| 4 | 6 | 0 | -4.788625 | 0.407678 | -4.157801 |
| 5 | 6 | 0 | -3.401623 | -5.064303 | -3.920525 |
| 6 | 6 | 0 | -1.006616 | -4.176149 | -2.703488 |
| 7 | 6 | 0 | -0.871546 | -2.38836 | -0.926158 |
| 8 | 6 | 0 | -3.247292 | -1.146478 | 0.084296 |
| 9 | 6 | 0 | 1.690539 | -1.449926 | 0.056295 |
| 10 | 6 | 0 | 1.385825 | -0.18236 | 2.626799 |
| 11 | 6 | 0 | -0.630071 | 1.234732 | 3.135494 |
| 12 | 6 | 0 | -2.707628 | 1.489045 | 1.219844 |
| 13 | 6 | 0 | 3.654069 | -3.577965 | 0.586165 |
| 14 | 6 | 0 | 3.81771 | -3.763694 | 3.471812 |
| 15 | 6 | 0 | 3.429059 | -1.033888 | 4.45703 |
| 16 | 6 | 0 | -7.885585 | -0.260043 | -0.595347 |
| 17 | 6 | 0 | -9.545266 | -6.356351 | -0.550644 |
| 18 | 6 | 0 | -5.222077 | -6.88057 | 1.130932 |
| 19 | 6 | 0 | 5.903463 | 0.564057 | 4.129803 |
| 20 | 6 | 0 | 8.063387 | -0.349856 | 5.832218 |
| 21 | 6 | 0 | 5.438055 | 3.401136 | 4.535065 |
| 22 | 6 | 0 | 7.565633 | 5.110971 | 3.500104 |
| 23 | 6 | 0 | 7.675942 | 4.99916 | 0.689465 |
| 24 | 6 | 0 | 9.534306 | 4.094665 | -0.783036 |
| 25 | 6 | 0 | 12.007123 | 3.033633 | 0.117571 |
| 26 | 1 | 0 | -3.864078 | -2.292832 | 1.712667 |
| 27 | 6 | 0 | 2.732207 | 0.431312 | -1.927179 |
| 28 | 6 | 0 | 2.53653 | -1.031493 | 7.206913 |
| 29 | 6 | 0 | -8.912585 | 2.258916 | -5.809302 |
| 30 | 8 | 0 | -8.772006 | 4.282381 | -4.760548 |
| 31 | 8 | 0 | -11.118135 | 1.3644 | -6.783828 |
| 32 | 6 | 0 | 9.041789 | 4.126966 | -3.549709 |
| 33 | 8 | 0 | 7.13882 | 4.872304 | -4.589225 |
| 34 | 8 | 0 | 11.025371 | 3.168676 | -4.8883 |
| 35 | 1 | 0 | -5.817103 | 1.238878 | -8.002985 |
| 36 | 1 | 0 | -7.459062 | -1.379749 | -6.816252 |
| 37 | 1 | 0 | -7.328353 | -4.081867 | -4.085855 |
| 38 | 1 | 0 | -2.976804 | -0.223872 | -4.929168 |
| 39 | 1 | 0 | -4.510263 | 2.357804 | -3.519895 |
| 40 | 1 | 0 | -3.374815 | -4.48937 | -5.922851 |
| 41 | 1 | 0 | -3.454029 | -7.139194 | -3.946039 |
| 42 | 1 | 0 | 0.725355 | -5.026591 | -3.423995 |
| 43 | 1 | 0 | -0.885057 | 2.090502 | 4.988565 |
| 44 | 1 | 0 | -4.414562 | 2.216575 | 2.129629 |
| 45 | 1 | 0 | -2.203145 | 2.854211 | -0.258645 |
| 46 | 1 | 0 | 5.491793 | -3.054947 | -0.216555 |
| 47 | 1 | 0 | 3.115544 | -5.380737 | -0.263657 |
| 48 | 1 | 0 | 2.250953 | -4.926332 | 4.180484 |
| 49 | 1 | 0 | 5.573393 | -4.638442 | 4.12814 |
| 50 | 1 | 0 | -9.536042 | -0.618281 | -1.796207 |
| 51 | 1 | 0 | -8.216686 | -1.195772 | 1.222025 |
| 52 | 1 | 0 | -7.798574 | 1.779866 | -0.281622 |
| 53 | 1 | 0 | -10.083514 | -7.296053 | -2.320989 |
| 54 | 1 | 0 | -10.068584 | -7.590987 | 1.021433 |
| 55 | 1 | 0 | -10.683253 | -4.630976 | -0.433811 |
| 56 | 1 | 0 | -5.966909 | -8.134677 | 2.576624 |
| 57 | 1 | 0 | -3.19091 | -6.585435 | 1.09483 |
| 58 | 1 | 0 | 6.513707 | 0.314183 | 2.160894 |
| 59 | 1 | 0 | 8.357086 | -2.391013 | 5.703841 |
| 60 | 1 | 0 | 7.705156 | 0.120327 | 7.816491 |
| 61 | 1 | 0 | 9.84912 | 0.545654 | 5.296746 |
| 62 | 1 | 0 | 3.658904 | 3.944758 | 3.622558 |
| 63 | 1 | 0 | 5.221522 | 3.790952 | 6.5613 |
| 64 | 1 | 0 | 7.132146 | 7.065613 | 4.053566 |
| 65 | 1 | 0 | 9.377605 | 4.636038 | 4.374742 |
| 66 | 1 | 0 | 5.994798 | 5.680356 | -0.289603 |
| 67 | 1 | 0 | 13.598765 | 4.012528 | -0.769379 |
| 68 | 1 | 0 | 12.204484 | 3.197861 | 2.163034 |
| 69 | 1 | 0 | 12.183436 | 1.034923 | -0.394838 |
| 70 | 1 | 0 | 4.49456 | 1.298182 | -1.284305 |
| 71 | 1 | 0 | 3.102653 | -0.560702 | -3.706362 |
| 72 | 1 | 0 | 1.387862 | 1.950874 | -2.321934 |
| 73 | 1 | 0 | 3.912314 | -1.973097 | 8.431994 |
| 74 | 1 | 0 | 2.248132 | 0.883115 | 7.937052 |
| 75 | 1 | 0 | 0.739597 | -2.043149 | 7.365252 |
| 76 | 1 | 0 | -12.381038 | 2.658344 | -6.472428 |
| 77 | 1 | 0 | 10.527512 | 3.259266 | -6.649629 |

Table S3.Gibbs free energies^a^ and equilibrium populations^b^ of low-energy conformers of compound **2**.

| Conformers | ∆G | P(%)/100 | Single point energy(a.u.) |
| --- | --- | --- | --- |
| C2000001_tddft_ | 0.00000 | 49.31 | -1470.5505621191 |
| C2000002_tddft_ | 0.00112 | 15.04 | -1470.5494414055 |
| C2000003_tddft_ | 0.00202 | 5.81 | -1470.5485438399 |
| C2000004_tddft_ | 0.00313 | 1.78 | -1470.5474292097 |
| C2000005_tddft_ | 0.00427 | 0.53 | -1470.5462896133 |
| C2000006_tddft_ | 0.00292 | 2.24 | -1470.5476427967 |
| C2000007_tddft_ | 0.00255 | 3.32 | -1470.5480169807 |
| C2000008_tddft_ | 0.00107 | 15.85 | -1470.5494912381 |
| C2000009_tddft_ | 0.00197 | 6.12 | -1470.5485929429 |

^a^PBE0-D3(BJ)/def2-TZVP, in kcal/mol.
^b^From ∆G values at 298.15K.

Table S4.Cartesian coordinates for the low-energy reoptimized random reseach conformers of Compound **2** at PBE0-D3(BJ)/def2-SVP level of theory in methanol.

| C2000001_en_ | | Standard Orientation (Ångstroms) | | | |
| --- | --- | --- | --- | --- | --- |
| Center number | Atomic number | Atomic Type | X | Y | Z |
| 0 | 6 | 0 | -6.213403 | -3.797323 | -5.291306 |
| 1 | 6 | 0 | -7.924407 | -6.266273 | 2.451101 |
| 2 | 6 | 0 | -5.961086 | -5.713523 | 0.42706 |
| 3 | 6 | 0 | -5.745907 | -2.919766 | -0.46869 |
| 4 | 6 | 0 | -4.52843 | -2.905599 | -3.117789 |
| 5 | 6 | 0 | -3.447788 | -6.772971 | 1.182662 |
| 6 | 6 | 0 | -1.391076 | -5.369963 | 1.615377 |
| 7 | 6 | 0 | -1.405566 | -2.63949 | 1.490864 |
| 8 | 6 | 0 | -3.995159 | -1.452158 | 1.374717 |
| 9 | 6 | 0 | 0.727158 | -1.269881 | 1.585135 |
| 10 | 6 | 0 | 0.817598 | 1.587321 | 1.539081 |
| 11 | 6 | 0 | -1.745621 | 2.641955 | 2.358572 |
| 12 | 6 | 0 | -3.922131 | 1.39335 | 0.954929 |
| 13 | 6 | 0 | 3.356098 | -2.290296 | 1.930308 |
| 14 | 6 | 0 | 4.98705 | 0.022041 | 2.520942 |
| 15 | 6 | 0 | 3.148126 | 2.132827 | 3.34269 |
| 16 | 6 | 0 | 1.368984 | 2.446185 | -1.180686 |
| 17 | 6 | 0 | -8.348732 | -1.693299 | -0.561382 |
| 18 | 6 | 0 | -7.508103 | -5.327401 | 5.088417 |
| 19 | 6 | 0 | -9.93417 | -7.668756 | 1.886485 |
| 20 | 6 | 0 | 4.385816 | 4.763499 | 3.02704 |
| 21 | 6 | 0 | 7.039183 | 4.888038 | 4.158145 |
| 22 | 6 | 0 | 2.800478 | 6.953566 | 4.065626 |
| 23 | 6 | 0 | 3.28688 | 9.479917 | 2.719693 |
| 24 | 6 | 0 | 2.267614 | 9.447003 | 0.108852 |
| 25 | 6 | 0 | 3.51747 | 9.456104 | -2.088873 |
| 26 | 6 | 0 | 6.33382 | 9.59462 | -2.341428 |
| 27 | 1 | 0 | -4.811526 | -1.736769 | 3.249575 |
| 28 | 6 | 0 | 2.409865 | 1.653943 | 6.094417 |
| 29 | 6 | 0 | -4.714734 | -4.085889 | -7.703831 |
| 30 | 8 | 0 | -4.100155 | -6.077256 | -8.614873 |
| 31 | 8 | 0 | -3.988579 | -1.893152 | -8.851599 |
| 32 | 6 | 0 | 1.933698 | 9.262452 | -4.423296 |
| 33 | 8 | 0 | -0.311626 | 8.842913 | -4.443162 |
| 34 | 8 | 0 | 3.15453 | 9.562167 | -6.67048 |
| 35 | 1 | 0 | -7.032032 | -5.636826 | -4.896658 |
| 36 | 1 | 0 | -7.770836 | -2.479252 | -5.587715 |
| 37 | 1 | 0 | -6.587318 | -6.806061 | -1.207175 |
| 38 | 1 | 0 | -3.892182 | -0.98914 | -3.524651 |
| 39 | 1 | 0 | -2.829684 | -4.064065 | -3.078794 |
| 40 | 1 | 0 | -3.343789 | -8.811292 | 1.352243 |
| 41 | 1 | 0 | 0.370815 | -6.312308 | 2.056026 |
| 42 | 1 | 0 | -1.839641 | 4.67153 | 2.030512 |
| 43 | 1 | 0 | -2.009347 | 2.34546 | 4.377448 |
| 44 | 1 | 0 | -5.694046 | 2.223869 | 1.595646 |
| 45 | 1 | 0 | -3.786324 | 1.849699 | -1.046089 |
| 46 | 1 | 0 | 3.417536 | -3.654929 | 3.473259 |
| 47 | 1 | 0 | 4.026759 | -3.29215 | 0.257717 |
| 48 | 1 | 0 | 6.039722 | 0.610098 | 0.852668 |
| 49 | 1 | 0 | 6.37939 | -0.375908 | 3.979486 |
| 50 | 1 | 0 | 3.225347 | 1.851732 | -1.829005 |
| 51 | 1 | 0 | 0.007875 | 1.62776 | -2.478772 |
| 52 | 1 | 0 | 1.245915 | 4.487609 | -1.371127 |
| 53 | 1 | 0 | -9.714577 | -2.89626 | -1.516035 |
| 54 | 1 | 0 | -9.08016 | -1.344131 | 1.327734 |
| 55 | 1 | 0 | -8.292685 | 0.110246 | -1.550966 |
| 56 | 1 | 0 | -7.82562 | -3.295697 | 5.238986 |
| 57 | 1 | 0 | -8.790072 | -6.243839 | 6.408001 |
| 58 | 1 | 0 | -5.576071 | -5.672012 | 5.708229 |
| 59 | 1 | 0 | -11.334889 | -8.160007 | 3.29416 |
| 60 | 1 | 0 | -10.256987 | -8.379372 | -0.004446 |
| 61 | 1 | 0 | 4.600617 | 5.063575 | 0.999902 |
| 62 | 1 | 0 | 7.83522 | 6.776591 | 3.957859 |
| 63 | 1 | 0 | 8.340169 | 3.58979 | 3.240807 |
| 64 | 1 | 0 | 7.029604 | 4.454104 | 6.169913 |
| 65 | 1 | 0 | 0.796717 | 6.533077 | 3.928079 |
| 66 | 1 | 0 | 3.193219 | 7.199597 | 6.075339 |
| 67 | 1 | 0 | 2.320843 | 10.969103 | 3.775893 |
| 68 | 1 | 0 | 5.289952 | 9.944063 | 2.769762 |
| 69 | 1 | 0 | 0.227397 | 9.309615 | -0.035096 |
| 70 | 1 | 0 | 7.263268 | 9.544021 | -0.515423 |
| 71 | 1 | 0 | 7.080106 | 8.007956 | -3.429245 |
| 72 | 1 | 0 | 6.949829 | 11.337396 | -3.265179 |
| 73 | 1 | 0 | 1.449858 | -0.154242 | 6.29598 |
| 74 | 1 | 0 | 4.074427 | 1.597188 | 7.301469 |
| 75 | 1 | 0 | 1.156067 | 3.097884 | 6.84236 |
| 76 | 1 | 0 | -4.610547 | -0.457695 | -7.9087 |
| 77 | 1 | 0 | 4.924987 | 9.910405 | -6.390605 |

| C2000001_tddft_ | | Standard Orientation (Ångstroms) | | | |
| --- | --- | --- | --- | --- | --- |
| Center number | Atomic number | Atomic Type | X | Y | Z |
| 0 | 6 | 0 | -6.213403 | -3.797323 | -5.291306 |
| 1 | 6 | 0 | -7.924407 | -6.266273 | 2.451101 |
| 2 | 6 | 0 | -5.961086 | -5.713523 | 0.42706 |
| 3 | 6 | 0 | -5.745907 | -2.919766 | -0.46869 |
| 4 | 6 | 0 | -4.52843 | -2.905599 | -3.117789 |
| 5 | 6 | 0 | -3.447788 | -6.772971 | 1.182662 |
| 6 | 6 | 0 | -1.391076 | -5.369963 | 1.615377 |
| 7 | 6 | 0 | -1.405566 | -2.63949 | 1.490864 |
| 8 | 6 | 0 | -3.995159 | -1.452158 | 1.374717 |
| 9 | 6 | 0 | 0.727158 | -1.269881 | 1.585135 |
| 10 | 6 | 0 | 0.817598 | 1.587321 | 1.539081 |
| 11 | 6 | 0 | -1.745621 | 2.641955 | 2.358572 |
| 12 | 6 | 0 | -3.922131 | 1.39335 | 0.954929 |
| 13 | 6 | 0 | 3.356098 | -2.290296 | 1.930308 |
| 14 | 6 | 0 | 4.98705 | 0.022041 | 2.520942 |
| 15 | 6 | 0 | 3.148126 | 2.132827 | 3.34269 |
| 16 | 6 | 0 | 1.368984 | 2.446185 | -1.180686 |
| 17 | 6 | 0 | -8.348732 | -1.693299 | -0.561382 |
| 18 | 6 | 0 | -7.508103 | -5.327401 | 5.088417 |
| 19 | 6 | 0 | -9.93417 | -7.668756 | 1.886485 |
| 20 | 6 | 0 | 4.385816 | 4.763499 | 3.02704 |
| 21 | 6 | 0 | 7.039183 | 4.888038 | 4.158145 |
| 22 | 6 | 0 | 2.800478 | 6.953566 | 4.065626 |
| 23 | 6 | 0 | 3.28688 | 9.479917 | 2.719693 |
| 24 | 6 | 0 | 2.267614 | 9.447003 | 0.108852 |
| 25 | 6 | 0 | 3.51747 | 9.456104 | -2.088873 |
| 26 | 6 | 0 | 6.33382 | 9.59462 | -2.341428 |
| 27 | 1 | 0 | -4.811526 | -1.736769 | 3.249575 |
| 28 | 6 | 0 | 2.409865 | 1.653943 | 6.094417 |
| 29 | 6 | 0 | -4.714734 | -4.085889 | -7.703831 |
| 30 | 8 | 0 | -4.100155 | -6.077256 | -8.614873 |
| 31 | 8 | 0 | -3.988579 | -1.893152 | -8.851599 |
| 32 | 6 | 0 | 1.933698 | 9.262452 | -4.423296 |
| 33 | 8 | 0 | -0.311626 | 8.842913 | -4.443162 |
| 34 | 8 | 0 | 3.15453 | 9.562167 | -6.67048 |
| 35 | 1 | 0 | -7.032032 | -5.636826 | -4.896658 |
| 36 | 1 | 0 | -7.770836 | -2.479252 | -5.587715 |
| 37 | 1 | 0 | -6.587318 | -6.806061 | -1.207175 |
| 38 | 1 | 0 | -3.892182 | -0.98914 | -3.524651 |
| 39 | 1 | 0 | -2.829684 | -4.064065 | -3.078794 |
| 40 | 1 | 0 | -3.343789 | -8.811292 | 1.352243 |
| 41 | 1 | 0 | 0.370815 | -6.312308 | 2.056026 |
| 42 | 1 | 0 | -1.839641 | 4.67153 | 2.030512 |
| 43 | 1 | 0 | -2.009347 | 2.34546 | 4.377448 |
| 44 | 1 | 0 | -5.694046 | 2.223869 | 1.595646 |
| 45 | 1 | 0 | -3.786324 | 1.849699 | -1.046089 |
| 46 | 1 | 0 | 3.417536 | -3.654929 | 3.473259 |
| 47 | 1 | 0 | 4.026759 | -3.29215 | 0.257717 |
| 48 | 1 | 0 | 6.039722 | 0.610098 | 0.852668 |
| 49 | 1 | 0 | 6.37939 | -0.375908 | 3.979486 |
| 50 | 1 | 0 | 3.225347 | 1.851732 | -1.829005 |
| 51 | 1 | 0 | 0.007875 | 1.62776 | -2.478772 |
| 52 | 1 | 0 | 1.245915 | 4.487609 | -1.371127 |
| 53 | 1 | 0 | -9.714577 | -2.89626 | -1.516035 |
| 54 | 1 | 0 | -9.08016 | -1.344131 | 1.327734 |
| 55 | 1 | 0 | -8.292685 | 0.110246 | -1.550966 |
| 56 | 1 | 0 | -7.82562 | -3.295697 | 5.238986 |
| 57 | 1 | 0 | -8.790072 | -6.243839 | 6.408001 |
| 58 | 1 | 0 | -5.576071 | -5.672012 | 5.708229 |
| 59 | 1 | 0 | -11.334889 | -8.160007 | 3.29416 |
| 60 | 1 | 0 | -10.256987 | -8.379372 | -0.004446 |
| 61 | 1 | 0 | 4.600617 | 5.063575 | 0.999902 |
| 62 | 1 | 0 | 7.83522 | 6.776591 | 3.957859 |
| 63 | 1 | 0 | 8.340169 | 3.58979 | 3.240807 |
| 64 | 1 | 0 | 7.029604 | 4.454104 | 6.169913 |
| 65 | 1 | 0 | 0.796717 | 6.533077 | 3.928079 |
| 66 | 1 | 0 | 3.193219 | 7.199597 | 6.075339 |
| 67 | 1 | 0 | 2.320843 | 10.969103 | 3.775893 |
| 68 | 1 | 0 | 5.289952 | 9.944063 | 2.769762 |
| 69 | 1 | 0 | 0.227397 | 9.309615 | -0.035096 |
| 70 | 1 | 0 | 7.263268 | 9.544021 | -0.515423 |
| 71 | 1 | 0 | 7.080106 | 8.007956 | -3.429245 |
| 72 | 1 | 0 | 6.949829 | 11.337396 | -3.265179 |
| 73 | 1 | 0 | 1.449858 | -0.154242 | 6.29598 |
| 74 | 1 | 0 | 4.074427 | 1.597188 | 7.301469 |
| 75 | 1 | 0 | 1.156067 | 3.097884 | 6.84236 |
| 76 | 1 | 0 | -4.610547 | -0.457695 | -7.9087 |
| 77 | 1 | 0 | 4.924987 | 9.910405 | -6.390605 |

| C2000002_en_ | | Standard Orientation (Ångstroms) | | | |
| --- | --- | --- | --- | --- | --- |
| Center number | Atomic number | Atomic Type | X | Y | Z |
| 0 | 6 | 0 | -6.216176 | -4.992667 | -5.085929 |
| 1 | 6 | 0 | -8.137782 | -5.373874 | 2.946626 |
| 2 | 6 | 0 | -6.533248 | -5.4206 | 0.55333 |
| 3 | 6 | 0 | -6.030519 | -2.786429 | -0.669827 |
| 4 | 6 | 0 | -4.901436 | -3.105862 | -3.341123 |
| 5 | 6 | 0 | -4.102803 | -6.78293 | 0.999843 |
| 6 | 6 | 0 | -1.896364 | -5.589519 | 1.317897 |
| 7 | 6 | 0 | -1.645385 | -2.868249 | 1.215951 |
| 8 | 6 | 0 | -4.079921 | -1.383171 | 1.002046 |
| 9 | 6 | 0 | 0.620795 | -1.732925 | 1.375705 |
| 10 | 6 | 0 | 1.04914 | 1.092129 | 1.30064 |
| 11 | 6 | 0 | -1.436028 | 2.487573 | 1.775324 |
| 12 | 6 | 0 | -3.612094 | 1.355096 | 0.274832 |
| 13 | 6 | 0 | 3.10593 | -3.020798 | 1.865978 |
| 14 | 6 | 0 | 4.890056 | -0.909699 | 2.725886 |
| 15 | 6 | 0 | 3.210154 | 1.394031 | 3.354973 |
| 16 | 6 | 0 | 2.020909 | 1.794488 | -1.350619 |
| 17 | 6 | 0 | -8.471851 | -1.271097 | -0.847118 |
| 18 | 6 | 0 | -10.902643 | -5.878806 | 2.61813 |
| 19 | 6 | 0 | -7.142258 | -5.042448 | 5.231696 |
| 20 | 6 | 0 | 4.759321 | 3.867645 | 3.187849 |
| 21 | 6 | 0 | 7.253441 | 3.724137 | 4.63644 |
| 22 | 6 | 0 | 3.298343 | 6.225267 | 4.012925 |
| 23 | 6 | 0 | 4.334739 | 8.702727 | 2.910032 |
| 24 | 6 | 0 | 4.01929 | 8.783933 | 0.126274 |
| 25 | 6 | 0 | 5.785535 | 8.767765 | -1.681985 |
| 26 | 6 | 0 | 8.576146 | 8.72865 | -1.207723 |
| 27 | 1 | 0 | -4.91641 | -1.356582 | 2.893367 |
| 28 | 6 | 0 | 2.129749 | 1.024336 | 6.008102 |
| 29 | 6 | 0 | -5.054524 | -4.993352 | -7.693135 |
| 30 | 8 | 0 | -6.064134 | -4.211621 | -9.574524 |
| 31 | 8 | 0 | -2.662514 | -5.950755 | -7.837144 |
| 32 | 6 | 0 | 4.839628 | 8.751796 | -4.34823 |
| 33 | 8 | 0 | 2.643111 | 8.597716 | -4.950184 |
| 34 | 8 | 0 | 6.622665 | 8.908033 | -6.201493 |
| 35 | 1 | 0 | -6.082246 | -6.899725 | -4.311693 |
| 36 | 1 | 0 | -8.206153 | -4.543992 | -5.313717 |
| 37 | 1 | 0 | -7.644334 | -6.504185 | -0.805887 |
| 38 | 1 | 0 | -4.927435 | -1.267477 | -4.267738 |
| 39 | 1 | 0 | -2.916733 | -3.618906 | -3.160811 |
| 40 | 1 | 0 | -4.194198 | -8.821337 | 1.174422 |
| 41 | 1 | 0 | -0.212991 | -6.702516 | 1.654506 |
| 42 | 1 | 0 | -1.254594 | 4.473775 | 1.266262 |
| 43 | 1 | 0 | -1.923467 | 2.418068 | 3.774067 |
| 44 | 1 | 0 | -5.307751 | 2.468035 | 0.624513 |
| 45 | 1 | 0 | -3.219728 | 1.523756 | -1.737598 |
| 46 | 1 | 0 | 2.920998 | -4.480126 | 3.306309 |
| 47 | 1 | 0 | 3.821427 | -3.971281 | 0.179985 |
| 48 | 1 | 0 | 6.211933 | -0.421524 | 1.227034 |
| 49 | 1 | 0 | 6.022098 | -1.478142 | 4.34419 |
| 50 | 1 | 0 | 3.813676 | 0.895738 | -1.793636 |
| 51 | 1 | 0 | 0.684385 | 1.190729 | -2.787557 |
| 52 | 1 | 0 | 2.263124 | 3.825065 | -1.555074 |
| 53 | 1 | 0 | -9.946324 | -2.336522 | -1.806954 |
| 54 | 1 | 0 | -9.175072 | -0.763694 | 1.017852 |
| 55 | 1 | 0 | -8.188199 | 0.464292 | -1.908597 |
| 56 | 1 | 0 | -11.196372 | -7.769146 | 1.84098 |
| 57 | 1 | 0 | -11.91015 | -5.770122 | 4.406307 |
| 58 | 1 | 0 | -11.786874 | -4.566857 | 1.302613 |
| 59 | 1 | 0 | -8.318366 | -5.017009 | 6.904699 |
| 60 | 1 | 0 | -5.132858 | -4.828128 | 5.521131 |
| 61 | 1 | 0 | 5.247239 | 4.106069 | 1.20058 |
| 62 | 1 | 0 | 8.315397 | 5.475606 | 4.43957 |
| 63 | 1 | 0 | 8.467714 | 2.214147 | 3.956142 |
| 64 | 1 | 0 | 6.950942 | 3.430025 | 6.651675 |
| 65 | 1 | 0 | 1.323971 | 6.073796 | 3.47411 |
| 66 | 1 | 0 | 3.318468 | 6.369497 | 6.069854 |
| 67 | 1 | 0 | 3.250866 | 10.261565 | 3.725019 |
| 68 | 1 | 0 | 6.285504 | 8.996252 | 3.488866 |
| 69 | 1 | 0 | 2.07751 | 8.787759 | -0.532752 |
| 70 | 1 | 0 | 9.006329 | 8.685985 | 0.795747 |
| 71 | 1 | 0 | 9.468337 | 7.062377 | -2.037562 |
| 72 | 1 | 0 | 9.521341 | 10.397979 | -1.973826 |
| 73 | 1 | 0 | 0.984593 | -0.68152 | 6.114329 |
| 74 | 1 | 0 | 3.643782 | 0.838653 | 7.388227 |
| 75 | 1 | 0 | 0.945825 | 2.591445 | 6.607901 |
| 76 | 1 | 0 | -2.110965 | -6.48035 | -6.176759 |
| 77 | 1 | 0 | 8.290792 | 9.033082 | -5.4699 |

| C2000002_tddft_ | | Standard Orientation (Ångstroms) | | | |
| --- | --- | --- | --- | --- | --- |
| Center number | Atomic number | Atomic Type | X | Y | Z |
| 0 | 6 | 0 | -6.216176 | -4.992667 | -5.085929 |
| 1 | 6 | 0 | -8.137782 | -5.373874 | 2.946626 |
| 2 | 6 | 0 | -6.533248 | -5.4206 | 0.55333 |
| 3 | 6 | 0 | -6.030519 | -2.786429 | -0.669827 |
| 4 | 6 | 0 | -4.901436 | -3.105862 | -3.341123 |
| 5 | 6 | 0 | -4.102803 | -6.78293 | 0.999843 |
| 6 | 6 | 0 | -1.896364 | -5.589519 | 1.317897 |
| 7 | 6 | 0 | -1.645385 | -2.868249 | 1.215951 |
| 8 | 6 | 0 | -4.079921 | -1.383171 | 1.002046 |
| 9 | 6 | 0 | 0.620795 | -1.732925 | 1.375705 |
| 10 | 6 | 0 | 1.04914 | 1.092129 | 1.30064 |
| 11 | 6 | 0 | -1.436028 | 2.487573 | 1.775324 |
| 12 | 6 | 0 | -3.612094 | 1.355096 | 0.274832 |
| 13 | 6 | 0 | 3.10593 | -3.020798 | 1.865978 |
| 14 | 6 | 0 | 4.890056 | -0.909699 | 2.725886 |
| 15 | 6 | 0 | 3.210154 | 1.394031 | 3.354973 |
| 16 | 6 | 0 | 2.020909 | 1.794488 | -1.350619 |
| 17 | 6 | 0 | -8.471851 | -1.271097 | -0.847118 |
| 18 | 6 | 0 | -10.902643 | -5.878806 | 2.61813 |
| 19 | 6 | 0 | -7.142258 | -5.042448 | 5.231696 |
| 20 | 6 | 0 | 4.759321 | 3.867645 | 3.187849 |
| 21 | 6 | 0 | 7.253441 | 3.724137 | 4.63644 |
| 22 | 6 | 0 | 3.298343 | 6.225267 | 4.012925 |
| 23 | 6 | 0 | 4.334739 | 8.702727 | 2.910032 |
| 24 | 6 | 0 | 4.01929 | 8.783933 | 0.126274 |
| 25 | 6 | 0 | 5.785535 | 8.767765 | -1.681985 |
| 26 | 6 | 0 | 8.576146 | 8.72865 | -1.207723 |
| 27 | 1 | 0 | -4.91641 | -1.356582 | 2.893367 |
| 28 | 6 | 0 | 2.129749 | 1.024336 | 6.008102 |
| 29 | 6 | 0 | -5.054524 | -4.993352 | -7.693135 |
| 30 | 8 | 0 | -6.064134 | -4.211621 | -9.574524 |
| 31 | 8 | 0 | -2.662514 | -5.950755 | -7.837144 |
| 32 | 6 | 0 | 4.839628 | 8.751796 | -4.34823 |
| 33 | 8 | 0 | 2.643111 | 8.597716 | -4.950184 |
| 34 | 8 | 0 | 6.622665 | 8.908033 | -6.201493 |
| 35 | 1 | 0 | -6.082246 | -6.899725 | -4.311693 |
| 36 | 1 | 0 | -8.206153 | -4.543992 | -5.313717 |
| 37 | 1 | 0 | -7.644334 | -6.504185 | -0.805887 |
| 38 | 1 | 0 | -4.927435 | -1.267477 | -4.267738 |
| 39 | 1 | 0 | -2.916733 | -3.618906 | -3.160811 |
| 40 | 1 | 0 | -4.194198 | -8.821337 | 1.174422 |
| 41 | 1 | 0 | -0.212991 | -6.702516 | 1.654506 |
| 42 | 1 | 0 | -1.254594 | 4.473775 | 1.266262 |
| 43 | 1 | 0 | -1.923467 | 2.418068 | 3.774067 |
| 44 | 1 | 0 | -5.307751 | 2.468035 | 0.624513 |
| 45 | 1 | 0 | -3.219728 | 1.523756 | -1.737598 |
| 46 | 1 | 0 | 2.920998 | -4.480126 | 3.306309 |
| 47 | 1 | 0 | 3.821427 | -3.971281 | 0.179985 |
| 48 | 1 | 0 | 6.211933 | -0.421524 | 1.227034 |
| 49 | 1 | 0 | 6.022098 | -1.478142 | 4.34419 |
| 50 | 1 | 0 | 3.813676 | 0.895738 | -1.793636 |
| 51 | 1 | 0 | 0.684385 | 1.190729 | -2.787557 |
| 52 | 1 | 0 | 2.263124 | 3.825065 | -1.555074 |
| 53 | 1 | 0 | -9.946324 | -2.336522 | -1.806954 |
| 54 | 1 | 0 | -9.175072 | -0.763694 | 1.017852 |
| 55 | 1 | 0 | -8.188199 | 0.464292 | -1.908597 |
| 56 | 1 | 0 | -11.196372 | -7.769146 | 1.84098 |
| 57 | 1 | 0 | -11.91015 | -5.770122 | 4.406307 |
| 58 | 1 | 0 | -11.786874 | -4.566857 | 1.302613 |
| 59 | 1 | 0 | -8.318366 | -5.017009 | 6.904699 |
| 60 | 1 | 0 | -5.132858 | -4.828128 | 5.521131 |
| 61 | 1 | 0 | 5.247239 | 4.106069 | 1.20058 |
| 62 | 1 | 0 | 8.315397 | 5.475606 | 4.43957 |
| 63 | 1 | 0 | 8.467714 | 2.214147 | 3.956142 |
| 64 | 1 | 0 | 6.950942 | 3.430025 | 6.651675 |
| 65 | 1 | 0 | 1.323971 | 6.073796 | 3.47411 |
| 66 | 1 | 0 | 3.318468 | 6.369497 | 6.069854 |
| 67 | 1 | 0 | 3.250866 | 10.261565 | 3.725019 |
| 68 | 1 | 0 | 6.285504 | 8.996252 | 3.488866 |
| 69 | 1 | 0 | 2.07751 | 8.787759 | -0.532752 |
| 70 | 1 | 0 | 9.006329 | 8.685985 | 0.795747 |
| 71 | 1 | 0 | 9.468337 | 7.062377 | -2.037562 |
| 72 | 1 | 0 | 9.521341 | 10.397979 | -1.973826 |
| 73 | 1 | 0 | 0.984593 | -0.68152 | 6.114329 |
| 74 | 1 | 0 | 3.643782 | 0.838653 | 7.388227 |
| 75 | 1 | 0 | 0.945825 | 2.591445 | 6.607901 |
| 76 | 1 | 0 | -2.110965 | -6.48035 | -6.176759 |
| 77 | 1 | 0 | 8.290792 | 9.033082 | -5.4699 |

| C2000003_en_ | | Standard Orientation (Ångstroms) | | | |
| --- | --- | --- | --- | --- | --- |
| Center number | Atomic number | Atomic Type | X | Y | Z |
| 0 | 6 | 0 | -6.658434 | -4.883047 | -4.95791 |
| 1 | 6 | 0 | -7.997139 | -5.735201 | 3.146135 |
| 2 | 6 | 0 | -6.536593 | -5.587016 | 0.666423 |
| 3 | 6 | 0 | -6.254758 | -2.878482 | -0.461589 |
| 4 | 6 | 0 | -5.306217 | -3.017581 | -3.216239 |
| 5 | 6 | 0 | -4.014366 | -6.828354 | 0.912375 |
| 6 | 6 | 0 | -1.854852 | -5.531413 | 1.13099 |
| 7 | 6 | 0 | -1.747238 | -2.798424 | 1.101246 |
| 8 | 6 | 0 | -4.26517 | -1.446583 | 1.139072 |
| 9 | 6 | 0 | 0.468512 | -1.555297 | 1.114842 |
| 10 | 6 | 0 | 0.751445 | 1.291291 | 1.086663 |
| 11 | 6 | 0 | -1.746951 | 2.51345 | 1.87772 |
| 12 | 6 | 0 | -3.999453 | 1.347142 | 0.52522 |
| 13 | 6 | 0 | 3.036484 | -2.740899 | 1.380785 |
| 14 | 6 | 0 | 4.801803 | -0.567963 | 2.103797 |
| 15 | 6 | 0 | 3.103878 | 1.669885 | 2.907971 |
| 16 | 6 | 0 | 1.399903 | 2.128029 | -1.618602 |
| 17 | 6 | 0 | -8.778345 | -1.493709 | -0.400095 |
| 18 | 6 | 0 | -10.735733 | -6.417963 | 2.94571 |
| 19 | 6 | 0 | -6.898704 | -5.422121 | 5.385786 |
| 20 | 6 | 0 | 4.564663 | 4.185292 | 2.582899 |
| 21 | 6 | 0 | 7.080679 | 4.201818 | 3.997207 |
| 22 | 6 | 0 | 3.032025 | 6.535376 | 3.269686 |
| 23 | 6 | 0 | 4.153139 | 9.043486 | 2.328103 |
| 24 | 6 | 0 | 4.661129 | 9.016734 | -0.440609 |
| 25 | 6 | 0 | 2.99163 | 9.409742 | -2.298525 |
| 26 | 6 | 0 | 0.263264 | 10.039458 | -1.872569 |
| 27 | 1 | 0 | -4.961272 | -1.557097 | 3.083222 |
| 28 | 6 | 0 | 2.305669 | 1.261273 | 5.653372 |
| 29 | 6 | 0 | -5.769848 | -4.598094 | -7.655121 |
| 30 | 8 | 0 | -6.991145 | -3.683348 | -9.341206 |
| 31 | 8 | 0 | -3.371122 | -5.423389 | -8.123516 |
| 32 | 6 | 0 | 3.962003 | 9.146231 | -4.941375 |
| 33 | 8 | 0 | 6.12239 | 8.660066 | -5.493116 |
| 34 | 8 | 0 | 2.243646 | 9.471228 | -6.834662 |
| 35 | 1 | 0 | -6.311914 | -6.820906 | -4.34382 |
| 36 | 1 | 0 | -8.687546 | -4.57258 | -4.95548 |
| 37 | 1 | 0 | -7.665616 | -6.674107 | -0.675427 |
| 38 | 1 | 0 | -5.487361 | -1.141801 | -4.04627 |
| 39 | 1 | 0 | -3.292273 | -3.437678 | -3.20874 |
| 40 | 1 | 0 | -3.989857 | -8.873274 | 1.018662 |
| 41 | 1 | 0 | -0.098455 | -6.563578 | 1.314498 |
| 42 | 1 | 0 | -1.724718 | 4.534722 | 1.493579 |
| 43 | 1 | 0 | -2.024636 | 2.303076 | 3.905204 |
| 44 | 1 | 0 | -5.705626 | 2.348765 | 1.092623 |
| 45 | 1 | 0 | -3.81349 | 1.630885 | -1.504507 |
| 46 | 1 | 0 | 3.023194 | -4.205735 | 2.827656 |
| 47 | 1 | 0 | 3.647521 | -3.663691 | -0.360375 |
| 48 | 1 | 0 | 5.976918 | -0.02092 | 0.505166 |
| 49 | 1 | 0 | 6.090237 | -1.107763 | 3.610325 |
| 50 | 1 | 0 | 3.162117 | 1.317554 | -2.291529 |
| 51 | 1 | 0 | -0.064179 | 1.532241 | -2.929236 |
| 52 | 1 | 0 | 1.569071 | 4.173693 | -1.745954 |
| 53 | 1 | 0 | -10.250116 | -2.577115 | -1.34328 |
| 54 | 1 | 0 | -9.396052 | -1.147217 | 1.530929 |
| 55 | 1 | 0 | -8.656598 | 0.318999 | -1.360467 |
| 56 | 1 | 0 | -10.940046 | -8.311093 | 2.146896 |
| 57 | 1 | 0 | -11.659343 | -6.407631 | 4.781566 |
| 58 | 1 | 0 | -11.768195 | -5.149523 | 1.697793 |
| 59 | 1 | 0 | -7.979458 | -5.537742 | 7.118254 |
| 60 | 1 | 0 | -4.893803 | -5.092872 | 5.581445 |
| 61 | 1 | 0 | 5.022721 | 4.317885 | 0.577615 |
| 62 | 1 | 0 | 8.168037 | 5.888534 | 3.545344 |
| 63 | 1 | 0 | 8.259118 | 2.593613 | 3.506599 |
| 64 | 1 | 0 | 6.809201 | 4.18684 | 6.038346 |
| 65 | 1 | 0 | 1.14252 | 6.380646 | 2.491042 |
| 66 | 1 | 0 | 2.802915 | 6.657581 | 5.315944 |
| 67 | 1 | 0 | 2.839381 | 10.555257 | 2.817746 |
| 68 | 1 | 0 | 5.90093 | 9.462455 | 3.330167 |
| 69 | 1 | 0 | 6.563065 | 8.554325 | -1.039928 |
| 70 | 1 | 0 | -0.170082 | 10.221148 | 0.123498 |
| 71 | 1 | 0 | -0.267999 | 11.8259 | -2.760992 |
| 72 | 1 | 0 | -0.989824 | 8.572836 | -2.610761 |
| 73 | 1 | 0 | 1.18298 | -0.451633 | 5.85055 |
| 74 | 1 | 0 | 3.952746 | 1.053475 | 6.865578 |
| 75 | 1 | 0 | 1.188433 | 2.817742 | 6.39428 |
| 76 | 1 | 0 | -2.63571 | -6.08275 | -6.585409 |
| 77 | 1 | 0 | 0.592226 | 9.829702 | -6.143877 |

| C2000003_tddft_ | | Standard Orientation (Ångstroms) | | | |
| --- | --- | --- | --- | --- | --- |
| Center number | Atomic number | Atomic Type | X | Y | Z |
| 0 | 6 | 0 | -6.658434 | -4.883047 | -4.95791 |
| 1 | 6 | 0 | -7.997139 | -5.735201 | 3.146135 |
| 2 | 6 | 0 | -6.536593 | -5.587016 | 0.666423 |
| 3 | 6 | 0 | -6.254758 | -2.878482 | -0.461589 |
| 4 | 6 | 0 | -5.306217 | -3.017581 | -3.216239 |
| 5 | 6 | 0 | -4.014366 | -6.828354 | 0.912375 |
| 6 | 6 | 0 | -1.854852 | -5.531413 | 1.13099 |
| 7 | 6 | 0 | -1.747238 | -2.798424 | 1.101246 |
| 8 | 6 | 0 | -4.26517 | -1.446583 | 1.139072 |
| 9 | 6 | 0 | 0.468512 | -1.555297 | 1.114842 |
| 10 | 6 | 0 | 0.751445 | 1.291291 | 1.086663 |
| 11 | 6 | 0 | -1.746951 | 2.51345 | 1.87772 |
| 12 | 6 | 0 | -3.999453 | 1.347142 | 0.52522 |
| 13 | 6 | 0 | 3.036484 | -2.740899 | 1.380785 |
| 14 | 6 | 0 | 4.801803 | -0.567963 | 2.103797 |
| 15 | 6 | 0 | 3.103878 | 1.669885 | 2.907971 |
| 16 | 6 | 0 | 1.399903 | 2.128029 | -1.618602 |
| 17 | 6 | 0 | -8.778345 | -1.493709 | -0.400095 |
| 18 | 6 | 0 | -10.735733 | -6.417963 | 2.94571 |
| 19 | 6 | 0 | -6.898704 | -5.422121 | 5.385786 |
| 20 | 6 | 0 | 4.564663 | 4.185292 | 2.582899 |
| 21 | 6 | 0 | 7.080679 | 4.201818 | 3.997207 |
| 22 | 6 | 0 | 3.032025 | 6.535376 | 3.269686 |
| 23 | 6 | 0 | 4.153139 | 9.043486 | 2.328103 |
| 24 | 6 | 0 | 4.661129 | 9.016734 | -0.440609 |
| 25 | 6 | 0 | 2.99163 | 9.409742 | -2.298525 |
| 26 | 6 | 0 | 0.263264 | 10.039458 | -1.872569 |
| 27 | 1 | 0 | -4.961272 | -1.557097 | 3.083222 |
| 28 | 6 | 0 | 2.305669 | 1.261273 | 5.653372 |
| 29 | 6 | 0 | -5.769848 | -4.598094 | -7.655121 |
| 30 | 8 | 0 | -6.991145 | -3.683348 | -9.341206 |
| 31 | 8 | 0 | -3.371122 | -5.423389 | -8.123516 |
| 32 | 6 | 0 | 3.962003 | 9.146231 | -4.941375 |
| 33 | 8 | 0 | 6.12239 | 8.660066 | -5.493116 |
| 34 | 8 | 0 | 2.243646 | 9.471228 | -6.834662 |
| 35 | 1 | 0 | -6.311914 | -6.820906 | -4.34382 |
| 36 | 1 | 0 | -8.687546 | -4.57258 | -4.95548 |
| 37 | 1 | 0 | -7.665616 | -6.674107 | -0.675427 |
| 38 | 1 | 0 | -5.487361 | -1.141801 | -4.04627 |
| 39 | 1 | 0 | -3.292273 | -3.437678 | -3.20874 |
| 40 | 1 | 0 | -3.989857 | -8.873274 | 1.018662 |
| 41 | 1 | 0 | -0.098455 | -6.563578 | 1.314498 |
| 42 | 1 | 0 | -1.724718 | 4.534722 | 1.493579 |
| 43 | 1 | 0 | -2.024636 | 2.303076 | 3.905204 |
| 44 | 1 | 0 | -5.705626 | 2.348765 | 1.092623 |
| 45 | 1 | 0 | -3.81349 | 1.630885 | -1.504507 |
| 46 | 1 | 0 | 3.023194 | -4.205735 | 2.827656 |
| 47 | 1 | 0 | 3.647521 | -3.663691 | -0.360375 |
| 48 | 1 | 0 | 5.976918 | -0.02092 | 0.505166 |
| 49 | 1 | 0 | 6.090237 | -1.107763 | 3.610325 |
| 50 | 1 | 0 | 3.162117 | 1.317554 | -2.291529 |
| 51 | 1 | 0 | -0.064179 | 1.532241 | -2.929236 |
| 52 | 1 | 0 | 1.569071 | 4.173693 | -1.745954 |
| 53 | 1 | 0 | -10.250116 | -2.577115 | -1.34328 |
| 54 | 1 | 0 | -9.396052 | -1.147217 | 1.530929 |
| 55 | 1 | 0 | -8.656598 | 0.318999 | -1.360467 |
| 56 | 1 | 0 | -10.940046 | -8.311093 | 2.146896 |
| 57 | 1 | 0 | -11.659343 | -6.407631 | 4.781566 |
| 58 | 1 | 0 | -11.768195 | -5.149523 | 1.697793 |
| 59 | 1 | 0 | -7.979458 | -5.537742 | 7.118254 |
| 60 | 1 | 0 | -4.893803 | -5.092872 | 5.581445 |
| 61 | 1 | 0 | 5.022721 | 4.317885 | 0.577615 |
| 62 | 1 | 0 | 8.168037 | 5.888534 | 3.545344 |
| 63 | 1 | 0 | 8.259118 | 2.593613 | 3.506599 |
| 64 | 1 | 0 | 6.809201 | 4.18684 | 6.038346 |
| 65 | 1 | 0 | 1.14252 | 6.380646 | 2.491042 |
| 66 | 1 | 0 | 2.802915 | 6.657581 | 5.315944 |
| 67 | 1 | 0 | 2.839381 | 10.555257 | 2.817746 |
| 68 | 1 | 0 | 5.90093 | 9.462455 | 3.330167 |
| 69 | 1 | 0 | 6.563065 | 8.554325 | -1.039928 |
| 70 | 1 | 0 | -0.170082 | 10.221148 | 0.123498 |
| 71 | 1 | 0 | -0.267999 | 11.8259 | -2.760992 |
| 72 | 1 | 0 | -0.989824 | 8.572836 | -2.610761 |
| 73 | 1 | 0 | 1.18298 | -0.451633 | 5.85055 |
| 74 | 1 | 0 | 3.952746 | 1.053475 | 6.865578 |
| 75 | 1 | 0 | 1.188433 | 2.817742 | 6.39428 |
| 76 | 1 | 0 | -2.63571 | -6.08275 | -6.585409 |
| 77 | 1 | 0 | 0.592226 | 9.829702 | -6.143877 |

| C2000004_en_ | | Standard Orientation (Ångstroms) | | | |
| --- | --- | --- | --- | --- | --- |
| Center number | Atomic number | Atomic Type | X | Y | Z |
| 0 | 6 | 0 | -6.790528 | -5.075075 | -4.987358 |
| 1 | 6 | 0 | -8.087873 | -6.151165 | 3.105712 |
| 2 | 6 | 0 | -6.644517 | -5.916083 | 0.622487 |
| 3 | 6 | 0 | -6.415215 | -3.176148 | -0.439924 |
| 4 | 6 | 0 | -5.47159 | -3.230445 | -3.199341 |
| 5 | 6 | 0 | -4.100018 | -7.118945 | 0.82677 |
| 6 | 6 | 0 | -1.962017 | -5.790479 | 1.066255 |
| 7 | 6 | 0 | -1.902679 | -3.055664 | 1.100897 |
| 8 | 6 | 0 | -4.443673 | -1.749196 | 1.188233 |
| 9 | 6 | 0 | 0.290869 | -1.775544 | 1.130064 |
| 10 | 6 | 0 | 0.519963 | 1.075988 | 1.172392 |
| 11 | 6 | 0 | -1.990086 | 2.238137 | 2.011894 |
| 12 | 6 | 0 | -4.234378 | 1.065534 | 0.64937 |
| 13 | 6 | 0 | 2.881144 | -2.922489 | 1.354462 |
| 14 | 6 | 0 | 4.613533 | -0.730794 | 2.096647 |
| 15 | 6 | 0 | 2.880793 | 1.445681 | 2.983786 |
| 16 | 6 | 0 | 1.119415 | 1.990672 | -1.519114 |
| 17 | 6 | 0 | -8.962367 | -1.836772 | -0.338317 |
| 18 | 6 | 0 | -10.814774 | -6.877198 | 2.901181 |
| 19 | 6 | 0 | -6.981568 | -5.875111 | 5.346258 |
| 20 | 6 | 0 | 4.277613 | 4.002922 | 2.737222 |
| 21 | 6 | 0 | 6.873333 | 3.977239 | 4.004901 |
| 22 | 6 | 0 | 2.757388 | 6.273196 | 3.689559 |
| 23 | 6 | 0 | 3.54039 | 8.780484 | 2.464585 |
| 24 | 6 | 0 | 1.969588 | 10.904569 | 3.413662 |
| 25 | 6 | 0 | 2.553542 | 12.572872 | 5.221776 |
| 26 | 6 | 0 | 5.014832 | 12.608912 | 6.623022 |
| 27 | 1 | 0 | -5.124139 | -1.924755 | 3.133131 |
| 28 | 6 | 0 | 2.10774 | 0.94374 | 5.720591 |
| 29 | 6 | 0 | -5.85655 | -4.753233 | -7.665199 |
| 30 | 8 | 0 | -7.051965 | -3.83346 | -9.366377 |
| 31 | 8 | 0 | -3.441623 | -5.556534 | -8.094966 |
| 32 | 6 | 0 | 0.572309 | 14.489011 | 5.853564 |
| 33 | 8 | 0 | -1.526834 | 14.53967 | 4.96019 |
| 34 | 8 | 0 | 1.229915 | 16.298249 | 7.570171 |
| 35 | 1 | 0 | -6.445105 | -7.020585 | -4.397277 |
| 36 | 1 | 0 | -8.820661 | -4.772961 | -5.012159 |
| 37 | 1 | 0 | -7.761411 | -6.990462 | -0.739309 |
| 38 | 1 | 0 | -5.687317 | -1.337855 | -3.980833 |
| 39 | 1 | 0 | -3.450398 | -3.614291 | -3.204384 |
| 40 | 1 | 0 | -4.03911 | -9.165146 | 0.885441 |
| 41 | 1 | 0 | -0.187346 | -6.796558 | 1.21829 |
| 42 | 1 | 0 | -2.001791 | 4.266944 | 1.668459 |
| 43 | 1 | 0 | -2.248007 | 1.975556 | 4.035857 |
| 44 | 1 | 0 | -5.95121 | 2.019983 | 1.262035 |
| 45 | 1 | 0 | -4.075914 | 1.413108 | -1.372581 |
| 46 | 1 | 0 | 2.905181 | -4.408441 | 2.779836 |
| 47 | 1 | 0 | 3.492988 | -3.809964 | -0.404841 |
| 48 | 1 | 0 | 5.741596 | -0.119997 | 0.487045 |
| 49 | 1 | 0 | 5.945277 | -1.271212 | 3.565113 |
| 50 | 1 | 0 | 2.909917 | 1.268424 | -2.219725 |
| 51 | 1 | 0 | -0.328723 | 1.361258 | -2.831129 |
| 52 | 1 | 0 | 1.187511 | 4.046417 | -1.610422 |
| 53 | 1 | 0 | -10.417026 | -2.919558 | -1.309015 |
| 54 | 1 | 0 | -9.583692 | -1.551965 | 1.601413 |
| 55 | 1 | 0 | -8.872948 | 0.002713 | -1.249536 |
| 56 | 1 | 0 | -10.988385 | -8.757535 | 2.065638 |
| 57 | 1 | 0 | -11.731621 | -6.919016 | 4.739888 |
| 58 | 1 | 0 | -11.872494 | -5.602447 | 1.681704 |
| 59 | 1 | 0 | -8.049342 | -6.05153 | 7.08153 |
| 60 | 1 | 0 | -4.981773 | -5.514343 | 5.537943 |
| 61 | 1 | 0 | 4.620845 | 4.28833 | 0.723054 |
| 62 | 1 | 0 | 7.812155 | 5.79579 | 3.786471 |
| 63 | 1 | 0 | 8.124399 | 2.563326 | 3.196217 |
| 64 | 1 | 0 | 6.729209 | 3.609452 | 6.024221 |
| 65 | 1 | 0 | 0.753523 | 6.001463 | 3.349568 |
| 66 | 1 | 0 | 2.958293 | 6.444991 | 5.735503 |
| 67 | 1 | 0 | 5.544698 | 9.125504 | 2.765993 |
| 68 | 1 | 0 | 3.278295 | 8.617563 | 0.423333 |
| 69 | 1 | 0 | 0.094266 | 11.077486 | 2.606945 |
| 70 | 1 | 0 | 6.216243 | 11.033387 | 6.093907 |
| 71 | 1 | 0 | 6.099995 | 14.328562 | 6.258354 |
| 72 | 1 | 0 | 4.732652 | 12.46393 | 8.66259 |
| 73 | 1 | 0 | 1.018118 | -0.794451 | 5.874684 |
| 74 | 1 | 0 | 3.762594 | 0.732684 | 6.922105 |
| 75 | 1 | 0 | 0.964059 | 2.458858 | 6.504286 |
| 76 | 1 | 0 | -2.734154 | -6.228735 | -6.549249 |
| 77 | 1 | 0 | 2.956333 | 16.061866 | 8.114904 |

| C2000004_tddft_ | | Standard Orientation (Ångstroms) | | | |
| --- | --- | --- | --- | --- | --- |
| Center number | Atomic number | Atomic Type | X | Y | Z |
| 0 | 6 | 0 | -6.790528 | -5.075075 | -4.987358 |
| 1 | 6 | 0 | -8.087873 | -6.151165 | 3.105712 |
| 2 | 6 | 0 | -6.644517 | -5.916083 | 0.622487 |
| 3 | 6 | 0 | -6.415215 | -3.176148 | -0.439924 |
| 4 | 6 | 0 | -5.47159 | -3.230445 | -3.199341 |
| 5 | 6 | 0 | -4.100018 | -7.118945 | 0.82677 |
| 6 | 6 | 0 | -1.962017 | -5.790479 | 1.066255 |
| 7 | 6 | 0 | -1.902679 | -3.055664 | 1.100897 |
| 8 | 6 | 0 | -4.443673 | -1.749196 | 1.188233 |
| 9 | 6 | 0 | 0.290869 | -1.775544 | 1.130064 |
| 10 | 6 | 0 | 0.519963 | 1.075988 | 1.172392 |
| 11 | 6 | 0 | -1.990086 | 2.238137 | 2.011894 |
| 12 | 6 | 0 | -4.234378 | 1.065534 | 0.64937 |
| 13 | 6 | 0 | 2.881144 | -2.922489 | 1.354462 |
| 14 | 6 | 0 | 4.613533 | -0.730794 | 2.096647 |
| 15 | 6 | 0 | 2.880793 | 1.445681 | 2.983786 |
| 16 | 6 | 0 | 1.119415 | 1.990672 | -1.519114 |
| 17 | 6 | 0 | -8.962367 | -1.836772 | -0.338317 |
| 18 | 6 | 0 | -10.814774 | -6.877198 | 2.901181 |
| 19 | 6 | 0 | -6.981568 | -5.875111 | 5.346258 |
| 20 | 6 | 0 | 4.277613 | 4.002922 | 2.737222 |
| 21 | 6 | 0 | 6.873333 | 3.977239 | 4.004901 |
| 22 | 6 | 0 | 2.757388 | 6.273196 | 3.689559 |
| 23 | 6 | 0 | 3.54039 | 8.780484 | 2.464585 |
| 24 | 6 | 0 | 1.969588 | 10.904569 | 3.413662 |
| 25 | 6 | 0 | 2.553542 | 12.572872 | 5.221776 |
| 26 | 6 | 0 | 5.014832 | 12.608912 | 6.623022 |
| 27 | 1 | 0 | -5.124139 | -1.924755 | 3.133131 |
| 28 | 6 | 0 | 2.10774 | 0.94374 | 5.720591 |
| 29 | 6 | 0 | -5.85655 | -4.753233 | -7.665199 |
| 30 | 8 | 0 | -7.051965 | -3.83346 | -9.366377 |
| 31 | 8 | 0 | -3.441623 | -5.556534 | -8.094966 |
| 32 | 6 | 0 | 0.572309 | 14.489011 | 5.853564 |
| 33 | 8 | 0 | -1.526834 | 14.53967 | 4.96019 |
| 34 | 8 | 0 | 1.229915 | 16.298249 | 7.570171 |
| 35 | 1 | 0 | -6.445105 | -7.020585 | -4.397277 |
| 36 | 1 | 0 | -8.820661 | -4.772961 | -5.012159 |
| 37 | 1 | 0 | -7.761411 | -6.990462 | -0.739309 |
| 38 | 1 | 0 | -5.687317 | -1.337855 | -3.980833 |
| 39 | 1 | 0 | -3.450398 | -3.614291 | -3.204384 |
| 40 | 1 | 0 | -4.03911 | -9.165146 | 0.885441 |
| 41 | 1 | 0 | -0.187346 | -6.796558 | 1.21829 |
| 42 | 1 | 0 | -2.001791 | 4.266944 | 1.668459 |
| 43 | 1 | 0 | -2.248007 | 1.975556 | 4.035857 |
| 44 | 1 | 0 | -5.95121 | 2.019983 | 1.262035 |
| 45 | 1 | 0 | -4.075914 | 1.413108 | -1.372581 |
| 46 | 1 | 0 | 2.905181 | -4.408441 | 2.779836 |
| 47 | 1 | 0 | 3.492988 | -3.809964 | -0.404841 |
| 48 | 1 | 0 | 5.741596 | -0.119997 | 0.487045 |
| 49 | 1 | 0 | 5.945277 | -1.271212 | 3.565113 |
| 50 | 1 | 0 | 2.909917 | 1.268424 | -2.219725 |
| 51 | 1 | 0 | -0.328723 | 1.361258 | -2.831129 |
| 52 | 1 | 0 | 1.187511 | 4.046417 | -1.610422 |
| 53 | 1 | 0 | -10.417026 | -2.919558 | -1.309015 |
| 54 | 1 | 0 | -9.583692 | -1.551965 | 1.601413 |
| 55 | 1 | 0 | -8.872948 | 0.002713 | -1.249536 |
| 56 | 1 | 0 | -10.988385 | -8.757535 | 2.065638 |
| 57 | 1 | 0 | -11.731621 | -6.919016 | 4.739888 |
| 58 | 1 | 0 | -11.872494 | -5.602447 | 1.681704 |
| 59 | 1 | 0 | -8.049342 | -6.05153 | 7.08153 |
| 60 | 1 | 0 | -4.981773 | -5.514343 | 5.537943 |
| 61 | 1 | 0 | 4.620845 | 4.28833 | 0.723054 |
| 62 | 1 | 0 | 7.812155 | 5.79579 | 3.786471 |
| 63 | 1 | 0 | 8.124399 | 2.563326 | 3.196217 |
| 64 | 1 | 0 | 6.729209 | 3.609452 | 6.024221 |
| 65 | 1 | 0 | 0.753523 | 6.001463 | 3.349568 |
| 66 | 1 | 0 | 2.958293 | 6.444991 | 5.735503 |
| 67 | 1 | 0 | 5.544698 | 9.125504 | 2.765993 |
| 68 | 1 | 0 | 3.278295 | 8.617563 | 0.423333 |
| 69 | 1 | 0 | 0.094266 | 11.077486 | 2.606945 |
| 70 | 1 | 0 | 6.216243 | 11.033387 | 6.093907 |
| 71 | 1 | 0 | 6.099995 | 14.328562 | 6.258354 |
| 72 | 1 | 0 | 4.732652 | 12.46393 | 8.66259 |
| 73 | 1 | 0 | 1.018118 | -0.794451 | 5.874684 |
| 74 | 1 | 0 | 3.762594 | 0.732684 | 6.922105 |
| 75 | 1 | 0 | 0.964059 | 2.458858 | 6.504286 |
| 76 | 1 | 0 | -2.734154 | -6.228735 | -6.549249 |
| 77 | 1 | 0 | 2.956333 | 16.061866 | 8.114904 |

| C2000005_en_ | | Standard Orientation (Ångstroms) | | | |
| --- | --- | --- | --- | --- | --- |
| Center number | Atomic number | Atomic Type | X | Y | Z |
| 0 | 6 | 0 | -6.554636 | -4.676634 | -4.986664 |
| 1 | 6 | 0 | -8.221242 | -5.218304 | 3.113419 |
| 2 | 6 | 0 | -6.668824 | -5.234439 | 0.685476 |
| 3 | 6 | 0 | -6.19256 | -2.586046 | -0.518595 |
| 4 | 6 | 0 | -5.144918 | -2.87377 | -3.225176 |
| 5 | 6 | 0 | -4.231303 | -6.605305 | 1.064455 |
| 6 | 6 | 0 | -2.008327 | -5.426062 | 1.316206 |
| 7 | 6 | 0 | -1.742776 | -2.706781 | 1.190745 |
| 8 | 6 | 0 | -4.180345 | -1.217539 | 1.110137 |
| 9 | 6 | 0 | 0.538647 | -1.590717 | 1.215053 |
| 10 | 6 | 0 | 0.977139 | 1.232906 | 1.084761 |
| 11 | 6 | 0 | -1.457452 | 2.621269 | 1.792597 |
| 12 | 6 | 0 | -3.753015 | 1.542576 | 0.438573 |
| 13 | 6 | 0 | 3.029236 | -2.907471 | 1.591667 |
| 14 | 6 | 0 | 4.910218 | -0.800695 | 2.2067 |
| 15 | 6 | 0 | 3.328156 | 1.54721 | 2.920093 |
| 16 | 6 | 0 | 1.689588 | 1.933567 | -1.64454 |
| 17 | 6 | 0 | -8.627058 | -1.053719 | -0.59981 |
| 18 | 6 | 0 | -10.993626 | -5.713287 | 2.837617 |
| 19 | 6 | 0 | -7.176392 | -4.924293 | 5.381369 |
| 20 | 6 | 0 | 4.912197 | 3.974867 | 2.539941 |
| 21 | 6 | 0 | 7.468377 | 3.870334 | 3.877288 |
| 22 | 6 | 0 | 3.5247 | 6.407382 | 3.270023 |
| 23 | 6 | 0 | 4.463099 | 8.736782 | 1.822877 |
| 24 | 6 | 0 | 3.017752 | 11.033564 | 2.538563 |
| 25 | 6 | 0 | 1.046015 | 12.049213 | 1.322347 |
| 26 | 6 | 0 | -0.042279 | 11.024798 | -1.084155 |
| 27 | 1 | 0 | -4.940703 | -1.244945 | 3.032954 |
| 28 | 6 | 0 | 2.477492 | 1.280761 | 5.666578 |
| 29 | 6 | 0 | -5.398169 | -4.655201 | -7.59554 |
| 30 | 8 | 0 | -6.330132 | -3.686943 | -9.430065 |
| 31 | 8 | 0 | -3.094329 | -5.800448 | -7.792823 |
| 32 | 6 | 0 | -0.120159 | 14.337462 | 2.504223 |
| 33 | 8 | 0 | 0.545439 | 15.271252 | 4.477077 |
| 34 | 8 | 0 | -2.081694 | 15.418722 | 1.224587 |
| 35 | 1 | 0 | -6.497998 | -6.600873 | -4.247549 |
| 36 | 1 | 0 | -8.524495 | -4.13668 | -5.19236 |
| 37 | 1 | 0 | -7.810121 | -6.302596 | -0.661523 |
| 38 | 1 | 0 | -5.144928 | -1.014312 | -4.109628 |
| 39 | 1 | 0 | -3.17168 | -3.444671 | -3.114017 |
| 40 | 1 | 0 | -4.329443 | -8.642658 | 1.24678 |
| 41 | 1 | 0 | -0.322848 | -6.549455 | 1.604044 |
| 42 | 1 | 0 | -1.30818 | 4.622315 | 1.336564 |
| 43 | 1 | 0 | -1.778187 | 2.495056 | 3.820835 |
| 44 | 1 | 0 | -5.409349 | 2.646336 | 0.962341 |
| 45 | 1 | 0 | -3.532144 | 1.774871 | -1.594491 |
| 46 | 1 | 0 | 2.910688 | -4.27131 | 3.130285 |
| 47 | 1 | 0 | 3.603976 | -3.978864 | -0.075042 |
| 48 | 1 | 0 | 6.08955 | -0.382588 | 0.572272 |
| 49 | 1 | 0 | 6.189527 | -1.333007 | 3.723867 |
| 50 | 1 | 0 | 3.429213 | 1.032438 | -2.258409 |
| 51 | 1 | 0 | 0.218505 | 1.339496 | -2.94766 |
| 52 | 1 | 0 | 1.920663 | 3.969436 | -1.851498 |
| 53 | 1 | 0 | -10.134003 | -2.090656 | -1.539375 |
| 54 | 1 | 0 | -9.276298 | -0.578716 | 1.292898 |
| 55 | 1 | 0 | -8.360283 | 0.700544 | -1.635523 |
| 56 | 1 | 0 | -11.30748 | -7.588692 | 2.032763 |
| 57 | 1 | 0 | -11.960749 | -5.636701 | 4.649301 |
| 58 | 1 | 0 | -11.905886 | -4.376707 | 1.566834 |
| 59 | 1 | 0 | -8.316769 | -4.922067 | 7.078938 |
| 60 | 1 | 0 | -5.160888 | -4.72321 | 5.633839 |
| 61 | 1 | 0 | 5.322088 | 4.084826 | 0.520766 |
| 62 | 1 | 0 | 8.533932 | 5.598173 | 3.539319 |
| 63 | 1 | 0 | 8.633055 | 2.312455 | 3.216889 |
| 64 | 1 | 0 | 7.255719 | 3.682265 | 5.915161 |
| 65 | 1 | 0 | 1.513903 | 6.226366 | 2.919218 |
| 66 | 1 | 0 | 3.709396 | 6.745173 | 5.296368 |
| 67 | 1 | 0 | 6.45912 | 9.049801 | 2.232062 |
| 68 | 1 | 0 | 4.336894 | 8.358989 | -0.197571 |
| 69 | 1 | 0 | 3.562071 | 11.950011 | 4.287892 |
| 70 | 1 | 0 | 0.938769 | 9.334294 | -1.702986 |
| 71 | 1 | 0 | -2.027058 | 10.500681 | -0.864017 |
| 72 | 1 | 0 | 0.08358 | 12.379486 | -2.638219 |
| 73 | 1 | 0 | 1.265527 | -0.363105 | 5.912502 |
| 74 | 1 | 0 | 4.095721 | 1.036367 | 6.910091 |
| 75 | 1 | 0 | 1.433059 | 2.920207 | 6.329625 |
| 76 | 1 | 0 | -2.594285 | -6.46043 | -6.162961 |
| 77 | 1 | 0 | -2.415537 | 14.505834 | -0.32109 |

| C2000005_tddft_ | | Standard Orientation (Ångstroms) | | | |
| --- | --- | --- | --- | --- | --- |
| Center number | Atomic number | Atomic Type | X | Y | Z |
| 0 | 6 | 0 | -6.554636 | -4.676634 | -4.986664 |
| 1 | 6 | 0 | -8.221242 | -5.218304 | 3.113419 |
| 2 | 6 | 0 | -6.668824 | -5.234439 | 0.685476 |
| 3 | 6 | 0 | -6.19256 | -2.586046 | -0.518595 |
| 4 | 6 | 0 | -5.144918 | -2.87377 | -3.225176 |
| 5 | 6 | 0 | -4.231303 | -6.605305 | 1.064455 |
| 6 | 6 | 0 | -2.008327 | -5.426062 | 1.316206 |
| 7 | 6 | 0 | -1.742776 | -2.706781 | 1.190745 |
| 8 | 6 | 0 | -4.180345 | -1.217539 | 1.110137 |
| 9 | 6 | 0 | 0.538647 | -1.590717 | 1.215053 |
| 10 | 6 | 0 | 0.977139 | 1.232906 | 1.084761 |
| 11 | 6 | 0 | -1.457452 | 2.621269 | 1.792597 |
| 12 | 6 | 0 | -3.753015 | 1.542576 | 0.438573 |
| 13 | 6 | 0 | 3.029236 | -2.907471 | 1.591667 |
| 14 | 6 | 0 | 4.910218 | -0.800695 | 2.2067 |
| 15 | 6 | 0 | 3.328156 | 1.54721 | 2.920093 |
| 16 | 6 | 0 | 1.689588 | 1.933567 | -1.64454 |
| 17 | 6 | 0 | -8.627058 | -1.053719 | -0.59981 |
| 18 | 6 | 0 | -10.993626 | -5.713287 | 2.837617 |
| 19 | 6 | 0 | -7.176392 | -4.924293 | 5.381369 |
| 20 | 6 | 0 | 4.912197 | 3.974867 | 2.539941 |
| 21 | 6 | 0 | 7.468377 | 3.870334 | 3.877288 |
| 22 | 6 | 0 | 3.5247 | 6.407382 | 3.270023 |
| 23 | 6 | 0 | 4.463099 | 8.736782 | 1.822877 |
| 24 | 6 | 0 | 3.017752 | 11.033564 | 2.538563 |
| 25 | 6 | 0 | 1.046015 | 12.049213 | 1.322347 |
| 26 | 6 | 0 | -0.042279 | 11.024798 | -1.084155 |
| 27 | 1 | 0 | -4.940703 | -1.244945 | 3.032954 |
| 28 | 6 | 0 | 2.477492 | 1.280761 | 5.666578 |
| 29 | 6 | 0 | -5.398169 | -4.655201 | -7.59554 |
| 30 | 8 | 0 | -6.330132 | -3.686943 | -9.430065 |
| 31 | 8 | 0 | -3.094329 | -5.800448 | -7.792823 |
| 32 | 6 | 0 | -0.120159 | 14.337462 | 2.504223 |
| 33 | 8 | 0 | 0.545439 | 15.271252 | 4.477077 |
| 34 | 8 | 0 | -2.081694 | 15.418722 | 1.224587 |
| 35 | 1 | 0 | -6.497998 | -6.600873 | -4.247549 |
| 36 | 1 | 0 | -8.524495 | -4.13668 | -5.19236 |
| 37 | 1 | 0 | -7.810121 | -6.302596 | -0.661523 |
| 38 | 1 | 0 | -5.144928 | -1.014312 | -4.109628 |
| 39 | 1 | 0 | -3.17168 | -3.444671 | -3.114017 |
| 40 | 1 | 0 | -4.329443 | -8.642658 | 1.24678 |
| 41 | 1 | 0 | -0.322848 | -6.549455 | 1.604044 |
| 42 | 1 | 0 | -1.30818 | 4.622315 | 1.336564 |
| 43 | 1 | 0 | -1.778187 | 2.495056 | 3.820835 |
| 44 | 1 | 0 | -5.409349 | 2.646336 | 0.962341 |
| 45 | 1 | 0 | -3.532144 | 1.774871 | -1.594491 |
| 46 | 1 | 0 | 2.910688 | -4.27131 | 3.130285 |
| 47 | 1 | 0 | 3.603976 | -3.978864 | -0.075042 |
| 48 | 1 | 0 | 6.08955 | -0.382588 | 0.572272 |
| 49 | 1 | 0 | 6.189527 | -1.333007 | 3.723867 |
| 50 | 1 | 0 | 3.429213 | 1.032438 | -2.258409 |
| 51 | 1 | 0 | 0.218505 | 1.339496 | -2.94766 |
| 52 | 1 | 0 | 1.920663 | 3.969436 | -1.851498 |
| 53 | 1 | 0 | -10.134003 | -2.090656 | -1.539375 |
| 54 | 1 | 0 | -9.276298 | -0.578716 | 1.292898 |
| 55 | 1 | 0 | -8.360283 | 0.700544 | -1.635523 |
| 56 | 1 | 0 | -11.30748 | -7.588692 | 2.032763 |
| 57 | 1 | 0 | -11.960749 | -5.636701 | 4.649301 |
| 58 | 1 | 0 | -11.905886 | -4.376707 | 1.566834 |
| 59 | 1 | 0 | -8.316769 | -4.922067 | 7.078938 |
| 60 | 1 | 0 | -5.160888 | -4.72321 | 5.633839 |
| 61 | 1 | 0 | 5.322088 | 4.084826 | 0.520766 |
| 62 | 1 | 0 | 8.533932 | 5.598173 | 3.539319 |
| 63 | 1 | 0 | 8.633055 | 2.312455 | 3.216889 |
| 64 | 1 | 0 | 7.255719 | 3.682265 | 5.915161 |
| 65 | 1 | 0 | 1.513903 | 6.226366 | 2.919218 |
| 66 | 1 | 0 | 3.709396 | 6.745173 | 5.296368 |
| 67 | 1 | 0 | 6.45912 | 9.049801 | 2.232062 |
| 68 | 1 | 0 | 4.336894 | 8.358989 | -0.197571 |
| 69 | 1 | 0 | 3.562071 | 11.950011 | 4.287892 |
| 70 | 1 | 0 | 0.938769 | 9.334294 | -1.702986 |
| 71 | 1 | 0 | -2.027058 | 10.500681 | -0.864017 |
| 72 | 1 | 0 | 0.08358 | 12.379486 | -2.638219 |
| 73 | 1 | 0 | 1.265527 | -0.363105 | 5.912502 |
| 74 | 1 | 0 | 4.095721 | 1.036367 | 6.910091 |
| 75 | 1 | 0 | 1.433059 | 2.920207 | 6.329625 |
| 76 | 1 | 0 | -2.594285 | -6.46043 | -6.162961 |
| 77 | 1 | 0 | -2.415537 | 14.505834 | -0.32109 |

| C2000006_en_ | | Standard Orientation (Ångstroms) | | | |
| --- | --- | --- | --- | --- | --- |
| Center number | Atomic number | Atomic Type | X | Y | Z |
| 0 | 6 | 0 | -6.896718 | -4.343128 | -4.984966 |
| 1 | 6 | 0 | -8.373126 | -5.848842 | 3.024539 |
| 2 | 6 | 0 | -6.525837 | -5.623698 | 0.835248 |
| 3 | 6 | 0 | -6.147554 | -2.942358 | -0.314172 |
| 4 | 6 | 0 | -5.093019 | -3.236999 | -3.01469 |
| 5 | 6 | 0 | -4.057907 | -6.833914 | 1.507957 |
| 6 | 6 | 0 | -1.876382 | -5.573615 | 1.715956 |
| 7 | 6 | 0 | -1.688246 | -2.866517 | 1.385901 |
| 8 | 6 | 0 | -4.177137 | -1.476774 | 1.294838 |
| 9 | 6 | 0 | 0.55071 | -1.674802 | 1.294165 |
| 10 | 6 | 0 | 0.876251 | 1.157347 | 1.059352 |
| 11 | 6 | 0 | -1.57409 | 2.479091 | 1.837025 |
| 12 | 6 | 0 | -3.885966 | 1.29831 | 0.601365 |
| 13 | 6 | 0 | 3.096761 | -2.888589 | 1.631383 |
| 14 | 6 | 0 | 4.92353 | -0.695583 | 2.080184 |
| 15 | 6 | 0 | 3.285088 | 1.612748 | 2.790282 |
| 16 | 6 | 0 | 1.448827 | 1.802396 | -1.715368 |
| 17 | 6 | 0 | -8.649638 | -1.520945 | -0.376615 |
| 18 | 6 | 0 | -7.691212 | -4.743623 | 5.537661 |
| 19 | 6 | 0 | -10.524612 | -7.113962 | 2.714416 |
| 20 | 6 | 0 | 4.752886 | 4.086648 | 2.276439 |
| 21 | 6 | 0 | 7.374682 | 4.1066 | 3.485228 |
| 22 | 6 | 0 | 3.322568 | 6.487878 | 3.03207 |
| 23 | 6 | 0 | 4.038438 | 8.788519 | 1.42344 |
| 24 | 6 | 0 | 2.693238 | 11.092154 | 2.288652 |
| 25 | 6 | 0 | 0.651942 | 12.17955 | 1.266093 |
| 26 | 6 | 0 | -0.6602 | 11.253721 | -1.06801 |
| 27 | 1 | 0 | -4.903105 | -1.516419 | 3.226977 |
| 28 | 6 | 0 | 2.555552 | 1.38917 | 5.576011 |
| 29 | 6 | 0 | -5.586436 | -4.656079 | -7.501321 |
| 30 | 8 | 0 | -5.909211 | -3.3169 | -9.310613 |
| 31 | 8 | 0 | -3.883811 | -6.589774 | -7.639172 |
| 32 | 6 | 0 | -0.360926 | 14.457159 | 2.603681 |
| 33 | 8 | 0 | 0.484468 | 15.319137 | 4.539958 |
| 34 | 8 | 0 | -2.39505 | 15.613222 | 1.518293 |
| 35 | 1 | 0 | -7.641203 | -6.168271 | -4.374886 |
| 36 | 1 | 0 | -8.495725 | -3.096925 | -5.290761 |
| 37 | 1 | 0 | -7.369087 | -6.780006 | -0.650759 |
| 38 | 1 | 0 | -4.51002 | -1.388721 | -3.706532 |
| 39 | 1 | 0 | -3.374914 | -4.366514 | -2.922307 |
| 40 | 1 | 0 | -4.102573 | -8.853624 | 1.848552 |
| 41 | 1 | 0 | -0.170377 | -6.620974 | 2.13833 |
| 42 | 1 | 0 | -1.50774 | 4.47374 | 1.333728 |
| 43 | 1 | 0 | -1.804751 | 2.388487 | 3.87912 |
| 44 | 1 | 0 | -5.562363 | 2.333384 | 1.19863 |
| 45 | 1 | 0 | -3.77694 | 1.535743 | -1.438102 |
| 46 | 1 | 0 | 3.085081 | -4.185702 | 3.231823 |
| 47 | 1 | 0 | 3.639528 | -4.016716 | -0.007584 |
| 48 | 1 | 0 | 6.00604 | -0.293815 | 0.37585 |
| 49 | 1 | 0 | 6.291352 | -1.128976 | 3.550903 |
| 50 | 1 | 0 | 3.202066 | 0.959406 | -2.372271 |
| 51 | 1 | 0 | -0.041964 | 1.101104 | -2.940288 |
| 52 | 1 | 0 | 1.579185 | 3.837978 | -1.995636 |
| 53 | 1 | 0 | -10.165686 | -2.69373 | -1.118298 |
| 54 | 1 | 0 | -9.221646 | -0.92843 | 1.507468 |
| 55 | 1 | 0 | -8.523896 | 0.162006 | -1.553817 |
| 56 | 1 | 0 | -7.86275 | -2.689403 | 5.536867 |
| 57 | 1 | 0 | -8.926547 | -5.456608 | 7.016869 |
| 58 | 1 | 0 | -5.744836 | -5.17814 | 6.046128 |
| 59 | 1 | 0 | -11.853055 | -7.370901 | 4.248428 |
| 60 | 1 | 0 | -11.044789 | -7.936155 | 0.914666 |
| 61 | 1 | 0 | 5.057035 | 4.164568 | 0.23761 |
| 62 | 1 | 0 | 8.340087 | 5.882011 | 3.093556 |
| 63 | 1 | 0 | 8.577734 | 2.602132 | 2.771501 |
| 64 | 1 | 0 | 7.268559 | 3.916992 | 5.530967 |
| 65 | 1 | 0 | 1.296232 | 6.220896 | 2.869526 |
| 66 | 1 | 0 | 3.668083 | 6.915488 | 5.020126 |
| 67 | 1 | 0 | 6.070661 | 9.116176 | 1.558342 |
| 68 | 1 | 0 | 3.650076 | 8.365595 | -0.554089 |
| 69 | 1 | 0 | 3.398992 | 11.955326 | 4.007812 |
| 70 | 1 | 0 | 0.243533 | 9.584219 | -1.843403 |
| 71 | 1 | 0 | -2.621622 | 10.733932 | -0.686596 |
| 72 | 1 | 0 | -0.666528 | 12.66612 | -2.575406 |
| 73 | 1 | 0 | 1.401893 | -0.281689 | 5.90631 |
| 74 | 1 | 0 | 4.227777 | 1.213867 | 6.758791 |
| 75 | 1 | 0 | 1.490478 | 3.010461 | 6.250001 |
| 76 | 1 | 0 | -3.816197 | -7.466185 | -6.03715 |
| 77 | 1 | 0 | -2.866347 | 14.749774 | -0.019733 |

| C2000006_tddft_ | | Standard Orientation (Ångstroms) | | | |
| --- | --- | --- | --- | --- | --- |
| Center number | Atomic number | Atomic Type | X | Y | Z |
| 0 | 6 | 0 | -6.896718 | -4.343128 | -4.984966 |
| 1 | 6 | 0 | -8.373126 | -5.848842 | 3.024539 |
| 2 | 6 | 0 | -6.525837 | -5.623698 | 0.835248 |
| 3 | 6 | 0 | -6.147554 | -2.942358 | -0.314172 |
| 4 | 6 | 0 | -5.093019 | -3.236999 | -3.01469 |
| 5 | 6 | 0 | -4.057907 | -6.833914 | 1.507957 |
| 6 | 6 | 0 | -1.876382 | -5.573615 | 1.715956 |
| 7 | 6 | 0 | -1.688246 | -2.866517 | 1.385901 |
| 8 | 6 | 0 | -4.177137 | -1.476774 | 1.294838 |
| 9 | 6 | 0 | 0.55071 | -1.674802 | 1.294165 |
| 10 | 6 | 0 | 0.876251 | 1.157347 | 1.059352 |
| 11 | 6 | 0 | -1.57409 | 2.479091 | 1.837025 |
| 12 | 6 | 0 | -3.885966 | 1.29831 | 0.601365 |
| 13 | 6 | 0 | 3.096761 | -2.888589 | 1.631383 |
| 14 | 6 | 0 | 4.92353 | -0.695583 | 2.080184 |
| 15 | 6 | 0 | 3.285088 | 1.612748 | 2.790282 |
| 16 | 6 | 0 | 1.448827 | 1.802396 | -1.715368 |
| 17 | 6 | 0 | -8.649638 | -1.520945 | -0.376615 |
| 18 | 6 | 0 | -7.691212 | -4.743623 | 5.537661 |
| 19 | 6 | 0 | -10.524612 | -7.113962 | 2.714416 |
| 20 | 6 | 0 | 4.752886 | 4.086648 | 2.276439 |
| 21 | 6 | 0 | 7.374682 | 4.1066 | 3.485228 |
| 22 | 6 | 0 | 3.322568 | 6.487878 | 3.03207 |
| 23 | 6 | 0 | 4.038438 | 8.788519 | 1.42344 |
| 24 | 6 | 0 | 2.693238 | 11.092154 | 2.288652 |
| 25 | 6 | 0 | 0.651942 | 12.17955 | 1.266093 |
| 26 | 6 | 0 | -0.6602 | 11.253721 | -1.06801 |
| 27 | 1 | 0 | -4.903105 | -1.516419 | 3.226977 |
| 28 | 6 | 0 | 2.555552 | 1.38917 | 5.576011 |
| 29 | 6 | 0 | -5.586436 | -4.656079 | -7.501321 |
| 30 | 8 | 0 | -5.909211 | -3.3169 | -9.310613 |
| 31 | 8 | 0 | -3.883811 | -6.589774 | -7.639172 |
| 32 | 6 | 0 | -0.360926 | 14.457159 | 2.603681 |
| 33 | 8 | 0 | 0.484468 | 15.319137 | 4.539958 |
| 34 | 8 | 0 | -2.39505 | 15.613222 | 1.518293 |
| 35 | 1 | 0 | -7.641203 | -6.168271 | -4.374886 |
| 36 | 1 | 0 | -8.495725 | -3.096925 | -5.290761 |
| 37 | 1 | 0 | -7.369087 | -6.780006 | -0.650759 |
| 38 | 1 | 0 | -4.51002 | -1.388721 | -3.706532 |
| 39 | 1 | 0 | -3.374914 | -4.366514 | -2.922307 |
| 40 | 1 | 0 | -4.102573 | -8.853624 | 1.848552 |
| 41 | 1 | 0 | -0.170377 | -6.620974 | 2.13833 |
| 42 | 1 | 0 | -1.50774 | 4.47374 | 1.333728 |
| 43 | 1 | 0 | -1.804751 | 2.388487 | 3.87912 |
| 44 | 1 | 0 | -5.562363 | 2.333384 | 1.19863 |
| 45 | 1 | 0 | -3.77694 | 1.535743 | -1.438102 |
| 46 | 1 | 0 | 3.085081 | -4.185702 | 3.231823 |
| 47 | 1 | 0 | 3.639528 | -4.016716 | -0.007584 |
| 48 | 1 | 0 | 6.00604 | -0.293815 | 0.37585 |
| 49 | 1 | 0 | 6.291352 | -1.128976 | 3.550903 |
| 50 | 1 | 0 | 3.202066 | 0.959406 | -2.372271 |
| 51 | 1 | 0 | -0.041964 | 1.101104 | -2.940288 |
| 52 | 1 | 0 | 1.579185 | 3.837978 | -1.995636 |
| 53 | 1 | 0 | -10.165686 | -2.69373 | -1.118298 |
| 54 | 1 | 0 | -9.221646 | -0.92843 | 1.507468 |
| 55 | 1 | 0 | -8.523896 | 0.162006 | -1.553817 |
| 56 | 1 | 0 | -7.86275 | -2.689403 | 5.536867 |
| 57 | 1 | 0 | -8.926547 | -5.456608 | 7.016869 |
| 58 | 1 | 0 | -5.744836 | -5.17814 | 6.046128 |
| 59 | 1 | 0 | -11.853055 | -7.370901 | 4.248428 |
| 60 | 1 | 0 | -11.044789 | -7.936155 | 0.914666 |
| 61 | 1 | 0 | 5.057035 | 4.164568 | 0.23761 |
| 62 | 1 | 0 | 8.340087 | 5.882011 | 3.093556 |
| 63 | 1 | 0 | 8.577734 | 2.602132 | 2.771501 |
| 64 | 1 | 0 | 7.268559 | 3.916992 | 5.530967 |
| 65 | 1 | 0 | 1.296232 | 6.220896 | 2.869526 |
| 66 | 1 | 0 | 3.668083 | 6.915488 | 5.020126 |
| 67 | 1 | 0 | 6.070661 | 9.116176 | 1.558342 |
| 68 | 1 | 0 | 3.650076 | 8.365595 | -0.554089 |
| 69 | 1 | 0 | 3.398992 | 11.955326 | 4.007812 |
| 70 | 1 | 0 | 0.243533 | 9.584219 | -1.843403 |
| 71 | 1 | 0 | -2.621622 | 10.733932 | -0.686596 |
| 72 | 1 | 0 | -0.666528 | 12.66612 | -2.575406 |
| 73 | 1 | 0 | 1.401893 | -0.281689 | 5.90631 |
| 74 | 1 | 0 | 4.227777 | 1.213867 | 6.758791 |
| 75 | 1 | 0 | 1.490478 | 3.010461 | 6.250001 |
| 76 | 1 | 0 | -3.816197 | -7.466185 | -6.03715 |
| 77 | 1 | 0 | -2.866347 | 14.749774 | -0.019733 |

| C2000007_en_ | | Standard Orientation (Ångstroms) | | | |
| --- | --- | --- | --- | --- | --- |
| Center number | Atomic number | Atomic Type | X | Y | Z |
| 0 | 6 | 0 | -7.035905 | -4.662172 | -4.85196 |
| 1 | 6 | 0 | -8.112791 | -5.784135 | 3.408695 |
| 2 | 6 | 0 | -6.484554 | -5.77839 | 1.03326 |
| 3 | 6 | 0 | -6.153534 | -3.142163 | -0.254971 |
| 4 | 6 | 0 | -5.156929 | -3.536697 | -2.962367 |
| 5 | 6 | 0 | -3.978518 | -6.980576 | 1.538567 |
| 6 | 6 | 0 | -1.806785 | -5.697558 | 1.672291 |
| 7 | 6 | 0 | -1.664773 | -2.985227 | 1.356711 |
| 8 | 6 | 0 | -4.174422 | -1.630649 | 1.292166 |
| 9 | 6 | 0 | 0.5547 | -1.759008 | 1.245589 |
| 10 | 6 | 0 | 0.841347 | 1.078938 | 1.024516 |
| 11 | 6 | 0 | -1.630882 | 2.363857 | 1.789972 |
| 12 | 6 | 0 | -3.921069 | 1.137079 | 0.559445 |
| 13 | 6 | 0 | 3.121774 | -2.934784 | 1.561533 |
| 14 | 6 | 0 | 4.910224 | -0.71758 | 2.053304 |
| 15 | 6 | 0 | 3.230625 | 1.557664 | 2.775559 |
| 16 | 6 | 0 | 1.42866 | 1.748528 | -1.741037 |
| 17 | 6 | 0 | -8.63923 | -1.69365 | -0.316742 |
| 18 | 6 | 0 | -10.832483 | -6.491162 | 3.080006 |
| 19 | 6 | 0 | -7.158012 | -5.32619 | 5.689523 |
| 20 | 6 | 0 | 4.660567 | 4.062751 | 2.299952 |
| 21 | 6 | 0 | 7.279666 | 4.100068 | 3.516339 |
| 22 | 6 | 0 | 3.191996 | 6.428011 | 3.092857 |
| 23 | 6 | 0 | 3.944077 | 8.801761 | 1.612428 |
| 24 | 6 | 0 | 2.342458 | 10.989425 | 2.328874 |
| 25 | 6 | 0 | 2.968565 | 13.014777 | 3.706936 |
| 26 | 6 | 0 | 5.542629 | 13.484827 | 4.791586 |
| 27 | 1 | 0 | -4.895721 | -1.657724 | 3.230706 |
| 28 | 6 | 0 | 2.486739 | 1.291877 | 5.553915 |
| 29 | 6 | 0 | -5.747524 | -5.223593 | -7.336384 |
| 30 | 8 | 0 | -5.015269 | -7.276089 | -7.982886 |
| 31 | 8 | 0 | -5.352447 | -3.191007 | -8.879802 |
| 32 | 6 | 0 | 0.90804 | 14.896293 | 4.172983 |
| 33 | 8 | 0 | -1.246098 | 14.681854 | 3.450187 |
| 34 | 8 | 0 | 1.556726 | 16.998158 | 5.518995 |
| 35 | 1 | 0 | -7.796692 | -6.440806 | -4.167545 |
| 36 | 1 | 0 | -8.622752 | -3.380329 | -5.150213 |
| 37 | 1 | 0 | -7.484515 | -6.981487 | -0.316275 |
| 38 | 1 | 0 | -4.516431 | -1.716607 | -3.687798 |
| 39 | 1 | 0 | -3.492965 | -4.739577 | -2.896212 |
| 40 | 1 | 0 | -3.984179 | -9.003626 | 1.850536 |
| 41 | 1 | 0 | -0.072987 | -6.728902 | 2.008165 |
| 42 | 1 | 0 | -1.598846 | 4.356731 | 1.276639 |
| 43 | 1 | 0 | -1.86757 | 2.277762 | 3.83144 |
| 44 | 1 | 0 | -5.607944 | 2.164803 | 1.136808 |
| 45 | 1 | 0 | -3.802189 | 1.345628 | -1.485183 |
| 46 | 1 | 0 | 3.134647 | -4.263346 | 3.135149 |
| 47 | 1 | 0 | 3.686312 | -4.01873 | -0.099751 |
| 48 | 1 | 0 | 6.0036 | -0.278981 | 0.364788 |
| 49 | 1 | 0 | 6.270277 | -1.14843 | 3.532435 |
| 50 | 1 | 0 | 3.197941 | 0.931939 | -2.388623 |
| 51 | 1 | 0 | -0.042753 | 1.031454 | -2.980803 |
| 52 | 1 | 0 | 1.532145 | 3.78727 | -2.011021 |
| 53 | 1 | 0 | -10.152317 | -2.81178 | -1.142007 |
| 54 | 1 | 0 | -9.227605 | -1.1505 | 1.577018 |
| 55 | 1 | 0 | -8.470158 | 0.022226 | -1.439733 |
| 56 | 1 | 0 | -10.983846 | -8.407727 | 2.32754 |
| 57 | 1 | 0 | -11.8518 | -6.43137 | 4.86375 |
| 58 | 1 | 0 | -11.808023 | -5.265837 | 1.744923 |
| 59 | 1 | 0 | -8.342644 | -5.339224 | 7.356621 |
| 60 | 1 | 0 | -5.168681 | -4.973563 | 5.982197 |
| 61 | 1 | 0 | 4.967177 | 4.180347 | 0.263342 |
| 62 | 1 | 0 | 8.233161 | 5.885331 | 3.140762 |
| 63 | 1 | 0 | 8.497466 | 2.611228 | 2.795601 |
| 64 | 1 | 0 | 7.169001 | 3.89106 | 5.56024 |
| 65 | 1 | 0 | 1.174785 | 6.145327 | 2.855181 |
| 66 | 1 | 0 | 3.469358 | 6.786593 | 5.105042 |
| 67 | 1 | 0 | 5.94578 | 9.199056 | 1.862075 |
| 68 | 1 | 0 | 3.672881 | 8.422217 | -0.40089 |
| 69 | 1 | 0 | 0.389632 | 10.903328 | 1.712022 |
| 70 | 1 | 0 | 6.834849 | 11.948486 | 4.374918 |
| 71 | 1 | 0 | 6.40504 | 15.202589 | 4.035779 |
| 72 | 1 | 0 | 5.48701 | 13.660641 | 6.847893 |
| 73 | 1 | 0 | 1.363897 | -0.404609 | 5.856367 |
| 74 | 1 | 0 | 4.153824 | 1.136774 | 6.747394 |
| 75 | 1 | 0 | 1.383927 | 2.884085 | 6.236582 |
| 76 | 1 | 0 | -6.053443 | -1.689821 | -8.111594 |
| 77 | 1 | 0 | 3.324593 | 16.941232 | 5.971443 |

| C2000007_tddft_ | | Standard Orientation (Ångstroms) | | | |
| --- | --- | --- | --- | --- | --- |
| Center number | Atomic number | Atomic Type | X | Y | Z |
| 0 | 6 | 0 | -7.035905 | -4.662172 | -4.85196 |
| 1 | 6 | 0 | -8.112791 | -5.784135 | 3.408695 |
| 2 | 6 | 0 | -6.484554 | -5.77839 | 1.03326 |
| 3 | 6 | 0 | -6.153534 | -3.142163 | -0.254971 |
| 4 | 6 | 0 | -5.156929 | -3.536697 | -2.962367 |
| 5 | 6 | 0 | -3.978518 | -6.980576 | 1.538567 |
| 6 | 6 | 0 | -1.806785 | -5.697558 | 1.672291 |
| 7 | 6 | 0 | -1.664773 | -2.985227 | 1.356711 |
| 8 | 6 | 0 | -4.174422 | -1.630649 | 1.292166 |
| 9 | 6 | 0 | 0.5547 | -1.759008 | 1.245589 |
| 10 | 6 | 0 | 0.841347 | 1.078938 | 1.024516 |
| 11 | 6 | 0 | -1.630882 | 2.363857 | 1.789972 |
| 12 | 6 | 0 | -3.921069 | 1.137079 | 0.559445 |
| 13 | 6 | 0 | 3.121774 | -2.934784 | 1.561533 |
| 14 | 6 | 0 | 4.910224 | -0.71758 | 2.053304 |
| 15 | 6 | 0 | 3.230625 | 1.557664 | 2.775559 |
| 16 | 6 | 0 | 1.42866 | 1.748528 | -1.741037 |
| 17 | 6 | 0 | -8.63923 | -1.69365 | -0.316742 |
| 18 | 6 | 0 | -10.832483 | -6.491162 | 3.080006 |
| 19 | 6 | 0 | -7.158012 | -5.32619 | 5.689523 |
| 20 | 6 | 0 | 4.660567 | 4.062751 | 2.299952 |
| 21 | 6 | 0 | 7.279666 | 4.100068 | 3.516339 |
| 22 | 6 | 0 | 3.191996 | 6.428011 | 3.092857 |
| 23 | 6 | 0 | 3.944077 | 8.801761 | 1.612428 |
| 24 | 6 | 0 | 2.342458 | 10.989425 | 2.328874 |
| 25 | 6 | 0 | 2.968565 | 13.014777 | 3.706936 |
| 26 | 6 | 0 | 5.542629 | 13.484827 | 4.791586 |
| 27 | 1 | 0 | -4.895721 | -1.657724 | 3.230706 |
| 28 | 6 | 0 | 2.486739 | 1.291877 | 5.553915 |
| 29 | 6 | 0 | -5.747524 | -5.223593 | -7.336384 |
| 30 | 8 | 0 | -5.015269 | -7.276089 | -7.982886 |
| 31 | 8 | 0 | -5.352447 | -3.191007 | -8.879802 |
| 32 | 6 | 0 | 0.90804 | 14.896293 | 4.172983 |
| 33 | 8 | 0 | -1.246098 | 14.681854 | 3.450187 |
| 34 | 8 | 0 | 1.556726 | 16.998158 | 5.518995 |
| 35 | 1 | 0 | -7.796692 | -6.440806 | -4.167545 |
| 36 | 1 | 0 | -8.622752 | -3.380329 | -5.150213 |
| 37 | 1 | 0 | -7.484515 | -6.981487 | -0.316275 |
| 38 | 1 | 0 | -4.516431 | -1.716607 | -3.687798 |
| 39 | 1 | 0 | -3.492965 | -4.739577 | -2.896212 |
| 40 | 1 | 0 | -3.984179 | -9.003626 | 1.850536 |
| 41 | 1 | 0 | -0.072987 | -6.728902 | 2.008165 |
| 42 | 1 | 0 | -1.598846 | 4.356731 | 1.276639 |
| 43 | 1 | 0 | -1.86757 | 2.277762 | 3.83144 |
| 44 | 1 | 0 | -5.607944 | 2.164803 | 1.136808 |
| 45 | 1 | 0 | -3.802189 | 1.345628 | -1.485183 |
| 46 | 1 | 0 | 3.134647 | -4.263346 | 3.135149 |
| 47 | 1 | 0 | 3.686312 | -4.01873 | -0.099751 |
| 48 | 1 | 0 | 6.0036 | -0.278981 | 0.364788 |
| 49 | 1 | 0 | 6.270277 | -1.14843 | 3.532435 |
| 50 | 1 | 0 | 3.197941 | 0.931939 | -2.388623 |
| 51 | 1 | 0 | -0.042753 | 1.031454 | -2.980803 |
| 52 | 1 | 0 | 1.532145 | 3.78727 | -2.011021 |
| 53 | 1 | 0 | -10.152317 | -2.81178 | -1.142007 |
| 54 | 1 | 0 | -9.227605 | -1.1505 | 1.577018 |
| 55 | 1 | 0 | -8.470158 | 0.022226 | -1.439733 |
| 56 | 1 | 0 | -10.983846 | -8.407727 | 2.32754 |
| 57 | 1 | 0 | -11.8518 | -6.43137 | 4.86375 |
| 58 | 1 | 0 | -11.808023 | -5.265837 | 1.744923 |
| 59 | 1 | 0 | -8.342644 | -5.339224 | 7.356621 |
| 60 | 1 | 0 | -5.168681 | -4.973563 | 5.982197 |
| 61 | 1 | 0 | 4.967177 | 4.180347 | 0.263342 |
| 62 | 1 | 0 | 8.233161 | 5.885331 | 3.140762 |
| 63 | 1 | 0 | 8.497466 | 2.611228 | 2.795601 |
| 64 | 1 | 0 | 7.169001 | 3.89106 | 5.56024 |
| 65 | 1 | 0 | 1.174785 | 6.145327 | 2.855181 |
| 66 | 1 | 0 | 3.469358 | 6.786593 | 5.105042 |
| 67 | 1 | 0 | 5.94578 | 9.199056 | 1.862075 |
| 68 | 1 | 0 | 3.672881 | 8.422217 | -0.40089 |
| 69 | 1 | 0 | 0.389632 | 10.903328 | 1.712022 |
| 70 | 1 | 0 | 6.834849 | 11.948486 | 4.374918 |
| 71 | 1 | 0 | 6.40504 | 15.202589 | 4.035779 |
| 72 | 1 | 0 | 5.48701 | 13.660641 | 6.847893 |
| 73 | 1 | 0 | 1.363897 | -0.404609 | 5.856367 |
| 74 | 1 | 0 | 4.153824 | 1.136774 | 6.747394 |
| 75 | 1 | 0 | 1.383927 | 2.884085 | 6.236582 |
| 76 | 1 | 0 | -6.053443 | -1.689821 | -8.111594 |
| 77 | 1 | 0 | 3.324593 | 16.941232 | 5.971443 |

| C2000008_en_ | | Standard Orientation (Ångstroms) | | | |
| --- | --- | --- | --- | --- | --- |
| Center number | Atomic number | Atomic Type | X | Y | Z |
| 0 | 6 | 0 | -5.916927 | -3.871076 | -5.364435 |
| 1 | 6 | 0 | -8.255211 | -5.729218 | 2.360672 |
| 2 | 6 | 0 | -6.150971 | -5.438204 | 0.426156 |
| 3 | 6 | 0 | -5.655432 | -2.723709 | -0.593519 |
| 4 | 6 | 0 | -4.29519 | -2.965167 | -3.163887 |
| 5 | 6 | 0 | -3.776253 | -6.655575 | 1.368032 |
| 6 | 6 | 0 | -1.637225 | -5.400142 | 1.854639 |
| 7 | 6 | 0 | -1.423004 | -2.686137 | 1.610223 |
| 8 | 6 | 0 | -3.894846 | -1.301586 | 1.275285 |
| 9 | 6 | 0 | 0.804757 | -1.485734 | 1.790116 |
| 10 | 6 | 0 | 1.132513 | 1.350099 | 1.637848 |
| 11 | 6 | 0 | -1.385595 | 2.64831 | 2.207009 |
| 12 | 6 | 0 | -3.553353 | 1.498392 | 0.707992 |
| 13 | 6 | 0 | 3.314366 | -2.690957 | 2.360897 |
| 14 | 6 | 0 | 5.07951 | -0.488212 | 2.981939 |
| 15 | 6 | 0 | 3.361081 | 1.788106 | 3.595199 |
| 16 | 6 | 0 | 1.961313 | 2.038035 | -1.061908 |
| 17 | 6 | 0 | -8.137323 | -1.300597 | -0.917578 |
| 18 | 6 | 0 | -7.909655 | -4.669915 | 4.961631 |
| 19 | 6 | 0 | -10.330202 | -7.014545 | 1.754868 |
| 20 | 6 | 0 | 4.82472 | 4.300765 | 3.288574 |
| 21 | 6 | 0 | 7.394664 | 4.262152 | 4.605516 |
| 22 | 6 | 0 | 3.347903 | 6.640255 | 4.142881 |
| 23 | 6 | 0 | 4.161843 | 9.082003 | 2.798366 |
| 24 | 6 | 0 | 3.306115 | 9.08581 | 0.1287 |
| 25 | 6 | 0 | 4.681343 | 8.912814 | -1.985363 |
| 26 | 6 | 0 | 7.507972 | 8.774631 | -2.064119 |
| 27 | 1 | 0 | -4.832429 | -1.410304 | 3.111365 |
| 28 | 6 | 0 | 2.39083 | 1.473898 | 6.297958 |
| 29 | 6 | 0 | -4.512428 | -4.596273 | -7.751778 |
| 30 | 8 | 0 | -5.223909 | -6.211504 | -9.187883 |
| 31 | 8 | 0 | -2.371477 | -3.278071 | -8.29565 |
| 32 | 6 | 0 | 3.230846 | 8.807045 | -4.411181 |
| 33 | 8 | 0 | 0.959799 | 8.619728 | -4.562919 |
| 34 | 8 | 0 | 4.611614 | 8.903666 | -6.582745 |
| 35 | 1 | 0 | -7.034436 | -5.51971 | -4.867545 |
| 36 | 1 | 0 | -7.272083 | -2.412788 | -5.906408 |
| 37 | 1 | 0 | -6.785187 | -6.560639 | -1.184312 |
| 38 | 1 | 0 | -3.525723 | -1.103601 | -3.619511 |
| 39 | 1 | 0 | -2.686097 | -4.223424 | -2.926551 |
| 40 | 1 | 0 | -3.848894 | -8.6847 | 1.635926 |
| 41 | 1 | 0 | 0.015818 | -6.45551 | 2.437494 |
| 42 | 1 | 0 | -1.280826 | 4.65655 | 1.772265 |
| 43 | 1 | 0 | -1.819035 | 2.486339 | 4.212628 |
| 44 | 1 | 0 | -5.290168 | 2.507341 | 1.159341 |
| 45 | 1 | 0 | -3.232412 | 1.815501 | -1.298768 |
| 46 | 1 | 0 | 3.161186 | -3.988333 | 3.954322 |
| 47 | 1 | 0 | 4.016007 | -3.81377 | 0.779997 |
| 48 | 1 | 0 | 6.280874 | -0.046639 | 1.370383 |
| 49 | 1 | 0 | 6.338794 | -0.930002 | 4.545162 |
| 50 | 1 | 0 | 3.783299 | 1.224542 | -1.547831 |
| 51 | 1 | 0 | 0.608358 | 1.317467 | -2.426735 |
| 52 | 1 | 0 | 2.070559 | 4.071734 | -1.324224 |
| 53 | 1 | 0 | -9.541895 | -2.451218 | -1.87942 |
| 54 | 1 | 0 | -8.940657 | -0.765949 | 0.898272 |
| 55 | 1 | 0 | -7.874371 | 0.420362 | -2.013513 |
| 56 | 1 | 0 | -8.118487 | -2.618865 | 4.982195 |
| 57 | 1 | 0 | -9.303707 | -5.436571 | 6.262724 |
| 58 | 1 | 0 | -6.031955 | -5.085289 | 5.693902 |
| 59 | 1 | 0 | -11.836725 | -7.325964 | 3.103302 |
| 60 | 1 | 0 | -10.60386 | -7.803664 | -0.112458 |
| 61 | 1 | 0 | 5.20486 | 4.509911 | 1.274082 |
| 62 | 1 | 0 | 8.343713 | 6.079255 | 4.413802 |
| 63 | 1 | 0 | 8.653873 | 2.843014 | 3.818424 |
| 64 | 1 | 0 | 7.213824 | 3.894712 | 6.622563 |
| 65 | 1 | 0 | 1.336296 | 6.385453 | 3.826258 |
| 66 | 1 | 0 | 3.576829 | 6.899266 | 6.175926 |
| 67 | 1 | 0 | 3.279905 | 10.678218 | 3.767777 |
| 68 | 1 | 0 | 6.193474 | 9.350106 | 2.968313 |
| 69 | 1 | 0 | 1.27445 | 9.146205 | -0.139323 |
| 70 | 1 | 0 | 8.319946 | 8.726473 | -0.182997 |
| 71 | 1 | 0 | 8.159608 | 7.074655 | -3.03541 |
| 72 | 1 | 0 | 8.34262 | 10.405892 | -3.019342 |
| 73 | 1 | 0 | 1.297517 | -0.257085 | 6.493183 |
| 74 | 1 | 0 | 3.956917 | 1.364108 | 7.626359 |
| 75 | 1 | 0 | 1.187341 | 3.026121 | 6.897179 |
| 76 | 1 | 0 | -2.012406 | -2.090551 | -6.953081 |
| 77 | 1 | 0 | 6.388313 | 9.080306 | -6.200431 |

| C2000009_en_ | | Standard Orientation (Ångstroms) | | | |
| --- | --- | --- | --- | --- | --- |
| Center number | Atomic number | Atomic Type | X | Y | Z |
| 0 | 6 | 0 | -6.863469 | -4.38901 | -4.960648 |
| 1 | 6 | 0 | -8.288394 | -6.230447 | 2.972719 |
| 2 | 6 | 0 | -6.455217 | -5.864737 | 0.789524 |
| 3 | 6 | 0 | -6.157367 | -3.128166 | -0.24301 |
| 4 | 6 | 0 | -5.108844 | -3.259287 | -2.959692 |
| 5 | 6 | 0 | -3.952599 | -7.034043 | 1.403065 |
| 6 | 6 | 0 | -1.806907 | -5.722163 | 1.663672 |
| 7 | 6 | 0 | -1.694645 | -2.99975 | 1.445094 |
| 8 | 6 | 0 | -4.222486 | -1.679442 | 1.421964 |
| 9 | 6 | 0 | 0.511585 | -1.7457 | 1.381839 |
| 10 | 6 | 0 | 0.758353 | 1.099969 | 1.220945 |
| 11 | 6 | 0 | -1.712688 | 2.313916 | 2.108056 |
| 12 | 6 | 0 | -4.014371 | 1.132296 | 0.854911 |
| 13 | 6 | 0 | 3.089862 | -2.902515 | 1.658043 |
| 14 | 6 | 0 | 4.870184 | -0.677632 | 2.134843 |
| 15 | 6 | 0 | 3.193754 | 1.589173 | 2.90111 |
| 16 | 6 | 0 | 1.252948 | 1.831757 | -1.546399 |
| 17 | 6 | 0 | -8.699964 | -1.777786 | -0.236838 |
| 18 | 6 | 0 | -7.621734 | -5.232524 | 5.534467 |
| 19 | 6 | 0 | -10.414917 | -7.523866 | 2.609825 |
| 20 | 6 | 0 | 4.612117 | 4.091421 | 2.361743 |
| 21 | 6 | 0 | 7.189139 | 4.209479 | 3.656271 |
| 22 | 6 | 0 | 3.091839 | 6.470998 | 2.972348 |
| 23 | 6 | 0 | 4.177076 | 8.929716 | 1.868343 |
| 24 | 6 | 0 | 4.625321 | 8.751126 | -0.904562 |
| 25 | 6 | 0 | 2.928703 | 9.078893 | -2.750428 |
| 26 | 6 | 0 | 0.219049 | 9.776323 | -2.311486 |
| 27 | 1 | 0 | -4.938763 | -1.821513 | 3.353343 |
| 28 | 6 | 0 | 2.540076 | 1.323814 | 5.701473 |
| 29 | 6 | 0 | -5.605684 | -4.407049 | -7.522955 |
| 30 | 8 | 0 | -6.093901 | -2.971373 | -9.217762 |
| 31 | 8 | 0 | -3.753359 | -6.172684 | -7.847252 |
| 32 | 6 | 0 | 3.850295 | 8.669092 | -5.39317 |
| 33 | 8 | 0 | 5.996897 | 8.134945 | -5.95462 |
| 34 | 8 | 0 | 2.104241 | 8.915828 | -7.271616 |
| 35 | 1 | 0 | -7.420976 | -6.31165 | -4.461361 |
| 36 | 1 | 0 | -8.575016 | -3.275113 | -5.143524 |
| 37 | 1 | 0 | -7.274491 | -6.978777 | -0.741056 |
| 38 | 1 | 0 | -4.641148 | -1.352598 | -3.577924 |
| 39 | 1 | 0 | -3.328425 | -4.291406 | -2.927554 |
| 40 | 1 | 0 | -3.938328 | -9.067329 | 1.653374 |
| 41 | 1 | 0 | -0.070272 | -6.737659 | 2.036147 |
| 42 | 1 | 0 | -1.718163 | 4.334127 | 1.718186 |
| 43 | 1 | 0 | -1.898994 | 2.106047 | 4.14659 |
| 44 | 1 | 0 | -5.70785 | 2.092176 | 1.526421 |
| 45 | 1 | 0 | -3.944863 | 1.46969 | -1.171907 |
| 46 | 1 | 0 | 3.128049 | -4.236357 | 3.227964 |
| 47 | 1 | 0 | 3.641319 | -3.97725 | -0.013359 |
| 48 | 1 | 0 | 5.934648 | -0.22341 | 0.432561 |
| 49 | 1 | 0 | 6.25366 | -1.115339 | 3.588952 |
| 50 | 1 | 0 | 2.98439 | 1.004195 | -2.276746 |
| 51 | 1 | 0 | -0.270331 | 1.169649 | -2.753013 |
| 52 | 1 | 0 | 1.397432 | 3.870879 | -1.772426 |
| 53 | 1 | 0 | -10.185236 | -2.965242 | -1.016433 |
| 54 | 1 | 0 | -9.279479 | -1.275979 | 1.6712 |
| 55 | 1 | 0 | -8.631197 | -0.0484 | -1.349887 |
| 56 | 1 | 0 | -7.849363 | -3.186317 | 5.63953 |
| 57 | 1 | 0 | -8.829531 | -6.052938 | 6.980738 |
| 58 | 1 | 0 | -5.661931 | -5.638038 | 6.012944 |
| 59 | 1 | 0 | -11.731687 | -7.880964 | 4.133991 |
| 60 | 1 | 0 | -10.925618 | -8.270582 | 0.774831 |
| 61 | 1 | 0 | 4.976335 | 4.103416 | 0.333485 |
| 62 | 1 | 0 | 8.243989 | 5.865324 | 3.043459 |
| 63 | 1 | 0 | 8.353809 | 2.576387 | 3.217874 |
| 64 | 1 | 0 | 7.008919 | 4.327468 | 5.703966 |
| 65 | 1 | 0 | 1.178498 | 6.275842 | 2.262072 |
| 66 | 1 | 0 | 2.926477 | 6.699085 | 5.015869 |
| 67 | 1 | 0 | 2.866944 | 10.46017 | 2.30524 |
| 68 | 1 | 0 | 5.945003 | 9.406578 | 2.807177 |
| 69 | 1 | 0 | 6.507118 | 8.223727 | -1.514395 |
| 70 | 1 | 0 | -0.171032 | 10.084969 | -0.321903 |
| 71 | 1 | 0 | -0.303981 | 11.515246 | -3.294129 |
| 72 | 1 | 0 | -1.071461 | 8.289802 | -2.938025 |
| 73 | 1 | 0 | 1.439509 | -0.380497 | 6.045474 |
| 74 | 1 | 0 | 4.247634 | 1.183248 | 6.837802 |
| 75 | 1 | 0 | 1.452134 | 2.911912 | 6.419357 |
| 76 | 1 | 0 | -3.565579 | -7.158031 | -6.320045 |
| 77 | 1 | 0 | 0.465635 | 9.316791 | -6.573925 |

Table S5.Gibbs free energies^a^ and equilibrium populations^b^ of low-energy conformers of compound **3**.

| Conformers | ∆G | P(%)/100 | Single point energy(a.u.) |
| --- | --- | --- | --- |
| C3000001_tddft_ | 0.00039 | 22.06 | -1471.7577972697 |
| C3000002_tddft_ | 0.00198 | 4.09 | -1471.7562062166 |
| C3000003_tddft_ | 0.00267 | 1.96 | -1471.7555150338 |
| C3000004_tddft_ | 0.00181 | 4.9 | -1471.7563782236 |
| C3000005_tddft_ | 0.0 | 33.24 | -1471.7581843883 |
| C3000006_tddft_ | 0.00153 | 6.55 | -1471.7566516118 |
| C3000007_tddft_ | 0.00412 | 0.42 | -1471.7540643251 |
| C3000008_tddft_ | 0.00398 | 0.49 | -1471.7542046211 |
| C3000009_tddft_ | 0.00022 | 26.29 | -1471.757963042 |

^a^PBE0-D3(BJ)/def2-TZVP, in kcal/mol.
^b^From ∆G values at 298.15K.

Table S6.Cartesian coordinates for the low-energy reoptimized random reseach conformers of compound **3** at PBE0-D3(BJ)/def2-SVP level of theory in methanol.

| C3000001_en_ | | Standard Orientation (Ångstroms) | | | |
| --- | --- | --- | --- | --- | --- |
| Center number | Atomic number | Atomic Type | X | Y | Z |
| 0 | 6 | 0 | 1.278368 | -8.679472 | 3.886843 |
| 1 | 6 | 0 | -4.875699 | -7.310967 | -3.622741 |
| 2 | 6 | 0 | -4.274323 | -7.703483 | -0.85108 |
| 3 | 6 | 0 | -1.44936 | -7.595787 | -0.075277 |
| 4 | 6 | 0 | -1.319127 | -8.59228 | 2.687411 |
| 5 | 6 | 0 | -5.9657 | -5.952529 | 0.791215 |
| 6 | 6 | 0 | -4.603533 | -3.670663 | 1.928045 |
| 7 | 6 | 0 | -2.376595 | -2.80304 | 0.330819 |
| 8 | 6 | 0 | -0.491274 | -4.895989 | -0.152068 |
| 9 | 6 | 0 | -1.05637 | -0.411186 | 1.295532 |
| 10 | 6 | 0 | 1.147379 | 0.32434 | -0.548846 |
| 11 | 6 | 0 | 3.045229 | -1.797326 | -0.297936 |
| 12 | 6 | 0 | 1.956722 | -4.345568 | -0.553569 |
| 13 | 6 | 0 | -2.640633 | 2.00033 | 1.28813 |
| 14 | 6 | 0 | -0.694841 | 4.155906 | 1.137079 |
| 15 | 6 | 0 | 1.889992 | 2.992868 | 0.370827 |
| 16 | 6 | 0 | 0.280874 | 0.43153 | -3.313472 |
| 17 | 6 | 0 | -0.07282 | -0.819465 | 3.991441 |
| 18 | 6 | 0 | 0.081175 | -9.401586 | -1.732613 |
| 19 | 6 | 0 | -7.235163 | -8.626032 | -4.474467 |
| 20 | 6 | 0 | -3.523315 | -5.940322 | -5.234552 |
| 21 | 6 | 0 | 3.364876 | 4.721851 | -1.428042 |
| 22 | 6 | 0 | 5.884853 | 3.65119 | -2.312456 |
| 23 | 6 | 0 | 3.844323 | 7.268781 | -0.158386 |
| 24 | 6 | 0 | 4.678865 | 9.350673 | -1.932438 |
| 25 | 6 | 0 | 6.354485 | 11.359377 | -0.786948 |
| 26 | 6 | 0 | 6.583917 | 13.804872 | -2.280095 |
| 27 | 6 | 0 | 4.103802 | 15.262589 | -2.266733 |
| 28 | 6 | 0 | 8.73834 | 15.416823 | -1.261129 |
| 29 | 1 | 0 | 3.064506 | 2.75328 | 2.046205 |
| 30 | 8 | 0 | 5.293503 | -1.53164 | 0.172406 |
| 31 | 8 | 0 | 4.027387 | 9.402979 | -4.136672 |
| 32 | 1 | 0 | -3.176742 | -2.337593 | -1.518366 |
| 33 | 6 | 0 | 1.171353 | -9.241092 | 6.688234 |
| 34 | 8 | 0 | -0.728561 | -9.518042 | 7.906682 |
| 35 | 8 | 0 | 3.461347 | -9.416108 | 7.853676 |
| 36 | 1 | 0 | 2.482263 | -10.096971 | 2.995038 |
| 37 | 1 | 0 | 2.25655 | -6.879039 | 3.641349 |
| 38 | 1 | 0 | -4.860754 | -9.640897 | -0.440193 |
| 39 | 1 | 0 | -2.121934 | -10.490708 | 2.690508 |
| 40 | 1 | 0 | -2.529262 | -7.485832 | 3.910477 |
| 41 | 1 | 0 | -6.829116 | -7.045318 | 2.305792 |
| 42 | 1 | 0 | -7.521305 | -5.281033 | -0.374657 |
| 43 | 1 | 0 | -3.979599 | -4.096669 | 3.83767 |
| 44 | 1 | 0 | -5.933145 | -2.109964 | 2.113697 |
| 45 | 1 | 0 | 3.336955 | -5.815038 | -0.88595 |
| 46 | 1 | 0 | -3.900314 | 2.029417 | -0.340059 |
| 47 | 1 | 0 | -3.833899 | 2.144848 | 2.957793 |
| 48 | 1 | 0 | -0.551406 | 5.151614 | 2.930536 |
| 49 | 1 | 0 | -1.285759 | 5.550301 | -0.257731 |
| 50 | 1 | 0 | -0.340378 | -1.388653 | -4.016862 |
| 51 | 1 | 0 | -1.254478 | 1.771352 | -3.571672 |
| 52 | 1 | 0 | 1.825385 | 1.035963 | -4.520008 |
| 53 | 1 | 0 | 0.917619 | 0.832222 | 4.696032 |
| 54 | 1 | 0 | -1.643864 | -1.162104 | 5.269988 |
| 55 | 1 | 0 | 1.210803 | -2.414611 | 4.1423 |
| 56 | 1 | 0 | 2.020204 | -9.599455 | -1.089532 |
| 57 | 1 | 0 | -0.773296 | -11.274456 | -1.669624 |
| 58 | 1 | 0 | 0.129268 | -8.802002 | -3.689395 |
| 59 | 1 | 0 | -6.993246 | -10.673432 | -4.367802 |
| 60 | 1 | 0 | -8.845831 | -8.170737 | -3.270056 |
| 61 | 1 | 0 | -7.728866 | -8.142224 | -6.409034 |
| 62 | 1 | 0 | -4.107548 | -5.712407 | -7.180353 |
| 63 | 1 | 0 | -1.774821 | -5.033426 | -4.709538 |
| 64 | 1 | 0 | 2.198558 | 5.101133 | -3.085575 |
| 65 | 1 | 0 | 6.837504 | 4.996518 | -3.544704 |
| 66 | 1 | 0 | 5.673719 | 1.898607 | -3.355665 |
| 67 | 1 | 0 | 7.109651 | 3.2366 | -0.712811 |
| 68 | 1 | 0 | 2.127481 | 7.997902 | 0.733582 |
| 69 | 1 | 0 | 5.212576 | 7.06547 | 1.375071 |
| 70 | 1 | 0 | 5.729487 | 11.734444 | 1.148843 |
| 71 | 1 | 0 | 8.211098 | 10.4637 | -0.571815 |
| 72 | 1 | 0 | 7.010437 | 13.293162 | -4.232799 |
| 73 | 1 | 0 | 4.261202 | 16.996065 | -3.366928 |
| 74 | 1 | 0 | 2.574485 | 14.149171 | -3.070186 |
| 75 | 1 | 0 | 3.576994 | 15.794201 | -0.34321 |
| 76 | 1 | 0 | 8.934374 | 17.155692 | -2.345963 |
| 77 | 1 | 0 | 10.539552 | 14.420024 | -1.342166 |
| 78 | 1 | 0 | 8.404427 | 15.948256 | 0.703915 |
| 79 | 1 | 0 | 4.798447 | -9.112998 | 6.644855 |

| C3000001_tddft_ | | Standard Orientation (Ångstroms) | | | |
| --- | --- | --- | --- | --- | --- |
| Center number | Atomic number | Atomic Type | X | Y | Z |
| 0 | 6 | 0 | 1.278368 | -8.679472 | 3.886843 |
| 1 | 6 | 0 | -4.875699 | -7.310967 | -3.622741 |
| 2 | 6 | 0 | -4.274323 | -7.703483 | -0.85108 |
| 3 | 6 | 0 | -1.44936 | -7.595787 | -0.075277 |
| 4 | 6 | 0 | -1.319127 | -8.59228 | 2.687411 |
| 5 | 6 | 0 | -5.9657 | -5.952529 | 0.791215 |
| 6 | 6 | 0 | -4.603533 | -3.670663 | 1.928045 |
| 7 | 6 | 0 | -2.376595 | -2.80304 | 0.330819 |
| 8 | 6 | 0 | -0.491274 | -4.895989 | -0.152068 |
| 9 | 6 | 0 | -1.05637 | -0.411186 | 1.295532 |
| 10 | 6 | 0 | 1.147379 | 0.32434 | -0.548846 |
| 11 | 6 | 0 | 3.045229 | -1.797326 | -0.297936 |
| 12 | 6 | 0 | 1.956722 | -4.345568 | -0.553569 |
| 13 | 6 | 0 | -2.640633 | 2.00033 | 1.28813 |
| 14 | 6 | 0 | -0.694841 | 4.155906 | 1.137079 |
| 15 | 6 | 0 | 1.889992 | 2.992868 | 0.370827 |
| 16 | 6 | 0 | 0.280874 | 0.43153 | -3.313472 |
| 17 | 6 | 0 | -0.07282 | -0.819465 | 3.991441 |
| 18 | 6 | 0 | 0.081175 | -9.401586 | -1.732613 |
| 19 | 6 | 0 | -7.235163 | -8.626032 | -4.474467 |
| 20 | 6 | 0 | -3.523315 | -5.940322 | -5.234552 |
| 21 | 6 | 0 | 3.364876 | 4.721851 | -1.428042 |
| 22 | 6 | 0 | 5.884853 | 3.65119 | -2.312456 |
| 23 | 6 | 0 | 3.844323 | 7.268781 | -0.158386 |
| 24 | 6 | 0 | 4.678865 | 9.350673 | -1.932438 |
| 25 | 6 | 0 | 6.354485 | 11.359377 | -0.786948 |
| 26 | 6 | 0 | 6.583917 | 13.804872 | -2.280095 |
| 27 | 6 | 0 | 4.103802 | 15.262589 | -2.266733 |
| 28 | 6 | 0 | 8.73834 | 15.416823 | -1.261129 |
| 29 | 1 | 0 | 3.064506 | 2.75328 | 2.046205 |
| 30 | 8 | 0 | 5.293503 | -1.53164 | 0.172406 |
| 31 | 8 | 0 | 4.027387 | 9.402979 | -4.136672 |
| 32 | 1 | 0 | -3.176742 | -2.337593 | -1.518366 |
| 33 | 6 | 0 | 1.171353 | -9.241092 | 6.688234 |
| 34 | 8 | 0 | -0.728561 | -9.518042 | 7.906682 |
| 35 | 8 | 0 | 3.461347 | -9.416108 | 7.853676 |
| 36 | 1 | 0 | 2.482263 | -10.096971 | 2.995038 |
| 37 | 1 | 0 | 2.25655 | -6.879039 | 3.641349 |
| 38 | 1 | 0 | -4.860754 | -9.640897 | -0.440193 |
| 39 | 1 | 0 | -2.121934 | -10.490708 | 2.690508 |
| 40 | 1 | 0 | -2.529262 | -7.485832 | 3.910477 |
| 41 | 1 | 0 | -6.829116 | -7.045318 | 2.305792 |
| 42 | 1 | 0 | -7.521305 | -5.281033 | -0.374657 |
| 43 | 1 | 0 | -3.979599 | -4.096669 | 3.83767 |
| 44 | 1 | 0 | -5.933145 | -2.109964 | 2.113697 |
| 45 | 1 | 0 | 3.336955 | -5.815038 | -0.88595 |
| 46 | 1 | 0 | -3.900314 | 2.029417 | -0.340059 |
| 47 | 1 | 0 | -3.833899 | 2.144848 | 2.957793 |
| 48 | 1 | 0 | -0.551406 | 5.151614 | 2.930536 |
| 49 | 1 | 0 | -1.285759 | 5.550301 | -0.257731 |
| 50 | 1 | 0 | -0.340378 | -1.388653 | -4.016862 |
| 51 | 1 | 0 | -1.254478 | 1.771352 | -3.571672 |
| 52 | 1 | 0 | 1.825385 | 1.035963 | -4.520008 |
| 53 | 1 | 0 | 0.917619 | 0.832222 | 4.696032 |
| 54 | 1 | 0 | -1.643864 | -1.162104 | 5.269988 |
| 55 | 1 | 0 | 1.210803 | -2.414611 | 4.1423 |
| 56 | 1 | 0 | 2.020204 | -9.599455 | -1.089532 |
| 57 | 1 | 0 | -0.773296 | -11.274456 | -1.669624 |
| 58 | 1 | 0 | 0.129268 | -8.802002 | -3.689395 |
| 59 | 1 | 0 | -6.993246 | -10.673432 | -4.367802 |
| 60 | 1 | 0 | -8.845831 | -8.170737 | -3.270056 |
| 61 | 1 | 0 | -7.728866 | -8.142224 | -6.409034 |
| 62 | 1 | 0 | -4.107548 | -5.712407 | -7.180353 |
| 63 | 1 | 0 | -1.774821 | -5.033426 | -4.709538 |
| 64 | 1 | 0 | 2.198558 | 5.101133 | -3.085575 |
| 65 | 1 | 0 | 6.837504 | 4.996518 | -3.544704 |
| 66 | 1 | 0 | 5.673719 | 1.898607 | -3.355665 |
| 67 | 1 | 0 | 7.109651 | 3.2366 | -0.712811 |
| 68 | 1 | 0 | 2.127481 | 7.997902 | 0.733582 |
| 69 | 1 | 0 | 5.212576 | 7.06547 | 1.375071 |
| 70 | 1 | 0 | 5.729487 | 11.734444 | 1.148843 |
| 71 | 1 | 0 | 8.211098 | 10.4637 | -0.571815 |
| 72 | 1 | 0 | 7.010437 | 13.293162 | -4.232799 |
| 73 | 1 | 0 | 4.261202 | 16.996065 | -3.366928 |
| 74 | 1 | 0 | 2.574485 | 14.149171 | -3.070186 |
| 75 | 1 | 0 | 3.576994 | 15.794201 | -0.34321 |
| 76 | 1 | 0 | 8.934374 | 17.155692 | -2.345963 |
| 77 | 1 | 0 | 10.539552 | 14.420024 | -1.342166 |
| 78 | 1 | 0 | 8.404427 | 15.948256 | 0.703915 |
| 79 | 1 | 0 | 4.798447 | -9.112998 | 6.644855 |

| C3000002_en_ | | Standard Orientation (Ångstroms) | | | |
| --- | --- | --- | --- | --- | --- |
| Center number | Atomic number | Atomic Type | X | Y | Z |
| 0 | 6 | 0 | 2.156712 | -8.561752 | 3.50337 |
| 1 | 6 | 0 | -4.24971 | -7.968827 | -3.89129 |
| 2 | 6 | 0 | -3.554536 | -8.378898 | -1.144371 |
| 3 | 6 | 0 | -0.780391 | -7.816881 | -0.384903 |
| 4 | 6 | 0 | -0.431573 | -8.904339 | 2.326942 |
| 5 | 6 | 0 | -5.504739 | -7.022414 | 0.58248 |
| 6 | 6 | 0 | -4.538425 | -4.611762 | 1.850893 |
| 7 | 6 | 0 | -2.505661 | -3.283649 | 0.313505 |
| 8 | 6 | 0 | -0.307448 | -4.992398 | -0.329619 |
| 9 | 6 | 0 | -1.573898 | -0.788706 | 1.454745 |
| 10 | 6 | 0 | 0.434492 | 0.449018 | -0.351241 |
| 11 | 6 | 0 | 2.658466 | -1.343787 | -0.289922 |
| 12 | 6 | 0 | 2.001916 | -4.01049 | -0.730666 |
| 13 | 6 | 0 | -3.524915 | 1.322032 | 1.676757 |
| 14 | 6 | 0 | -1.959127 | 3.771216 | 1.683589 |
| 15 | 6 | 0 | 0.755683 | 3.117433 | 0.78276 |
| 16 | 6 | 0 | -0.496539 | 0.641841 | -3.089912 |
| 17 | 6 | 0 | -0.459636 | -1.253657 | 4.092367 |
| 18 | 6 | 0 | 1.014657 | -9.246252 | -2.141913 |
| 19 | 6 | 0 | -6.321849 | -9.67018 | -4.803063 |
| 20 | 6 | 0 | -3.217416 | -6.27496 | -5.430823 |
| 21 | 6 | 0 | 1.910409 | 5.221553 | -0.84798 |
| 22 | 6 | 0 | 4.539861 | 4.621505 | -1.851361 |
| 23 | 6 | 0 | 2.041063 | 7.683417 | 0.68663 |
| 24 | 6 | 0 | -0.341638 | 9.268544 | 0.759108 |
| 25 | 6 | 0 | -0.943972 | 10.514449 | 3.255101 |
| 26 | 6 | 0 | -3.390124 | 12.019093 | 3.372874 |
| 27 | 6 | 0 | -5.685781 | 10.286997 | 3.29358 |
| 28 | 6 | 0 | -3.433258 | 13.683159 | 5.720192 |
| 29 | 1 | 0 | 1.996882 | 2.927719 | 2.416157 |
| 30 | 8 | 0 | 4.841183 | -0.749794 | 0.191026 |
| 31 | 8 | 0 | -1.596584 | 9.62247 | -1.136111 |
| 32 | 1 | 0 | -3.398866 | -2.827065 | -1.494474 |
| 33 | 6 | 0 | 2.159947 | -9.047411 | 6.320863 |
| 34 | 8 | 0 | 0.319157 | -9.459289 | 7.591228 |
| 35 | 8 | 0 | 4.477636 | -8.960678 | 7.438492 |
| 36 | 1 | 0 | 3.563372 | -9.789226 | 2.626967 |
| 37 | 1 | 0 | 2.829826 | -6.633099 | 3.207485 |
| 38 | 1 | 0 | -3.788172 | -10.405416 | -0.819112 |
| 39 | 1 | 0 | -0.872023 | -10.916278 | 2.240475 |
| 40 | 1 | 0 | -1.805054 | -8.09921 | 3.611419 |
| 41 | 1 | 0 | -6.171352 | -8.320565 | 2.033147 |
| 42 | 1 | 0 | -7.155597 | -6.558041 | -0.553305 |
| 43 | 1 | 0 | -3.833416 | -5.031566 | 3.732091 |
| 44 | 1 | 0 | -6.112551 | -3.313783 | 2.124026 |
| 45 | 1 | 0 | 3.600918 | -5.201485 | -1.171581 |
| 46 | 1 | 0 | -4.816892 | 1.289201 | 0.073924 |
| 47 | 1 | 0 | -4.680494 | 1.127396 | 3.367039 |
| 48 | 1 | 0 | -1.924153 | 4.628476 | 3.552616 |
| 49 | 1 | 0 | -2.816794 | 5.148898 | 0.429455 |
| 50 | 1 | 0 | -0.893303 | -1.185011 | -3.926274 |
| 51 | 1 | 0 | -2.191668 | 1.792246 | -3.231737 |
| 52 | 1 | 0 | 0.936865 | 1.529008 | -4.259642 |
| 53 | 1 | 0 | 0.377805 | 0.446142 | 4.876382 |
| 54 | 1 | 0 | -1.934765 | -1.841498 | 5.395637 |
| 55 | 1 | 0 | 0.989248 | -2.706643 | 4.096135 |
| 56 | 1 | 0 | 2.9645 | -9.162314 | -1.504715 |
| 57 | 1 | 0 | 0.478957 | -11.234099 | -2.19338 |
| 58 | 1 | 0 | 0.952821 | -8.530858 | -4.059589 |
| 59 | 1 | 0 | -5.686111 | -11.634006 | -4.814801 |
| 60 | 1 | 0 | -7.966877 | -9.602711 | -3.561107 |
| 61 | 1 | 0 | -6.935339 | -9.186547 | -6.703118 |
| 62 | 1 | 0 | -3.868631 | -6.068848 | -7.358079 |
| 63 | 1 | 0 | -1.68198 | -5.056601 | -4.870183 |
| 64 | 1 | 0 | 0.661132 | 5.592026 | -2.445863 |
| 65 | 1 | 0 | 5.223807 | 6.180525 | -3.011726 |
| 66 | 1 | 0 | 4.580551 | 2.922016 | -2.99786 |
| 67 | 1 | 0 | 5.87428 | 4.320615 | -0.315599 |
| 68 | 1 | 0 | 2.730346 | 7.309321 | 2.594099 |
| 69 | 1 | 0 | 3.447397 | 8.924652 | -0.183042 |
| 70 | 1 | 0 | -0.914832 | 9.059517 | 4.725127 |
| 71 | 1 | 0 | 0.680667 | 11.72145 | 3.699271 |
| 72 | 1 | 0 | -3.450849 | 13.233218 | 1.704849 |
| 73 | 1 | 0 | -7.432164 | 11.37453 | 3.378455 |
| 74 | 1 | 0 | -5.722056 | 9.170738 | 1.570698 |
| 75 | 1 | 0 | -5.679307 | 8.992878 | 4.901019 |
| 76 | 1 | 0 | -5.159736 | 14.802198 | 5.803277 |
| 77 | 1 | 0 | -1.838193 | 14.987035 | 5.756828 |
| 78 | 1 | 0 | -3.351159 | 12.546085 | 7.439445 |
| 79 | 1 | 0 | 5.754787 | -8.574752 | 6.189099 |

| C3000002_tddft_ | | Standard Orientation (Ångstroms) | | | |
| --- | --- | --- | --- | --- | --- |
| Center number | Atomic number | Atomic Type | X | Y | Z |
| 0 | 6 | 0 | 2.156712 | -8.561752 | 3.50337 |
| 1 | 6 | 0 | -4.24971 | -7.968827 | -3.89129 |
| 2 | 6 | 0 | -3.554536 | -8.378898 | -1.144371 |
| 3 | 6 | 0 | -0.780391 | -7.816881 | -0.384903 |
| 4 | 6 | 0 | -0.431573 | -8.904339 | 2.326942 |
| 5 | 6 | 0 | -5.504739 | -7.022414 | 0.58248 |
| 6 | 6 | 0 | -4.538425 | -4.611762 | 1.850893 |
| 7 | 6 | 0 | -2.505661 | -3.283649 | 0.313505 |
| 8 | 6 | 0 | -0.307448 | -4.992398 | -0.329619 |
| 9 | 6 | 0 | -1.573898 | -0.788706 | 1.454745 |
| 10 | 6 | 0 | 0.434492 | 0.449018 | -0.351241 |
| 11 | 6 | 0 | 2.658466 | -1.343787 | -0.289922 |
| 12 | 6 | 0 | 2.001916 | -4.01049 | -0.730666 |
| 13 | 6 | 0 | -3.524915 | 1.322032 | 1.676757 |
| 14 | 6 | 0 | -1.959127 | 3.771216 | 1.683589 |
| 15 | 6 | 0 | 0.755683 | 3.117433 | 0.78276 |
| 16 | 6 | 0 | -0.496539 | 0.641841 | -3.089912 |
| 17 | 6 | 0 | -0.459636 | -1.253657 | 4.092367 |
| 18 | 6 | 0 | 1.014657 | -9.246252 | -2.141913 |
| 19 | 6 | 0 | -6.321849 | -9.67018 | -4.803063 |
| 20 | 6 | 0 | -3.217416 | -6.27496 | -5.430823 |
| 21 | 6 | 0 | 1.910409 | 5.221553 | -0.84798 |
| 22 | 6 | 0 | 4.539861 | 4.621505 | -1.851361 |
| 23 | 6 | 0 | 2.041063 | 7.683417 | 0.68663 |
| 24 | 6 | 0 | -0.341638 | 9.268544 | 0.759108 |
| 25 | 6 | 0 | -0.943972 | 10.514449 | 3.255101 |
| 26 | 6 | 0 | -3.390124 | 12.019093 | 3.372874 |
| 27 | 6 | 0 | -5.685781 | 10.286997 | 3.29358 |
| 28 | 6 | 0 | -3.433258 | 13.683159 | 5.720192 |
| 29 | 1 | 0 | 1.996882 | 2.927719 | 2.416157 |
| 30 | 8 | 0 | 4.841183 | -0.749794 | 0.191026 |
| 31 | 8 | 0 | -1.596584 | 9.62247 | -1.136111 |
| 32 | 1 | 0 | -3.398866 | -2.827065 | -1.494474 |
| 33 | 6 | 0 | 2.159947 | -9.047411 | 6.320863 |
| 34 | 8 | 0 | 0.319157 | -9.459289 | 7.591228 |
| 35 | 8 | 0 | 4.477636 | -8.960678 | 7.438492 |
| 36 | 1 | 0 | 3.563372 | -9.789226 | 2.626967 |
| 37 | 1 | 0 | 2.829826 | -6.633099 | 3.207485 |
| 38 | 1 | 0 | -3.788172 | -10.405416 | -0.819112 |
| 39 | 1 | 0 | -0.872023 | -10.916278 | 2.240475 |
| 40 | 1 | 0 | -1.805054 | -8.09921 | 3.611419 |
| 41 | 1 | 0 | -6.171352 | -8.320565 | 2.033147 |
| 42 | 1 | 0 | -7.155597 | -6.558041 | -0.553305 |
| 43 | 1 | 0 | -3.833416 | -5.031566 | 3.732091 |
| 44 | 1 | 0 | -6.112551 | -3.313783 | 2.124026 |
| 45 | 1 | 0 | 3.600918 | -5.201485 | -1.171581 |
| 46 | 1 | 0 | -4.816892 | 1.289201 | 0.073924 |
| 47 | 1 | 0 | -4.680494 | 1.127396 | 3.367039 |
| 48 | 1 | 0 | -1.924153 | 4.628476 | 3.552616 |
| 49 | 1 | 0 | -2.816794 | 5.148898 | 0.429455 |
| 50 | 1 | 0 | -0.893303 | -1.185011 | -3.926274 |
| 51 | 1 | 0 | -2.191668 | 1.792246 | -3.231737 |
| 52 | 1 | 0 | 0.936865 | 1.529008 | -4.259642 |
| 53 | 1 | 0 | 0.377805 | 0.446142 | 4.876382 |
| 54 | 1 | 0 | -1.934765 | -1.841498 | 5.395637 |
| 55 | 1 | 0 | 0.989248 | -2.706643 | 4.096135 |
| 56 | 1 | 0 | 2.9645 | -9.162314 | -1.504715 |
| 57 | 1 | 0 | 0.478957 | -11.234099 | -2.19338 |
| 58 | 1 | 0 | 0.952821 | -8.530858 | -4.059589 |
| 59 | 1 | 0 | -5.686111 | -11.634006 | -4.814801 |
| 60 | 1 | 0 | -7.966877 | -9.602711 | -3.561107 |
| 61 | 1 | 0 | -6.935339 | -9.186547 | -6.703118 |
| 62 | 1 | 0 | -3.868631 | -6.068848 | -7.358079 |
| 63 | 1 | 0 | -1.68198 | -5.056601 | -4.870183 |
| 64 | 1 | 0 | 0.661132 | 5.592026 | -2.445863 |
| 65 | 1 | 0 | 5.223807 | 6.180525 | -3.011726 |
| 66 | 1 | 0 | 4.580551 | 2.922016 | -2.99786 |
| 67 | 1 | 0 | 5.87428 | 4.320615 | -0.315599 |
| 68 | 1 | 0 | 2.730346 | 7.309321 | 2.594099 |
| 69 | 1 | 0 | 3.447397 | 8.924652 | -0.183042 |
| 70 | 1 | 0 | -0.914832 | 9.059517 | 4.725127 |
| 71 | 1 | 0 | 0.680667 | 11.72145 | 3.699271 |
| 72 | 1 | 0 | -3.450849 | 13.233218 | 1.704849 |
| 73 | 1 | 0 | -7.432164 | 11.37453 | 3.378455 |
| 74 | 1 | 0 | -5.722056 | 9.170738 | 1.570698 |
| 75 | 1 | 0 | -5.679307 | 8.992878 | 4.901019 |
| 76 | 1 | 0 | -5.159736 | 14.802198 | 5.803277 |
| 77 | 1 | 0 | -1.838193 | 14.987035 | 5.756828 |
| 78 | 1 | 0 | -3.351159 | 12.546085 | 7.439445 |
| 79 | 1 | 0 | 5.754787 | -8.574752 | 6.189099 |

| C3000003_en_ | | Standard Orientation (Ångstroms) | | | |
| --- | --- | --- | --- | --- | --- |
| Center number | Atomic number | Atomic Type | X | Y | Z |
| 0 | 6 | 0 | 1.801228 | -7.795049 | 4.034948 |
| 1 | 6 | 0 | -4.359234 | -7.711999 | -3.583612 |
| 2 | 6 | 0 | -3.761315 | -7.893461 | -0.790183 |
| 3 | 6 | 0 | -0.995332 | -7.33902 | 0.005609 |
| 4 | 6 | 0 | -0.75095 | -8.206374 | 2.805573 |
| 5 | 6 | 0 | -5.726474 | -6.339193 | 0.741562 |
| 6 | 6 | 0 | -4.728064 | -3.856046 | 1.831776 |
| 7 | 6 | 0 | -2.608099 | -2.718547 | 0.256691 |
| 8 | 6 | 0 | -0.441505 | -4.534131 | -0.160013 |
| 9 | 6 | 0 | -1.644338 | -0.154105 | 1.199157 |
| 10 | 6 | 0 | 0.452802 | 0.863065 | -0.64141 |
| 11 | 6 | 0 | 2.625291 | -0.973825 | -0.369745 |
| 12 | 6 | 0 | 1.906525 | -3.652344 | -0.568986 |
| 13 | 6 | 0 | -3.544227 | 2.0134 | 1.165784 |
| 14 | 6 | 0 | -1.907412 | 4.411344 | 1.032267 |
| 15 | 6 | 0 | 0.811102 | 3.604477 | 0.28993 |
| 16 | 6 | 0 | -0.385893 | 0.835979 | -3.41603 |
| 17 | 6 | 0 | -0.619446 | -0.388494 | 3.902015 |
| 18 | 6 | 0 | 0.800801 | -8.960845 | -1.575012 |
| 19 | 6 | 0 | -6.423797 | -9.459493 | -4.419548 |
| 20 | 6 | 0 | -3.254224 | -6.159801 | -5.219119 |
| 21 | 6 | 0 | 2.075099 | 5.533185 | -1.462092 |
| 22 | 6 | 0 | 4.72369 | 4.815331 | -2.321132 |
| 23 | 6 | 0 | 2.097737 | 8.195996 | -0.242773 |
| 24 | 6 | 0 | 3.588547 | 8.344246 | 2.192376 |
| 25 | 6 | 0 | 6.198675 | 9.492175 | 2.049806 |
| 26 | 6 | 0 | 7.894788 | 9.027065 | 4.323217 |
| 27 | 6 | 0 | 8.669375 | 6.260825 | 4.487617 |
| 28 | 6 | 0 | 10.200564 | 10.744676 | 4.233928 |
| 29 | 1 | 0 | 1.967712 | 3.514423 | 1.983174 |
| 30 | 8 | 0 | 4.820635 | -0.389594 | 0.068156 |
| 31 | 8 | 0 | 2.683211 | 7.621575 | 4.183503 |
| 32 | 1 | 0 | -3.433238 | -2.396804 | -1.6125 |
| 33 | 6 | 0 | 1.747138 | -8.264021 | 6.85395 |
| 34 | 8 | 0 | -0.070959 | -8.936898 | 8.042741 |
| 35 | 8 | 0 | 3.981956 | -7.867439 | 8.069837 |
| 36 | 1 | 0 | 3.246733 | -9.019631 | 3.217942 |
| 37 | 1 | 0 | 2.453096 | -5.866128 | 3.710037 |
| 38 | 1 | 0 | -4.059388 | -9.875916 | -0.293148 |
| 39 | 1 | 0 | -1.212608 | -10.213669 | 2.872466 |
| 40 | 1 | 0 | -2.156687 | -7.282389 | 3.970204 |
| 41 | 1 | 0 | -6.47664 | -7.488775 | 2.274743 |
| 42 | 1 | 0 | -7.325256 | -5.935457 | -0.488807 |
| 43 | 1 | 0 | -4.09929 | -4.140936 | 3.765593 |
| 44 | 1 | 0 | -6.268436 | -2.495481 | 1.946781 |
| 45 | 1 | 0 | 3.484248 | -4.918401 | -0.851601 |
| 46 | 1 | 0 | -4.780061 | 1.871761 | -0.476684 |
| 47 | 1 | 0 | -4.766025 | 1.991859 | 2.820571 |
| 48 | 1 | 0 | -1.877436 | 5.421854 | 2.819342 |
| 49 | 1 | 0 | -2.680008 | 5.71344 | -0.364385 |
| 50 | 1 | 0 | -0.767469 | -1.051891 | -4.113846 |
| 51 | 1 | 0 | -2.068076 | 1.977819 | -3.706196 |
| 52 | 1 | 0 | 1.09296 | 1.614757 | -4.607544 |
| 53 | 1 | 0 | 0.120109 | 1.395711 | 4.588964 |
| 54 | 1 | 0 | -2.122788 | -0.938352 | 5.189347 |
| 55 | 1 | 0 | 0.883046 | -1.777968 | 4.070496 |
| 56 | 1 | 0 | 2.733276 | -8.888676 | -0.887208 |
| 57 | 1 | 0 | 0.199162 | -10.92837 | -1.481334 |
| 58 | 1 | 0 | 0.815157 | -8.402918 | -3.545379 |
| 59 | 1 | 0 | -5.821371 | -11.425066 | -4.235034 |
| 60 | 1 | 0 | -8.1093 | -9.25872 | -3.248164 |
| 61 | 1 | 0 | -6.96145 | -9.136683 | -6.375926 |
| 62 | 1 | 0 | -3.833851 | -6.105776 | -7.179329 |
| 63 | 1 | 0 | -1.725227 | -4.914646 | -4.70034 |
| 64 | 1 | 0 | 0.887642 | 5.741816 | -3.140844 |
| 65 | 1 | 0 | 5.546825 | 6.326118 | -3.456962 |
| 66 | 1 | 0 | 4.731575 | 3.107918 | -3.457046 |
| 67 | 1 | 0 | 5.962735 | 4.446949 | -0.727165 |
| 68 | 1 | 0 | 2.866839 | 9.523427 | -1.618742 |
| 69 | 1 | 0 | 0.171639 | 8.775367 | 0.181929 |
| 70 | 1 | 0 | 7.113892 | 8.872285 | 0.306436 |
| 71 | 1 | 0 | 5.889431 | 11.524945 | 1.781167 |
| 72 | 1 | 0 | 6.799386 | 9.482352 | 6.011374 |
| 73 | 1 | 0 | 9.841156 | 5.924099 | 6.14702 |
| 74 | 1 | 0 | 7.034456 | 5.023269 | 4.61601 |
| 75 | 1 | 0 | 9.764462 | 5.700872 | 2.831632 |
| 76 | 1 | 0 | 11.41009 | 10.441583 | 5.872758 |
| 77 | 1 | 0 | 9.667269 | 12.735034 | 4.207547 |
| 78 | 1 | 0 | 11.335135 | 10.377444 | 2.550703 |
| 79 | 1 | 0 | 5.244527 | -7.289876 | 6.881126 |

| C3000003_tddft_ | | Standard Orientation (Ångstroms) | | | |
| --- | --- | --- | --- | --- | --- |
| Center number | Atomic number | Atomic Type | X | Y | Z |
| 0 | 6 | 0 | 1.801228 | -7.795049 | 4.034948 |
| 1 | 6 | 0 | -4.359234 | -7.711999 | -3.583612 |
| 2 | 6 | 0 | -3.761315 | -7.893461 | -0.790183 |
| 3 | 6 | 0 | -0.995332 | -7.33902 | 0.005609 |
| 4 | 6 | 0 | -0.75095 | -8.206374 | 2.805573 |
| 5 | 6 | 0 | -5.726474 | -6.339193 | 0.741562 |
| 6 | 6 | 0 | -4.728064 | -3.856046 | 1.831776 |
| 7 | 6 | 0 | -2.608099 | -2.718547 | 0.256691 |
| 8 | 6 | 0 | -0.441505 | -4.534131 | -0.160013 |
| 9 | 6 | 0 | -1.644338 | -0.154105 | 1.199157 |
| 10 | 6 | 0 | 0.452802 | 0.863065 | -0.64141 |
| 11 | 6 | 0 | 2.625291 | -0.973825 | -0.369745 |
| 12 | 6 | 0 | 1.906525 | -3.652344 | -0.568986 |
| 13 | 6 | 0 | -3.544227 | 2.0134 | 1.165784 |
| 14 | 6 | 0 | -1.907412 | 4.411344 | 1.032267 |
| 15 | 6 | 0 | 0.811102 | 3.604477 | 0.28993 |
| 16 | 6 | 0 | -0.385893 | 0.835979 | -3.41603 |
| 17 | 6 | 0 | -0.619446 | -0.388494 | 3.902015 |
| 18 | 6 | 0 | 0.800801 | -8.960845 | -1.575012 |
| 19 | 6 | 0 | -6.423797 | -9.459493 | -4.419548 |
| 20 | 6 | 0 | -3.254224 | -6.159801 | -5.219119 |
| 21 | 6 | 0 | 2.075099 | 5.533185 | -1.462092 |
| 22 | 6 | 0 | 4.72369 | 4.815331 | -2.321132 |
| 23 | 6 | 0 | 2.097737 | 8.195996 | -0.242773 |
| 24 | 6 | 0 | 3.588547 | 8.344246 | 2.192376 |
| 25 | 6 | 0 | 6.198675 | 9.492175 | 2.049806 |
| 26 | 6 | 0 | 7.894788 | 9.027065 | 4.323217 |
| 27 | 6 | 0 | 8.669375 | 6.260825 | 4.487617 |
| 28 | 6 | 0 | 10.200564 | 10.744676 | 4.233928 |
| 29 | 1 | 0 | 1.967712 | 3.514423 | 1.983174 |
| 30 | 8 | 0 | 4.820635 | -0.389594 | 0.068156 |
| 31 | 8 | 0 | 2.683211 | 7.621575 | 4.183503 |
| 32 | 1 | 0 | -3.433238 | -2.396804 | -1.6125 |
| 33 | 6 | 0 | 1.747138 | -8.264021 | 6.85395 |
| 34 | 8 | 0 | -0.070959 | -8.936898 | 8.042741 |
| 35 | 8 | 0 | 3.981956 | -7.867439 | 8.069837 |
| 36 | 1 | 0 | 3.246733 | -9.019631 | 3.217942 |
| 37 | 1 | 0 | 2.453096 | -5.866128 | 3.710037 |
| 38 | 1 | 0 | -4.059388 | -9.875916 | -0.293148 |
| 39 | 1 | 0 | -1.212608 | -10.213669 | 2.872466 |
| 40 | 1 | 0 | -2.156687 | -7.282389 | 3.970204 |
| 41 | 1 | 0 | -6.47664 | -7.488775 | 2.274743 |
| 42 | 1 | 0 | -7.325256 | -5.935457 | -0.488807 |
| 43 | 1 | 0 | -4.09929 | -4.140936 | 3.765593 |
| 44 | 1 | 0 | -6.268436 | -2.495481 | 1.946781 |
| 45 | 1 | 0 | 3.484248 | -4.918401 | -0.851601 |
| 46 | 1 | 0 | -4.780061 | 1.871761 | -0.476684 |
| 47 | 1 | 0 | -4.766025 | 1.991859 | 2.820571 |
| 48 | 1 | 0 | -1.877436 | 5.421854 | 2.819342 |
| 49 | 1 | 0 | -2.680008 | 5.71344 | -0.364385 |
| 50 | 1 | 0 | -0.767469 | -1.051891 | -4.113846 |
| 51 | 1 | 0 | -2.068076 | 1.977819 | -3.706196 |
| 52 | 1 | 0 | 1.09296 | 1.614757 | -4.607544 |
| 53 | 1 | 0 | 0.120109 | 1.395711 | 4.588964 |
| 54 | 1 | 0 | -2.122788 | -0.938352 | 5.189347 |
| 55 | 1 | 0 | 0.883046 | -1.777968 | 4.070496 |
| 56 | 1 | 0 | 2.733276 | -8.888676 | -0.887208 |
| 57 | 1 | 0 | 0.199162 | -10.92837 | -1.481334 |
| 58 | 1 | 0 | 0.815157 | -8.402918 | -3.545379 |
| 59 | 1 | 0 | -5.821371 | -11.425066 | -4.235034 |
| 60 | 1 | 0 | -8.1093 | -9.25872 | -3.248164 |
| 61 | 1 | 0 | -6.96145 | -9.136683 | -6.375926 |
| 62 | 1 | 0 | -3.833851 | -6.105776 | -7.179329 |
| 63 | 1 | 0 | -1.725227 | -4.914646 | -4.70034 |
| 64 | 1 | 0 | 0.887642 | 5.741816 | -3.140844 |
| 65 | 1 | 0 | 5.546825 | 6.326118 | -3.456962 |
| 66 | 1 | 0 | 4.731575 | 3.107918 | -3.457046 |
| 67 | 1 | 0 | 5.962735 | 4.446949 | -0.727165 |
| 68 | 1 | 0 | 2.866839 | 9.523427 | -1.618742 |
| 69 | 1 | 0 | 0.171639 | 8.775367 | 0.181929 |
| 70 | 1 | 0 | 7.113892 | 8.872285 | 0.306436 |
| 71 | 1 | 0 | 5.889431 | 11.524945 | 1.781167 |
| 72 | 1 | 0 | 6.799386 | 9.482352 | 6.011374 |
| 73 | 1 | 0 | 9.841156 | 5.924099 | 6.14702 |
| 74 | 1 | 0 | 7.034456 | 5.023269 | 4.61601 |
| 75 | 1 | 0 | 9.764462 | 5.700872 | 2.831632 |
| 76 | 1 | 0 | 11.41009 | 10.441583 | 5.872758 |
| 77 | 1 | 0 | 9.667269 | 12.735034 | 4.207547 |
| 78 | 1 | 0 | 11.335135 | 10.377444 | 2.550703 |
| 79 | 1 | 0 | 5.244527 | -7.289876 | 6.881126 |

| C3000004_en_ | | Standard Orientation (Ångstroms) | | | |
| --- | --- | --- | --- | --- | --- |
| Center number | Atomic number | Atomic Type | X | Y | Z |
| 0 | 6 | 0 | 1.394501 | -8.11393 | 3.726479 |
| 1 | 6 | 0 | -4.449009 | -6.879312 | -4.073452 |
| 2 | 6 | 0 | -3.926102 | -7.387875 | -1.307006 |
| 3 | 6 | 0 | -1.140646 | -7.170499 | -0.425546 |
| 4 | 6 | 0 | -1.047944 | -8.432262 | 2.237742 |
| 5 | 6 | 0 | -5.771462 | -5.813435 | 0.362265 |
| 6 | 6 | 0 | -4.533999 | -3.639992 | 1.805169 |
| 7 | 6 | 0 | -2.364504 | -2.491186 | 0.312765 |
| 8 | 6 | 0 | -0.349491 | -4.419812 | -0.283321 |
| 9 | 6 | 0 | -1.213814 | -0.086935 | 1.44208 |
| 10 | 6 | 0 | 0.944299 | 0.917483 | -0.330782 |
| 11 | 6 | 0 | 2.977803 | -1.091858 | -0.217603 |
| 12 | 6 | 0 | 2.057402 | -3.685561 | -0.639156 |
| 13 | 6 | 0 | -2.962517 | 2.200655 | 1.579085 |
| 14 | 6 | 0 | -1.165382 | 4.484997 | 1.602975 |
| 15 | 6 | 0 | 1.500265 | 3.556063 | 0.791396 |
| 16 | 6 | 0 | 0.094888 | 1.167674 | -3.091781 |
| 17 | 6 | 0 | -0.203915 | -0.591678 | 4.110891 |
| 18 | 6 | 0 | 0.579563 | -8.744785 | -2.135905 |
| 19 | 6 | 0 | -6.69607 | -8.274486 | -5.085284 |
| 20 | 6 | 0 | -3.125084 | -5.335524 | -5.546258 |
| 21 | 6 | 0 | 2.872956 | 5.508504 | -0.854275 |
| 22 | 6 | 0 | 5.453151 | 4.673994 | -1.806218 |
| 23 | 6 | 0 | 3.171921 | 8.003365 | 0.635272 |
| 24 | 6 | 0 | 3.713893 | 10.188436 | -1.106301 |
| 25 | 6 | 0 | 1.443115 | 11.348349 | -2.41629 |
| 26 | 6 | 0 | 1.971474 | 12.414459 | -5.037482 |
| 27 | 6 | 0 | 2.54677 | 10.299893 | -6.901809 |
| 28 | 6 | 0 | -0.226571 | 14.031133 | -5.945058 |
| 29 | 1 | 0 | 2.673602 | 3.272191 | 2.459664 |
| 30 | 8 | 0 | 5.20742 | -0.713756 | 0.261498 |
| 31 | 8 | 0 | 5.84024 | 10.997699 | -1.443631 |
| 32 | 1 | 0 | -3.179733 | -1.959481 | -1.513171 |
| 33 | 6 | 0 | 1.02147 | -6.903852 | 6.295934 |
| 34 | 8 | 0 | -0.985973 | -6.526153 | 7.299782 |
| 35 | 8 | 0 | 3.182289 | -6.254266 | 7.520899 |
| 36 | 1 | 0 | 2.315387 | -9.931443 | 4.054032 |
| 37 | 1 | 0 | 2.759536 | -6.976557 | 2.68508 |
| 38 | 1 | 0 | -4.413839 | -9.374788 | -1.018587 |
| 39 | 1 | 0 | -1.415633 | -10.435251 | 1.929611 |
| 40 | 1 | 0 | -2.583898 | -7.765483 | 3.412182 |
| 41 | 1 | 0 | -6.740039 | -7.049338 | 1.693001 |
| 42 | 1 | 0 | -7.237866 | -5.03997 | -0.856704 |
| 43 | 1 | 0 | -3.875102 | -4.272147 | 3.642126 |
| 44 | 1 | 0 | -5.945491 | -2.188404 | 2.179707 |
| 45 | 1 | 0 | 3.529381 | -5.031521 | -1.082259 |
| 46 | 1 | 0 | -4.209991 | 2.258146 | -0.058566 |
| 47 | 1 | 0 | -4.175145 | 2.144209 | 3.239565 |
| 48 | 1 | 0 | -1.10573 | 5.363569 | 3.460589 |
| 49 | 1 | 0 | -1.840429 | 5.93159 | 0.3019 |
| 50 | 1 | 0 | -0.459788 | -0.62208 | -3.917614 |
| 51 | 1 | 0 | -1.485429 | 2.466306 | -3.282539 |
| 52 | 1 | 0 | 1.62764 | 1.897843 | -4.243457 |
| 53 | 1 | 0 | 0.520833 | 1.118751 | 4.980351 |
| 54 | 1 | 0 | -1.701369 | -1.291277 | 5.327456 |
| 55 | 1 | 0 | 1.310355 | -1.975597 | 4.143821 |
| 56 | 1 | 0 | 2.429367 | -9.04583 | -1.288492 |
| 57 | 1 | 0 | -0.261582 | -10.602175 | -2.424258 |
| 58 | 1 | 0 | 0.858406 | -7.893658 | -3.977806 |
| 59 | 1 | 0 | -6.347815 | -10.30912 | -5.063976 |
| 60 | 1 | 0 | -8.373569 | -7.963002 | -3.926819 |
| 61 | 1 | 0 | -7.140099 | -7.722568 | -7.013714 |
| 62 | 1 | 0 | -3.647165 | -5.024715 | -7.498278 |
| 63 | 1 | 0 | -1.464942 | -4.352891 | -4.887746 |
| 64 | 1 | 0 | 1.668247 | 5.903858 | -2.486927 |
| 65 | 1 | 0 | 6.382654 | 6.198077 | -2.826452 |
| 66 | 1 | 0 | 5.335571 | 3.051371 | -3.055631 |
| 67 | 1 | 0 | 6.66573 | 4.12727 | -0.240354 |
| 68 | 1 | 0 | 1.44625 | 8.421036 | 1.677499 |
| 69 | 1 | 0 | 4.717556 | 7.816314 | 1.981173 |
| 70 | 1 | 0 | -0.107173 | 9.986689 | -2.485597 |
| 71 | 1 | 0 | 0.795851 | 12.859008 | -1.155255 |
| 72 | 1 | 0 | 3.648311 | 13.605261 | -4.88159 |
| 73 | 1 | 0 | 3.002447 | 11.056072 | -8.761825 |
| 74 | 1 | 0 | 4.144328 | 9.160875 | -6.285692 |
| 75 | 1 | 0 | 0.917072 | 9.052951 | -7.120811 |
| 76 | 1 | 0 | 0.154785 | 14.824579 | -7.806765 |
| 77 | 1 | 0 | -0.593955 | 15.597041 | -4.658398 |
| 78 | 1 | 0 | -1.958381 | 12.918941 | -6.089105 |
| 79 | 1 | 0 | 4.631716 | -6.595658 | 6.460777 |

| C3000004_tddft_ | | Standard Orientation (Ångstroms) | | | |
| --- | --- | --- | --- | --- | --- |
| Center number | Atomic number | Atomic Type | X | Y | Z |
| 0 | 6 | 0 | 1.394501 | -8.11393 | 3.726479 |
| 1 | 6 | 0 | -4.449009 | -6.879312 | -4.073452 |
| 2 | 6 | 0 | -3.926102 | -7.387875 | -1.307006 |
| 3 | 6 | 0 | -1.140646 | -7.170499 | -0.425546 |
| 4 | 6 | 0 | -1.047944 | -8.432262 | 2.237742 |
| 5 | 6 | 0 | -5.771462 | -5.813435 | 0.362265 |
| 6 | 6 | 0 | -4.533999 | -3.639992 | 1.805169 |
| 7 | 6 | 0 | -2.364504 | -2.491186 | 0.312765 |
| 8 | 6 | 0 | -0.349491 | -4.419812 | -0.283321 |
| 9 | 6 | 0 | -1.213814 | -0.086935 | 1.44208 |
| 10 | 6 | 0 | 0.944299 | 0.917483 | -0.330782 |
| 11 | 6 | 0 | 2.977803 | -1.091858 | -0.217603 |
| 12 | 6 | 0 | 2.057402 | -3.685561 | -0.639156 |
| 13 | 6 | 0 | -2.962517 | 2.200655 | 1.579085 |
| 14 | 6 | 0 | -1.165382 | 4.484997 | 1.602975 |
| 15 | 6 | 0 | 1.500265 | 3.556063 | 0.791396 |
| 16 | 6 | 0 | 0.094888 | 1.167674 | -3.091781 |
| 17 | 6 | 0 | -0.203915 | -0.591678 | 4.110891 |
| 18 | 6 | 0 | 0.579563 | -8.744785 | -2.135905 |
| 19 | 6 | 0 | -6.69607 | -8.274486 | -5.085284 |
| 20 | 6 | 0 | -3.125084 | -5.335524 | -5.546258 |
| 21 | 6 | 0 | 2.872956 | 5.508504 | -0.854275 |
| 22 | 6 | 0 | 5.453151 | 4.673994 | -1.806218 |
| 23 | 6 | 0 | 3.171921 | 8.003365 | 0.635272 |
| 24 | 6 | 0 | 3.713893 | 10.188436 | -1.106301 |
| 25 | 6 | 0 | 1.443115 | 11.348349 | -2.41629 |
| 26 | 6 | 0 | 1.971474 | 12.414459 | -5.037482 |
| 27 | 6 | 0 | 2.54677 | 10.299893 | -6.901809 |
| 28 | 6 | 0 | -0.226571 | 14.031133 | -5.945058 |
| 29 | 1 | 0 | 2.673602 | 3.272191 | 2.459664 |
| 30 | 8 | 0 | 5.20742 | -0.713756 | 0.261498 |
| 31 | 8 | 0 | 5.84024 | 10.997699 | -1.443631 |
| 32 | 1 | 0 | -3.179733 | -1.959481 | -1.513171 |
| 33 | 6 | 0 | 1.02147 | -6.903852 | 6.295934 |
| 34 | 8 | 0 | -0.985973 | -6.526153 | 7.299782 |
| 35 | 8 | 0 | 3.182289 | -6.254266 | 7.520899 |
| 36 | 1 | 0 | 2.315387 | -9.931443 | 4.054032 |
| 37 | 1 | 0 | 2.759536 | -6.976557 | 2.68508 |
| 38 | 1 | 0 | -4.413839 | -9.374788 | -1.018587 |
| 39 | 1 | 0 | -1.415633 | -10.435251 | 1.929611 |
| 40 | 1 | 0 | -2.583898 | -7.765483 | 3.412182 |
| 41 | 1 | 0 | -6.740039 | -7.049338 | 1.693001 |
| 42 | 1 | 0 | -7.237866 | -5.03997 | -0.856704 |
| 43 | 1 | 0 | -3.875102 | -4.272147 | 3.642126 |
| 44 | 1 | 0 | -5.945491 | -2.188404 | 2.179707 |
| 45 | 1 | 0 | 3.529381 | -5.031521 | -1.082259 |
| 46 | 1 | 0 | -4.209991 | 2.258146 | -0.058566 |
| 47 | 1 | 0 | -4.175145 | 2.144209 | 3.239565 |
| 48 | 1 | 0 | -1.10573 | 5.363569 | 3.460589 |
| 49 | 1 | 0 | -1.840429 | 5.93159 | 0.3019 |
| 50 | 1 | 0 | -0.459788 | -0.62208 | -3.917614 |
| 51 | 1 | 0 | -1.485429 | 2.466306 | -3.282539 |
| 52 | 1 | 0 | 1.62764 | 1.897843 | -4.243457 |
| 53 | 1 | 0 | 0.520833 | 1.118751 | 4.980351 |
| 54 | 1 | 0 | -1.701369 | -1.291277 | 5.327456 |
| 55 | 1 | 0 | 1.310355 | -1.975597 | 4.143821 |
| 56 | 1 | 0 | 2.429367 | -9.04583 | -1.288492 |
| 57 | 1 | 0 | -0.261582 | -10.602175 | -2.424258 |
| 58 | 1 | 0 | 0.858406 | -7.893658 | -3.977806 |
| 59 | 1 | 0 | -6.347815 | -10.30912 | -5.063976 |
| 60 | 1 | 0 | -8.373569 | -7.963002 | -3.926819 |
| 61 | 1 | 0 | -7.140099 | -7.722568 | -7.013714 |
| 62 | 1 | 0 | -3.647165 | -5.024715 | -7.498278 |
| 63 | 1 | 0 | -1.464942 | -4.352891 | -4.887746 |
| 64 | 1 | 0 | 1.668247 | 5.903858 | -2.486927 |
| 65 | 1 | 0 | 6.382654 | 6.198077 | -2.826452 |
| 66 | 1 | 0 | 5.335571 | 3.051371 | -3.055631 |
| 67 | 1 | 0 | 6.66573 | 4.12727 | -0.240354 |
| 68 | 1 | 0 | 1.44625 | 8.421036 | 1.677499 |
| 69 | 1 | 0 | 4.717556 | 7.816314 | 1.981173 |
| 70 | 1 | 0 | -0.107173 | 9.986689 | -2.485597 |
| 71 | 1 | 0 | 0.795851 | 12.859008 | -1.155255 |
| 72 | 1 | 0 | 3.648311 | 13.605261 | -4.88159 |
| 73 | 1 | 0 | 3.002447 | 11.056072 | -8.761825 |
| 74 | 1 | 0 | 4.144328 | 9.160875 | -6.285692 |
| 75 | 1 | 0 | 0.917072 | 9.052951 | -7.120811 |
| 76 | 1 | 0 | 0.154785 | 14.824579 | -7.806765 |
| 77 | 1 | 0 | -0.593955 | 15.597041 | -4.658398 |
| 78 | 1 | 0 | -1.958381 | 12.918941 | -6.089105 |
| 79 | 1 | 0 | 4.631716 | -6.595658 | 6.460777 |

| C3000005_en_ | | Standard Orientation (Ångstroms) | | | |
| --- | --- | --- | --- | --- | --- |
| Center number | Atomic number | Atomic Type | X | Y | Z |
| 0 | 6 | 0 | 1.540179 | -7.739299 | 4.08982 |
| 1 | 6 | 0 | -4.348829 | -8.272719 | -3.606439 |
| 2 | 6 | 0 | -4.000216 | -7.955736 | -0.790179 |
| 3 | 6 | 0 | -1.218262 | -7.39379 | 0.011717 |
| 4 | 6 | 0 | -1.018469 | -8.117194 | 2.855919 |
| 5 | 6 | 0 | -5.962448 | -6.077086 | 0.318137 |
| 6 | 6 | 0 | -4.883916 | -3.718213 | 1.574575 |
| 7 | 6 | 0 | -2.641829 | -2.687927 | 0.107193 |
| 8 | 6 | 0 | -0.571383 | -4.620127 | -0.282317 |
| 9 | 6 | 0 | -1.548737 | -0.189029 | 1.076571 |
| 10 | 6 | 0 | 0.586013 | 0.731856 | -0.770291 |
| 11 | 6 | 0 | 2.663125 | -1.213053 | -0.526146 |
| 12 | 6 | 0 | 1.809813 | -3.851756 | -0.736347 |
| 13 | 6 | 0 | -3.335059 | 2.07474 | 1.067834 |
| 14 | 6 | 0 | -1.57528 | 4.38638 | 0.938775 |
| 15 | 6 | 0 | 1.096164 | 3.450036 | 0.159752 |
| 16 | 6 | 0 | -0.274268 | 0.756056 | -3.540599 |
| 17 | 6 | 0 | -0.541045 | -0.509895 | 3.774464 |
| 18 | 6 | 0 | 0.552577 | -9.131532 | -1.465359 |
| 19 | 6 | 0 | -3.787924 | -6.095227 | -5.319184 |
| 20 | 6 | 0 | -5.183773 | -10.45623 | -4.532782 |
| 21 | 6 | 0 | 2.419983 | 5.314586 | -1.620047 |
| 22 | 6 | 0 | 5.011757 | 4.459414 | -2.532166 |
| 23 | 6 | 0 | 2.715313 | 7.867639 | -0.300224 |
| 24 | 6 | 0 | 3.461659 | 10.015389 | -2.036612 |
| 25 | 6 | 0 | 5.192849 | 11.983757 | -0.907621 |
| 26 | 6 | 0 | 5.449749 | 14.43579 | -2.38388 |
| 27 | 6 | 0 | 3.012602 | 15.961814 | -2.295722 |
| 28 | 6 | 0 | 7.673907 | 15.97761 | -1.406889 |
| 29 | 1 | 0 | 2.288492 | 3.295002 | 1.833434 |
| 30 | 8 | 0 | 4.883796 | -0.7487 | -0.075874 |
| 31 | 8 | 0 | 2.707197 | 10.133399 | -4.205254 |
| 32 | 1 | 0 | -3.386222 | -2.284411 | -1.778757 |
| 33 | 6 | 0 | 1.380224 | -7.53723 | 6.938571 |
| 34 | 8 | 0 | -0.542068 | -7.384294 | 8.14504 |
| 35 | 8 | 0 | 3.644964 | -7.484989 | 8.158163 |
| 36 | 1 | 0 | 2.850437 | -9.255859 | 3.602931 |
| 37 | 1 | 0 | 2.416807 | -5.999121 | 3.409663 |
| 38 | 1 | 0 | -4.399013 | -9.80638 | 0.020023 |
| 39 | 1 | 0 | -1.552978 | -10.101712 | 3.015754 |
| 40 | 1 | 0 | -2.400664 | -7.090545 | 3.960063 |
| 41 | 1 | 0 | -7.158383 | -7.076255 | 1.661754 |
| 42 | 1 | 0 | -7.224779 | -5.493842 | -1.201245 |
| 43 | 1 | 0 | -4.374569 | -4.096385 | 3.52749 |
| 44 | 1 | 0 | -6.349079 | -2.272874 | 1.650082 |
| 45 | 1 | 0 | 3.326712 | -5.190909 | -1.013277 |
| 46 | 1 | 0 | -4.585806 | 2.012612 | -0.56732 |
| 47 | 1 | 0 | -4.546241 | 2.104929 | 2.730311 |
| 48 | 1 | 0 | -1.512085 | 5.370048 | 2.743602 |
| 49 | 1 | 0 | -2.282637 | 5.743628 | -0.4383 |
| 50 | 1 | 0 | -0.744571 | -1.114401 | -4.236782 |
| 51 | 1 | 0 | -1.904296 | 1.975497 | -3.812029 |
| 52 | 1 | 0 | 1.22613 | 1.474389 | -4.740343 |
| 53 | 1 | 0 | 0.224133 | 1.245548 | 4.508475 |
| 54 | 1 | 0 | -2.059607 | -1.07228 | 5.038817 |
| 55 | 1 | 0 | 0.938635 | -1.924586 | 3.916526 |
| 56 | 1 | 0 | 2.470795 | -9.09086 | -0.736467 |
| 57 | 1 | 0 | -0.115499 | -11.072057 | -1.329038 |
| 58 | 1 | 0 | 0.635054 | -8.642552 | -3.456393 |
| 59 | 1 | 0 | -5.000894 | -4.47622 | -4.923664 |
| 60 | 1 | 0 | -1.847302 | -5.44243 | -5.095038 |
| 61 | 1 | 0 | -4.061951 | -6.607866 | -7.2892 |
| 62 | 1 | 0 | -5.492127 | -10.731167 | -6.536122 |
| 63 | 1 | 0 | -5.596528 | -12.05106 | -3.320622 |
| 64 | 1 | 0 | 1.215804 | 5.633898 | -3.263158 |
| 65 | 1 | 0 | 5.853876 | 5.895799 | -3.742056 |
| 66 | 1 | 0 | 4.934273 | 2.715246 | -3.606721 |
| 67 | 1 | 0 | 6.269074 | 4.110399 | -0.942706 |
| 68 | 1 | 0 | 0.938755 | 8.474721 | 0.564377 |
| 69 | 1 | 0 | 4.067514 | 7.71374 | 1.252481 |
| 70 | 1 | 0 | 4.60435 | 12.354117 | 1.041052 |
| 71 | 1 | 0 | 7.033235 | 11.047748 | -0.725181 |
| 72 | 1 | 0 | 5.812684 | 13.931095 | -4.351636 |
| 73 | 1 | 0 | 3.190981 | 17.698818 | -3.387116 |
| 74 | 1 | 0 | 1.432655 | 14.897717 | -3.066753 |
| 75 | 1 | 0 | 2.551027 | 16.492741 | -0.355356 |
| 76 | 1 | 0 | 7.892131 | 17.719986 | -2.481692 |
| 77 | 1 | 0 | 9.443213 | 14.930814 | -1.542326 |
| 78 | 1 | 0 | 7.405502 | 16.499763 | 0.570632 |
| 79 | 1 | 0 | 5.007802 | -7.577915 | 6.94392 |

| C3000005_tddft_ | | Standard Orientation (Ångstroms) | | | |
| --- | --- | --- | --- | --- | --- |
| Center number | Atomic number | Atomic Type | X | Y | Z |
| 0 | 6 | 0 | 1.540179 | -7.739299 | 4.08982 |
| 1 | 6 | 0 | -4.348829 | -8.272719 | -3.606439 |
| 2 | 6 | 0 | -4.000216 | -7.955736 | -0.790179 |
| 3 | 6 | 0 | -1.218262 | -7.39379 | 0.011717 |
| 4 | 6 | 0 | -1.018469 | -8.117194 | 2.855919 |
| 5 | 6 | 0 | -5.962448 | -6.077086 | 0.318137 |
| 6 | 6 | 0 | -4.883916 | -3.718213 | 1.574575 |
| 7 | 6 | 0 | -2.641829 | -2.687927 | 0.107193 |
| 8 | 6 | 0 | -0.571383 | -4.620127 | -0.282317 |
| 9 | 6 | 0 | -1.548737 | -0.189029 | 1.076571 |
| 10 | 6 | 0 | 0.586013 | 0.731856 | -0.770291 |
| 11 | 6 | 0 | 2.663125 | -1.213053 | -0.526146 |
| 12 | 6 | 0 | 1.809813 | -3.851756 | -0.736347 |
| 13 | 6 | 0 | -3.335059 | 2.07474 | 1.067834 |
| 14 | 6 | 0 | -1.57528 | 4.38638 | 0.938775 |
| 15 | 6 | 0 | 1.096164 | 3.450036 | 0.159752 |
| 16 | 6 | 0 | -0.274268 | 0.756056 | -3.540599 |
| 17 | 6 | 0 | -0.541045 | -0.509895 | 3.774464 |
| 18 | 6 | 0 | 0.552577 | -9.131532 | -1.465359 |
| 19 | 6 | 0 | -3.787924 | -6.095227 | -5.319184 |
| 20 | 6 | 0 | -5.183773 | -10.45623 | -4.532782 |
| 21 | 6 | 0 | 2.419983 | 5.314586 | -1.620047 |
| 22 | 6 | 0 | 5.011757 | 4.459414 | -2.532166 |
| 23 | 6 | 0 | 2.715313 | 7.867639 | -0.300224 |
| 24 | 6 | 0 | 3.461659 | 10.015389 | -2.036612 |
| 25 | 6 | 0 | 5.192849 | 11.983757 | -0.907621 |
| 26 | 6 | 0 | 5.449749 | 14.43579 | -2.38388 |
| 27 | 6 | 0 | 3.012602 | 15.961814 | -2.295722 |
| 28 | 6 | 0 | 7.673907 | 15.97761 | -1.406889 |
| 29 | 1 | 0 | 2.288492 | 3.295002 | 1.833434 |
| 30 | 8 | 0 | 4.883796 | -0.7487 | -0.075874 |
| 31 | 8 | 0 | 2.707197 | 10.133399 | -4.205254 |
| 32 | 1 | 0 | -3.386222 | -2.284411 | -1.778757 |
| 33 | 6 | 0 | 1.380224 | -7.53723 | 6.938571 |
| 34 | 8 | 0 | -0.542068 | -7.384294 | 8.14504 |
| 35 | 8 | 0 | 3.644964 | -7.484989 | 8.158163 |
| 36 | 1 | 0 | 2.850437 | -9.255859 | 3.602931 |
| 37 | 1 | 0 | 2.416807 | -5.999121 | 3.409663 |
| 38 | 1 | 0 | -4.399013 | -9.80638 | 0.020023 |
| 39 | 1 | 0 | -1.552978 | -10.101712 | 3.015754 |
| 40 | 1 | 0 | -2.400664 | -7.090545 | 3.960063 |
| 41 | 1 | 0 | -7.158383 | -7.076255 | 1.661754 |
| 42 | 1 | 0 | -7.224779 | -5.493842 | -1.201245 |
| 43 | 1 | 0 | -4.374569 | -4.096385 | 3.52749 |
| 44 | 1 | 0 | -6.349079 | -2.272874 | 1.650082 |
| 45 | 1 | 0 | 3.326712 | -5.190909 | -1.013277 |
| 46 | 1 | 0 | -4.585806 | 2.012612 | -0.56732 |
| 47 | 1 | 0 | -4.546241 | 2.104929 | 2.730311 |
| 48 | 1 | 0 | -1.512085 | 5.370048 | 2.743602 |
| 49 | 1 | 0 | -2.282637 | 5.743628 | -0.4383 |
| 50 | 1 | 0 | -0.744571 | -1.114401 | -4.236782 |
| 51 | 1 | 0 | -1.904296 | 1.975497 | -3.812029 |
| 52 | 1 | 0 | 1.22613 | 1.474389 | -4.740343 |
| 53 | 1 | 0 | 0.224133 | 1.245548 | 4.508475 |
| 54 | 1 | 0 | -2.059607 | -1.07228 | 5.038817 |
| 55 | 1 | 0 | 0.938635 | -1.924586 | 3.916526 |
| 56 | 1 | 0 | 2.470795 | -9.09086 | -0.736467 |
| 57 | 1 | 0 | -0.115499 | -11.072057 | -1.329038 |
| 58 | 1 | 0 | 0.635054 | -8.642552 | -3.456393 |
| 59 | 1 | 0 | -5.000894 | -4.47622 | -4.923664 |
| 60 | 1 | 0 | -1.847302 | -5.44243 | -5.095038 |
| 61 | 1 | 0 | -4.061951 | -6.607866 | -7.2892 |
| 62 | 1 | 0 | -5.492127 | -10.731167 | -6.536122 |
| 63 | 1 | 0 | -5.596528 | -12.05106 | -3.320622 |
| 64 | 1 | 0 | 1.215804 | 5.633898 | -3.263158 |
| 65 | 1 | 0 | 5.853876 | 5.895799 | -3.742056 |
| 66 | 1 | 0 | 4.934273 | 2.715246 | -3.606721 |
| 67 | 1 | 0 | 6.269074 | 4.110399 | -0.942706 |
| 68 | 1 | 0 | 0.938755 | 8.474721 | 0.564377 |
| 69 | 1 | 0 | 4.067514 | 7.71374 | 1.252481 |
| 70 | 1 | 0 | 4.60435 | 12.354117 | 1.041052 |
| 71 | 1 | 0 | 7.033235 | 11.047748 | -0.725181 |
| 72 | 1 | 0 | 5.812684 | 13.931095 | -4.351636 |
| 73 | 1 | 0 | 3.190981 | 17.698818 | -3.387116 |
| 74 | 1 | 0 | 1.432655 | 14.897717 | -3.066753 |
| 75 | 1 | 0 | 2.551027 | 16.492741 | -0.355356 |
| 76 | 1 | 0 | 7.892131 | 17.719986 | -2.481692 |
| 77 | 1 | 0 | 9.443213 | 14.930814 | -1.542326 |
| 78 | 1 | 0 | 7.405502 | 16.499763 | 0.570632 |
| 79 | 1 | 0 | 5.007802 | -7.577915 | 6.94392 |

| C3000006_en_ | | Standard Orientation (Ångstroms) | | | |
| --- | --- | --- | --- | --- | --- |
| Center number | Atomic number | Atomic Type | X | Y | Z |
| 0 | 6 | 0 | 1.804616 | -7.607687 | 3.987609 |
| 1 | 6 | 0 | -4.217639 | -8.141798 | -3.60171 |
| 2 | 6 | 0 | -3.818363 | -7.898389 | -0.783857 |
| 3 | 6 | 0 | -1.04348 | -7.247385 | -0.019046 |
| 4 | 6 | 0 | -0.754437 | -8.067552 | 2.789625 |
| 5 | 6 | 0 | -5.832932 | -6.143142 | 0.426533 |
| 6 | 6 | 0 | -4.827444 | -3.769931 | 1.71642 |
| 7 | 6 | 0 | -2.660445 | -2.609745 | 0.234754 |
| 8 | 6 | 0 | -0.515314 | -4.439409 | -0.227864 |
| 9 | 6 | 0 | -1.668004 | -0.082221 | 1.237997 |
| 10 | 6 | 0 | 0.392648 | 0.970131 | -0.623552 |
| 11 | 6 | 0 | 2.559626 | -0.885447 | -0.459624 |
| 12 | 6 | 0 | 1.824084 | -3.556287 | -0.688393 |
| 13 | 6 | 0 | -3.557318 | 2.093847 | 1.306515 |
| 14 | 6 | 0 | -1.908525 | 4.485682 | 1.18917 |
| 15 | 6 | 0 | 0.790545 | 3.680187 | 0.377973 |
| 16 | 6 | 0 | -0.510122 | 1.02242 | -3.379305 |
| 17 | 6 | 0 | -0.596966 | -0.393637 | 3.913614 |
| 18 | 6 | 0 | 0.756631 | -8.85246 | -1.607742 |
| 19 | 6 | 0 | -3.833339 | -5.880111 | -5.252313 |
| 20 | 6 | 0 | -4.950113 | -10.338603 | -4.582346 |
| 21 | 6 | 0 | 2.036091 | 5.651026 | -1.34005 |
| 22 | 6 | 0 | 4.657425 | 4.940777 | -2.284301 |
| 23 | 6 | 0 | 2.109893 | 8.273929 | -0.039952 |
| 24 | 6 | 0 | 3.66807 | 8.332924 | 2.35639 |
| 25 | 6 | 0 | 6.278475 | 9.474809 | 2.180732 |
| 26 | 6 | 0 | 8.034313 | 8.931467 | 4.390747 |
| 27 | 6 | 0 | 8.796312 | 6.157483 | 4.450813 |
| 28 | 6 | 0 | 10.347366 | 10.638604 | 4.287675 |
| 29 | 1 | 0 | 1.974382 | 3.525724 | 2.047682 |
| 30 | 8 | 0 | 4.769993 | -0.32255 | -0.070587 |
| 31 | 8 | 0 | 2.813492 | 7.545914 | 4.345621 |
| 32 | 1 | 0 | -3.454147 | -2.203396 | -1.629744 |
| 33 | 6 | 0 | 1.776822 | -7.988554 | 6.820216 |
| 34 | 8 | 0 | -0.039531 | -8.582568 | 8.052795 |
| 35 | 8 | 0 | 4.034528 | -7.598602 | 7.995373 |
| 36 | 1 | 0 | 3.247052 | -8.849773 | 3.191696 |
| 37 | 1 | 0 | 2.448822 | -5.687593 | 3.602573 |
| 38 | 1 | 0 | -4.122056 | -9.789468 | -0.028396 |
| 39 | 1 | 0 | -1.18249 | -10.080691 | 2.886121 |
| 40 | 1 | 0 | -2.169646 | -7.154296 | 3.952672 |
| 41 | 1 | 0 | -6.945717 | -7.229057 | 1.774255 |
| 42 | 1 | 0 | -7.161682 | -5.580845 | -1.042619 |
| 43 | 1 | 0 | -4.261643 | -4.175307 | 3.647915 |
| 44 | 1 | 0 | -6.349314 | -2.390371 | 1.862888 |
| 45 | 1 | 0 | 3.39592 | -4.821471 | -1.002477 |
| 46 | 1 | 0 | -4.840081 | 2.002268 | -0.303404 |
| 47 | 1 | 0 | -4.732268 | 2.036506 | 2.994164 |
| 48 | 1 | 0 | -1.839444 | 5.462583 | 2.993907 |
| 49 | 1 | 0 | -2.699833 | 5.818896 | -0.167195 |
| 50 | 1 | 0 | -0.934234 | -0.844057 | -4.113682 |
| 51 | 1 | 0 | -2.18116 | 2.196161 | -3.601268 |
| 52 | 1 | 0 | 0.952505 | 1.81026 | -4.584749 |
| 53 | 1 | 0 | 0.011038 | 1.40135 | 4.696375 |
| 54 | 1 | 0 | -2.04571 | -1.126309 | 5.172858 |
| 55 | 1 | 0 | 1.007508 | -1.671842 | 3.999146 |
| 56 | 1 | 0 | 2.694894 | -8.755142 | -0.93987 |
| 57 | 1 | 0 | 0.177176 | -10.82417 | -1.52585 |
| 58 | 1 | 0 | 0.752709 | -8.286847 | -3.580097 |
| 59 | 1 | 0 | -5.131332 | -4.353962 | -4.767532 |
| 60 | 1 | 0 | -1.929122 | -5.117854 | -5.058647 |
| 61 | 1 | 0 | -4.133026 | -6.343056 | -7.23098 |
| 62 | 1 | 0 | -5.292028 | -10.565859 | -6.586365 |
| 63 | 1 | 0 | -5.240515 | -11.993021 | -3.415109 |
| 64 | 1 | 0 | 0.81384 | 5.91967 | -2.984915 |
| 65 | 1 | 0 | 5.463912 | 6.481697 | -3.391263 |
| 66 | 1 | 0 | 4.623381 | 3.271084 | -3.474607 |
| 67 | 1 | 0 | 5.93431 | 4.51134 | -0.735955 |
| 68 | 1 | 0 | 2.849538 | 9.639156 | -1.395041 |
| 69 | 1 | 0 | 0.200248 | 8.850727 | 0.456681 |
| 70 | 1 | 0 | 7.143396 | 8.908702 | 0.39386 |
| 71 | 1 | 0 | 5.971337 | 11.516355 | 1.985747 |
| 72 | 1 | 0 | 6.989656 | 9.341424 | 6.122195 |
| 73 | 1 | 0 | 10.018165 | 5.766993 | 6.0616 |
| 74 | 1 | 0 | 7.158272 | 4.925988 | 4.596723 |
| 75 | 1 | 0 | 9.83464 | 5.639709 | 2.745399 |
| 76 | 1 | 0 | 11.603857 | 10.275169 | 5.878188 |
| 77 | 1 | 0 | 9.82675 | 12.631686 | 4.34205 |
| 78 | 1 | 0 | 11.428283 | 10.320458 | 2.559735 |
| 79 | 1 | 0 | 5.294232 | -7.089438 | 6.772702 |

| C3000006_tddft_ | | Standard Orientation (Ångstroms) | | | |
| --- | --- | --- | --- | --- | --- |
| Center number | Atomic number | Atomic Type | X | Y | Z |
| 0 | 6 | 0 | 1.804616 | -7.607687 | 3.987609 |
| 1 | 6 | 0 | -4.217639 | -8.141798 | -3.60171 |
| 2 | 6 | 0 | -3.818363 | -7.898389 | -0.783857 |
| 3 | 6 | 0 | -1.04348 | -7.247385 | -0.019046 |
| 4 | 6 | 0 | -0.754437 | -8.067552 | 2.789625 |
| 5 | 6 | 0 | -5.832932 | -6.143142 | 0.426533 |
| 6 | 6 | 0 | -4.827444 | -3.769931 | 1.71642 |
| 7 | 6 | 0 | -2.660445 | -2.609745 | 0.234754 |
| 8 | 6 | 0 | -0.515314 | -4.439409 | -0.227864 |
| 9 | 6 | 0 | -1.668004 | -0.082221 | 1.237997 |
| 10 | 6 | 0 | 0.392648 | 0.970131 | -0.623552 |
| 11 | 6 | 0 | 2.559626 | -0.885447 | -0.459624 |
| 12 | 6 | 0 | 1.824084 | -3.556287 | -0.688393 |
| 13 | 6 | 0 | -3.557318 | 2.093847 | 1.306515 |
| 14 | 6 | 0 | -1.908525 | 4.485682 | 1.18917 |
| 15 | 6 | 0 | 0.790545 | 3.680187 | 0.377973 |
| 16 | 6 | 0 | -0.510122 | 1.02242 | -3.379305 |
| 17 | 6 | 0 | -0.596966 | -0.393637 | 3.913614 |
| 18 | 6 | 0 | 0.756631 | -8.85246 | -1.607742 |
| 19 | 6 | 0 | -3.833339 | -5.880111 | -5.252313 |
| 20 | 6 | 0 | -4.950113 | -10.338603 | -4.582346 |
| 21 | 6 | 0 | 2.036091 | 5.651026 | -1.34005 |
| 22 | 6 | 0 | 4.657425 | 4.940777 | -2.284301 |
| 23 | 6 | 0 | 2.109893 | 8.273929 | -0.039952 |
| 24 | 6 | 0 | 3.66807 | 8.332924 | 2.35639 |
| 25 | 6 | 0 | 6.278475 | 9.474809 | 2.180732 |
| 26 | 6 | 0 | 8.034313 | 8.931467 | 4.390747 |
| 27 | 6 | 0 | 8.796312 | 6.157483 | 4.450813 |
| 28 | 6 | 0 | 10.347366 | 10.638604 | 4.287675 |
| 29 | 1 | 0 | 1.974382 | 3.525724 | 2.047682 |
| 30 | 8 | 0 | 4.769993 | -0.32255 | -0.070587 |
| 31 | 8 | 0 | 2.813492 | 7.545914 | 4.345621 |
| 32 | 1 | 0 | -3.454147 | -2.203396 | -1.629744 |
| 33 | 6 | 0 | 1.776822 | -7.988554 | 6.820216 |
| 34 | 8 | 0 | -0.039531 | -8.582568 | 8.052795 |
| 35 | 8 | 0 | 4.034528 | -7.598602 | 7.995373 |
| 36 | 1 | 0 | 3.247052 | -8.849773 | 3.191696 |
| 37 | 1 | 0 | 2.448822 | -5.687593 | 3.602573 |
| 38 | 1 | 0 | -4.122056 | -9.789468 | -0.028396 |
| 39 | 1 | 0 | -1.18249 | -10.080691 | 2.886121 |
| 40 | 1 | 0 | -2.169646 | -7.154296 | 3.952672 |
| 41 | 1 | 0 | -6.945717 | -7.229057 | 1.774255 |
| 42 | 1 | 0 | -7.161682 | -5.580845 | -1.042619 |
| 43 | 1 | 0 | -4.261643 | -4.175307 | 3.647915 |
| 44 | 1 | 0 | -6.349314 | -2.390371 | 1.862888 |
| 45 | 1 | 0 | 3.39592 | -4.821471 | -1.002477 |
| 46 | 1 | 0 | -4.840081 | 2.002268 | -0.303404 |
| 47 | 1 | 0 | -4.732268 | 2.036506 | 2.994164 |
| 48 | 1 | 0 | -1.839444 | 5.462583 | 2.993907 |
| 49 | 1 | 0 | -2.699833 | 5.818896 | -0.167195 |
| 50 | 1 | 0 | -0.934234 | -0.844057 | -4.113682 |
| 51 | 1 | 0 | -2.18116 | 2.196161 | -3.601268 |
| 52 | 1 | 0 | 0.952505 | 1.81026 | -4.584749 |
| 53 | 1 | 0 | 0.011038 | 1.40135 | 4.696375 |
| 54 | 1 | 0 | -2.04571 | -1.126309 | 5.172858 |
| 55 | 1 | 0 | 1.007508 | -1.671842 | 3.999146 |
| 56 | 1 | 0 | 2.694894 | -8.755142 | -0.93987 |
| 57 | 1 | 0 | 0.177176 | -10.82417 | -1.52585 |
| 58 | 1 | 0 | 0.752709 | -8.286847 | -3.580097 |
| 59 | 1 | 0 | -5.131332 | -4.353962 | -4.767532 |
| 60 | 1 | 0 | -1.929122 | -5.117854 | -5.058647 |
| 61 | 1 | 0 | -4.133026 | -6.343056 | -7.23098 |
| 62 | 1 | 0 | -5.292028 | -10.565859 | -6.586365 |
| 63 | 1 | 0 | -5.240515 | -11.993021 | -3.415109 |
| 64 | 1 | 0 | 0.81384 | 5.91967 | -2.984915 |
| 65 | 1 | 0 | 5.463912 | 6.481697 | -3.391263 |
| 66 | 1 | 0 | 4.623381 | 3.271084 | -3.474607 |
| 67 | 1 | 0 | 5.93431 | 4.51134 | -0.735955 |
| 68 | 1 | 0 | 2.849538 | 9.639156 | -1.395041 |
| 69 | 1 | 0 | 0.200248 | 8.850727 | 0.456681 |
| 70 | 1 | 0 | 7.143396 | 8.908702 | 0.39386 |
| 71 | 1 | 0 | 5.971337 | 11.516355 | 1.985747 |
| 72 | 1 | 0 | 6.989656 | 9.341424 | 6.122195 |
| 73 | 1 | 0 | 10.018165 | 5.766993 | 6.0616 |
| 74 | 1 | 0 | 7.158272 | 4.925988 | 4.596723 |
| 75 | 1 | 0 | 9.83464 | 5.639709 | 2.745399 |
| 76 | 1 | 0 | 11.603857 | 10.275169 | 5.878188 |
| 77 | 1 | 0 | 9.82675 | 12.631686 | 4.34205 |
| 78 | 1 | 0 | 11.428283 | 10.320458 | 2.559735 |
| 79 | 1 | 0 | 5.294232 | -7.089438 | 6.772702 |

| C3000007_en_ | | Standard Orientation (Ångstroms) | | | |
| --- | --- | --- | --- | --- | --- |
| Center number | Atomic number | Atomic Type | X | Y | Z |
| 0 | 6 | 0 | 1.956334 | -8.309068 | 3.508083 |
| 1 | 6 | 0 | -4.581781 | -7.554058 | -3.783149 |
| 2 | 6 | 0 | -3.798198 | -8.044383 | -1.076419 |
| 3 | 6 | 0 | -0.989095 | -7.549213 | -0.402518 |
| 4 | 6 | 0 | -0.553025 | -8.852616 | 2.207506 |
| 5 | 6 | 0 | -5.666087 | -6.69654 | 0.757713 |
| 6 | 6 | 0 | -4.554425 | -4.433141 | 2.164101 |
| 7 | 6 | 0 | -2.625721 | -3.037854 | 0.556596 |
| 8 | 6 | 0 | -0.475213 | -4.73647 | -0.233867 |
| 9 | 6 | 0 | -1.651067 | -0.550836 | 1.672328 |
| 10 | 6 | 0 | 0.275012 | 0.702903 | -0.209676 |
| 11 | 6 | 0 | 2.497755 | -1.094056 | -0.264098 |
| 12 | 6 | 0 | 1.816487 | -3.752842 | -0.72298 |
| 13 | 6 | 0 | -3.607028 | 1.545905 | 1.980396 |
| 14 | 6 | 0 | -2.059963 | 4.007799 | 1.910781 |
| 15 | 6 | 0 | 0.630718 | 3.360188 | 0.940458 |
| 16 | 6 | 0 | -0.772925 | 0.919946 | -2.903484 |
| 17 | 6 | 0 | -0.425588 | -1.002646 | 4.257201 |
| 18 | 6 | 0 | 0.746555 | -8.892913 | -2.28752 |
| 19 | 6 | 0 | -6.732842 | -9.175835 | -4.656002 |
| 20 | 6 | 0 | -3.563996 | -5.842946 | -5.312816 |
| 21 | 6 | 0 | 1.748685 | 5.477621 | -0.70062 |
| 22 | 6 | 0 | 4.338155 | 4.876543 | -1.803952 |
| 23 | 6 | 0 | 1.965051 | 7.926764 | 0.869494 |
| 24 | 6 | 0 | -0.374112 | 9.522662 | 1.172909 |
| 25 | 6 | 0 | -1.641812 | 10.448581 | -1.228978 |
| 26 | 6 | 0 | -2.543857 | 13.185122 | -1.084904 |
| 27 | 6 | 0 | -0.319581 | 14.992724 | -0.83791 |
| 28 | 6 | 0 | -4.168706 | 13.837868 | -3.364717 |
| 29 | 1 | 0 | 1.908796 | 3.158045 | 2.541586 |
| 30 | 8 | 0 | 4.702339 | -0.512374 | 0.124633 |
| 31 | 8 | 0 | -1.173301 | 10.132374 | 3.242551 |
| 32 | 1 | 0 | -3.622354 | -2.556551 | -1.192143 |
| 33 | 6 | 0 | 1.675646 | -7.108606 | 6.093177 |
| 34 | 8 | 0 | -0.257065 | -6.992586 | 7.28932 |
| 35 | 8 | 0 | 3.83825 | -6.160854 | 7.101894 |
| 36 | 1 | 0 | 3.04227 | -10.041976 | 3.785009 |
| 37 | 1 | 0 | 3.144063 | -7.082249 | 2.358058 |
| 38 | 1 | 0 | -4.053634 | -10.075 | -0.793826 |
| 39 | 1 | 0 | -0.732705 | -10.875585 | 1.865801 |
| 40 | 1 | 0 | -2.051423 | -8.375006 | 3.516974 |
| 41 | 1 | 0 | -6.409008 | -8.046298 | 2.121937 |
| 42 | 1 | 0 | -7.287051 | -6.05459 | -0.335396 |
| 43 | 1 | 0 | -3.709763 | -5.022847 | 3.937706 |
| 44 | 1 | 0 | -6.074976 | -3.138427 | 2.66574 |
| 45 | 1 | 0 | 3.383929 | -4.925988 | -1.307769 |
| 46 | 1 | 0 | -4.972513 | 1.493766 | 0.439771 |
| 47 | 1 | 0 | -4.674417 | 1.344733 | 3.726228 |
| 48 | 1 | 0 | -1.983432 | 4.925105 | 3.745326 |
| 49 | 1 | 0 | -2.965255 | 5.352658 | 0.645961 |
| 50 | 1 | 0 | -1.198297 | -0.900309 | -3.73929 |
| 51 | 1 | 0 | -2.483521 | 2.056571 | -2.959081 |
| 52 | 1 | 0 | 0.603677 | 1.824947 | -4.127843 |
| 53 | 1 | 0 | 0.200788 | 0.752409 | 5.112568 |
| 54 | 1 | 0 | -1.776756 | -1.844289 | 5.5519 |
| 55 | 1 | 0 | 1.199576 | -2.252426 | 4.170159 |
| 56 | 1 | 0 | 2.677147 | -9.019666 | -1.590079 |
| 57 | 1 | 0 | 0.080445 | -10.819993 | -2.574831 |
| 58 | 1 | 0 | 0.795268 | -7.971136 | -4.115562 |
| 59 | 1 | 0 | -6.161551 | -11.15833 | -4.719557 |
| 60 | 1 | 0 | -8.333325 | -9.075633 | -3.359075 |
| 61 | 1 | 0 | -7.392492 | -8.640471 | -6.526561 |
| 62 | 1 | 0 | -4.275995 | -5.566812 | -7.209585 |
| 63 | 1 | 0 | -1.98649 | -4.672776 | -4.767589 |
| 64 | 1 | 0 | 0.454422 | 5.831735 | -2.269976 |
| 65 | 1 | 0 | 4.99193 | 6.44424 | -2.970697 |
| 66 | 1 | 0 | 4.330303 | 3.188085 | -2.966514 |
| 67 | 1 | 0 | 5.720243 | 4.557112 | -0.316174 |
| 68 | 1 | 0 | 2.717471 | 7.509108 | 2.739009 |
| 69 | 1 | 0 | 3.331259 | 9.163211 | -0.070457 |
| 70 | 1 | 0 | -0.389318 | 10.187853 | -2.848658 |
| 71 | 1 | 0 | -3.262241 | 9.209187 | -1.558506 |
| 72 | 1 | 0 | -3.694841 | 13.350256 | 0.618518 |
| 73 | 1 | 0 | -0.963969 | 16.942842 | -0.693827 |
| 74 | 1 | 0 | 0.803006 | 14.587846 | 0.838237 |
| 75 | 1 | 0 | 0.919613 | 14.870445 | -2.483522 |
| 76 | 1 | 0 | -4.860399 | 15.774258 | -3.250627 |
| 77 | 1 | 0 | -5.805246 | 12.594893 | -3.509041 |
| 78 | 1 | 0 | -3.09521 | 13.666402 | -5.117764 |
| 79 | 1 | 0 | 5.205435 | -6.296093 | 5.896315 |

| C3000007_tddft_ | | Standard Orientation (Ångstroms) | | | |
| --- | --- | --- | --- | --- | --- |
| Center number | Atomic number | Atomic Type | X | Y | Z |
| 0 | 6 | 0 | 1.956334 | -8.309068 | 3.508083 |
| 1 | 6 | 0 | -4.581781 | -7.554058 | -3.783149 |
| 2 | 6 | 0 | -3.798198 | -8.044383 | -1.076419 |
| 3 | 6 | 0 | -0.989095 | -7.549213 | -0.402518 |
| 4 | 6 | 0 | -0.553025 | -8.852616 | 2.207506 |
| 5 | 6 | 0 | -5.666087 | -6.69654 | 0.757713 |
| 6 | 6 | 0 | -4.554425 | -4.433141 | 2.164101 |
| 7 | 6 | 0 | -2.625721 | -3.037854 | 0.556596 |
| 8 | 6 | 0 | -0.475213 | -4.73647 | -0.233867 |
| 9 | 6 | 0 | -1.651067 | -0.550836 | 1.672328 |
| 10 | 6 | 0 | 0.275012 | 0.702903 | -0.209676 |
| 11 | 6 | 0 | 2.497755 | -1.094056 | -0.264098 |
| 12 | 6 | 0 | 1.816487 | -3.752842 | -0.72298 |
| 13 | 6 | 0 | -3.607028 | 1.545905 | 1.980396 |
| 14 | 6 | 0 | -2.059963 | 4.007799 | 1.910781 |
| 15 | 6 | 0 | 0.630718 | 3.360188 | 0.940458 |
| 16 | 6 | 0 | -0.772925 | 0.919946 | -2.903484 |
| 17 | 6 | 0 | -0.425588 | -1.002646 | 4.257201 |
| 18 | 6 | 0 | 0.746555 | -8.892913 | -2.28752 |
| 19 | 6 | 0 | -6.732842 | -9.175835 | -4.656002 |
| 20 | 6 | 0 | -3.563996 | -5.842946 | -5.312816 |
| 21 | 6 | 0 | 1.748685 | 5.477621 | -0.70062 |
| 22 | 6 | 0 | 4.338155 | 4.876543 | -1.803952 |
| 23 | 6 | 0 | 1.965051 | 7.926764 | 0.869494 |
| 24 | 6 | 0 | -0.374112 | 9.522662 | 1.172909 |
| 25 | 6 | 0 | -1.641812 | 10.448581 | -1.228978 |
| 26 | 6 | 0 | -2.543857 | 13.185122 | -1.084904 |
| 27 | 6 | 0 | -0.319581 | 14.992724 | -0.83791 |
| 28 | 6 | 0 | -4.168706 | 13.837868 | -3.364717 |
| 29 | 1 | 0 | 1.908796 | 3.158045 | 2.541586 |
| 30 | 8 | 0 | 4.702339 | -0.512374 | 0.124633 |
| 31 | 8 | 0 | -1.173301 | 10.132374 | 3.242551 |
| 32 | 1 | 0 | -3.622354 | -2.556551 | -1.192143 |
| 33 | 6 | 0 | 1.675646 | -7.108606 | 6.093177 |
| 34 | 8 | 0 | -0.257065 | -6.992586 | 7.28932 |
| 35 | 8 | 0 | 3.83825 | -6.160854 | 7.101894 |
| 36 | 1 | 0 | 3.04227 | -10.041976 | 3.785009 |
| 37 | 1 | 0 | 3.144063 | -7.082249 | 2.358058 |
| 38 | 1 | 0 | -4.053634 | -10.075 | -0.793826 |
| 39 | 1 | 0 | -0.732705 | -10.875585 | 1.865801 |
| 40 | 1 | 0 | -2.051423 | -8.375006 | 3.516974 |
| 41 | 1 | 0 | -6.409008 | -8.046298 | 2.121937 |
| 42 | 1 | 0 | -7.287051 | -6.05459 | -0.335396 |
| 43 | 1 | 0 | -3.709763 | -5.022847 | 3.937706 |
| 44 | 1 | 0 | -6.074976 | -3.138427 | 2.66574 |
| 45 | 1 | 0 | 3.383929 | -4.925988 | -1.307769 |
| 46 | 1 | 0 | -4.972513 | 1.493766 | 0.439771 |
| 47 | 1 | 0 | -4.674417 | 1.344733 | 3.726228 |
| 48 | 1 | 0 | -1.983432 | 4.925105 | 3.745326 |
| 49 | 1 | 0 | -2.965255 | 5.352658 | 0.645961 |
| 50 | 1 | 0 | -1.198297 | -0.900309 | -3.73929 |
| 51 | 1 | 0 | -2.483521 | 2.056571 | -2.959081 |
| 52 | 1 | 0 | 0.603677 | 1.824947 | -4.127843 |
| 53 | 1 | 0 | 0.200788 | 0.752409 | 5.112568 |
| 54 | 1 | 0 | -1.776756 | -1.844289 | 5.5519 |
| 55 | 1 | 0 | 1.199576 | -2.252426 | 4.170159 |
| 56 | 1 | 0 | 2.677147 | -9.019666 | -1.590079 |
| 57 | 1 | 0 | 0.080445 | -10.819993 | -2.574831 |
| 58 | 1 | 0 | 0.795268 | -7.971136 | -4.115562 |
| 59 | 1 | 0 | -6.161551 | -11.15833 | -4.719557 |
| 60 | 1 | 0 | -8.333325 | -9.075633 | -3.359075 |
| 61 | 1 | 0 | -7.392492 | -8.640471 | -6.526561 |
| 62 | 1 | 0 | -4.275995 | -5.566812 | -7.209585 |
| 63 | 1 | 0 | -1.98649 | -4.672776 | -4.767589 |
| 64 | 1 | 0 | 0.454422 | 5.831735 | -2.269976 |
| 65 | 1 | 0 | 4.99193 | 6.44424 | -2.970697 |
| 66 | 1 | 0 | 4.330303 | 3.188085 | -2.966514 |
| 67 | 1 | 0 | 5.720243 | 4.557112 | -0.316174 |
| 68 | 1 | 0 | 2.717471 | 7.509108 | 2.739009 |
| 69 | 1 | 0 | 3.331259 | 9.163211 | -0.070457 |
| 70 | 1 | 0 | -0.389318 | 10.187853 | -2.848658 |
| 71 | 1 | 0 | -3.262241 | 9.209187 | -1.558506 |
| 72 | 1 | 0 | -3.694841 | 13.350256 | 0.618518 |
| 73 | 1 | 0 | -0.963969 | 16.942842 | -0.693827 |
| 74 | 1 | 0 | 0.803006 | 14.587846 | 0.838237 |
| 75 | 1 | 0 | 0.919613 | 14.870445 | -2.483522 |
| 76 | 1 | 0 | -4.860399 | 15.774258 | -3.250627 |
| 77 | 1 | 0 | -5.805246 | 12.594893 | -3.509041 |
| 78 | 1 | 0 | -3.09521 | 13.666402 | -5.117764 |
| 79 | 1 | 0 | 5.205435 | -6.296093 | 5.896315 |

| C3000008_en_ | | Standard Orientation (Ångstroms) | | | |
| --- | --- | --- | --- | --- | --- |
| Center number | Atomic number | Atomic Type | X | Y | Z |
| 0 | 6 | 0 | 1.482085 | -8.118318 | 4.141739 |
| 1 | 6 | 0 | -5.009467 | -7.811772 | -3.201837 |
| 2 | 6 | 0 | -4.288489 | -7.866096 | -0.432973 |
| 3 | 6 | 0 | -1.437042 | -7.619348 | 0.195308 |
| 4 | 6 | 0 | -1.160503 | -8.290353 | 3.04648 |
| 5 | 6 | 0 | -5.954927 | -5.957554 | 1.057782 |
| 6 | 6 | 0 | -4.580072 | -3.576997 | 1.945738 |
| 7 | 6 | 0 | -2.441584 | -2.824907 | 0.182289 |
| 8 | 6 | 0 | -0.551829 | -4.928234 | -0.226816 |
| 9 | 6 | 0 | -1.102149 | -0.35732 | 0.894862 |
| 10 | 6 | 0 | 0.972029 | 0.269348 | -1.130978 |
| 11 | 6 | 0 | 2.913362 | -1.810685 | -0.868702 |
| 12 | 6 | 0 | 1.854388 | -4.382932 | -0.838871 |
| 13 | 6 | 0 | -2.71301 | 2.032964 | 0.822609 |
| 14 | 6 | 0 | -0.795851 | 4.185781 | 0.449741 |
| 15 | 6 | 0 | 1.740536 | 2.996568 | -0.431973 |
| 16 | 6 | 0 | -0.064398 | 0.17578 | -3.837849 |
| 17 | 6 | 0 | 0.053505 | -0.571052 | 3.54533 |
| 18 | 6 | 0 | 0.072445 | -9.572156 | -1.306541 |
| 19 | 6 | 0 | -7.359922 | -9.276542 | -3.793454 |
| 20 | 6 | 0 | -3.776019 | -6.587424 | -5.014249 |
| 21 | 6 | 0 | 3.060221 | 4.626037 | -2.432351 |
| 22 | 6 | 0 | 5.517969 | 3.523985 | -3.442661 |
| 23 | 6 | 0 | 3.578222 | 7.266425 | -1.334797 |
| 24 | 6 | 0 | 4.486839 | 9.090606 | -3.340368 |
| 25 | 6 | 0 | 7.123062 | 10.169686 | -3.145477 |
| 26 | 6 | 0 | 7.105203 | 12.895377 | -2.141158 |
| 27 | 6 | 0 | 5.968784 | 13.06702 | 0.495966 |
| 28 | 6 | 0 | 9.762953 | 13.989486 | -2.194631 |
| 29 | 1 | 0 | 3.0402 | 2.869726 | 1.162233 |
| 30 | 8 | 0 | 5.18715 | -1.486402 | -0.596654 |
| 31 | 8 | 0 | 3.092956 | 9.709046 | -5.06661 |
| 32 | 1 | 0 | -3.338439 | -2.50781 | -1.654486 |
| 33 | 6 | 0 | 1.482988 | -7.797924 | 6.98443 |
| 34 | 8 | 0 | -0.35116 | -7.394887 | 8.26778 |
| 35 | 8 | 0 | 3.798312 | -7.942832 | 8.098024 |
| 36 | 1 | 0 | 2.624586 | -9.765793 | 3.658038 |
| 37 | 1 | 0 | 2.479925 | -6.491305 | 3.355192 |
| 38 | 1 | 0 | -4.81506 | -9.749952 | 0.231749 |
| 39 | 1 | 0 | -1.870149 | -10.210743 | 3.292071 |
| 40 | 1 | 0 | -2.374686 | -7.104862 | 4.186797 |
| 41 | 1 | 0 | -6.799736 | -6.892265 | 2.684987 |
| 42 | 1 | 0 | -7.525585 | -5.396992 | -0.146744 |
| 43 | 1 | 0 | -3.868812 | -3.83065 | 3.854867 |
| 44 | 1 | 0 | -5.923518 | -2.021159 | 2.0523 |
| 45 | 1 | 0 | 3.238064 | -5.85844 | -1.123753 |
| 46 | 1 | 0 | -4.045629 | 1.959934 | -0.744892 |
| 47 | 1 | 0 | -3.828233 | 2.266015 | 2.535515 |
| 48 | 1 | 0 | -0.539555 | 5.261684 | 2.182779 |
| 49 | 1 | 0 | -1.483745 | 5.517761 | -0.961142 |
| 50 | 1 | 0 | -0.716728 | -1.69347 | -4.364476 |
| 51 | 1 | 0 | -1.620258 | 1.490973 | -4.097374 |
| 52 | 1 | 0 | 1.400643 | 0.692086 | -5.177813 |
| 53 | 1 | 0 | 1.016908 | 1.153063 | 4.095436 |
| 54 | 1 | 0 | -1.421156 | -0.898268 | 4.937684 |
| 55 | 1 | 0 | 1.406025 | -2.106439 | 3.706776 |
| 56 | 1 | 0 | 2.027635 | -9.68182 | -0.688405 |
| 57 | 1 | 0 | -0.756788 | -11.435904 | -1.025618 |
| 58 | 1 | 0 | 0.068936 | -9.180188 | -3.316458 |
| 59 | 1 | 0 | -7.046675 | -11.293151 | -3.482746 |
| 60 | 1 | 0 | -8.924881 | -8.737883 | -2.563253 |
| 61 | 1 | 0 | -7.957883 | -9.016371 | -5.741486 |
| 62 | 1 | 0 | -4.455968 | -6.598022 | -6.942645 |
| 63 | 1 | 0 | -2.040622 | -5.569791 | -4.683195 |
| 64 | 1 | 0 | 1.760925 | 4.909851 | -4.010383 |
| 65 | 1 | 0 | 6.350884 | 4.779902 | -4.847165 |
| 66 | 1 | 0 | 5.254626 | 1.69534 | -4.331135 |
| 67 | 1 | 0 | 6.881398 | 3.245689 | -1.928174 |
| 68 | 1 | 0 | 1.842534 | 8.05778 | -0.563338 |
| 69 | 1 | 0 | 4.940686 | 7.118768 | 0.204222 |
| 70 | 1 | 0 | 8.30625 | 8.995938 | -1.931447 |
| 71 | 1 | 0 | 7.938307 | 10.168328 | -5.03703 |
| 72 | 1 | 0 | 5.920703 | 13.991827 | -3.429539 |
| 73 | 1 | 0 | 5.987592 | 15.00963 | 1.177262 |
| 74 | 1 | 0 | 4.016465 | 12.41777 | 0.536555 |
| 75 | 1 | 0 | 7.039908 | 11.925595 | 1.838631 |
| 76 | 1 | 0 | 9.766254 | 15.949015 | -1.560982 |
| 77 | 1 | 0 | 10.564604 | 13.938228 | -4.090311 |
| 78 | 1 | 0 | 11.021144 | 12.930568 | -0.950441 |
| 79 | 1 | 0 | 5.081565 | -8.22905 | 6.82827 |

| C3000008_tddft_ | | Standard Orientation (Ångstroms) | | | |
| --- | --- | --- | --- | --- | --- |
| Center number | Atomic number | Atomic Type | X | Y | Z |
| 0 | 6 | 0 | 1.482085 | -8.118318 | 4.141739 |
| 1 | 6 | 0 | -5.009467 | -7.811772 | -3.201837 |
| 2 | 6 | 0 | -4.288489 | -7.866096 | -0.432973 |
| 3 | 6 | 0 | -1.437042 | -7.619348 | 0.195308 |
| 4 | 6 | 0 | -1.160503 | -8.290353 | 3.04648 |
| 5 | 6 | 0 | -5.954927 | -5.957554 | 1.057782 |
| 6 | 6 | 0 | -4.580072 | -3.576997 | 1.945738 |
| 7 | 6 | 0 | -2.441584 | -2.824907 | 0.182289 |
| 8 | 6 | 0 | -0.551829 | -4.928234 | -0.226816 |
| 9 | 6 | 0 | -1.102149 | -0.35732 | 0.894862 |
| 10 | 6 | 0 | 0.972029 | 0.269348 | -1.130978 |
| 11 | 6 | 0 | 2.913362 | -1.810685 | -0.868702 |
| 12 | 6 | 0 | 1.854388 | -4.382932 | -0.838871 |
| 13 | 6 | 0 | -2.71301 | 2.032964 | 0.822609 |
| 14 | 6 | 0 | -0.795851 | 4.185781 | 0.449741 |
| 15 | 6 | 0 | 1.740536 | 2.996568 | -0.431973 |
| 16 | 6 | 0 | -0.064398 | 0.17578 | -3.837849 |
| 17 | 6 | 0 | 0.053505 | -0.571052 | 3.54533 |
| 18 | 6 | 0 | 0.072445 | -9.572156 | -1.306541 |
| 19 | 6 | 0 | -7.359922 | -9.276542 | -3.793454 |
| 20 | 6 | 0 | -3.776019 | -6.587424 | -5.014249 |
| 21 | 6 | 0 | 3.060221 | 4.626037 | -2.432351 |
| 22 | 6 | 0 | 5.517969 | 3.523985 | -3.442661 |
| 23 | 6 | 0 | 3.578222 | 7.266425 | -1.334797 |
| 24 | 6 | 0 | 4.486839 | 9.090606 | -3.340368 |
| 25 | 6 | 0 | 7.123062 | 10.169686 | -3.145477 |
| 26 | 6 | 0 | 7.105203 | 12.895377 | -2.141158 |
| 27 | 6 | 0 | 5.968784 | 13.06702 | 0.495966 |
| 28 | 6 | 0 | 9.762953 | 13.989486 | -2.194631 |
| 29 | 1 | 0 | 3.0402 | 2.869726 | 1.162233 |
| 30 | 8 | 0 | 5.18715 | -1.486402 | -0.596654 |
| 31 | 8 | 0 | 3.092956 | 9.709046 | -5.06661 |
| 32 | 1 | 0 | -3.338439 | -2.50781 | -1.654486 |
| 33 | 6 | 0 | 1.482988 | -7.797924 | 6.98443 |
| 34 | 8 | 0 | -0.35116 | -7.394887 | 8.26778 |
| 35 | 8 | 0 | 3.798312 | -7.942832 | 8.098024 |
| 36 | 1 | 0 | 2.624586 | -9.765793 | 3.658038 |
| 37 | 1 | 0 | 2.479925 | -6.491305 | 3.355192 |
| 38 | 1 | 0 | -4.81506 | -9.749952 | 0.231749 |
| 39 | 1 | 0 | -1.870149 | -10.210743 | 3.292071 |
| 40 | 1 | 0 | -2.374686 | -7.104862 | 4.186797 |
| 41 | 1 | 0 | -6.799736 | -6.892265 | 2.684987 |
| 42 | 1 | 0 | -7.525585 | -5.396992 | -0.146744 |
| 43 | 1 | 0 | -3.868812 | -3.83065 | 3.854867 |
| 44 | 1 | 0 | -5.923518 | -2.021159 | 2.0523 |
| 45 | 1 | 0 | 3.238064 | -5.85844 | -1.123753 |
| 46 | 1 | 0 | -4.045629 | 1.959934 | -0.744892 |
| 47 | 1 | 0 | -3.828233 | 2.266015 | 2.535515 |
| 48 | 1 | 0 | -0.539555 | 5.261684 | 2.182779 |
| 49 | 1 | 0 | -1.483745 | 5.517761 | -0.961142 |
| 50 | 1 | 0 | -0.716728 | -1.69347 | -4.364476 |
| 51 | 1 | 0 | -1.620258 | 1.490973 | -4.097374 |
| 52 | 1 | 0 | 1.400643 | 0.692086 | -5.177813 |
| 53 | 1 | 0 | 1.016908 | 1.153063 | 4.095436 |
| 54 | 1 | 0 | -1.421156 | -0.898268 | 4.937684 |
| 55 | 1 | 0 | 1.406025 | -2.106439 | 3.706776 |
| 56 | 1 | 0 | 2.027635 | -9.68182 | -0.688405 |
| 57 | 1 | 0 | -0.756788 | -11.435904 | -1.025618 |
| 58 | 1 | 0 | 0.068936 | -9.180188 | -3.316458 |
| 59 | 1 | 0 | -7.046675 | -11.293151 | -3.482746 |
| 60 | 1 | 0 | -8.924881 | -8.737883 | -2.563253 |
| 61 | 1 | 0 | -7.957883 | -9.016371 | -5.741486 |
| 62 | 1 | 0 | -4.455968 | -6.598022 | -6.942645 |
| 63 | 1 | 0 | -2.040622 | -5.569791 | -4.683195 |
| 64 | 1 | 0 | 1.760925 | 4.909851 | -4.010383 |
| 65 | 1 | 0 | 6.350884 | 4.779902 | -4.847165 |
| 66 | 1 | 0 | 5.254626 | 1.69534 | -4.331135 |
| 67 | 1 | 0 | 6.881398 | 3.245689 | -1.928174 |
| 68 | 1 | 0 | 1.842534 | 8.05778 | -0.563338 |
| 69 | 1 | 0 | 4.940686 | 7.118768 | 0.204222 |
| 70 | 1 | 0 | 8.30625 | 8.995938 | -1.931447 |
| 71 | 1 | 0 | 7.938307 | 10.168328 | -5.03703 |
| 72 | 1 | 0 | 5.920703 | 13.991827 | -3.429539 |
| 73 | 1 | 0 | 5.987592 | 15.00963 | 1.177262 |
| 74 | 1 | 0 | 4.016465 | 12.41777 | 0.536555 |
| 75 | 1 | 0 | 7.039908 | 11.925595 | 1.838631 |
| 76 | 1 | 0 | 9.766254 | 15.949015 | -1.560982 |
| 77 | 1 | 0 | 10.564604 | 13.938228 | -4.090311 |
| 78 | 1 | 0 | 11.021144 | 12.930568 | -0.950441 |
| 79 | 1 | 0 | 5.081565 | -8.22905 | 6.82827 |

| C3000009_en_ | | Standard Orientation (Ångstroms) | | | |
| --- | --- | --- | --- | --- | --- |
| Center number | Atomic number | Atomic Type | X | Y | Z |
| 0 | 6 | 0 | 0.584323 | -7.979925 | 4.68858 |
| 1 | 6 | 0 | -4.029816 | -8.145185 | -3.847024 |
| 2 | 6 | 0 | -4.088574 | -7.751608 | -1.018304 |
| 3 | 6 | 0 | -1.421251 | -7.463399 | 0.211206 |
| 4 | 6 | 0 | -1.766032 | -8.112037 | 3.058526 |
| 5 | 6 | 0 | -5.978217 | -5.635382 | -0.271607 |
| 6 | 6 | 0 | -4.844371 | -3.362185 | 1.087042 |
| 7 | 6 | 0 | -2.317458 | -2.637273 | -0.062884 |
| 8 | 6 | 0 | -0.430695 | -4.783801 | -0.029028 |
| 9 | 6 | 0 | -1.136496 | -0.197082 | 0.946429 |
| 10 | 6 | 0 | 1.349916 | 0.390856 | -0.562217 |
| 11 | 6 | 0 | 3.144614 | -1.72402 | 0.128161 |
| 12 | 6 | 0 | 2.060422 | -4.277646 | -0.090738 |
| 13 | 6 | 0 | -2.650536 | 2.217002 | 0.516704 |
| 14 | 6 | 0 | -0.665117 | 4.337857 | 0.599137 |
| 15 | 6 | 0 | 1.986061 | 3.108634 | 0.29628 |
| 16 | 6 | 0 | 0.952971 | 0.304679 | -3.435237 |
| 17 | 6 | 0 | -0.618871 | -0.431904 | 3.787937 |
| 18 | 6 | 0 | 0.358598 | -9.423736 | -0.940441 |
| 19 | 6 | 0 | -3.015555 | -6.097347 | -5.509338 |
| 20 | 6 | 0 | -4.919379 | -10.278392 | -4.8379 |
| 21 | 6 | 0 | 3.745341 | 4.711078 | -1.361481 |
| 22 | 6 | 0 | 6.33889 | 3.56029 | -1.814372 |
| 23 | 6 | 0 | 4.072729 | 7.352226 | -0.160359 |
| 24 | 6 | 0 | 5.128168 | 9.230776 | -2.02296 |
| 25 | 6 | 0 | 3.256836 | 10.341437 | -3.88855 |
| 26 | 6 | 0 | 4.360091 | 11.032176 | -6.455856 |
| 27 | 6 | 0 | 5.131775 | 8.671188 | -7.907545 |
| 28 | 6 | 0 | 2.49983 | 12.617054 | -7.971973 |
| 29 | 1 | 0 | 2.896374 | 2.969911 | 2.138401 |
| 30 | 8 | 0 | 5.299747 | -1.439811 | 0.914625 |
| 31 | 8 | 0 | 7.34373 | 9.84698 | -2.027602 |
| 32 | 1 | 0 | -2.730303 | -2.289127 | -2.056203 |
| 33 | 6 | 0 | -0.013259 | -7.953867 | 7.484822 |
| 34 | 8 | 0 | -2.099336 | -7.874578 | 8.386815 |
| 35 | 8 | 0 | 2.037904 | -7.997075 | 9.039552 |
| 36 | 1 | 0 | 1.856315 | -9.559061 | 4.308783 |
| 37 | 1 | 0 | 1.669042 | -6.275782 | 4.269353 |
| 38 | 1 | 0 | -4.816207 | -9.516175 | -0.246581 |
| 39 | 1 | 0 | -2.535261 | -10.021744 | 3.155412 |
| 40 | 1 | 0 | -3.192313 | -6.912074 | 3.903659 |
| 41 | 1 | 0 | -7.478203 | -6.444733 | 0.880297 |
| 42 | 1 | 0 | -6.908184 | -4.9657 | -1.982362 |
| 43 | 1 | 0 | -4.655639 | -3.719213 | 3.099419 |
| 44 | 1 | 0 | -6.140461 | -1.770096 | 0.921153 |
| 45 | 1 | 0 | 3.451577 | -5.772195 | -0.051541 |
| 46 | 1 | 0 | -3.592437 | 2.164338 | -1.313792 |
| 47 | 1 | 0 | -4.123211 | 2.471321 | 1.930162 |
| 48 | 1 | 0 | -0.786106 | 5.397141 | 2.356714 |
| 49 | 1 | 0 | -1.0084 | 5.690984 | -0.915021 |
| 50 | 1 | 0 | 0.411134 | -1.556497 | -4.101866 |
| 51 | 1 | 0 | -0.478316 | 1.645492 | -4.047177 |
| 52 | 1 | 0 | 2.695074 | 0.789279 | -4.403914 |
| 53 | 1 | 0 | 0.225379 | 1.274187 | 4.551077 |
| 54 | 1 | 0 | -2.377723 | -0.736709 | 4.805304 |
| 55 | 1 | 0 | 0.639343 | -1.98753 | 4.243245 |
| 56 | 1 | 0 | 2.150572 | -9.530541 | 0.053767 |
| 57 | 1 | 0 | -0.511703 | -11.285069 | -0.849372 |
| 58 | 1 | 0 | 0.767408 | -9.028067 | -2.911579 |
| 59 | 1 | 0 | -4.099713 | -4.35528 | -5.319172 |
| 60 | 1 | 0 | -1.0682 | -5.631109 | -5.026986 |
| 61 | 1 | 0 | -3.064017 | -6.647389 | -7.487407 |
| 62 | 1 | 0 | -4.942122 | -10.602599 | -6.857432 |
| 63 | 1 | 0 | -5.664424 | -11.781641 | -3.66768 |
| 64 | 1 | 0 | 2.808779 | 4.974335 | -3.18531 |
| 65 | 1 | 0 | 7.52116 | 4.83529 | -2.911119 |
| 66 | 1 | 0 | 6.236823 | 1.770264 | -2.810173 |
| 67 | 1 | 0 | 7.290825 | 3.189856 | -0.032384 |
| 68 | 1 | 0 | 2.254446 | 8.055217 | 0.503693 |
| 69 | 1 | 0 | 5.346884 | 7.216655 | 1.450045 |
| 70 | 1 | 0 | 1.642918 | 9.074343 | -4.114619 |
| 71 | 1 | 0 | 2.502308 | 12.034597 | -2.964765 |
| 72 | 1 | 0 | 6.05546 | 12.154603 | -6.110424 |
| 73 | 1 | 0 | 5.952433 | 9.158386 | -9.731735 |
| 74 | 1 | 0 | 6.52586 | 7.567033 | -6.875809 |
| 75 | 1 | 0 | 3.498172 | 7.46205 | -8.265353 |
| 76 | 1 | 0 | 3.299617 | 13.160755 | -9.790182 |
| 77 | 1 | 0 | 1.98234 | 14.343634 | -6.973914 |
| 78 | 1 | 0 | 0.763469 | 11.568507 | -8.349878 |
| 79 | 1 | 0 | 3.572595 | -8.015957 | 8.046826 |

| C3000009_tddft_ | | Standard Orientation (Ångstroms) | | | |
| --- | --- | --- | --- | --- | --- |
| Center number | Atomic number | Atomic Type | X | Y | Z |
| 0 | 6 | 0 | 0.584323 | -7.979925 | 4.68858 |
| 1 | 6 | 0 | -4.029816 | -8.145185 | -3.847024 |
| 2 | 6 | 0 | -4.088574 | -7.751608 | -1.018304 |
| 3 | 6 | 0 | -1.421251 | -7.463399 | 0.211206 |
| 4 | 6 | 0 | -1.766032 | -8.112037 | 3.058526 |
| 5 | 6 | 0 | -5.978217 | -5.635382 | -0.271607 |
| 6 | 6 | 0 | -4.844371 | -3.362185 | 1.087042 |
| 7 | 6 | 0 | -2.317458 | -2.637273 | -0.062884 |
| 8 | 6 | 0 | -0.430695 | -4.783801 | -0.029028 |
| 9 | 6 | 0 | -1.136496 | -0.197082 | 0.946429 |
| 10 | 6 | 0 | 1.349916 | 0.390856 | -0.562217 |
| 11 | 6 | 0 | 3.144614 | -1.72402 | 0.128161 |
| 12 | 6 | 0 | 2.060422 | -4.277646 | -0.090738 |
| 13 | 6 | 0 | -2.650536 | 2.217002 | 0.516704 |
| 14 | 6 | 0 | -0.665117 | 4.337857 | 0.599137 |
| 15 | 6 | 0 | 1.986061 | 3.108634 | 0.29628 |
| 16 | 6 | 0 | 0.952971 | 0.304679 | -3.435237 |
| 17 | 6 | 0 | -0.618871 | -0.431904 | 3.787937 |
| 18 | 6 | 0 | 0.358598 | -9.423736 | -0.940441 |
| 19 | 6 | 0 | -3.015555 | -6.097347 | -5.509338 |
| 20 | 6 | 0 | -4.919379 | -10.278392 | -4.8379 |
| 21 | 6 | 0 | 3.745341 | 4.711078 | -1.361481 |
| 22 | 6 | 0 | 6.33889 | 3.56029 | -1.814372 |
| 23 | 6 | 0 | 4.072729 | 7.352226 | -0.160359 |
| 24 | 6 | 0 | 5.128168 | 9.230776 | -2.02296 |
| 25 | 6 | 0 | 3.256836 | 10.341437 | -3.88855 |
| 26 | 6 | 0 | 4.360091 | 11.032176 | -6.455856 |
| 27 | 6 | 0 | 5.131775 | 8.671188 | -7.907545 |
| 28 | 6 | 0 | 2.49983 | 12.617054 | -7.971973 |
| 29 | 1 | 0 | 2.896374 | 2.969911 | 2.138401 |
| 30 | 8 | 0 | 5.299747 | -1.439811 | 0.914625 |
| 31 | 8 | 0 | 7.34373 | 9.84698 | -2.027602 |
| 32 | 1 | 0 | -2.730303 | -2.289127 | -2.056203 |
| 33 | 6 | 0 | -0.013259 | -7.953867 | 7.484822 |
| 34 | 8 | 0 | -2.099336 | -7.874578 | 8.386815 |
| 35 | 8 | 0 | 2.037904 | -7.997075 | 9.039552 |
| 36 | 1 | 0 | 1.856315 | -9.559061 | 4.308783 |
| 37 | 1 | 0 | 1.669042 | -6.275782 | 4.269353 |
| 38 | 1 | 0 | -4.816207 | -9.516175 | -0.246581 |
| 39 | 1 | 0 | -2.535261 | -10.021744 | 3.155412 |
| 40 | 1 | 0 | -3.192313 | -6.912074 | 3.903659 |
| 41 | 1 | 0 | -7.478203 | -6.444733 | 0.880297 |
| 42 | 1 | 0 | -6.908184 | -4.9657 | -1.982362 |
| 43 | 1 | 0 | -4.655639 | -3.719213 | 3.099419 |
| 44 | 1 | 0 | -6.140461 | -1.770096 | 0.921153 |
| 45 | 1 | 0 | 3.451577 | -5.772195 | -0.051541 |
| 46 | 1 | 0 | -3.592437 | 2.164338 | -1.313792 |
| 47 | 1 | 0 | -4.123211 | 2.471321 | 1.930162 |
| 48 | 1 | 0 | -0.786106 | 5.397141 | 2.356714 |
| 49 | 1 | 0 | -1.0084 | 5.690984 | -0.915021 |
| 50 | 1 | 0 | 0.411134 | -1.556497 | -4.101866 |
| 51 | 1 | 0 | -0.478316 | 1.645492 | -4.047177 |
| 52 | 1 | 0 | 2.695074 | 0.789279 | -4.403914 |
| 53 | 1 | 0 | 0.225379 | 1.274187 | 4.551077 |
| 54 | 1 | 0 | -2.377723 | -0.736709 | 4.805304 |
| 55 | 1 | 0 | 0.639343 | -1.98753 | 4.243245 |
| 56 | 1 | 0 | 2.150572 | -9.530541 | 0.053767 |
| 57 | 1 | 0 | -0.511703 | -11.285069 | -0.849372 |
| 58 | 1 | 0 | 0.767408 | -9.028067 | -2.911579 |
| 59 | 1 | 0 | -4.099713 | -4.35528 | -5.319172 |
| 60 | 1 | 0 | -1.0682 | -5.631109 | -5.026986 |
| 61 | 1 | 0 | -3.064017 | -6.647389 | -7.487407 |
| 62 | 1 | 0 | -4.942122 | -10.602599 | -6.857432 |
| 63 | 1 | 0 | -5.664424 | -11.781641 | -3.66768 |
| 64 | 1 | 0 | 2.808779 | 4.974335 | -3.18531 |
| 65 | 1 | 0 | 7.52116 | 4.83529 | -2.911119 |
| 66 | 1 | 0 | 6.236823 | 1.770264 | -2.810173 |
| 67 | 1 | 0 | 7.290825 | 3.189856 | -0.032384 |
| 68 | 1 | 0 | 2.254446 | 8.055217 | 0.503693 |
| 69 | 1 | 0 | 5.346884 | 7.216655 | 1.450045 |
| 70 | 1 | 0 | 1.642918 | 9.074343 | -4.114619 |
| 71 | 1 | 0 | 2.502308 | 12.034597 | -2.964765 |
| 72 | 1 | 0 | 6.05546 | 12.154603 | -6.110424 |
| 73 | 1 | 0 | 5.952433 | 9.158386 | -9.731735 |
| 74 | 1 | 0 | 6.52586 | 7.567033 | -6.875809 |
| 75 | 1 | 0 | 3.498172 | 7.46205 | -8.265353 |
| 76 | 1 | 0 | 3.299617 | 13.160755 | -9.790182 |
| 77 | 1 | 0 | 1.98234 | 14.343634 | -6.973914 |
| 78 | 1 | 0 | 0.763469 | 11.568507 | -8.349878 |
| 79 | 1 | 0 | 3.572595 | -8.015957 | 8.046826 |

3 NMR calculations results

Table S7.Gibbs free energies^a^ and equilibrium populations^b^ of low-energy conformers of C1-1.

| Conformers | ∆G(a.u.) | P(%)/100 | G(a.u.) |
| --- | --- | --- | --- |
| M1000004.out | 0.00097 | 21.16 | -1469.925714 |
| M1000005.out | 0.00102 | 19.95 | -1469.925658 |
| M1000009.out | 0.0 | 58.89 | -1469.92668 |

^a^wB97M-V/def2-TZVP, in a.u.
^b^From ∆G values at 298.15K.

Table S8.Cartesian coordinates for the low-energy reoptimized random reseach conformers of M10 at B3LYP-D3(BJ)/6-31G** level of theory in methanol.

| M1000004_en_ | | Standard Orientation (A.U.) | | | |
| --- | --- | --- | --- | --- | --- |
| Center number | Atomic number | Atomic Type | X | Y | Z |
| 0 | 6 | 0 | -4.470388 | 4.005753 | -3.414281 |
| 1 | 6 | 0 | -8.103398 | -2.569244 | 2.781481 |
| 2 | 6 | 0 | -7.211626 | -1.8314 | 0.147282 |
| 3 | 6 | 0 | -5.411268 | 0.529121 | 0.068197 |
| 4 | 6 | 0 | -5.039091 | 1.223725 | -2.76437 |
| 5 | 6 | 0 | -6.013534 | -4.065573 | -1.277472 |
| 6 | 6 | 0 | -3.23084 | -4.335744 | -0.870709 |
| 7 | 6 | 0 | -1.766305 | -2.608256 | 0.244181 |
| 8 | 6 | 0 | -2.88403 | -0.222721 | 1.355152 |
| 9 | 6 | 0 | 1.087805 | -3.029131 | 0.380256 |
| 10 | 6 | 0 | 2.419512 | -1.276066 | 2.225075 |
| 11 | 6 | 0 | 1.442452 | 0.971576 | 2.795745 |
| 12 | 6 | 0 | -0.899749 | 1.898616 | 1.492011 |
| 13 | 6 | 0 | 1.828711 | -5.635278 | 1.470532 |
| 14 | 6 | 0 | 4.575055 | -5.294642 | 2.305179 |
| 15 | 6 | 0 | 4.769014 | -2.545692 | 3.349681 |
| 16 | 6 | 0 | -6.655556 | 2.707279 | 1.512531 |
| 17 | 6 | 0 | -10.729613 | -1.727197 | 3.476528 |
| 18 | 6 | 0 | -6.739092 | -3.985284 | 4.362832 |
| 19 | 6 | 0 | 7.264852 | -1.29712 | 2.379744 |
| 20 | 6 | 0 | 9.629622 | -2.835023 | 3.047817 |
| 21 | 6 | 0 | 7.666719 | 1.469849 | 3.205898 |
| 22 | 6 | 0 | 8.622017 | 3.150639 | 1.017088 |
| 23 | 6 | 0 | 6.578155 | 3.559136 | -0.87659 |
| 24 | 6 | 0 | 6.451962 | 2.732931 | -3.273078 |
| 25 | 6 | 0 | 8.467366 | 1.214722 | -4.586604 |
| 26 | 1 | 0 | -3.358537 | -0.652975 | 3.340221 |
| 27 | 6 | 0 | 2.175069 | -2.677119 | -2.311222 |
| 28 | 6 | 0 | 4.624053 | -2.549854 | 6.246643 |
| 29 | 6 | 0 | -4.250825 | 4.24197 | -6.269998 |
| 30 | 8 | 0 | -6.067633 | 4.121471 | -7.648178 |
| 31 | 8 | 0 | -1.912439 | 4.521215 | -7.24093 |
| 32 | 6 | 0 | 4.090071 | 3.324938 | -4.685426 |
| 33 | 8 | 0 | 2.289369 | 4.47416 | -3.821486 |
| 34 | 8 | 0 | 3.932328 | 2.492165 | -7.094777 |
| 35 | 1 | 0 | -6.059218 | 5.202383 | -2.849449 |
| 36 | 1 | 0 | -2.770867 | 4.701524 | -2.467181 |
| 37 | 1 | 0 | -8.91543 | -1.262697 | -0.898698 |
| 38 | 1 | 0 | -6.767962 | 0.74246 | -3.804053 |
| 39 | 1 | 0 | -3.536556 | 0.026886 | -3.548791 |
| 40 | 1 | 0 | -6.351332 | -3.854194 | -3.320334 |
| 41 | 1 | 0 | -6.982994 | -5.817774 | -0.732934 |
| 42 | 1 | 0 | -2.373746 | -6.043974 | -1.641465 |
| 43 | 1 | 0 | 2.270018 | 2.175262 | 4.240116 |
| 44 | 1 | 0 | -1.672154 | 3.528276 | 2.502046 |
| 45 | 1 | 0 | -0.39722 | 2.553628 | -0.408957 |
| 46 | 1 | 0 | 1.592388 | -7.180684 | 0.113626 |
| 47 | 1 | 0 | 0.612056 | -6.037655 | 3.100705 |
| 48 | 1 | 0 | 5.165646 | -6.700687 | 3.70451 |
| 49 | 1 | 0 | 5.819994 | -5.516657 | 0.663969 |
| 50 | 1 | 0 | -6.899095 | 2.211983 | 3.507211 |
| 51 | 1 | 0 | -5.53359 | 4.43886 | 1.426314 |
| 52 | 1 | 0 | -8.51823 | 3.139922 | 0.71473 |
| 53 | 1 | 0 | -12.113105 | -2.598496 | 2.198279 |
| 54 | 1 | 0 | -11.213307 | -2.260571 | 5.413402 |
| 55 | 1 | 0 | -10.960591 | 0.320408 | 3.278818 |
| 56 | 1 | 0 | -7.469042 | -4.496454 | 6.213254 |
| 57 | 1 | 0 | -4.889529 | -4.710882 | 3.84669 |
| 58 | 1 | 0 | 7.087117 | -1.298133 | 0.308683 |
| 59 | 1 | 0 | 11.313191 | -1.944276 | 2.231689 |
| 60 | 1 | 0 | 9.547323 | -4.773977 | 2.338689 |
| 61 | 1 | 0 | 9.914699 | -2.912837 | 5.096436 |
| 62 | 1 | 0 | 5.921516 | 2.301381 | 3.923506 |
| 63 | 1 | 0 | 9.040046 | 1.553699 | 4.756776 |
| 64 | 1 | 0 | 9.167262 | 4.999401 | 1.790103 |
| 65 | 1 | 0 | 10.323944 | 2.322233 | 0.179387 |
| 66 | 1 | 0 | 4.935486 | 4.595674 | -0.192379 |
| 67 | 1 | 0 | 10.069058 | 0.852434 | -3.342055 |
| 68 | 1 | 0 | 7.754574 | -0.641874 | -5.186723 |
| 69 | 1 | 0 | 9.216492 | 2.197993 | -6.254691 |
| 70 | 1 | 0 | 1.679681 | -0.803643 | -3.031843 |
| 71 | 1 | 0 | 4.236563 | -2.862145 | -2.342038 |
| 72 | 1 | 0 | 1.365788 | -4.084714 | -3.594312 |
| 73 | 1 | 0 | 2.87696 | -3.470104 | 6.864621 |
| 74 | 1 | 0 | 6.216901 | -3.575618 | 7.080235 |
| 75 | 1 | 0 | 4.616675 | -0.630124 | 7.019282 |
| 76 | 1 | 0 | -0.605679 | 4.554373 | -5.929822 |
| 77 | 1 | 0 | 5.44179 | 1.585202 | -7.577998 |

| M1000005_en_ | | Standard Orientation (A.U.) | | | |
| --- | --- | --- | --- | --- | --- |
| Center number | Atomic number | Atomic Type | X | Y | Z |
| 0 | 6 | 0 | -3.985622 | 3.432436 | -4.182421 |
| 1 | 6 | 0 | -7.930685 | -1.628412 | 3.136936 |
| 2 | 6 | 0 | -7.124345 | -1.417923 | 0.383145 |
| 3 | 6 | 0 | -5.077618 | 0.666777 | -0.147996 |
| 4 | 6 | 0 | -4.810837 | 0.87038 | -3.069489 |
| 5 | 6 | 0 | -6.275002 | -3.970651 | -0.721365 |
| 6 | 6 | 0 | -3.520042 | -4.511154 | -0.402704 |
| 7 | 6 | 0 | -1.803402 | -2.825703 | 0.360644 |
| 8 | 6 | 0 | -2.57649 | -0.182278 | 1.129796 |
| 9 | 6 | 0 | 0.987949 | -3.56085 | 0.45247 |
| 10 | 6 | 0 | 2.597569 | -1.731971 | 1.972818 |
| 11 | 6 | 0 | 1.929261 | 0.684987 | 2.192734 |
| 12 | 6 | 0 | -0.354066 | 1.668005 | 0.824957 |
| 13 | 6 | 0 | 1.473834 | -6.056725 | 1.888578 |
| 14 | 6 | 0 | 4.269392 | -5.928118 | 2.611578 |
| 15 | 6 | 0 | 4.826231 | -3.098648 | 3.218672 |
| 16 | 6 | 0 | -5.971978 | 3.174628 | 0.987728 |
| 17 | 6 | 0 | -10.398527 | -0.378656 | 3.798003 |
| 18 | 6 | 0 | -6.639388 | -2.936109 | 4.865801 |
| 19 | 6 | 0 | 7.406793 | -2.319226 | 2.00908 |
| 20 | 6 | 0 | 9.603155 | -4.016772 | 2.847807 |
| 21 | 6 | 0 | 8.170542 | 0.471537 | 2.405148 |
| 22 | 6 | 0 | 9.032986 | 1.797939 | -0.053814 |
| 23 | 6 | 0 | 6.852383 | 2.328862 | -1.753734 |
| 24 | 6 | 0 | 5.912879 | 4.591586 | -2.428303 |
| 25 | 6 | 0 | 6.948694 | 7.113304 | -1.610652 |
| 26 | 1 | 0 | -2.965808 | -0.237895 | 3.178077 |
| 27 | 6 | 0 | 1.991491 | -3.724155 | -2.290115 |
| 28 | 6 | 0 | 4.803283 | -2.65946 | 6.085778 |
| 29 | 6 | 0 | -3.92228 | 3.187607 | -7.045363 |
| 30 | 8 | 0 | -5.81847 | 3.237124 | -8.31692 |
| 31 | 8 | 0 | -1.64917 | 2.806216 | -8.128966 |
| 32 | 6 | 0 | 3.6143 | 4.554124 | -4.044765 |
| 33 | 8 | 0 | 2.699546 | 2.666558 | -4.994142 |
| 34 | 8 | 0 | 2.434052 | 6.789436 | -4.455175 |
| 35 | 1 | 0 | -5.390472 | 4.884159 | -3.745935 |
| 36 | 1 | 0 | -2.159176 | 4.05834 | -3.444152 |
| 37 | 1 | 0 | -8.81386 | -0.813319 | -0.665709 |
| 38 | 1 | 0 | -6.646155 | 0.441902 | -3.935298 |
| 39 | 1 | 0 | -3.501574 | -0.600808 | -3.720964 |
| 40 | 1 | 0 | -6.716779 | -4.032434 | -2.754435 |
| 41 | 1 | 0 | -7.399503 | -5.489927 | 0.135539 |
| 42 | 1 | 0 | -2.910739 | -6.406449 | -0.933589 |
| 43 | 1 | 0 | 2.967128 | 1.994054 | 3.387977 |
| 44 | 1 | 0 | -0.871791 | 3.533683 | 1.550783 |
| 45 | 1 | 0 | 0.097732 | 1.894197 | -1.183828 |
| 46 | 1 | 0 | 1.021656 | -7.73506 | 0.764668 |
| 47 | 1 | 0 | 0.275029 | -6.092775 | 3.580161 |
| 48 | 1 | 0 | 4.738516 | -7.166778 | 4.202125 |
| 49 | 1 | 0 | 5.418825 | -6.551194 | 1.005022 |
| 50 | 1 | 0 | -6.139611 | 3.020557 | 3.044824 |
| 51 | 1 | 0 | -4.668694 | 4.723117 | 0.575452 |
| 52 | 1 | 0 | -7.821088 | 3.706355 | 0.21906 |
| 53 | 1 | 0 | -10.821834 | -0.546722 | 5.813678 |
| 54 | 1 | 0 | -10.409422 | 1.627482 | 3.286887 |
| 55 | 1 | 0 | -11.945966 | -1.261834 | 2.73386 |
| 56 | 1 | 0 | -7.307535 | -3.06506 | 6.80353 |
| 57 | 1 | 0 | -4.91702 | -3.94784 | 4.393259 |
| 58 | 1 | 0 | 7.154314 | -2.6222 | -0.031314 |
| 59 | 1 | 0 | 11.334984 | -3.480874 | 1.844802 |
| 60 | 1 | 0 | 9.25613 | -6.016755 | 2.463697 |
| 61 | 1 | 0 | 9.976473 | -3.806163 | 4.872873 |
| 62 | 1 | 0 | 6.627453 | 1.577184 | 3.210064 |
| 63 | 1 | 0 | 9.729188 | 0.571598 | 3.76858 |
| 64 | 1 | 0 | 10.071712 | 3.521681 | 0.417239 |
| 65 | 1 | 0 | 10.362033 | 0.545198 | -1.048518 |
| 66 | 1 | 0 | 5.843077 | 0.688838 | -2.478691 |
| 67 | 1 | 0 | 5.628538 | 8.124265 | -0.364369 |
| 68 | 1 | 0 | 8.701885 | 6.901543 | -0.550173 |
| 69 | 1 | 0 | 7.376565 | 8.332108 | -3.234298 |
| 70 | 1 | 0 | 0.922702 | -5.140813 | -3.354727 |
| 71 | 1 | 0 | 1.785462 | -1.906047 | -3.254108 |
| 72 | 1 | 0 | 3.992731 | -4.251645 | -2.34389 |
| 73 | 1 | 0 | 5.029729 | -0.658338 | 6.560728 |
| 74 | 1 | 0 | 2.995905 | -3.279767 | 6.879406 |
| 75 | 1 | 0 | 6.312352 | -3.722158 | 7.022612 |
| 76 | 1 | 0 | -0.270725 | 2.840876 | -6.892561 |
| 77 | 1 | 0 | 3.300861 | 8.152803 | -3.603397 |

| M1000009_en_ | | Standard Orientation (A.U.) | | | |
| --- | --- | --- | --- | --- | --- |
| Center number | Atomic number | Atomic Type | X | Y | Z |
| 0 | 6 | 0 | -3.443288 | 3.274538 | -3.453431 |
| 1 | 6 | 0 | -8.195395 | -2.245739 | 3.159358 |
| 2 | 6 | 0 | -7.331354 | -1.67125 | 0.4792 |
| 3 | 6 | 0 | -5.484963 | 0.652456 | 0.25386 |
| 4 | 6 | 0 | -5.284753 | 1.224934 | -2.613338 |
| 5 | 6 | 0 | -6.184309 | -4.014174 | -0.832921 |
| 6 | 6 | 0 | -3.367885 | -4.215989 | -0.655169 |
| 7 | 6 | 0 | -1.851189 | -2.461076 | 0.346544 |
| 8 | 6 | 0 | -2.967815 | -0.124567 | 1.547119 |
| 9 | 6 | 0 | 1.006452 | -2.835654 | 0.33984 |
| 10 | 6 | 0 | 2.362117 | -1.215316 | 2.288406 |
| 11 | 6 | 0 | 1.362709 | 0.94121 | 3.120299 |
| 12 | 6 | 0 | -1.023939 | 1.994304 | 2.006986 |
| 13 | 6 | 0 | 1.828297 | -5.508703 | 1.183909 |
| 14 | 6 | 0 | 4.586529 | -5.189492 | 1.989128 |
| 15 | 6 | 0 | 4.783133 | -2.51349 | 3.20795 |
| 16 | 6 | 0 | -6.62308 | 2.946038 | 1.606719 |
| 17 | 6 | 0 | -10.735125 | -1.212671 | 3.913193 |
| 18 | 6 | 0 | -6.868347 | -3.698625 | 4.74085 |
| 19 | 6 | 0 | 7.206864 | -1.156587 | 2.195575 |
| 20 | 6 | 0 | 9.630898 | -2.670492 | 2.682817 |
| 21 | 6 | 0 | 7.58315 | 1.571119 | 3.139244 |
| 22 | 6 | 0 | 8.624023 | 3.319753 | 1.044362 |
| 23 | 6 | 0 | 6.637657 | 3.826127 | -0.887233 |
| 24 | 6 | 0 | 6.541902 | 3.039749 | -3.298798 |
| 25 | 6 | 0 | 8.528597 | 1.457429 | -4.580258 |
| 26 | 1 | 0 | -3.516869 | -0.68929 | 3.478275 |
| 27 | 6 | 0 | 1.996901 | -2.251722 | -2.346461 |
| 28 | 6 | 0 | 4.79355 | -2.701877 | 6.101917 |
| 29 | 6 | 0 | -3.877963 | 4.127446 | -6.164214 |
| 30 | 8 | 0 | -5.817209 | 3.81075 | -7.321193 |
| 31 | 8 | 0 | -1.917127 | 5.367959 | -7.22779 |
| 32 | 6 | 0 | 4.245954 | 3.754904 | -4.763971 |
| 33 | 8 | 0 | 2.524025 | 5.05735 | -3.964388 |
| 34 | 8 | 0 | 4.062964 | 2.86456 | -7.154051 |
| 35 | 1 | 0 | -3.583007 | 4.986867 | -2.28604 |
| 36 | 1 | 0 | -1.486269 | 2.629707 | -3.294463 |
| 37 | 1 | 0 | -9.036856 | -1.133204 | -0.580946 |
| 38 | 1 | 0 | -7.175745 | 1.750532 | -3.281438 |
| 39 | 1 | 0 | -4.774657 | -0.497159 | -3.636366 |
| 40 | 1 | 0 | -6.717883 | -4.019759 | -2.842501 |
| 41 | 1 | 0 | -7.058819 | -5.721344 | -0.039036 |
| 42 | 1 | 0 | -2.544463 | -5.917796 | -1.474998 |
| 43 | 1 | 0 | 2.235736 | 2.001457 | 4.649355 |
| 44 | 1 | 0 | -1.837763 | 3.390048 | 3.29861 |
| 45 | 1 | 0 | -0.553365 | 3.021748 | 0.273896 |
| 46 | 1 | 0 | 1.597746 | -6.935472 | -0.298311 |
| 47 | 1 | 0 | 0.652889 | -6.07827 | 2.793675 |
| 48 | 1 | 0 | 5.212352 | -6.67824 | 3.283619 |
| 49 | 1 | 0 | 5.805293 | -5.298509 | 0.316943 |
| 50 | 1 | 0 | -6.751902 | 2.623195 | 3.647826 |
| 51 | 1 | 0 | -5.487232 | 4.648641 | 1.31703 |
| 52 | 1 | 0 | -8.521258 | 3.344065 | 0.880983 |
| 53 | 1 | 0 | -10.851949 | 0.836238 | 3.64927 |
| 54 | 1 | 0 | -12.21536 | -2.042591 | 2.718678 |
| 55 | 1 | 0 | -11.177 | -1.647496 | 5.884425 |
| 56 | 1 | 0 | -7.548879 | -4.096048 | 6.637128 |
| 57 | 1 | 0 | -5.093163 | -4.566288 | 4.179043 |
| 58 | 1 | 0 | 6.935973 | -1.053102 | 0.137506 |
| 59 | 1 | 0 | 11.259853 | -1.710112 | 1.835764 |
| 60 | 1 | 0 | 9.554822 | -4.576323 | 1.888105 |
| 61 | 1 | 0 | 10.011361 | -2.837396 | 4.710245 |
| 62 | 1 | 0 | 5.805044 | 2.38144 | 3.79906 |
| 63 | 1 | 0 | 8.89073 | 1.597018 | 4.748263 |
| 64 | 1 | 0 | 9.169263 | 5.131622 | 1.899129 |
| 65 | 1 | 0 | 10.337959 | 2.503018 | 0.22035 |
| 66 | 1 | 0 | 5.01551 | 4.910213 | -0.226759 |
| 67 | 1 | 0 | 7.771326 | -0.384004 | -5.174638 |
| 68 | 1 | 0 | 9.322216 | 2.406283 | -6.247005 |
| 69 | 1 | 0 | 10.103859 | 1.056164 | -3.313835 |
| 70 | 1 | 0 | 1.55366 | -0.298747 | -2.862984 |
| 71 | 1 | 0 | 4.04797 | -2.500163 | -2.479378 |
| 72 | 1 | 0 | 1.088779 | -3.50231 | -3.722933 |
| 73 | 1 | 0 | 3.092568 | -3.683769 | 6.752648 |
| 74 | 1 | 0 | 6.440824 | -3.751949 | 6.785348 |
| 75 | 1 | 0 | 4.807759 | -0.833059 | 6.990877 |
| 76 | 1 | 0 | -0.444674 | 5.361158 | -6.106377 |
| 77 | 1 | 0 | 5.505907 | 1.830761 | -7.583135 |

Table S9.Gibbs free energies^a^ and equilibrium populations^b^ of low-energy conformers of C1-2.

| Conformers | ∆G(a.u.) | P(%)/100 | G(a.u.) |
| --- | --- | --- | --- |
| M1-2000009.out | 0.0000 | 100.0 | -1469.916403 |

^a^wB97M-V/def2-TZVP, in a.u.
^b^From ∆G values at 298.15K.

Table S10.Cartesian coordinates for the low-energy reoptimized random reseach conformers of C1-2 at B3LYP-D3(BJ)/6-31G** level of theory in methanol.

| C1-2000009_en_ | | Standard Orientation (A.U.) | | | |
| --- | --- | --- | --- | --- | --- |
| Center number | Atomic number | Atomic Type | X | Y | Z |
| 0 | 6 | 0 | -9.341998 | -1.369442 | 0.625477 |
| 1 | 6 | 0 | -5.383936 | -8.35107 | -1.342652 |
| 2 | 6 | 0 | -3.719556 | -6.022099 | -1.116965 |
| 3 | 6 | 0 | -4.91638 | -3.708528 | 0.28602 |
| 4 | 6 | 0 | -7.624308 | -3.302583 | -0.73722 |
| 5 | 6 | 0 | -1.135665 | -6.643604 | 0.036898 |
| 6 | 6 | 0 | 0.486193 | -4.341182 | 0.280861 |
| 7 | 6 | 0 | -0.389091 | -1.979163 | 0.124904 |
| 8 | 6 | 0 | -3.16649 | -1.390453 | -0.137894 |
| 9 | 6 | 0 | 1.412947 | 0.292186 | -0.058943 |
| 10 | 6 | 0 | -0.008893 | 2.709018 | -0.727007 |
| 11 | 6 | 0 | -2.129059 | 2.653357 | -2.083419 |
| 12 | 6 | 0 | -3.421607 | 0.166393 | -2.612431 |
| 13 | 6 | 0 | 2.747357 | 0.979167 | 2.483147 |
| 14 | 6 | 0 | 1.694944 | 3.577907 | 3.242764 |
| 15 | 6 | 0 | 1.04994 | 4.952427 | 0.730567 |
| 16 | 6 | 0 | -5.063434 | -4.189289 | 3.14853 |
| 17 | 6 | 0 | -6.50618 | -8.812533 | -3.913364 |
| 18 | 6 | 0 | -5.782531 | -9.968595 | 0.552171 |
| 19 | 6 | 0 | 3.509812 | 6.032318 | -0.480952 |
| 20 | 6 | 0 | 3.177172 | 6.719224 | -3.269597 |
| 21 | 6 | 0 | 4.606354 | 8.293947 | 0.990482 |
| 22 | 6 | 0 | 7.524182 | 8.370116 | 0.872866 |
| 23 | 6 | 0 | 8.613049 | 6.284227 | 2.42889 |
| 24 | 6 | 0 | 9.726278 | 4.151298 | 1.616146 |
| 25 | 6 | 0 | 10.225586 | 3.452336 | -1.087228 |
| 26 | 1 | 0 | -3.6417 | -0.036522 | 1.365995 |
| 27 | 6 | 0 | 3.379016 | -0.268802 | -2.151764 |
| 28 | 6 | 0 | -0.921117 | 7.027923 | 1.133127 |
| 29 | 6 | 0 | -8.67469 | 1.391729 | 0.271156 |
| 30 | 8 | 0 | -7.521913 | 2.665165 | 1.760429 |
| 31 | 8 | 0 | -9.495515 | 2.464283 | -1.935531 |
| 32 | 6 | 0 | 10.555264 | 2.20797 | 3.455474 |
| 33 | 8 | 0 | 11.534696 | 0.221076 | 2.854501 |
| 34 | 8 | 0 | 10.114852 | 2.804336 | 5.921252 |
| 35 | 1 | 0 | -11.284447 | -1.67086 | -0.044553 |
| 36 | 1 | 0 | -9.348045 | -1.734506 | 2.655941 |
| 37 | 1 | 0 | -3.353948 | -5.355462 | -3.055552 |
| 38 | 1 | 0 | -8.600438 | -5.12205 | -0.557338 |
| 39 | 1 | 0 | -7.552929 | -2.881138 | -2.768451 |
| 40 | 1 | 0 | -0.179769 | -8.0234 | -1.185162 |
| 41 | 1 | 0 | -1.363225 | -7.612158 | 1.860381 |
| 42 | 1 | 0 | 2.509448 | -4.664665 | 0.476056 |
| 43 | 1 | 0 | -3.165025 | 4.379052 | -2.498495 |
| 44 | 1 | 0 | -5.388609 | 0.53783 | -3.11006 |
| 45 | 1 | 0 | -2.58518 | -0.873788 | -4.205447 |
| 46 | 1 | 0 | 4.799789 | 1.072872 | 2.201914 |
| 47 | 1 | 0 | 2.399804 | -0.453696 | 3.931656 |
| 48 | 1 | 0 | -0.063047 | 3.339675 | 4.31784 |
| 49 | 1 | 0 | 3.010194 | 4.64169 | 4.437828 |
| 50 | 1 | 0 | -3.208607 | -4.649994 | 3.929273 |
| 51 | 1 | 0 | -5.710132 | -2.488089 | 4.129775 |
| 52 | 1 | 0 | -6.371336 | -5.731227 | 3.589701 |
| 53 | 1 | 0 | -7.602249 | -7.180055 | -4.569036 |
| 54 | 1 | 0 | -4.994264 | -9.09498 | -5.306457 |
| 55 | 1 | 0 | -7.729047 | -10.478072 | -3.934258 |
| 56 | 1 | 0 | -6.972659 | -11.621815 | 0.294171 |
| 57 | 1 | 0 | -4.932822 | -9.724434 | 2.402209 |
| 58 | 1 | 0 | 4.922786 | 4.520337 | -0.368066 |
| 59 | 1 | 0 | 1.768152 | 8.218268 | -3.507651 |
| 60 | 1 | 0 | 4.955422 | 7.410227 | -4.077018 |
| 61 | 1 | 0 | 2.557118 | 5.091855 | -4.38245 |
| 62 | 1 | 0 | 3.847342 | 10.067724 | 0.22918 |
| 63 | 1 | 0 | 4.048258 | 8.22491 | 2.984769 |
| 64 | 1 | 0 | 8.156706 | 8.288662 | -1.094354 |
| 65 | 1 | 0 | 8.175306 | 10.182815 | 1.646059 |
| 66 | 1 | 0 | 8.367249 | 6.504528 | 4.459569 |
| 67 | 1 | 0 | 9.363602 | 1.623186 | -1.526788 |
| 68 | 1 | 0 | 9.49283 | 4.862123 | -2.400285 |
| 69 | 1 | 0 | 12.257238 | 3.226568 | -1.412585 |
| 70 | 1 | 0 | 4.619613 | -1.829405 | -1.59939 |
| 71 | 1 | 0 | 2.431272 | -0.787396 | -3.915451 |
| 72 | 1 | 0 | 4.568326 | 1.379759 | -2.518103 |
| 73 | 1 | 0 | -2.66919 | 6.215814 | 1.882859 |
| 74 | 1 | 0 | -0.24052 | 8.448606 | 2.474394 |
| 75 | 1 | 0 | -1.388478 | 8.005099 | -0.630265 |
| 76 | 1 | 0 | -10.402594 | 1.225868 | -2.925182 |
| 77 | 1 | 0 | 10.729262 | 1.376067 | 6.888943 |

Table S11.Gibbs free energies^a^ and equilibrium populations^b^ of low-energy conformers of C2-1.

| Conformers | ∆G(a.u.) | P(%)/100 | G(a.u.) |
| --- | --- | --- | --- |
| M2000004.out | 0.0 | 100.0 | -1469.916097 |

^a^wB97M-V/def2-TZVP, in a.u.
^b^From ∆G values at 298.15K.

Table S12.Cartesian coordinates for the low-energy reoptimized random reseach conformers of C2-1 at B3LYP-D3(BJ)/6-31G** level of theory in methanol.

| M2000004_en_ | | Standard Orientation (A.U.) | | | |
| --- | --- | --- | --- | --- | --- |
| Center number | Atomic number | Atomic Type | X | Y | Z |
| 0 | 1 | 0 | -4.378904 | -0.190529 | 2.201491 |
| 1 | 6 | 0 | -6.161545 | -6.561683 | -2.768358 |
| 2 | 6 | 0 | -7.155515 | -3.587603 | 5.39252 |
| 3 | 6 | 0 | -6.639685 | -4.699122 | 2.765702 |
| 4 | 6 | 0 | -6.908799 | -2.776601 | 0.521244 |
| 5 | 6 | 0 | -7.639469 | -4.213331 | -1.934916 |
| 6 | 6 | 0 | -4.105012 | -5.980144 | 2.79712 |
| 7 | 6 | 0 | -2.040035 | -5.0556 | 1.650156 |
| 8 | 6 | 0 | -2.052589 | -2.72074 | 0.213686 |
| 9 | 6 | 0 | -4.456565 | -1.164701 | 0.356339 |
| 10 | 6 | 0 | 0.038068 | -1.877978 | -0.977689 |
| 11 | 6 | 0 | 0.265314 | 0.63113 | -2.354021 |
| 12 | 6 | 0 | -1.963336 | 2.353485 | -1.651745 |
| 13 | 6 | 0 | -4.471768 | 0.906486 | -1.657095 |
| 14 | 6 | 0 | 2.609172 | -3.118083 | -0.889807 |
| 15 | 6 | 0 | 4.478199 | -1.016804 | -1.666317 |
| 16 | 6 | 0 | 2.985795 | 1.49638 | -1.449275 |
| 17 | 6 | 0 | 0.20606 | 0.140997 | -5.229016 |
| 18 | 6 | 0 | -9.144586 | -0.984257 | 1.01697 |
| 19 | 6 | 0 | -9.742584 | -4.037917 | 6.475787 |
| 20 | 6 | 0 | -5.373283 | -2.386665 | 6.711534 |
| 21 | 6 | 0 | 4.304783 | 3.736447 | -2.848982 |
| 22 | 6 | 0 | 2.623598 | 6.074999 | -3.189481 |
| 23 | 6 | 0 | 5.66921 | 3.12241 | -5.364946 |
| 24 | 6 | 0 | 8.531046 | 2.637597 | -5.040794 |
| 25 | 6 | 0 | 9.877063 | 4.982942 | -4.252126 |
| 26 | 6 | 0 | 10.962846 | 5.505698 | -2.020751 |
| 27 | 6 | 0 | 11.067284 | 3.720541 | 0.192912 |
| 28 | 6 | 0 | 2.897139 | 2.190745 | 1.382141 |
| 29 | 6 | 0 | -3.84147 | -6.005529 | -4.355036 |
| 30 | 8 | 0 | -3.843369 | -4.542257 | -6.099422 |
| 31 | 8 | 0 | -1.746036 | -7.407837 | -3.860428 |
| 32 | 6 | 0 | 12.125341 | 8.0803 | -1.770356 |
| 33 | 8 | 0 | 12.100094 | 9.684352 | -3.389658 |
| 34 | 8 | 0 | 13.282041 | 8.598733 | 0.477242 |
| 35 | 1 | 0 | -5.73672 | -7.803849 | -1.172419 |
| 36 | 1 | 0 | -7.382301 | -7.645135 | -4.053419 |
| 37 | 1 | 0 | -8.100818 | -6.156441 | 2.468216 |
| 38 | 1 | 0 | -9.608708 | -4.810888 | -1.675491 |
| 39 | 1 | 0 | -7.654229 | -2.882408 | -3.521659 |
| 40 | 1 | 0 | -3.960523 | -7.687345 | 3.937669 |
| 41 | 1 | 0 | -0.265804 | -6.081223 | 1.829293 |
| 42 | 1 | 0 | -2.0923 | 3.939755 | -2.973033 |
| 43 | 1 | 0 | -1.686466 | 3.157405 | 0.237628 |
| 44 | 1 | 0 | -6.003137 | 2.248189 | -1.287557 |
| 45 | 1 | 0 | -4.816134 | 0.087628 | -3.528664 |
| 46 | 1 | 0 | 3.027612 | -3.825445 | 1.010872 |
| 47 | 1 | 0 | 2.711139 | -4.746652 | -2.170754 |
| 48 | 1 | 0 | 5.125666 | -1.333644 | -3.600385 |
| 49 | 1 | 0 | 6.163222 | -0.998427 | -0.460873 |
| 50 | 1 | 0 | 1.588218 | -1.278969 | -5.814302 |
| 51 | 1 | 0 | -1.635137 | -0.591389 | -5.795255 |
| 52 | 1 | 0 | 0.56746 | 1.886333 | -6.278984 |
| 53 | 1 | 0 | -10.879032 | -2.056877 | 1.364938 |
| 54 | 1 | 0 | -8.788853 | 0.234482 | 2.650411 |
| 55 | 1 | 0 | -9.50081 | 0.214282 | -0.628943 |
| 56 | 1 | 0 | -9.943008 | -3.171447 | 8.340477 |
| 57 | 1 | 0 | -11.238296 | -3.302662 | 5.249359 |
| 58 | 1 | 0 | -10.084891 | -6.075423 | 6.669355 |
| 59 | 1 | 0 | -5.780782 | -1.602328 | 8.564527 |
| 60 | 1 | 0 | -3.459866 | -2.180479 | 6.000446 |
| 61 | 1 | 0 | 5.809685 | 4.327258 | -1.538188 |
| 62 | 1 | 0 | 1.575436 | 6.55702 | -1.475056 |
| 63 | 1 | 0 | 1.258277 | 5.823527 | -4.722716 |
| 64 | 1 | 0 | 3.802707 | 7.705566 | -3.678045 |
| 65 | 1 | 0 | 4.828648 | 1.499681 | -6.323178 |
| 66 | 1 | 0 | 5.438265 | 4.724341 | -6.662881 |
| 67 | 1 | 0 | 8.838431 | 1.079345 | -3.718042 |
| 68 | 1 | 0 | 9.301697 | 2.034826 | -6.872903 |
| 69 | 1 | 0 | 9.935225 | 6.496743 | -5.648762 |
| 70 | 1 | 0 | 13.020007 | 3.238148 | 0.709758 |
| 71 | 1 | 0 | 10.132696 | 4.522227 | 1.865224 |
| 72 | 1 | 0 | 10.105537 | 1.945039 | -0.22305 |
| 73 | 1 | 0 | 4.823762 | 2.267331 | 2.139568 |
| 74 | 1 | 0 | 2.032562 | 4.037491 | 1.718464 |
| 75 | 1 | 0 | 1.836159 | 0.79272 | 2.480512 |
| 76 | 1 | 0 | -1.969077 | -8.251968 | -2.253113 |
| 77 | 1 | 0 | 13.177969 | 7.163056 | 1.598509 |

Table S13.Gibbs free energies^a^ and equilibrium populations^b^ of low-energy conformers of C2-2.

| Conformers | ∆G(a.u.) | P(%)/100 | G(a.u.) |
| --- | --- | --- | --- |
| M2B000008.out | 0.0 | 100.0 | -1469.9237 |

^a^wB97M-V/def2-TZVP, in a.u.
^b^From ∆G values at 298.15K.

Table S14 Cartesian coordinates for the low-energy reoptimized random reseach conformers of C2-2 at B3LYP-D3(BJ)/6-31G** level of theory in methanol.

| M2B000008_en_ | | Standard Orientation (A.U.) | | | |
| --- | --- | --- | --- | --- | --- |
| Center number | Atomic number | Atomic Type | X | Y | Z |
| 0 | 1 | 0 | -5.40476 | -1.571295 | 1.602822 |
| 1 | 6 | 0 | -5.687267 | -4.749511 | -6.027779 |
| 2 | 6 | 0 | -8.228757 | -6.171851 | 2.225393 |
| 3 | 6 | 0 | -7.20314 | -5.853752 | -0.46669 |
| 4 | 6 | 0 | -7.343451 | -3.088013 | -1.534344 |
| 5 | 6 | 0 | -7.543164 | -3.168289 | -4.463532 |
| 6 | 6 | 0 | -4.577097 | -6.927022 | -0.58195 |
| 7 | 6 | 0 | -2.478728 | -5.511645 | -0.748996 |
| 8 | 6 | 0 | -2.531515 | -2.773081 | -0.866748 |
| 9 | 6 | 0 | -5.088581 | -1.539294 | -0.460435 |
| 10 | 6 | 0 | -0.393245 | -1.403833 | -1.104454 |
| 11 | 6 | 0 | -0.239289 | 1.460157 | -1.031548 |
| 12 | 6 | 0 | -2.737636 | 2.568017 | -0.055105 |
| 13 | 6 | 0 | -5.021797 | 1.241042 | -1.241148 |
| 14 | 6 | 0 | 2.255615 | -2.451073 | -1.209757 |
| 15 | 6 | 0 | 3.970313 | -0.24358 | -0.37514 |
| 16 | 6 | 0 | 2.185698 | 1.840139 | 0.686915 |
| 17 | 6 | 0 | 0.251133 | 2.446716 | -3.734311 |
| 18 | 6 | 0 | -9.813403 | -1.820231 | -0.668694 |
| 19 | 6 | 0 | -10.879417 | -7.165444 | 2.463096 |
| 20 | 6 | 0 | -6.814911 | -5.709192 | 4.261249 |
| 21 | 6 | 0 | 3.469562 | 4.470487 | 0.482 |
| 22 | 6 | 0 | 1.753052 | 6.705168 | 1.149834 |
| 23 | 6 | 0 | 5.906286 | 4.59599 | 2.080092 |
| 24 | 6 | 0 | 7.80743 | 6.578665 | 1.106122 |
| 25 | 6 | 0 | 10.066927 | 6.718215 | 2.790003 |
| 26 | 6 | 0 | 12.22176 | 5.403699 | 2.551898 |
| 27 | 6 | 0 | 12.737719 | 3.543377 | 0.460894 |
| 28 | 6 | 0 | 1.580732 | 1.180657 | 3.450445 |
| 29 | 6 | 0 | -3.254935 | -3.398268 | -6.711648 |
| 30 | 8 | 0 | -3.191581 | -1.269587 | -7.518726 |
| 31 | 8 | 0 | -1.093905 | -4.781591 | -6.575595 |
| 32 | 6 | 0 | 14.190783 | 5.838563 | 4.54107 |
| 33 | 8 | 0 | 13.930511 | 7.189561 | 6.358601 |
| 34 | 8 | 0 | 16.428423 | 4.591223 | 4.225518 |
| 35 | 1 | 0 | -5.333226 | -6.590931 | -5.159372 |
| 36 | 1 | 0 | -6.574275 | -5.119976 | -7.869286 |
| 37 | 1 | 0 | -8.433286 | -7.014539 | -1.685538 |
| 38 | 1 | 0 | -9.440387 | -3.897884 | -4.876548 |
| 39 | 1 | 0 | -7.505446 | -1.234012 | -5.203086 |
| 40 | 1 | 0 | -4.416864 | -8.96964 | -0.387595 |
| 41 | 1 | 0 | -0.650881 | -6.454911 | -0.756386 |
| 42 | 1 | 0 | -2.842577 | 4.592621 | -0.465491 |
| 43 | 1 | 0 | -2.86862 | 2.360457 | 2.002523 |
| 44 | 1 | 0 | -6.742296 | 2.203472 | -0.612767 |
| 45 | 1 | 0 | -4.942839 | 1.405577 | -3.304788 |
| 46 | 1 | 0 | 2.476651 | -4.08405 | 0.042288 |
| 47 | 1 | 0 | 2.717046 | -3.10095 | -3.125406 |
| 48 | 1 | 0 | 5.055045 | 0.501603 | -1.974386 |
| 49 | 1 | 0 | 5.339333 | -0.861124 | 1.045411 |
| 50 | 1 | 0 | 1.960317 | 1.639337 | -4.570055 |
| 51 | 1 | 0 | -1.306867 | 1.937925 | -4.984585 |
| 52 | 1 | 0 | 0.442357 | 4.507156 | -3.738228 |
| 53 | 1 | 0 | -9.83989 | -1.532652 | 1.379927 |
| 54 | 1 | 0 | -10.059769 | 0.013843 | -1.58971 |
| 55 | 1 | 0 | -11.449058 | -2.975006 | -1.189128 |
| 56 | 1 | 0 | -12.247578 | -5.978044 | 1.4629 |
| 57 | 1 | 0 | -11.005505 | -9.055481 | 1.615804 |
| 58 | 1 | 0 | -11.459902 | -7.306479 | 4.44076 |
| 59 | 1 | 0 | -7.584162 | -5.932971 | 6.15153 |
| 60 | 1 | 0 | -4.8526 | -5.130093 | 4.106538 |
| 61 | 1 | 0 | 4.048518 | 4.685643 | -1.503125 |
| 62 | 1 | 0 | 0.84448 | 6.440551 | 2.990155 |
| 63 | 1 | 0 | 2.84277 | 8.461037 | 1.251157 |
| 64 | 1 | 0 | 0.268306 | 7.00012 | -0.252102 |
| 65 | 1 | 0 | 5.433191 | 5.013801 | 4.057342 |
| 66 | 1 | 0 | 6.874485 | 2.765839 | 2.087121 |
| 67 | 1 | 0 | 8.335367 | 6.100143 | -0.839461 |
| 68 | 1 | 0 | 6.907828 | 8.443715 | 1.037606 |
| 69 | 1 | 0 | 9.935802 | 7.949169 | 4.434521 |
| 70 | 1 | 0 | 11.103971 | 3.333456 | -0.78025 |
| 71 | 1 | 0 | 14.331269 | 4.130343 | -0.735166 |
| 72 | 1 | 0 | 13.155368 | 1.65421 | 1.212988 |
| 73 | 1 | 0 | 0.53747 | -0.605518 | 3.560407 |
| 74 | 1 | 0 | 3.322192 | 0.959264 | 4.544465 |
| 75 | 1 | 0 | 0.438316 | 2.625937 | 4.386561 |
| 76 | 1 | 0 | -1.432952 | -6.311475 | -5.631696 |
| 77 | 1 | 0 | 16.397368 | 3.596805 | 2.69581 |
